# Incremental diagnostic value of tumor habitat radiomics for risk stratification in thymic epithelial tumors

**DOI:** 10.3389/fonc.2025.1630485

**Published:** 2025-09-03

**Authors:** Yiqiao Wang, Zhe Shi, Qinliang Sun, Tianzuo Wang, Zhizhen Ran, Jinling Zhang

**Affiliations:** ^1^ Department of CT Diagnosis, Second Affiliated Hospital of Harbin Medical University, Harbin, China; ^2^ Department of Radiology, Heilongjiang Red Cross Sengong General Hospital, Harbin, China; ^3^ Department of Ultrasound, Second Affiliated Hospital of Harbin Medical University, Harbin, China

**Keywords:** thymic epithelial tumors, habitat radiomics, risk stratification, computed tomography, machine learning

## Abstract

**Purpose:**

To determine the incremental diagnostic value of habitat radiomics for risk stratification of thymic epithelial tumors (TETs) based on contrast-enhanced CT (CECT).

**Methods:**

This retrospective study included 220 patients with pathologically confirmed TETs (82 high-risk [B2/B3/thymic carcinoma] and 138 low-risk [A/AB/B1]) who underwent preoperative CECT. Tumors were segmented into 3 subregions (habitats) using k-means clustering, and radiomic features were extracted from both whole-tumor and subregions. After feature selection (variance threshold, reproducibility evaluation, XGBoost-based importance ranking, and recursive feature elimination), three machine learning models were developed (1): a conventional radiomics model (2), a habitat radiomics model, and (3) a combined model integrating both feature sets. Model performance was evaluated using ROC analysis, net reclassification improvement (NRI), integrated discrimination improvement (IDI), calibration metrics, and decision curve analysis (DCA).

**Results:**

The combined model demonstrated superior discrimination (AUC: 0.900) compared to the conventional (AUC: 0.819) and habitat (AUC: 0.734) radiomics models in the independent test set. Although DeLong’s test showed no statistically significant difference (p=0.161), the performance of combined model demonstrated incremental diagnostic value (NRI: 0.286; IDI: 0.209). Calibration and DCA confirmed its robustness and higher net benefit across decision thresholds. While the models’ training performance might suggest overfitting, their test results demonstrate generalizability.

**Conclusions:**

The habitat radiomics approach enables accurate risk stratification prediction in TETs and demonstrates potential as a clinically valuable tool to augment the performance of conventional radiomics models in routine practice.

## Introduction

Thymic epithelial tumors (TETs) represented the most prevalent primary neoplasms originating in the anterior mediastinum (1–3). According to the 2021 WHO classification, TETs were histologically stratified into six distinct subtypes: type A, AB, B1, B2, B3, and thymic carcinoma (TC) (4). The histopathological classification was one of the important prognostic factors, particularly in guiding postoperative therapeutic decision-making within the context of neoadjuvant treatment strategies (5). Clinically, these subtypes exhibited markedly divergent biological behaviors: types A, AB, and B1 TETs were associated with favorable prognoses and significantly lower recurrence rates, whereas types B2, B3, and TC demonstrated aggressive clinical courses with substantially elevated recurrence risks (6–8). TETs could be further classified into two prognostic groups: low-risk TETs (LRT, encompassing A, AB, and B1) and high-risk TETs (HRT, including B2, B3, and TC) (9–11). Reliable noninvasive risk stratification prior to therapeutic intervention was critical for prognostication and treatment optimization.

Among imaging modalities, contrast-enhanced computed tomography (CECT) was the first choice for evaluating TETs due to its cost-effectiveness, widespread accessibility, and capacity to delineate detailed tumor morphology and vascular enhancement patterns (12, 13). Previous studies had demonstrated the utility of CECT features in TETs risk stratification (14–17). However, models relying exclusively on conventional radiological signs exhibited limited predictive performance in discriminating high-risk from low-risk subtypes (18). Numerous studies had demonstrated the efficacy of CECT-based radiomics in accurately differentiating LRT from HRT (19–21). Nevertheless, most prior conventional radiomics investigations had predominantly focused on whole-tumor analysis as a single region of interest (ROI), with insufficient attention paid to tumor subregions exhibiting heterogeneous (22, 23). Recently, habitat imaging had emerged as a novel paradigm that specifically identifies intratumoral heterogeneous regions or cellular subpopulations (24–26). By analyzing tumor subregions, this approach enabled more precise characterization of spatial heterogeneity and enhanced delineation of intrinsic biological features, thereby holding significant promise for refining TETs risk stratification. To date, no studies had investigated the potential of habitat-based radiomics for the risk stratification of TETs. Therefore, we implemented habitat-specific radiomics analysis using CECT imaging for TETs risk stratification, quantitatively assessing its complementary benefits to standard whole-tumor radiomics.

## Materials and methods

### Patients

**Figure 1:**
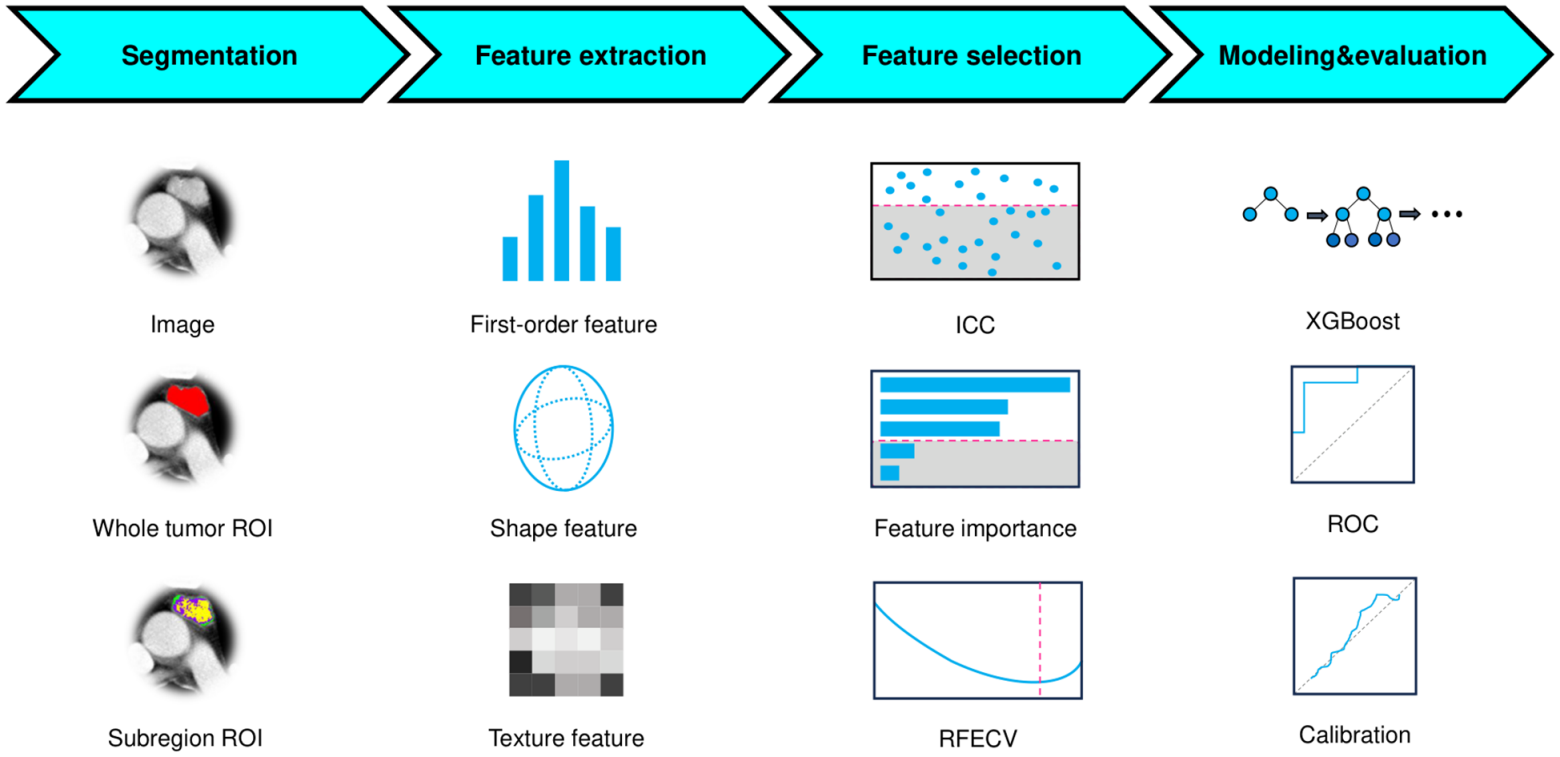
Flowchart of the research.

**Figure 2:**
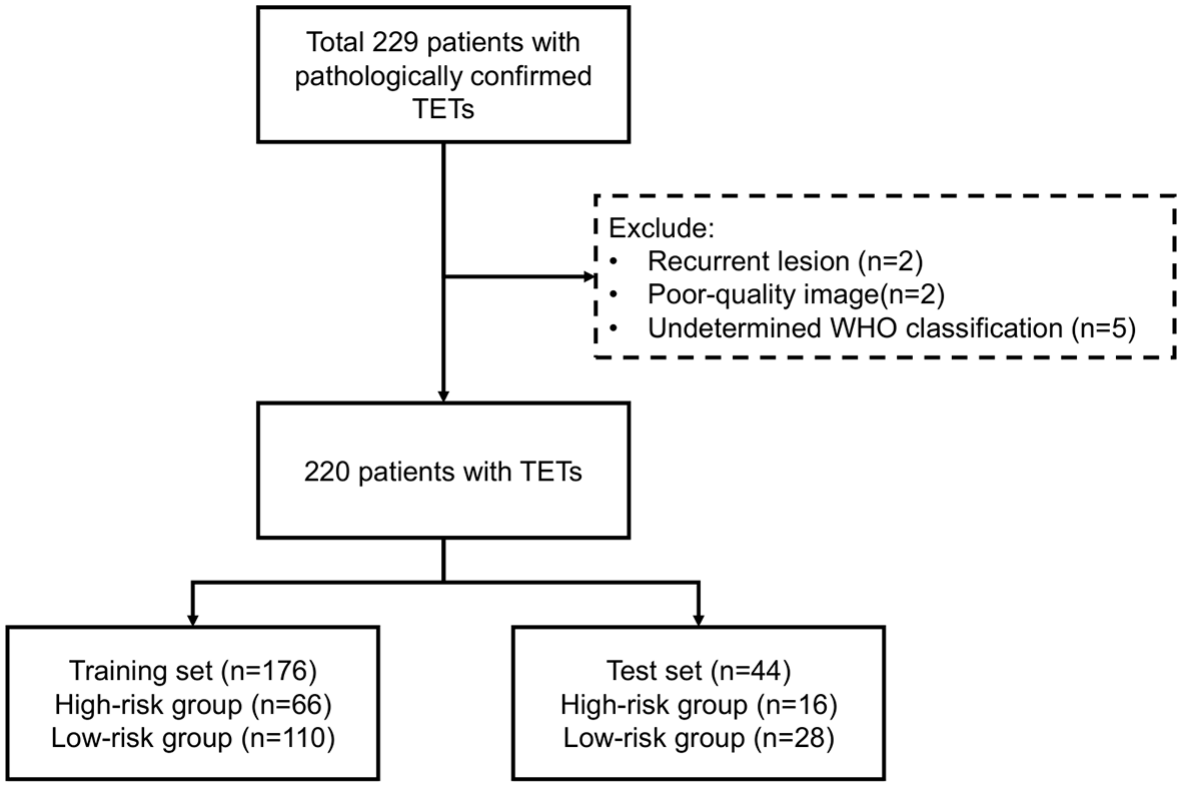
Workflow of patient inclusion.

This study was approved by the institutional review board of the hospital with a waiver for informed (KY2025-157). **Figure 1** outlined the study workflow. This retrospective study consecutively enrolled patients with pathologically confirmed TETs at the Affiliated Second Hospital of Harbin Medical University between December 2017 and December 2024, with **Figure 2** illustrating the workflow of patient inclusion. The inclusion criteria were (1): histologically confirmed WHO classification of TETs (2); preoperative CECT performed within 1 month prior to surgery (3); no prior oncologic therapy (chemotherapy/radiotherapy). The exclusion criteria were (1): recurrent lesion (2); poor-quality image (motion artifacts or incomplete coverage) (3); undetermined WHO classification. The TETs were classified into LRT (A, AB, and B1) and HRT (B2, B3, and TC). Patients with TETs were randomly divided into training (80%) and test sets (20%) using stratified sampling while preserving the original WHO subtype distribution.

### Image acquisition

Three CT scanners (GE Discovery CT750HD; GE LightSpeed VCT; PHILIPS iCT256) were used to perform CECT. The scanning parameters were listed in **Supplementary Material 1**. The contrast material about 80–100 mL was intravenously administered at a rate of 2.5 mL/s. All patients underwent supine-positioned CT examinations spanning from the pulmonary apex to the infradiaphragmatic region. Arterial phase was obtained by threshold trigger and the venous phase scanning was performed after 30s. Image reconstruction was consistently performed using a mediastinum-optimized convolution kernel to ensure accurate interpretation of mediastinal anatomy. The axial images in venous phase were exclusively selected for subsequent quantitative analysis to avoid potential influences from superior vena cava artifacts that were commonly observed in the arterial phase.

### Segmentation

ROIs were manually delineated on axial slices (3D volume) through consensus-based delineation by two radiologists (each with >5 years of expertise in thoracic imaging) using ITK-SNAP software (version 4.2.2, www.itksnap.org). To ensure unbiased evaluation, both radiologists were blinded to all pathological findings and clinical outcomes.

To mitigate outlier effects, intensity values were normalized by truncating the histogram extremities (0.5th to 99.5th percentiles). All images were first spatially normalized to align with a common coordinate space, then isotropically resampled to 1×1×1 mm^3^ voxel dimensions to ensure uniform spatial resolution.

The tumor heterogeneity was quantitatively assessed by partitioning the images into distinct subregions (habitats) using the k-means clustering algorithm. The elbow method, based on the within-cluster sum of squared errors (SSE), was employed to identify the most plausible segmentation. To determine the optimal number of clusters, we evaluated values of k ranging from 2 to 10. The elbow point was determined by locating the value of k where the rate of decrease in within-cluster SSE sharply diminishes.

### Feature extraction

To strictly prevent data leakage, the test set was reserved solely for independent model evaluation and never involved in any prior steps, including feature selection or model development. Radiomic feature extraction was performed on both the whole-tumor ROI and its subregions using PyRadiomics (version 3.1.0, https://pyradiomics.readthedocs.io/en/latest/), encompassing shape-based, first-order statistical, and texture-based features with all available filters applied. Feature definitions and computational methods were listed in the PyRadiomics documentation. For each patient, a total of 6,752 radiomic features were obtained, consisting of 1,688 features from the whole-tumor ROI and an additional 5,064 features (1,688 × 3) derived from three distinct subregions. The extracted features from each subregion were labeled with their corresponding cluster number. Feature normalization was subsequently performed using z-score standardization, where the mean and standard deviation (SD) calculated exclusively from the training set were applied to both training and test sets.

### Feature selection

The feature selection pipeline was implemented in four sequential steps (1): Initial variance thresholding eliminated non-informative features (zero variance across the training set) (2); Reproducibility assessment using a randomly selected subset of 30 patients by two radiologists independently delineating ROIs, retaining only features demonstrating good reproducibility (ICC > 0.75) (3); Feature importance was assessed using an XGBoost classifier, with the top 300 most discriminative features selected according to the importance (4); Final selection through recursive feature elimination with 5-fold cross-validation (RFECV) using a XGBoost classifier to determine the minimal optimal feature subset maximizing AUC. To mitigate the class imbalance between the two groups, the support vector machine synthetic minority over-sampling technique (SVMSMOTE) was employed to augment the sample size of high-risk TETs through synthetic data generation.

### Model development

The conventional radiomics feature and habitat radiomics feature were independently selected through the established pipeline. Subsequently, two machine learning models were developed using the XGBoost algorithm on the SVMSMOTE-balanced training set (1): a conventional radiomics model incorporating conventional imaging features, and (2) a habitat radiomics model capturing tumor subregional heterogeneity patterns. Following the same selection procedure, a combined model was constructed by using the selected features extracted from the whole tumor and subregions.

### Statistics

Sex and group were reported as counts (percentages), while age was summarized as mean ± SD. The difference between training set and test set were assessed using chi-square test (or Fisher’s exact test) for categorical variables and independent t-test (or Mann-Whitney U test) for continuous variables. Model performance was evaluated through receiver operating characteristic (ROC) analysis with calculation of AUC, complemented by metrics including accuracy, sensitivity, and specificity. The incremental prognostic value of habitat features was quantitatively assessed using net reclassification improvement (NRI), integrated discrimination improvement (IDI), and DeLong’s test for AUC comparison. Calibration was verified through Brier score, Brier skill score and calibration curve analysis, while clinical utility was appraised via decision curve analysis (DCA). To enhance interpretability, SHAP (SHapley Additive exPlanations) values were computed to determine feature importance and explain model decisions. All statistical analyses were conducted in Python 3.11. A two-tailed p-values <0.05 considered statistically significant.

## Results

### Baseline characteristics of patients

Of 229 patients, 9 were excluded according to exclusion criteria. The workflow was shown in **Figure 2**. The eligible 220 patients with TETs, randomly divided into training (n=176, 80%) and test (n=44, 20%) sets. According to WHO-based risk stratification, 82 (37.3%) were HRT and 138 (62.7%) LRT. Detailed baseline characteristics are summarized in **Table 1**. There were no statistically significant differences (p>0.05) between the training and test sets in terms of age, gender, group and WHO classification.

**Table 1:** Baseline characteristics.

Characteristics	Overall	Train set	Test set	p-value
N	220	176	44	
gender, n (%)				1
female	110 (50.0)	88 (50.0)	22 (50.0)	
male	110 (50.0)	88 (50.0)	22 (50.0)	
age, mean (SD)	52.9 (11.5)	53.3 (10.5)	51.4 (14.9)	0.425
group, n (%)				1
HR	82 (37.3)	66 (37.5)	16 (36.4)	
LR	138 (62.7)	110 (62.5)	28 (63.6)
WHO classification, n (%)				0.510
A	9 (4.1)	9 (5.1)	0 (0.0)	
AB	118 (53.6)	91 (51.7)	27 (61.4)	
B1	9 (4.1)	8 (4.5)	1 (2.3)	
B2	23 (10.5)	20 (11.4)	3 (6.8)	
B3	39 (17.7)	30 (17.0)	9 (20.5)	
TC	22 (10.0)	18 (10.2)	4 (9.1)	

HR, high-risk; LR, low-risk; TC, thymic carcinoma.

### Feature selection

**Figure 3:**
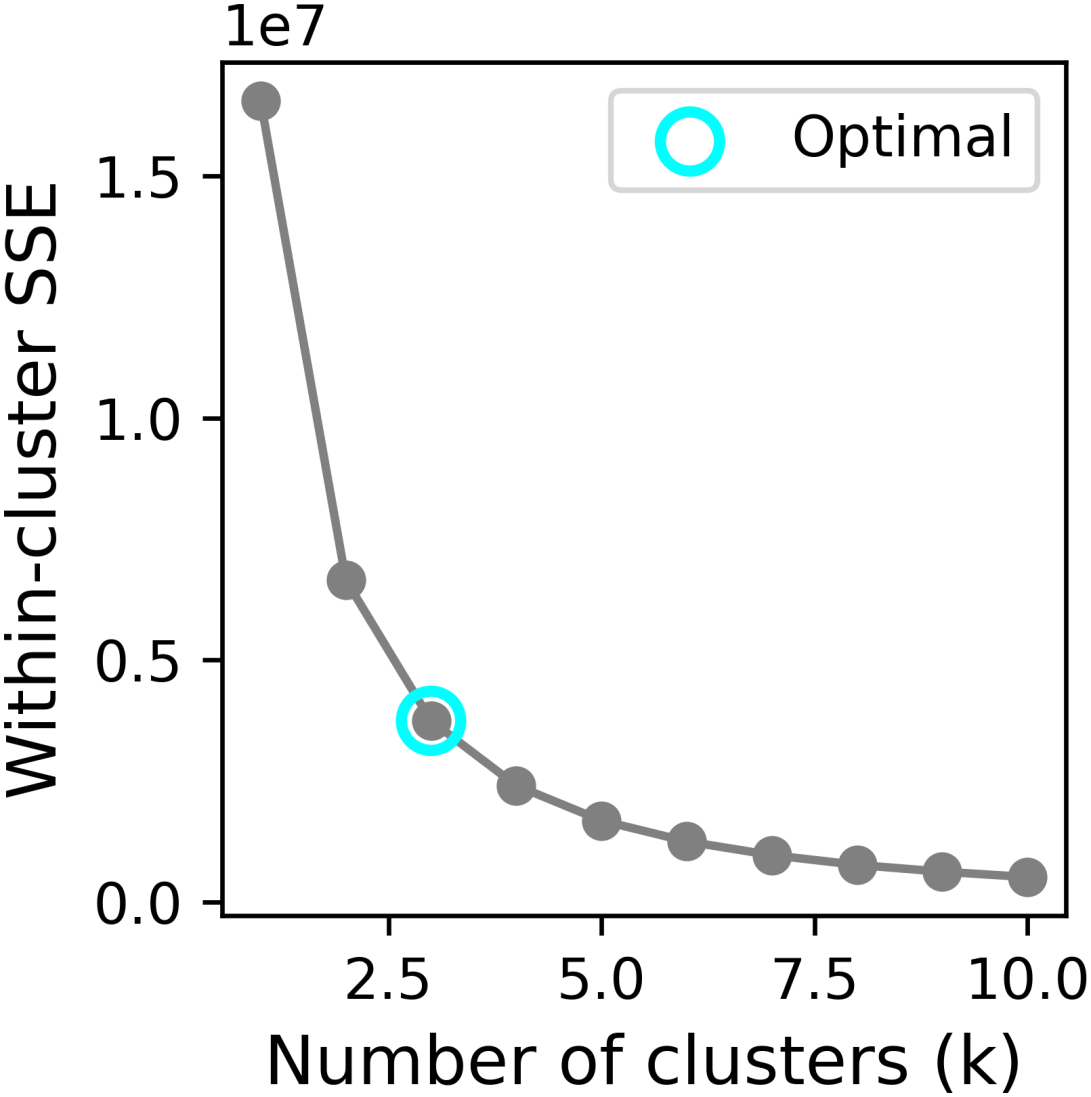
Line plot of finding optimal k.

**Figure 4:**
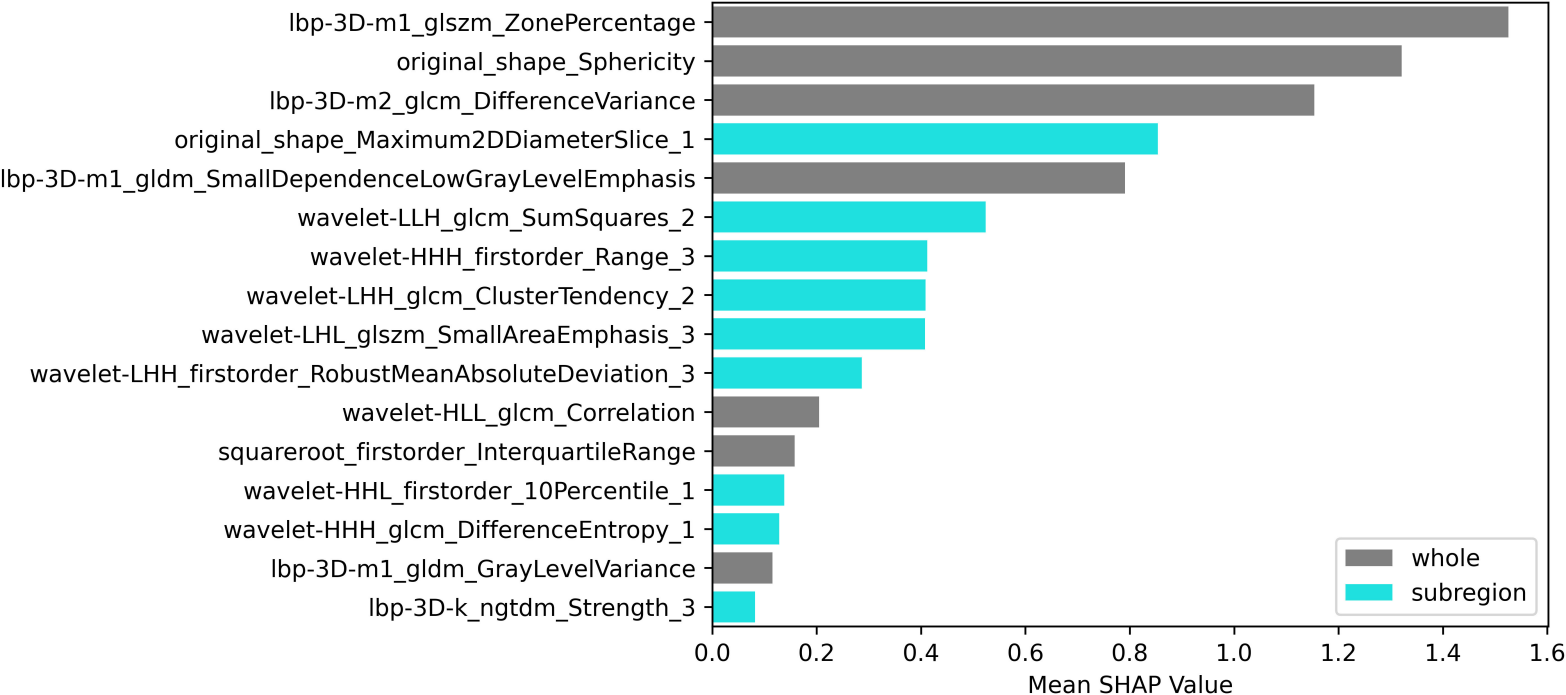
SHAP bar plot (combined model).

**Figure 5:**
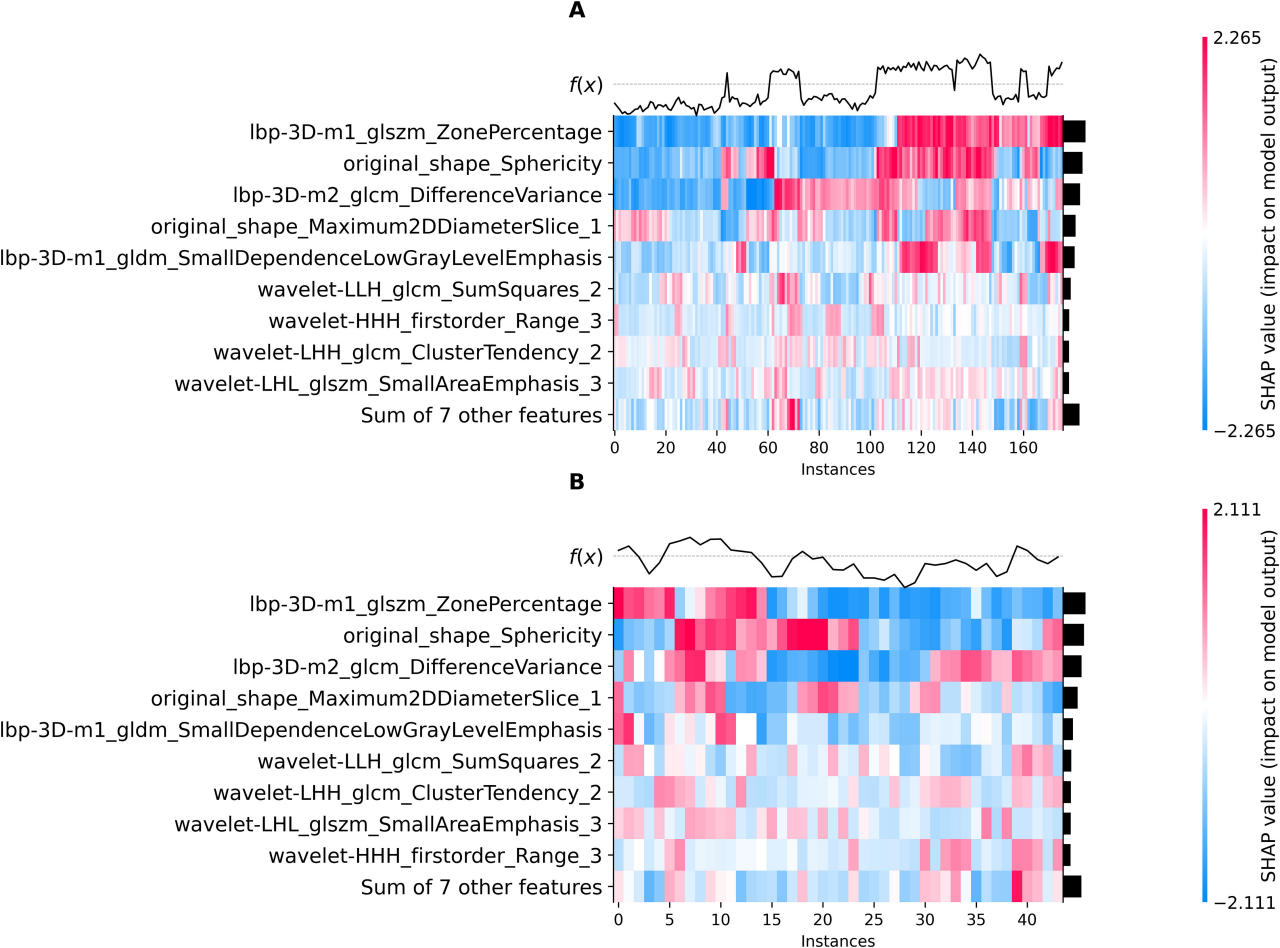
SHAP heatmap (combined model): training set **(A)**, test set **(B)**.

Using the elbow method (**Figure 3**), we identified k = 3 as the optimal number of tumor subregions. From the initial 6,752 extracted features, 6,164 demonstrated non-zero variance, with subsequent reproducibility testing (ICC>0.75) retaining 1,946 robust features (1,429 whole-tumor and 517 subregion-derived). All ICC values were listed in **Supplementary Material 2**. RFECV distilled these to a 16-feature signature selected from the top 300 features for combined model, whose importance is quantified in **Figure 4**. The features developed (n=28) conventional and habitat (n=40) radiomics models were listed in **Supplementary Material 3**. Model interpretability was enhanced through SHAP value heatmap plots **Figure 5**, which delineate individualized feature impacts across the training and test sets.

### Evaluation

**Figure 6:**
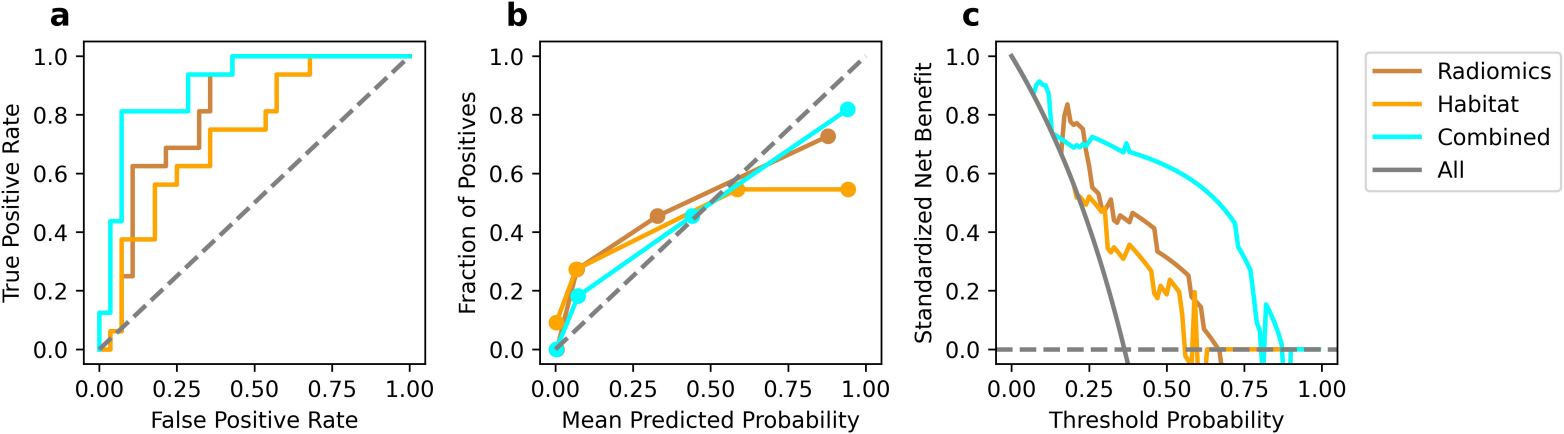
Performance comparison in test set. ROC curves **(a)**, calibration curve **(b)**, and DCA curves **(c)**.

The comparative performance analysis of the three predictive models was listed in **Table 2**. All three models attained an AUC of 1.000, along with accuracy, sensitivity, and specificity all reaching 1.000 on the training set. In the test set, the combined model achieved best performance compared to other models (AUC: 0.819, 0.734 vs 0.900; accuracy: 0.750, 0.705 vs 0.864). Although the models’ performance on the training set could indicate potential overfitting, their strong and consistent results on the independent test set suggest reasonable generalizability. The ROC curve was shown in **Figure 6a**. The comparison via DeLong’s test revealed no statistically significant difference between conventional radiomics and combined models (p=0.161). However, the combined model showed clinically meaningful incremental value, evidenced by NRI (0.286) and IDI (0.209).

**Table 2:** Performance comparison of models.

Model	Cohort	AUC (95% CI)	Accuracy (95% CI)	Sensitivity (95% CI)	Specificity (95% CI)
Conventional	Training	1.000 (1.000-1.000)	1.000 (1.000-1.000)	1.000 (1.000-1.000)	1.000 (1.000-1.000)
	Test	0.819 (0.685-0.934)	0.750 (0.742-0.758)	0.500 (0.255-0.745)	0.893 (0.778-1.000)
Habitat	Training	1.000 (1.000-1.000)	1.000 (1.000-1.000)	1.000 (1.000-1.000)	1.000 (1.000-1.000)
	Test	0.734 (0.575-0.871)	0.705 (0.695-0.714)	0.625 (0.388-0.862)	0.750 (0.590-0.910)
Combined	Training	1.000 (1.000-1.000)	1.000 (1.000-1.000)	1.000 (1.000-1.000)	1.000 (1.000-1.000)
	Test	0.900 (0.797-0.981)	0.864 (0.858-0.869)	0.750 (0.538-0.962)	0.929 (0.833-1.000)

95% CI, 95% confidence interval.

Calibration curves (**Figure 6b**) revealed that the combined model show favorable agreement and had best alignment across the full probability range compared to other models. Quantitative evaluation revealed that the combined model achieved a significantly lower Brier score (0.121) compared to the conventional (0.193) and habitat radiomics models (0.255), corresponding to a Brier skill score of 37.2% relative to the conventional radiomics model as the reference. DCA (**Figure 6c**) demonstrated superior clinical utility of the combined model, which provided a higher net benefit across a wide range of threshold probabilities compared to other models.

### Discussion

This study developed and validated a CECT-based radiomics model for the risk stratification of TETs, demonstrating favorable predictive performance. Radiomic features extracted from tumor habitat heterogeneity could improve the diagnostic accuracy compared to conventional radiomics models. Moreover, SHAP value analysis was employed to quantitatively visualize the feature importance in the combined prediction model.

CECT serves as the preferred non-invasive imaging modality for preoperative evaluation of TETs, providing critical information regarding tumor morphology and vascularity that guides therapeutic decision-making (27). Given the high recurrence rates and poor prognosis associated with HRT, comprehensive preoperative assessment are essential (28). This underscores the clinical importance of CECT-based risk stratification prior to intervention. The arterial phase imaging is susceptible to beam-hardening artifacts from high-concentration contrast media in the superior vena cava, which may compromise image quality and distort texture analysis of adjacent lesions (29). Therefore, our study specifically employed venous phase images for radiomic feature extraction and subsequent model development.

Radiomics represented a robust and well-validated quantitative imaging analysis methodology that facilitated high-throughput extraction of minable data from radiological images (30, 31). By leveraging advanced machine learning algorithms, this approach enabled the identification of clinically relevant imaging biomarkers and the development of predictive models for precise estimation of clinical outcomes and therapeutic endpoints. Prior investigations had demonstrated the superior performance of radiomics in TETs risk stratification, with enhanced discriminative capability compared to conventional clinical model (18, 29). In the current study, our conventional radiomics model demonstrated favorable performance, achieving an AUC of 0.819 and an accuracy of 0.750 the independent test set, with balanced sensitivity and specificity indicating minimal class prediction bias. Ren et al. reported a radiomics model for TET histologic subtyping with an AUC of 0.860 and accuracy of 0.750, while Zhou et al. achieved an AUC of 0.716 and accuracy of 0.736 in predicting histological risk categorization (18, 32). The slight variations of observed performance may be attributable to methodological differences in feature selection and algorithmic implementation. Our approach utilized RFECV, an iterative selection process that optimizes feature combinations while preserving synergistic predictive value and model generalizability. Furthermore, the XGBoost algorithm’s tree-based architecture could accept feature collinearity while autonomously capturing critical nonlinear relationships through hierarchical feature interactions.

The complex cellular and molecular milieu surrounding tumor cells - the tumor microenvironment - critically influenced oncogenesis, disease progression, and therapeutic outcomes (33). Most prior studies on the risk stratification of TETs had primarily focused on whole-tumor analysis, while largely neglecting the potential significance of intratumoral subregion (23). Our study implemented habitat imaging to explore the incremental value of characterizing spatial tumor heterogeneity in TETs. We systematically partitioned TETs into three distinct subregions and extracted habitat-specific features. Habitat analysis identified distinct subregions: Habitat_1/2 represented solid tumor components (Habitat_2 showing lower enhancement), while Habitat_3 in corresponded to necrotic foci or tumor margin. Among 16 features developed combined model, 7 were conventional radiomic features, and other 9 were habitat-derived features. Quantitative analysis of tumor subregional architecture provided enhanced characterization of intratumoral spatial heterogeneity and enabled more biologically faithful representation of tumor phenotypic features. The combined model achieved superior discriminative performance (AUC=0.900, accuracy=0.864) in the test set compared to either feature set alone. Although DeLong test revealed no statistically significant AUC difference between conventional and combined models (p>0.05), the numerical improvement and enhanced classification metrics substantiated the incremental value of habitat radiomics for TETs risk stratification, suggesting that spatial heterogeneity provided complementary biological information.

The radiomic feature selection for the combined predictive model identified three primary discriminators with significant prognostic value: Ibp-3D-m1_glszm_ZonePercentage, Ibp-3D-m2_glcm_DifferenceVariance, and original_shape_Sphericity. These biomarkers collectively characterize distinct tumor biological behaviors, where the high-risk cohort exhibited (1): decreased Sphericity values, indicative of irregular morphological patterns and invasive growth tendencies (2); elevated DifferenceVariance, reflecting marked intratumoral textural heterogeneity; and (3) reduced ZonePercentage, suggesting disordered spatial distribution of tumor zones. Furthermore, three additional habitat-specific features provided complementary pathophysiological insights: original_shape_Maximum2DDiameterSlice_1 demonstrated strong correlation with proliferative activity in solid tumor components. wavelet-LIH_glcm_SumSquares_2 precisely quantified textural heterogeneity within viable tumor regions, where heightened values corresponded to areas of cellular atypia and structural disorganization. wavelet-HHH_firstorder_Range_3 effectively captured the complexity of necrotic and marginal zones through density dispersion metrics, with elevated values indicating pathological processes including hemorrhagic transformation, dystrophic calcification, or residual tumor infiltration.

Unlike manually crafted radiomics features, deep learning autonomously extracted task-specific features directly from images. With rapid advancements in deep learning methodologies, these data-driven features had emerged as a powerful complement to conventional radiomics features in medical imaging (34). This advantage is exemplified in the work of Zhou et al, who systematically compared conventional radiomics, deep learning, and combined models for the risk stratification of TETs (32). Their findings demonstrated that the combined model significantly outperformed conventional radiomics (AUC improvement from 0.716 to 0.786; accuracy increase from 0.736 to 0.774), likely due to the complementary nature of hand-crafted radiomic features and deep learning-derived representations (32). Building upon the established risk stratification framework of Zhou et al., our study demonstrates that integrating habitat-specific radiomic features yields significant improvements in predictive performance compared to conventional approaches. The combined model exhibited enhanced diagnostic capability, with accuracy increasing from 0.750 to 0.864 and AUC improving from 0.819 to 0.900. This performance surpasses previously reported results (accuracy: 0.864 vs 0.774; AUC: 0.900 vs 0.786), representing a meaningful advancement in predictive modeling. Notably, our findings reveal that habitat feature incorporation provides greater performance enhancement in conventional radiomics models, suggesting these biologically relevant features may capture distinct and complementary tumor characteristics.

Despite its methodological rigor, this study had several limitations that should be acknowledged. First, the retrospective nature of the analysis might still introduce selection bias, particularly given the specific inclusion/exclusion criteria applied. Second, although SVMSMOTE was implemented to address class imbalance between high- and low-risk TETs (37.3% vs. 62.7%), the fundamental disproportion in WHO subtype prevalence might still influence model performance in real-world clinical settings. Third, the utilization of multiple CT scanners, while reflecting clinical reality, introduced inherent technical variability despite rigorous standardization protocols. Fourth, while interobserver variability in manual segmentation was mitigated through ICC-based filtering, residual subjectivity inherent in manual annotations may persist. Finally, although not employed in this study, the harmonization technique (e.g., ComBat) could further improve feature robustness in future multi-center investigations by explicitly addressing scanner-induced heterogeneity.

In conclusion, the present study establishes that CECT-based habitat radiomics offered significant improvements in risk stratification for TETs compared to conventional radiomic approaches, particularly within clinically relevant decision-making ranges. The enhanced predictive performance of our combined model substantiated the importance of characterizing intratumoral heterogeneity through advanced habitat analysis, demonstrating substantial potential for clinical translation.

## Data Availability

The original contributions presented in the study are included in the article/**Supplementary Material**, further inquiries can be directed to the corresponding authors.
