# Supplementary material for: Incremental diagnostic value of tumor habitat radiomics for risk stratification in thymic epithelial tumors
Source: Front Oncol. 2025 Sep 3;15:1630485. doi: 10.3389/fonc.2025.1630485 (PMC12440988; doi:10.3389/fonc.2025.1630485)
Supplement: Supplementary file 1 [file DataSheet1.pdf]

Parameters	GE Discovery CT750HD	GE LightSpeed VCT	PHILIPS iCT256
Tube voltage, kVp	120	120	120
Tube current, mAs	Auto	Auto	Auto
Slice thickness, mm	5	5	5
Slice increment, mm	5	5	5
Matrix	512×512	512×512	512×512
Pitch	1.375	0.985	0.8
