# Supplementary material for: Incremental diagnostic value of tumor habitat radiomics for risk stratification in thymic epithelial tumors
Source: Front Oncol. 2025 Sep 3;15:1630485. doi: 10.3389/fonc.2025.1630485 (PMC12440988; doi:10.3389/fonc.2025.1630485)
Supplement: Supplementary file 2 [file DataSheet2.pdf]

feature	ICC
original_shape_Elongation	0.987
original_shape_Flatness	0.994
original_shape_LeastAxisLength	0.999
original_shape_MajorAxisLength	0.999
original_shape_Maximum2DDiameterColumn	0.999
original_shape_Maximum2DDiameterRow	0.999
original_shape_Maximum2DDiameterSlice	0.997
original_shape_Maximum3DDiameter	0.999
original_shape_MeshVolume	0.999
original_shape_MinorAxisLength	0.999
original_shape_Sphericity	0.993
original_shape_SurfaceArea	0.999
original_shape_SurfaceVolumeRatio	0.998
original_shape_VoxelVolume	0.999
original_firstorder_10Percentile	0.999
original_firstorder_90Percentile	1.000
original_firstorder_Energy	0.999
original_firstorder_Entropy	0.796
original_firstorder_InterquartileRange	0.984
original_firstorder_Kurtosis	0.577
original_firstorder_Maximum	0.993
original_firstorder_MeanAbsoluteDeviation	0.945
original_firstorder_Mean	1.000
original_firstorder_Median	1.000
original_firstorder_Minimum	0.880
original_firstorder_Range	0.960
original_firstorder_RobustMeanAbsoluteDeviation	0.980
original_firstorder_RootMeanSquared	1.000
original_firstorder_Skewness	0.715
original_firstorder_TotalEnergy	0.999
original_firstorder_Uniformity	0.758
original_firstorder_Variance	0.884
original_glcm_Autocorrelation	0.850
original_glcm_ClusterProminence	0.665
original_glcm_ClusterShade	0.691
original_glcm_ClusterTendency	0.722
original_glcm_Contrast	0.805
original_glcm_Correlation	0.900
original_glcm_DifferenceAverage	0.805
original_glcm_DifferenceEntropy	0.837
original_glcm_DifferenceVariance	0.805
original_glcm_Id	0.805
original_glcm_Idm	0.805
original_glcm_Idmn	0.805
original_glcm_Idn	0.805
original_glcm_Imc1	0.934
original_glcm_Imc2	0.793
original_glcm_InverseVariance	0.805
original_glcm_JointAverage	0.850

original_glcm_JointEnergy	0.773
original_glcm_JointEntropy	0.802
original_glcm_MCC	0.812
original_glcm_MaximumProbability	0.773
original_glcm_SumAverage	0.850
original_glcm_SumEntropy	0.802
original_glcm_SumSquares	0.751
original_gldm_DependenceEntropy	0.998
original_gldm_DependenceNonUniformity	0.999
original_gldm_DependenceNonUniformityNormalized	0.998
original_gldm_DependenceVariance	0.998
original_gldm_GrayLevelNonUniformity	0.999
original_gldm_GrayLevelVariance	0.758
original_gldm_HighGrayLevelEmphasis	0.850
original_gldm_LargeDependenceEmphasis	0.998
original_gldm_LargeDependenceHighGrayLevelEmphasis	0.871
original_gldm_LargeDependenceLowGrayLevelEmphasis	0.846
original_gldm_LowGrayLevelEmphasis	0.850
original_gldm_SmallDependenceEmphasis	0.995
original_gldm_SmallDependenceHighGrayLevelEmphasis	0.811
original_gldm_SmallDependenceLowGrayLevelEmphasis	0.953
original_glrlm_GrayLevelNonUniformity	0.999
original_glrlm_GrayLevelNonUniformityNormalized	0.783
original_glrlm_GrayLevelVariance	0.783
original_glrlm_HighGrayLevelRunEmphasis	0.848
original_glrlm_LongRunEmphasis	0.999
original_glrlm_LongRunHighGrayLevelEmphasis	0.982
original_glrlm_LongRunLowGrayLevelEmphasis	0.983
original_glrlm_LowGrayLevelRunEmphasis	0.848
original_glrlm_RunEntropy	0.998
original_glrlm_RunLengthNonUniformity	0.998
original_glrlm_RunLengthNonUniformityNormalized	0.998
original_glrlm_RunPercentage	0.998
original_glrlm_RunVariance	0.999
original_glrlm_ShortRunEmphasis	0.990
original_glrlm_ShortRunHighGrayLevelEmphasis	0.835
original_glrlm_ShortRunLowGrayLevelEmphasis	0.968
original_glszm_GrayLevelNonUniformity	0.699
original_glszm_GrayLevelNonUniformityNormalized	0.731
original_glszm_GrayLevelVariance	0.731
original_glszm_HighGrayLevelZoneEmphasis	0.671
original_glszm_LargeAreaEmphasis	0.982
original_glszm_LargeAreaHighGrayLevelEmphasis	0.971
original_glszm_LargeAreaLowGrayLevelEmphasis	0.995
original_glszm_LowGrayLevelZoneEmphasis	0.671
original_glszm_SizeZoneNonUniformity	0.634
original_glszm_SizeZoneNonUniformityNormalized	0.774
original_glszm_SmallAreaEmphasis	0.766
original_glszm_SmallAreaHighGrayLevelEmphasis	0.766
original_glszm_SmallAreaLowGrayLevelEmphasis	0.766

original_glszm_ZoneEntropy	0.881
original_glszm_ZonePercentage	0.995
original_glszm_ZoneVariance	0.994
original_ngtdm_Busyness	0.789
original_ngtdm_Coarseness	0.850
original_ngtdm_Complexity	0.808
original_ngtdm_Contrast	0.512
original_ngtdm_Strength	0.583
exponential_firstorder_10Percentile	1.000
exponential_firstorder_90Percentile	1.000
exponential_firstorder_Energy	0.999
exponential_firstorder_Entropy	0.000
exponential_firstorder_InterquartileRange	0.992
exponential_firstorder_Kurtosis	0.598
exponential_firstorder_Maximum	0.996
exponential_firstorder_MeanAbsoluteDeviation	0.968
exponential_firstorder_Mean	1.000
exponential_firstorder_Median	1.000
exponential_firstorder_Minimum	0.870
exponential_firstorder_Range	0.978
exponential_firstorder_RobustMeanAbsoluteDeviation	0.989
exponential_firstorder_RootMeanSquared	1.000
exponential_firstorder_Skewness	0.764
exponential_firstorder_TotalEnergy	0.999
exponential_firstorder_Variance	0.920
exponential_gldm_DependenceEntropy	0.998
exponential_gldm_DependenceNonUniformity	0.999
exponential_gldm_DependenceNonUniformityNormalized	0.998
exponential_gldm_DependenceVariance	0.998
exponential_gldm_GrayLevelNonUniformity	0.999
exponential_gldm_LargeDependenceEmphasis	0.998
exponential_gldm_LargeDependenceHighGrayLevelEmphasis	0.998
exponential_gldm_LargeDependenceLowGrayLevelEmphasis	0.998
exponential_gldm_SmallDependenceEmphasis	0.995
exponential_gldm_SmallDependenceHighGrayLevelEmphasis	0.995
exponential_gldm_SmallDependenceLowGrayLevelEmphasis	0.995
exponential_glrlm_GrayLevelNonUniformity	0.999
exponential_glrlm_LongRunEmphasis	0.999
exponential_glrlm_LongRunHighGrayLevelEmphasis	0.999
exponential_glrlm_LongRunLowGrayLevelEmphasis	0.999
exponential_glrlm_RunEntropy	0.998
exponential_glrlm_RunLengthNonUniformity	0.998
exponential_glrlm_RunLengthNonUniformityNormalized	0.998
exponential_glrlm_RunPercentage	0.998
exponential_glrlm_RunVariance	0.999
exponential_glrlm_ShortRunEmphasis	0.991
exponential_glrlm_ShortRunHighGrayLevelEmphasis	0.991
exponential_glrlm_ShortRunLowGrayLevelEmphasis	0.991
exponential_glszm_GrayLevelNonUniformity	0.000
exponential_glszm_LargeAreaEmphasis	0.996

exponential_glszm_LargeAreaHighGrayLevelEmphasis	0.996
exponential_glszm_LargeAreaLowGrayLevelEmphasis	0.996
exponential_glszm_SizeZoneNonUniformityNormalized	0.000
exponential_glszm_SmallAreaEmphasis	0.000
exponential_glszm_SmallAreaHighGrayLevelEmphasis	0.000
exponential_glszm_SmallAreaLowGrayLevelEmphasis	0.000
exponential_glszm_ZoneEntropy	0.000
exponential_glszm_ZonePercentage	0.996
exponential_glszm_ZoneVariance	0.000
gradient_firstorder_10Percentile	0.996
gradient_firstorder_90Percentile	0.865
gradient_firstorder_Energy	0.920
gradient_firstorder_Entropy	0.000
gradient_firstorder_InterquartileRange	0.956
gradient_firstorder_Kurtosis	0.876
gradient_firstorder_Maximum	0.965
gradient_firstorder_MeanAbsoluteDeviation	0.849
gradient_firstorder_Mean	0.919
gradient_firstorder_Median	0.991
gradient_firstorder_Minimum	1.000
gradient_firstorder_Range	0.965
gradient_firstorder_RobustMeanAbsoluteDeviation	0.923
gradient_firstorder_RootMeanSquared	0.865
gradient_firstorder_Skewness	0.964
gradient_firstorder_TotalEnergy	0.920
gradient_firstorder_Variance	0.796
gradient_gldm_DependenceEntropy	0.998
gradient_gldm_DependenceNonUniformity	0.999
gradient_gldm_DependenceNonUniformityNormalized	0.998
gradient_gldm_DependenceVariance	0.998
gradient_gldm_GrayLevelNonUniformity	0.999
gradient_gldm_LargeDependenceEmphasis	0.998
gradient_gldm_LargeDependenceHighGrayLevelEmphasis	0.998
gradient_gldm_LargeDependenceLowGrayLevelEmphasis	0.998
gradient_gldm_SmallDependenceEmphasis	0.995
gradient_gldm_SmallDependenceHighGrayLevelEmphasis	0.995
gradient_gldm_SmallDependenceLowGrayLevelEmphasis	0.995
gradient_glrlm_GrayLevelNonUniformity	0.999
gradient_glrlm_LongRunEmphasis	0.999
gradient_glrlm_LongRunHighGrayLevelEmphasis	0.999
gradient_glrlm_LongRunLowGrayLevelEmphasis	0.999
gradient_glrlm_RunEntropy	0.998
gradient_glrlm_RunLengthNonUniformity	0.998
gradient_glrlm_RunLengthNonUniformityNormalized	0.998
gradient_glrlm_RunPercentage	0.998
gradient_glrlm_RunVariance	0.999
gradient_glrlm_ShortRunEmphasis	0.991
gradient_glrlm_ShortRunHighGrayLevelEmphasis	0.991
gradient_glrlm_ShortRunLowGrayLevelEmphasis	0.991
gradient_glszm_GrayLevelNonUniformity	0.000

gradient_glszm_LargeAreaEmphasis	0.996
gradient_glszm_LargeAreaHighGrayLevelEmphasis	0.996
gradient_glszm_LargeAreaLowGrayLevelEmphasis	0.996
gradient_glszm_SizeZoneNonUniformityNormalized	0.000
gradient_glszm_SmallAreaEmphasis	0.000
gradient_glszm_SmallAreaHighGrayLevelEmphasis	0.000
gradient_glszm_SmallAreaLowGrayLevelEmphasis	0.000
gradient_glszm_ZoneEntropy	0.000
gradient_glszm_ZonePercentage	0.996
gradient_glszm_ZoneVariance	0.000
lbp-2D_firstorder_10Percentile	0.914
lbp-2D_firstorder_90Percentile	0.000
lbp-2D_firstorder_Energy	0.999
lbp-2D_firstorder_Entropy	0.955
lbp-2D_firstorder_InterquartileRange	0.927
lbp-2D_firstorder_Kurtosis	0.968
lbp-2D_firstorder_MeanAbsoluteDeviation	0.969
lbp-2D_firstorder_Mean	0.990
lbp-2D_firstorder_Median	0.932
lbp-2D_firstorder_RobustMeanAbsoluteDeviation	0.965
lbp-2D_firstorder_RootMeanSquared	0.986
lbp-2D_firstorder_Skewness	0.987
lbp-2D_firstorder_TotalEnergy	0.999
lbp-2D_firstorder_Uniformity	0.955
lbp-2D_firstorder_Variance	0.969
lbp-2D_glcm_Autocorrelation	0.979
lbp-2D_glcm_ClusterProminence	0.917
lbp-2D_glcm_ClusterShade	0.976
lbp-2D_glcm_ClusterTendency	0.915
lbp-2D_glcm_Contrast	0.910
lbp-2D_glcm_Correlation	0.912
lbp-2D_glcm_DifferenceAverage	0.910
lbp-2D_glcm_DifferenceEntropy	0.999
lbp-2D_glcm_DifferenceVariance	0.998
lbp-2D_glcm_Id	0.910
lbp-2D_glcm_Idm	0.910
lbp-2D_glcm_Idmn	0.910
lbp-2D_glcm_Idn	0.910
lbp-2D_glcm_Imc1	0.999
lbp-2D_glcm_Imc2	0.962
lbp-2D_glcm_InverseVariance	0.910
lbp-2D_glcm_JointAverage	0.976
lbp-2D_glcm_JointEnergy	0.955
lbp-2D_glcm_JointEntropy	0.962
lbp-2D_glcm_MCC	0.970
lbp-2D_glcm_MaximumProbability	0.915
lbp-2D_glcm_SumAverage	0.976
lbp-2D_glcm_SumEntropy	0.934
lbp-2D_glcm_SumSquares	0.926
lbp-2D_gldm_DependenceEntropy	0.954

lbp-2D_gldm_DependenceNonUniformity	0.999
lbp-2D_gldm_DependenceNonUniformityNormalized	0.948
lbp-2D_gldm_DependenceVariance	0.921
lbp-2D_gldm_GrayLevelNonUniformity	0.999
lbp-2D_gldm_GrayLevelVariance	0.955
lbp-2D_gldm_HighGrayLevelEmphasis	0.981
lbp-2D_gldm_LargeDependenceEmphasis	0.980
lbp-2D_gldm_LargeDependenceHighGrayLevelEmphasis	0.999
lbp-2D_gldm_LargeDependenceLowGrayLevelEmphasis	0.919
lbp-2D_gldm_LowGrayLevelEmphasis	0.981
lbp-2D_gldm_SmallDependenceEmphasis	0.980
lbp-2D_gldm_SmallDependenceHighGrayLevelEmphasis	0.974
lbp-2D_gldm_SmallDependenceLowGrayLevelEmphasis	0.984
lbp-2D_glrlm_GrayLevelNonUniformity	0.999
lbp-2D_glrlm_GrayLevelNonUniformityNormalized	0.968
lbp-2D_glrlm_GrayLevelVariance	0.968
lbp-2D_glrlm_HighGrayLevelRunEmphasis	0.979
lbp-2D_glrlm_LongRunEmphasis	0.998
lbp-2D_glrlm_LongRunHighGrayLevelEmphasis	1.000
lbp-2D_glrlm_LongRunLowGrayLevelEmphasis	0.991
lbp-2D_glrlm_LowGrayLevelRunEmphasis	0.979
lbp-2D_glrlm_RunEntropy	0.987
lbp-2D_glrlm_RunLengthNonUniformity	0.999
lbp-2D_glrlm_RunLengthNonUniformityNormalized	0.983
lbp-2D_glrlm_RunPercentage	0.985
lbp-2D_glrlm_RunVariance	0.998
lbp-2D_glrlm_ShortRunEmphasis	0.985
lbp-2D_glrlm_ShortRunHighGrayLevelEmphasis	0.988
lbp-2D_glrlm_ShortRunLowGrayLevelEmphasis	0.962
lbp-2D_glszm_GrayLevelNonUniformity	0.933
lbp-2D_glszm_GrayLevelNonUniformityNormalized	0.797
lbp-2D_glszm_GrayLevelVariance	0.797
lbp-2D_glszm_HighGrayLevelZoneEmphasis	0.831
lbp-2D_glszm_LargeAreaEmphasis	0.981
lbp-2D_glszm_LargeAreaHighGrayLevelEmphasis	0.981
lbp-2D_glszm_LargeAreaLowGrayLevelEmphasis	0.981
lbp-2D_glszm_LowGrayLevelZoneEmphasis	0.831
lbp-2D_glszm_SizeZoneNonUniformity	0.953
lbp-2D_glszm_SizeZoneNonUniformityNormalized	0.780
lbp-2D_glszm_SmallAreaEmphasis	0.794
lbp-2D_glszm_SmallAreaHighGrayLevelEmphasis	0.775
lbp-2D_glszm_SmallAreaLowGrayLevelEmphasis	0.844
lbp-2D_glszm_ZoneEntropy	0.821
lbp-2D_glszm_ZonePercentage	0.907
lbp-2D_glszm_ZoneVariance	0.984
lbp-2D_ngtdm_Busyness	0.997
lbp-2D_ngtdm_Coarseness	0.997
lbp-2D_ngtdm_Complexity	0.928
lbp-2D_ngtdm_Contrast	0.940
lbp-2D_ngtdm_Strength	0.997

lbp-3D-m1_firstorder_10Percentile	0.904
lbp-3D-m1_firstorder_90Percentile	0.987
lbp-3D-m1_firstorder_Energy	0.999
lbp-3D-m1_firstorder_Entropy	0.995
lbp-3D-m1_firstorder_InterquartileRange	0.954
lbp-3D-m1_firstorder_Kurtosis	0.986
lbp-3D-m1_firstorder_Maximum	1.000
lbp-3D-m1_firstorder_MeanAbsoluteDeviation	0.987
lbp-3D-m1_firstorder_Mean	0.994
lbp-3D-m1_firstorder_Median	0.955
lbp-3D-m1_firstorder_Range	1.000
lbp-3D-m1_firstorder_RobustMeanAbsoluteDeviation	0.976
lbp-3D-m1_firstorder_RootMeanSquared	0.998
lbp-3D-m1_firstorder_Skewness	0.999
lbp-3D-m1_firstorder_TotalEnergy	0.999
lbp-3D-m1_firstorder_Uniformity	0.992
lbp-3D-m1_firstorder_Variance	0.990
lbp-3D-m1_glcm_Autocorrelation	0.996
lbp-3D-m1_glcm_ClusterProminence	0.993
lbp-3D-m1_glcm_ClusterShade	0.986
lbp-3D-m1_glcm_ClusterTendency	0.992
lbp-3D-m1_glcm_Contrast	0.990
lbp-3D-m1_glcm_Correlation	0.991
lbp-3D-m1_glcm_DifferenceAverage	0.987
lbp-3D-m1_glcm_DifferenceEntropy	0.995
lbp-3D-m1_glcm_DifferenceVariance	0.996
lbp-3D-m1_glcm_Id	0.983
lbp-3D-m1_glcm_Idm	0.984
lbp-3D-m1_glcm_Idmn	0.989
lbp-3D-m1_glcm_Idn	0.986
lbp-3D-m1_glcm_Imc1	0.999
lbp-3D-m1_glcm_Imc2	0.998
lbp-3D-m1_glcm_InverseVariance	0.999
lbp-3D-m1_glcm_JointAverage	0.997
lbp-3D-m1_glcm_JointEnergy	0.991
lbp-3D-m1_glcm_JointEntropy	0.994
lbp-3D-m1_glcm_MCC	0.998
lbp-3D-m1_glcm_MaximumProbability	0.967
lbp-3D-m1_glcm_SumAverage	0.997
lbp-3D-m1_glcm_SumEntropy	0.995
lbp-3D-m1_glcm_SumSquares	0.991
lbp-3D-m1_gldm_DependenceEntropy	0.989
lbp-3D-m1_gldm_DependenceNonUniformity	1.000
lbp-3D-m1_gldm_DependenceNonUniformityNormalized	0.946
lbp-3D-m1_gldm_DependenceVariance	0.933
lbp-3D-m1_gldm_GrayLevelNonUniformity	0.998
lbp-3D-m1_gldm_GrayLevelVariance	0.991
lbp-3D-m1_gldm_HighGrayLevelEmphasis	0.997
lbp-3D-m1_gldm_LargeDependenceEmphasis	0.933
lbp-3D-m1_gldm_LargeDependenceHighGrayLevelEmphasis	0.910

lbp-3D-m1_gldm_LargeDependenceLowGrayLevelEmphasis	0.954
lbp-3D-m1_gldm_LowGrayLevelEmphasis	0.934
lbp-3D-m1_gldm_SmallDependenceEmphasis	0.970
lbp-3D-m1_gldm_SmallDependenceHighGrayLevelEmphasis	0.992
lbp-3D-m1_gldm_SmallDependenceLowGrayLevelEmphasis	0.877
lbp-3D-m1_glrlm_GrayLevelNonUniformity	0.999
lbp-3D-m1_glrlm_GrayLevelNonUniformityNormalized	0.996
lbp-3D-m1_glrlm_GrayLevelVariance	0.995
lbp-3D-m1_glrlm_HighGrayLevelRunEmphasis	0.998
lbp-3D-m1_glrlm_LongRunEmphasis	0.986
lbp-3D-m1_glrlm_LongRunHighGrayLevelEmphasis	0.985
lbp-3D-m1_glrlm_LongRunLowGrayLevelEmphasis	0.999
lbp-3D-m1_glrlm_LowGrayLevelRunEmphasis	0.924
lbp-3D-m1_glrlm_RunEntropy	0.995
lbp-3D-m1_glrlm_RunLengthNonUniformity	1.000
lbp-3D-m1_glrlm_RunLengthNonUniformityNormalized	0.939
lbp-3D-m1_glrlm_RunPercentage	0.935
lbp-3D-m1_glrlm_RunVariance	0.982
lbp-3D-m1_glrlm_ShortRunEmphasis	0.952
lbp-3D-m1_glrlm_ShortRunHighGrayLevelEmphasis	0.996
lbp-3D-m1_glrlm_ShortRunLowGrayLevelEmphasis	0.915
lbp-3D-m1_glszm_GrayLevelNonUniformity	1.000
lbp-3D-m1_glszm_GrayLevelNonUniformityNormalized	0.993
lbp-3D-m1_glszm_GrayLevelVariance	0.984
lbp-3D-m1_glszm_HighGrayLevelZoneEmphasis	0.996
lbp-3D-m1_glszm_LargeAreaEmphasis	0.997
lbp-3D-m1_glszm_LargeAreaHighGrayLevelEmphasis	0.997
lbp-3D-m1_glszm_LargeAreaLowGrayLevelEmphasis	0.999
lbp-3D-m1_glszm_LowGrayLevelZoneEmphasis	0.975
lbp-3D-m1_glszm_SizeZoneNonUniformity	0.999
lbp-3D-m1_glszm_SizeZoneNonUniformityNormalized	0.991
lbp-3D-m1_glszm_SmallAreaEmphasis	0.992
lbp-3D-m1_glszm_SmallAreaHighGrayLevelEmphasis	0.998
lbp-3D-m1_glszm_SmallAreaLowGrayLevelEmphasis	0.859
lbp-3D-m1_glszm_ZoneEntropy	0.996
lbp-3D-m1_glszm_ZonePercentage	0.983
lbp-3D-m1_glszm_ZoneVariance	0.997
lbp-3D-m1_ngtdm_Busyness	1.000
lbp-3D-m1_ngtdm_Coarseness	0.997
lbp-3D-m1_ngtdm_Complexity	0.960
lbp-3D-m1_ngtdm_Contrast	0.988
lbp-3D-m1_ngtdm_Strength	0.997
lbp-3D-m2_firstorder_10Percentile	0.980
lbp-3D-m2_firstorder_90Percentile	0.993
lbp-3D-m2_firstorder_Energy	0.999
lbp-3D-m2_firstorder_Entropy	0.981
lbp-3D-m2_firstorder_InterquartileRange	0.972
lbp-3D-m2_firstorder_Kurtosis	0.981
lbp-3D-m2_firstorder_Maximum	0.998
lbp-3D-m2_firstorder_MeanAbsoluteDeviation	0.974

lbp-3D-m2_firstorder_Mean	0.995
lbp-3D-m2_firstorder_Median	0.997
lbp-3D-m2_firstorder_Range	0.998
lbp-3D-m2_firstorder_RobustMeanAbsoluteDeviation	0.972
lbp-3D-m2_firstorder_RootMeanSquared	0.996
lbp-3D-m2_firstorder_Skewness	0.995
lbp-3D-m2_firstorder_TotalEnergy	0.999
lbp-3D-m2_firstorder_Uniformity	0.981
lbp-3D-m2_firstorder_Variance	0.978
lbp-3D-m2_glcm_Autocorrelation	0.996
lbp-3D-m2_glcm_ClusterProminence	0.983
lbp-3D-m2_glcm_ClusterShade	0.995
lbp-3D-m2_glcm_ClusterTendency	0.979
lbp-3D-m2_glcm_Contrast	0.978
lbp-3D-m2_glcm_Correlation	0.968
lbp-3D-m2_glcm_DifferenceAverage	0.973
lbp-3D-m2_glcm_DifferenceEntropy	0.985
lbp-3D-m2_glcm_DifferenceVariance	0.990
lbp-3D-m2_glcm_Id	0.967
lbp-3D-m2_glcm_Idm	0.969
lbp-3D-m2_glcm_Idmn	0.993
lbp-3D-m2_glcm_Idn	0.984
lbp-3D-m2_glcm_Imc1	0.998
lbp-3D-m2_glcm_Imc2	0.998
lbp-3D-m2_glcm_InverseVariance	0.993
lbp-3D-m2_glcm_JointAverage	0.996
lbp-3D-m2_glcm_JointEnergy	0.982
lbp-3D-m2_glcm_JointEntropy	0.984
lbp-3D-m2_glcm_MCC	0.998
lbp-3D-m2_glcm_MaximumProbability	0.979
lbp-3D-m2_glcm_SumAverage	0.996
lbp-3D-m2_glcm_SumEntropy	0.982
lbp-3D-m2_glcm_SumSquares	0.981
lbp-3D-m2_gldm_DependenceEntropy	0.957
lbp-3D-m2_gldm_DependenceNonUniformity	1.000
lbp-3D-m2_gldm_DependenceNonUniformityNormalized	0.966
lbp-3D-m2_gldm_DependenceVariance	0.963
lbp-3D-m2_gldm_GrayLevelNonUniformity	0.998
lbp-3D-m2_gldm_GrayLevelVariance	0.978
lbp-3D-m2_gldm_HighGrayLevelEmphasis	0.996
lbp-3D-m2_gldm_LargeDependenceEmphasis	0.946
lbp-3D-m2_gldm_LargeDependenceHighGrayLevelEmphasis	0.959
lbp-3D-m2_gldm_LargeDependenceLowGrayLevelEmphasis	0.993
lbp-3D-m2_gldm_LowGrayLevelEmphasis	0.990
lbp-3D-m2_gldm_SmallDependenceEmphasis	0.979
lbp-3D-m2_gldm_SmallDependenceHighGrayLevelEmphasis	0.979
lbp-3D-m2_gldm_SmallDependenceLowGrayLevelEmphasis	0.970
lbp-3D-m2_glrlm_GrayLevelNonUniformity	0.999
lbp-3D-m2_glrlm_GrayLevelNonUniformityNormalized	0.985
lbp-3D-m2_glrlm_GrayLevelVariance	0.980

lbp-3D-m2_glrlm_HighGrayLevelRunEmphasis	0.996
lbp-3D-m2_glrlm_LongRunEmphasis	0.987
lbp-3D-m2_glrlm_LongRunHighGrayLevelEmphasis	0.973
lbp-3D-m2_glrlm_LongRunLowGrayLevelEmphasis	1.000
lbp-3D-m2_glrlm_LowGrayLevelRunEmphasis	0.990
lbp-3D-m2_glrlm_RunEntropy	0.975
lbp-3D-m2_glrlm_RunLengthNonUniformity	1.000
lbp-3D-m2_glrlm_RunLengthNonUniformityNormalized	0.941
lbp-3D-m2_glrlm_RunPercentage	0.943
lbp-3D-m2_glrlm_RunVariance	0.984
lbp-3D-m2_glrlm_ShortRunEmphasis	0.954
lbp-3D-m2_glrlm_ShortRunHighGrayLevelEmphasis	0.997
lbp-3D-m2_glrlm_ShortRunLowGrayLevelEmphasis	0.981
lbp-3D-m2_glszm_GrayLevelNonUniformity	0.998
lbp-3D-m2_glszm_GrayLevelNonUniformityNormalized	0.959
lbp-3D-m2_glszm_GrayLevelVariance	0.974
lbp-3D-m2_glszm_HighGrayLevelZoneEmphasis	0.974
lbp-3D-m2_glszm_LargeAreaEmphasis	0.999
lbp-3D-m2_glszm_LargeAreaHighGrayLevelEmphasis	0.999
lbp-3D-m2_glszm_LargeAreaLowGrayLevelEmphasis	0.999
lbp-3D-m2_glszm_LowGrayLevelZoneEmphasis	0.968
lbp-3D-m2_glszm_SizeZoneNonUniformity	0.998
lbp-3D-m2_glszm_SizeZoneNonUniformityNormalized	0.977
lbp-3D-m2_glszm_SmallAreaEmphasis	0.971
lbp-3D-m2_glszm_SmallAreaHighGrayLevelEmphasis	0.913
lbp-3D-m2_glszm_SmallAreaLowGrayLevelEmphasis	0.983
lbp-3D-m2_glszm_ZoneEntropy	0.987
lbp-3D-m2_glszm_ZonePercentage	0.993
lbp-3D-m2_glszm_ZoneVariance	0.999
lbp-3D-m2_ngtdm_Busyness	1.000
lbp-3D-m2_ngtdm_Coarseness	0.998
lbp-3D-m2_ngtdm_Complexity	0.998
lbp-3D-m2_ngtdm_Contrast	0.989
lbp-3D-m2_ngtdm_Strength	0.997
lbp-3D-k_firstorder_10Percentile	0.972
lbp-3D-k_firstorder_90Percentile	0.997
lbp-3D-k_firstorder_Energy	0.998
lbp-3D-k_firstorder_Entropy	0.997
lbp-3D-k_firstorder_InterquartileRange	0.996
lbp-3D-k_firstorder_Kurtosis	0.997
lbp-3D-k_firstorder_Maximum	0.995
lbp-3D-k_firstorder_MeanAbsoluteDeviation	0.997
lbp-3D-k_firstorder_Mean	0.994
lbp-3D-k_firstorder_Median	0.992
lbp-3D-k_firstorder_Minimum	0.996
lbp-3D-k_firstorder_Range	0.996
lbp-3D-k_firstorder_RobustMeanAbsoluteDeviation	0.997
lbp-3D-k_firstorder_RootMeanSquared	0.984
lbp-3D-k_firstorder_Skewness	0.995
lbp-3D-k_firstorder_TotalEnergy	0.998

lbp-3D-k_firstorder_Uniformity	0.997
lbp-3D-k_firstorder_Variance	0.997
lbp-3D-k_glcm_Autocorrelation	0.997
lbp-3D-k_glcm_ClusterProminence	0.996
lbp-3D-k_glcm_ClusterShade	0.996
lbp-3D-k_glcm_ClusterTendency	0.997
lbp-3D-k_glcm_Contrast	0.997
lbp-3D-k_glcm_Correlation	0.988
lbp-3D-k_glcm_DifferenceAverage	0.997
lbp-3D-k_glcm_DifferenceEntropy	0.996
lbp-3D-k_glcm_DifferenceVariance	0.996
lbp-3D-k_glcm_Id	0.997
lbp-3D-k_glcm_Idm	0.997
lbp-3D-k_glcm_Idmn	0.998
lbp-3D-k_glcm_Idn	0.997
lbp-3D-k_glcm_Imc1	0.992
lbp-3D-k_glcm_Imc2	0.987
lbp-3D-k_glcm_InverseVariance	0.997
lbp-3D-k_glcm_JointAverage	0.997
lbp-3D-k_glcm_JointEnergy	0.997
lbp-3D-k_glcm_JointEntropy	0.997
lbp-3D-k_glcm_MCC	0.991
lbp-3D-k_glcm_MaximumProbability	0.997
lbp-3D-k_glcm_SumAverage	0.997
lbp-3D-k_glcm_SumEntropy	0.997
lbp-3D-k_glcm_SumSquares	0.997
lbp-3D-k_gldm_DependenceEntropy	0.995
lbp-3D-k_gldm_DependenceNonUniformity	0.998
lbp-3D-k_gldm_DependenceNonUniformityNormalized	0.990
lbp-3D-k_gldm_DependenceVariance	0.990
lbp-3D-k_gldm_GrayLevelNonUniformity	0.999
lbp-3D-k_gldm_GrayLevelVariance	0.997
lbp-3D-k_gldm_HighGrayLevelEmphasis	0.997
lbp-3D-k_gldm_LargeDependenceEmphasis	0.996
lbp-3D-k_gldm_LargeDependenceHighGrayLevelEmphasis	0.996
lbp-3D-k_gldm_LargeDependenceLowGrayLevelEmphasis	0.996
lbp-3D-k_gldm_LowGrayLevelEmphasis	0.997
lbp-3D-k_gldm_SmallDependenceEmphasis	0.969
lbp-3D-k_gldm_SmallDependenceHighGrayLevelEmphasis	0.963
lbp-3D-k_gldm_SmallDependenceLowGrayLevelEmphasis	0.983
lbp-3D-k_glrlm_GrayLevelNonUniformity	0.999
lbp-3D-k_glrlm_GrayLevelNonUniformityNormalized	0.999
lbp-3D-k_glrlm_GrayLevelVariance	0.999
lbp-3D-k_glrlm_HighGrayLevelRunEmphasis	0.998
lbp-3D-k_glrlm_LongRunEmphasis	0.997
lbp-3D-k_glrlm_LongRunHighGrayLevelEmphasis	0.997
lbp-3D-k_glrlm_LongRunLowGrayLevelEmphasis	0.997
lbp-3D-k_glrlm_LowGrayLevelRunEmphasis	0.998
lbp-3D-k_glrlm_RunEntropy	0.996
lbp-3D-k_glrlm_RunLengthNonUniformity	0.999

lbp-3D-k_glrlm_RunLengthNonUniformityNormalized	0.994
lbp-3D-k_glrlm_RunPercentage	0.996
lbp-3D-k_glrlm_RunVariance	0.998
lbp-3D-k_glrlm_ShortRunEmphasis	0.994
lbp-3D-k_glrlm_ShortRunHighGrayLevelEmphasis	0.997
lbp-3D-k_glrlm_ShortRunLowGrayLevelEmphasis	0.993
lbp-3D-k_glszm_GrayLevelNonUniformity	1.000
lbp-3D-k_glszm_GrayLevelNonUniformityNormalized	0.996
lbp-3D-k_glszm_GrayLevelVariance	0.996
lbp-3D-k_glszm_HighGrayLevelZoneEmphasis	0.996
lbp-3D-k_glszm_LargeAreaEmphasis	0.997
lbp-3D-k_glszm_LargeAreaHighGrayLevelEmphasis	0.997
lbp-3D-k_glszm_LargeAreaLowGrayLevelEmphasis	0.997
lbp-3D-k_glszm_LowGrayLevelZoneEmphasis	0.996
lbp-3D-k_glszm_SizeZoneNonUniformity	0.999
lbp-3D-k_glszm_SizeZoneNonUniformityNormalized	0.979
lbp-3D-k_glszm_SmallAreaEmphasis	0.980
lbp-3D-k_glszm_SmallAreaHighGrayLevelEmphasis	0.980
lbp-3D-k_glszm_SmallAreaLowGrayLevelEmphasis	0.981
lbp-3D-k_glszm_ZoneEntropy	0.994
lbp-3D-k_glszm_ZonePercentage	0.983
lbp-3D-k_glszm_ZoneVariance	0.997
lbp-3D-k_ngtdm_Busyness	0.999
lbp-3D-k_ngtdm_Coarseness	0.999
lbp-3D-k_ngtdm_Complexity	1.000
lbp-3D-k_ngtdm_Contrast	0.997
lbp-3D-k_ngtdm_Strength	0.999
logarithm_firstorder_10Percentile	1.000
logarithm_firstorder_90Percentile	1.000
logarithm_firstorder_Energy	0.999
logarithm_firstorder_Entropy	0.796
logarithm_firstorder_InterquartileRange	0.985
logarithm_firstorder_Kurtosis	0.615
logarithm_firstorder_Maximum	0.997
logarithm_firstorder_MeanAbsoluteDeviation	0.939
logarithm_firstorder_Mean	1.000
logarithm_firstorder_Median	1.000
logarithm_firstorder_Minimum	0.885
logarithm_firstorder_Range	0.955
logarithm_firstorder_RobustMeanAbsoluteDeviation	0.980
logarithm_firstorder_RootMeanSquared	1.000
logarithm_firstorder_Skewness	0.701
logarithm_firstorder_TotalEnergy	0.999
logarithm_firstorder_Uniformity	0.758
logarithm_firstorder_Variance	0.859
logarithm_glcm_Autocorrelation	0.850
logarithm_glcm_ClusterProminence	0.665
logarithm_glcm_ClusterShade	0.691
logarithm_glcm_ClusterTendency	0.722
logarithm_glcm_Contrast	0.805

logarithm_glcm_Correlation	0.900
logarithm_glcm_DifferenceAverage	0.805
logarithm_glcm_DifferenceEntropy	0.837
logarithm_glcm_DifferenceVariance	0.805
logarithm_glcm_Id	0.805
logarithm_glcm_Idm	0.805
logarithm_glcm_Idmn	0.805
logarithm_glcm_Idn	0.805
logarithm_glcm_Imc1	0.934
logarithm_glcm_Imc2	0.793
logarithm_glcm_InverseVariance	0.805
logarithm_glcm_JointAverage	0.850
logarithm_glcm_JointEnergy	0.773
logarithm_glcm_JointEntropy	0.802
logarithm_glcm_MCC	0.812
logarithm_glcm_MaximumProbability	0.773
logarithm_glcm_SumAverage	0.850
logarithm_glcm_SumEntropy	0.802
logarithm_glcm_SumSquares	0.751
logarithm_gldm_DependenceEntropy	0.998
logarithm_gldm_DependenceNonUniformity	0.999
logarithm_gldm_DependenceNonUniformityNormalized	0.998
logarithm_gldm_DependenceVariance	0.998
logarithm_gldm_GrayLevelNonUniformity	0.999
logarithm_gldm_GrayLevelVariance	0.758
logarithm_gldm_HighGrayLevelEmphasis	0.850
logarithm_gldm_LargeDependenceEmphasis	0.998
logarithm_gldm_LargeDependenceHighGrayLevelEmphasis	0.871
logarithm_gldm_LargeDependenceLowGrayLevelEmphasis	0.846
logarithm_gldm_LowGrayLevelEmphasis	0.850
logarithm_gldm_SmallDependenceEmphasis	0.995
logarithm_gldm_SmallDependenceHighGrayLevelEmphasis	0.811
logarithm_gldm_SmallDependenceLowGrayLevelEmphasis	0.953
logarithm_glrlm_GrayLevelNonUniformity	0.999
logarithm_glrlm_GrayLevelNonUniformityNormalized	0.783
logarithm_glrlm_GrayLevelVariance	0.783
logarithm_glrlm_HighGrayLevelRunEmphasis	0.848
logarithm_glrlm_LongRunEmphasis	0.999
logarithm_glrlm_LongRunHighGrayLevelEmphasis	0.982
logarithm_glrlm_LongRunLowGrayLevelEmphasis	0.983
logarithm_glrlm_LowGrayLevelRunEmphasis	0.848
logarithm_glrlm_RunEntropy	0.998
logarithm_glrlm_RunLengthNonUniformity	0.998
logarithm_glrlm_RunLengthNonUniformityNormalized	0.998
logarithm_glrlm_RunPercentage	0.998
logarithm_glrlm_RunVariance	0.999
logarithm_glrlm_ShortRunEmphasis	0.990
logarithm_glrlm_ShortRunHighGrayLevelEmphasis	0.835
logarithm_glrlm_ShortRunLowGrayLevelEmphasis	0.968
logarithm_glszm_GrayLevelNonUniformity	0.699

logarithm_glszm_GrayLevelNonUniformityNormalized	0.731
logarithm_glszm_GrayLevelVariance	0.731
logarithm_glszm_HighGrayLevelZoneEmphasis	0.671
logarithm_glszm_LargeAreaEmphasis	0.982
logarithm_glszm_LargeAreaHighGrayLevelEmphasis	0.971
logarithm_glszm_LargeAreaLowGrayLevelEmphasis	0.995
logarithm_glszm_LowGrayLevelZoneEmphasis	0.671
logarithm_glszm_SizeZoneNonUniformity	0.634
logarithm_glszm_SizeZoneNonUniformityNormalized	0.774
logarithm_glszm_SmallAreaEmphasis	0.766
logarithm_glszm_SmallAreaHighGrayLevelEmphasis	0.766
logarithm_glszm_SmallAreaLowGrayLevelEmphasis	0.766
logarithm_glszm_ZoneEntropy	0.881
logarithm_glszm_ZonePercentage	0.995
logarithm_glszm_ZoneVariance	0.994
logarithm_ngtdm_Busyness	0.789
logarithm_ngtdm_Coarseness	0.850
logarithm_ngtdm_Complexity	0.808
logarithm_ngtdm_Contrast	0.512
logarithm_ngtdm_Strength	0.583
square_firstorder_10Percentile	0.994
square_firstorder_90Percentile	0.999
square_firstorder_Energy	1.000
square_firstorder_Entropy	0.000
square_firstorder_InterquartileRange	0.987
square_firstorder_Kurtosis	0.855
square_firstorder_Maximum	0.986
square_firstorder_MeanAbsoluteDeviation	0.974
square_firstorder_Mean	0.998
square_firstorder_Median	0.999
square_firstorder_Minimum	0.899
square_firstorder_Range	0.969
square_firstorder_RobustMeanAbsoluteDeviation	0.986
square_firstorder_RootMeanSquared	0.999
square_firstorder_Skewness	0.896
square_firstorder_TotalEnergy	1.000
square_firstorder_Variance	0.968
square_gldm_DependenceEntropy	0.998
square_gldm_DependenceNonUniformity	0.999
square_gldm_DependenceNonUniformityNormalized	0.998
square_gldm_DependenceVariance	0.998
square_gldm_GrayLevelNonUniformity	0.999
square_gldm_LargeDependenceEmphasis	0.998
square_gldm_LargeDependenceHighGrayLevelEmphasis	0.998
square_gldm_LargeDependenceLowGrayLevelEmphasis	0.998
square_gldm_SmallDependenceEmphasis	0.995
square_gldm_SmallDependenceHighGrayLevelEmphasis	0.995
square_gldm_SmallDependenceLowGrayLevelEmphasis	0.995
square_glrlm_GrayLevelNonUniformity	0.999
square_glrlm_LongRunEmphasis	0.999

square_glrlm_LongRunHighGrayLevelEmphasis	0.999
square_glrlm_LongRunLowGrayLevelEmphasis	0.999
square_glrlm_RunEntropy	0.998
square_glrlm_RunLengthNonUniformity	0.998
square_glrlm_RunLengthNonUniformityNormalized	0.998
square_glrlm_RunPercentage	0.998
square_glrlm_RunVariance	0.999
square_glrlm_ShortRunEmphasis	0.991
square_glrlm_ShortRunHighGrayLevelEmphasis	0.991
square_glrlm_ShortRunLowGrayLevelEmphasis	0.991
square_glszm_GrayLevelNonUniformity	0.000
square_glszm_LargeAreaEmphasis	0.996
square_glszm_LargeAreaHighGrayLevelEmphasis	0.996
square_glszm_LargeAreaLowGrayLevelEmphasis	0.996
square_glszm_SizeZoneNonUniformityNormalized	0.000
square_glszm_SmallAreaEmphasis	0.000
square_glszm_SmallAreaHighGrayLevelEmphasis	0.000
square_glszm_SmallAreaLowGrayLevelEmphasis	0.000
square_glszm_ZoneEntropy	0.000
square_glszm_ZonePercentage	0.996
square_glszm_ZoneVariance	0.000
squareroot_firstorder_10Percentile	1.000
squareroot_firstorder_90Percentile	1.000
squareroot_firstorder_Energy	0.999
squareroot_firstorder_Entropy	0.796
squareroot_firstorder_InterquartileRange	0.987
squareroot_firstorder_Kurtosis	0.764
squareroot_firstorder_Maximum	0.998
squareroot_firstorder_MeanAbsoluteDeviation	0.943
squareroot_firstorder_Mean	1.000
squareroot_firstorder_Median	1.000
squareroot_firstorder_Minimum	0.880
squareroot_firstorder_Range	0.943
squareroot_firstorder_RobustMeanAbsoluteDeviation	0.982
squareroot_firstorder_RootMeanSquared	1.000
squareroot_firstorder_Skewness	0.742
squareroot_firstorder_TotalEnergy	0.999
squareroot_firstorder_Uniformity	0.758
squareroot_firstorder_Variance	0.849
squareroot_glcm_Autocorrelation	0.850
squareroot_glcm_ClusterProminence	0.665
squareroot_glcm_ClusterShade	0.691
squareroot_glcm_ClusterTendency	0.722
squareroot_glcm_Contrast	0.805
squareroot_glcm_Correlation	0.900
squareroot_glcm_DifferenceAverage	0.805
squareroot_glcm_DifferenceEntropy	0.837
squareroot_glcm_DifferenceVariance	0.805
squareroot_glcm_Id	0.805
squareroot_glcm_Idm	0.805

squareroot_glcm_Idmn	0.805
squareroot_glcm_Idn	0.805
squareroot_glcm_Imc1	0.934
squareroot_glcm_Imc2	0.793
squareroot_glcm_InverseVariance	0.805
squareroot_glcm_JointAverage	0.850
squareroot_glcm_JointEnergy	0.773
squareroot_glcm_JointEntropy	0.802
squareroot_glcm_MCC	0.812
squareroot_glcm_MaximumProbability	0.773
squareroot_glcm_SumAverage	0.850
squareroot_glcm_SumEntropy	0.802
squareroot_glcm_SumSquares	0.751
squareroot_gldm_DependenceEntropy	0.998
squareroot_gldm_DependenceNonUniformity	0.999
squareroot_gldm_DependenceNonUniformityNormalized	0.998
squareroot_gldm_DependenceVariance	0.998
squareroot_gldm_GrayLevelNonUniformity	0.999
squareroot_gldm_GrayLevelVariance	0.758
squareroot_gldm_HighGrayLevelEmphasis	0.850
squareroot_gldm_LargeDependenceEmphasis	0.998
squareroot_gldm_LargeDependenceHighGrayLevelEmphasis	0.871
squareroot_gldm_LargeDependenceLowGrayLevelEmphasis	0.846
squareroot_gldm_LowGrayLevelEmphasis	0.850
squareroot_gldm_SmallDependenceEmphasis	0.995
squareroot_gldm_SmallDependenceHighGrayLevelEmphasis	0.811
squareroot_gldm_SmallDependenceLowGrayLevelEmphasis	0.953
squareroot_glrlm_GrayLevelNonUniformity	0.999
squareroot_glrlm_GrayLevelNonUniformityNormalized	0.783
squareroot_glrlm_GrayLevelVariance	0.783
squareroot_glrlm_HighGrayLevelRunEmphasis	0.848
squareroot_glrlm_LongRunEmphasis	0.999
squareroot_glrlm_LongRunHighGrayLevelEmphasis	0.982
squareroot_glrlm_LongRunLowGrayLevelEmphasis	0.983
squareroot_glrlm_LowGrayLevelRunEmphasis	0.848
squareroot_glrlm_RunEntropy	0.998
squareroot_glrlm_RunLengthNonUniformity	0.998
squareroot_glrlm_RunLengthNonUniformityNormalized	0.998
squareroot_glrlm_RunPercentage	0.998
squareroot_glrlm_RunVariance	0.999
squareroot_glrlm_ShortRunEmphasis	0.990
squareroot_glrlm_ShortRunHighGrayLevelEmphasis	0.835
squareroot_glrlm_ShortRunLowGrayLevelEmphasis	0.968
squareroot_glszm_GrayLevelNonUniformity	0.699
squareroot_glszm_GrayLevelNonUniformityNormalized	0.731
squareroot_glszm_GrayLevelVariance	0.731
squareroot_glszm_HighGrayLevelZoneEmphasis	0.671
squareroot_glszm_LargeAreaEmphasis	0.982
squareroot_glszm_LargeAreaHighGrayLevelEmphasis	0.971
squareroot_glszm_LargeAreaLowGrayLevelEmphasis	0.995

squareroot_glszm_LowGrayLevelZoneEmphasis	0.671
squareroot_glszm_SizeZoneNonUniformity	0.634
squareroot_glszm_SizeZoneNonUniformityNormalized	0.774
squareroot_glszm_SmallAreaEmphasis	0.766
squareroot_glszm_SmallAreaHighGrayLevelEmphasis	0.766
squareroot_glszm_SmallAreaLowGrayLevelEmphasis	0.766
squareroot_glszm_ZoneEntropy	0.881
squareroot_glszm_ZonePercentage	0.995
squareroot_glszm_ZoneVariance	0.994
squareroot_ngtdm_Busyness	0.789
squareroot_ngtdm_Coarseness	0.850
squareroot_ngtdm_Complexity	0.808
squareroot_ngtdm_Contrast	0.512
squareroot_ngtdm_Strength	0.583
wavelet-LLH_firstorder_10Percentile	0.991
wavelet-LLH_firstorder_90Percentile	0.985
wavelet-LLH_firstorder_Energy	0.978
wavelet-LLH_firstorder_Entropy	0.983
wavelet-LLH_firstorder_InterquartileRange	0.991
wavelet-LLH_firstorder_Kurtosis	0.968
wavelet-LLH_firstorder_Maximum	0.971
wavelet-LLH_firstorder_MeanAbsoluteDeviation	0.992
wavelet-LLH_firstorder_Mean	0.994
wavelet-LLH_firstorder_Median	0.998
wavelet-LLH_firstorder_Minimum	0.978
wavelet-LLH_firstorder_Range	0.981
wavelet-LLH_firstorder_RobustMeanAbsoluteDeviation	0.991
wavelet-LLH_firstorder_RootMeanSquared	0.991
wavelet-LLH_firstorder_Skewness	0.966
wavelet-LLH_firstorder_TotalEnergy	0.978
wavelet-LLH_firstorder_Uniformity	0.984
wavelet-LLH_firstorder_Variance	0.984
wavelet-LLH_glcm_Autocorrelation	0.995
wavelet-LLH_glcm_ClusterProminence	0.996
wavelet-LLH_glcm_ClusterShade	0.996
wavelet-LLH_glcm_ClusterTendency	0.994
wavelet-LLH_glcm_Contrast	0.998
wavelet-LLH_glcm_Correlation	0.998
wavelet-LLH_glcm_DifferenceAverage	0.998
wavelet-LLH_glcm_DifferenceEntropy	0.997
wavelet-LLH_glcm_DifferenceVariance	0.997
wavelet-LLH_glcm_Id	0.998
wavelet-LLH_glcm_Idm	0.998
wavelet-LLH_glcm_Idmn	0.998
wavelet-LLH_glcm_Idn	0.998
wavelet-LLH_glcm_Imc1	0.998
wavelet-LLH_glcm_Imc2	0.998
wavelet-LLH_glcm_InverseVariance	0.998
wavelet-LLH_glcm_JointAverage	0.995
wavelet-LLH_glcm_JointEnergy	0.993

wavelet-LLH_glcm_JointEntropy	0.993
wavelet-LLH_glcm_MCC	0.998
wavelet-LLH_glcm_MaximumProbability	0.996
wavelet-LLH_glcm_SumAverage	0.995
wavelet-LLH_glcm_SumEntropy	0.992
wavelet-LLH_glcm_SumSquares	0.992
wavelet-LLH_gldm_DependenceEntropy	0.995
wavelet-LLH_gldm_DependenceNonUniformity	0.998
wavelet-LLH_gldm_DependenceNonUniformityNormalized	0.990
wavelet-LLH_gldm_DependenceVariance	0.992
wavelet-LLH_gldm_GrayLevelNonUniformity	0.999
wavelet-LLH_gldm_GrayLevelVariance	0.984
wavelet-LLH_gldm_HighGrayLevelEmphasis	0.991
wavelet-LLH_gldm_LargeDependenceEmphasis	0.998
wavelet-LLH_gldm_LargeDependenceHighGrayLevelEmphasis	0.998
wavelet-LLH_gldm_LargeDependenceLowGrayLevelEmphasis	0.997
wavelet-LLH_gldm_LowGrayLevelEmphasis	0.991
wavelet-LLH_gldm_SmallDependenceEmphasis	0.975
wavelet-LLH_gldm_SmallDependenceHighGrayLevelEmphasis	0.953
wavelet-LLH_gldm_SmallDependenceLowGrayLevelEmphasis	0.984
wavelet-LLH_glrlm_GrayLevelNonUniformity	0.999
wavelet-LLH_glrlm_GrayLevelNonUniformityNormalized	0.858
wavelet-LLH_glrlm_GrayLevelVariance	0.858
wavelet-LLH_glrlm_HighGrayLevelRunEmphasis	0.924
wavelet-LLH_glrlm_LongRunEmphasis	0.995
wavelet-LLH_glrlm_LongRunHighGrayLevelEmphasis	0.995
wavelet-LLH_glrlm_LongRunLowGrayLevelEmphasis	0.994
wavelet-LLH_glrlm_LowGrayLevelRunEmphasis	0.924
wavelet-LLH_glrlm_RunEntropy	0.998
wavelet-LLH_glrlm_RunLengthNonUniformity	0.999
wavelet-LLH_glrlm_RunLengthNonUniformityNormalized	0.998
wavelet-LLH_glrlm_RunPercentage	0.998
wavelet-LLH_glrlm_RunVariance	0.992
wavelet-LLH_glrlm_ShortRunEmphasis	0.996
wavelet-LLH_glrlm_ShortRunHighGrayLevelEmphasis	0.993
wavelet-LLH_glrlm_ShortRunLowGrayLevelEmphasis	0.983
wavelet-LLH_glszm_GrayLevelNonUniformity	0.973
wavelet-LLH_glszm_GrayLevelNonUniformityNormalized	0.712
wavelet-LLH_glszm_GrayLevelVariance	0.712
wavelet-LLH_glszm_HighGrayLevelZoneEmphasis	0.736
wavelet-LLH_glszm_LargeAreaEmphasis	0.986
wavelet-LLH_glszm_LargeAreaHighGrayLevelEmphasis	0.983
wavelet-LLH_glszm_LargeAreaLowGrayLevelEmphasis	0.988
wavelet-LLH_glszm_LowGrayLevelZoneEmphasis	0.736
wavelet-LLH_glszm_SizeZoneNonUniformity	0.967
wavelet-LLH_glszm_SizeZoneNonUniformityNormalized	0.772
wavelet-LLH_glszm_SmallAreaEmphasis	0.664
wavelet-LLH_glszm_SmallAreaHighGrayLevelEmphasis	0.626
wavelet-LLH_glszm_SmallAreaLowGrayLevelEmphasis	0.715
wavelet-LLH_glszm_ZoneEntropy	0.911

wavelet-LLH_glszm_ZonePercentage	0.870
wavelet-LLH_glszm_ZoneVariance	0.986
wavelet-LLH_ngtdm_Busyness	0.872
wavelet-LLH_ngtdm_Coarseness	0.990
wavelet-LLH_ngtdm_Complexity	0.997
wavelet-LLH_ngtdm_Contrast	0.997
wavelet-LLH_ngtdm_Strength	0.992
wavelet-LHL_firstorder_10Percentile	0.997
wavelet-LHL_firstorder_90Percentile	0.989
wavelet-LHL_firstorder_Energy	0.995
wavelet-LHL_firstorder_Entropy	0.950
wavelet-LHL_firstorder_InterquartileRange	0.998
wavelet-LHL_firstorder_Kurtosis	0.809
wavelet-LHL_firstorder_Maximum	0.948
wavelet-LHL_firstorder_MeanAbsoluteDeviation	0.990
wavelet-LHL_firstorder_Mean	0.870
wavelet-LHL_firstorder_Median	0.970
wavelet-LHL_firstorder_Minimum	0.931
wavelet-LHL_firstorder_Range	0.949
wavelet-LHL_firstorder_RobustMeanAbsoluteDeviation	0.998
wavelet-LHL_firstorder_RootMeanSquared	0.974
wavelet-LHL_firstorder_Skewness	0.856
wavelet-LHL_firstorder_TotalEnergy	0.995
wavelet-LHL_firstorder_Uniformity	0.950
wavelet-LHL_firstorder_Variance	0.974
wavelet-LHL_glcm_Autocorrelation	0.961
wavelet-LHL_glcm_ClusterProminence	0.909
wavelet-LHL_glcm_ClusterShade	0.978
wavelet-LHL_glcm_ClusterTendency	0.881
wavelet-LHL_glcm_Contrast	0.970
wavelet-LHL_glcm_Correlation	0.942
wavelet-LHL_glcm_DifferenceAverage	0.970
wavelet-LHL_glcm_DifferenceEntropy	0.997
wavelet-LHL_glcm_DifferenceVariance	0.997
wavelet-LHL_glcm_Id	0.970
wavelet-LHL_glcm_Idm	0.970
wavelet-LHL_glcm_Idmn	0.970
wavelet-LHL_glcm_Idn	0.970
wavelet-LHL_glcm_Imc1	0.997
wavelet-LHL_glcm_Imc2	0.993
wavelet-LHL_glcm_InverseVariance	0.970
wavelet-LHL_glcm_JointAverage	0.968
wavelet-LHL_glcm_JointEnergy	0.962
wavelet-LHL_glcm_JointEntropy	0.970
wavelet-LHL_glcm_MCC	0.994
wavelet-LHL_glcm_MaximumProbability	0.967
wavelet-LHL_glcm_SumAverage	0.968
wavelet-LHL_glcm_SumEntropy	0.950
wavelet-LHL_glcm_SumSquares	0.928
wavelet-LHL_gldm_DependenceEntropy	0.951

wavelet-LHL_gldm_DependenceNonUniformity	0.999
wavelet-LHL_gldm_DependenceNonUniformityNormalized	0.986
wavelet-LHL_gldm_DependenceVariance	0.981
wavelet-LHL_gldm_GrayLevelNonUniformity	0.999
wavelet-LHL_gldm_GrayLevelVariance	0.950
wavelet-LHL_gldm_HighGrayLevelEmphasis	0.974
wavelet-LHL_gldm_LargeDependenceEmphasis	0.980
wavelet-LHL_gldm_LargeDependenceHighGrayLevelEmphasis	0.974
wavelet-LHL_gldm_LargeDependenceLowGrayLevelEmphasis	0.973
wavelet-LHL_gldm_LowGrayLevelEmphasis	0.974
wavelet-LHL_gldm_SmallDependenceEmphasis	0.994
wavelet-LHL_gldm_SmallDependenceHighGrayLevelEmphasis	0.990
wavelet-LHL_gldm_SmallDependenceLowGrayLevelEmphasis	0.995
wavelet-LHL_glrlm_GrayLevelNonUniformity	0.999
wavelet-LHL_glrlm_GrayLevelNonUniformityNormalized	0.991
wavelet-LHL_glrlm_GrayLevelVariance	0.991
wavelet-LHL_glrlm_HighGrayLevelRunEmphasis	0.988
wavelet-LHL_glrlm_LongRunEmphasis	0.999
wavelet-LHL_glrlm_LongRunHighGrayLevelEmphasis	0.998
wavelet-LHL_glrlm_LongRunLowGrayLevelEmphasis	0.999
wavelet-LHL_glrlm_LowGrayLevelRunEmphasis	0.988
wavelet-LHL_glrlm_RunEntropy	0.987
wavelet-LHL_glrlm_RunLengthNonUniformity	0.999
wavelet-LHL_glrlm_RunLengthNonUniformityNormalized	0.982
wavelet-LHL_glrlm_RunPercentage	0.984
wavelet-LHL_glrlm_RunVariance	0.998
wavelet-LHL_glrlm_ShortRunEmphasis	0.988
wavelet-LHL_glrlm_ShortRunHighGrayLevelEmphasis	0.984
wavelet-LHL_glrlm_ShortRunLowGrayLevelEmphasis	0.984
wavelet-LHL_glszm_GrayLevelNonUniformity	0.967
wavelet-LHL_glszm_GrayLevelNonUniformityNormalized	0.836
wavelet-LHL_glszm_GrayLevelVariance	0.836
wavelet-LHL_glszm_HighGrayLevelZoneEmphasis	0.889
wavelet-LHL_glszm_LargeAreaEmphasis	0.995
wavelet-LHL_glszm_LargeAreaHighGrayLevelEmphasis	0.995
wavelet-LHL_glszm_LargeAreaLowGrayLevelEmphasis	0.995
wavelet-LHL_glszm_LowGrayLevelZoneEmphasis	0.889
wavelet-LHL_glszm_SizeZoneNonUniformity	0.925
wavelet-LHL_glszm_SizeZoneNonUniformityNormalized	0.707
wavelet-LHL_glszm_SmallAreaEmphasis	0.767
wavelet-LHL_glszm_SmallAreaHighGrayLevelEmphasis	0.860
wavelet-LHL_glszm_SmallAreaLowGrayLevelEmphasis	0.636
wavelet-LHL_glszm_ZoneEntropy	0.855
wavelet-LHL_glszm_ZonePercentage	0.974
wavelet-LHL_glszm_ZoneVariance	0.996
wavelet-LHL_ngtdm_Busyness	0.999
wavelet-LHL_ngtdm_Coarseness	0.998
wavelet-LHL_ngtdm_Complexity	0.970
wavelet-LHL_ngtdm_Contrast	0.981
wavelet-LHL_ngtdm_Strength	0.998

wavelet-LHH_firstorder_10Percentile	0.994
wavelet-LHH_firstorder_90Percentile	0.996
wavelet-LHH_firstorder_Energy	0.960
wavelet-LHH_firstorder_Entropy	0.739
wavelet-LHH_firstorder_InterquartileRange	0.998
wavelet-LHH_firstorder_Kurtosis	0.940
wavelet-LHH_firstorder_Maximum	0.964
wavelet-LHH_firstorder_MeanAbsoluteDeviation	0.991
wavelet-LHH_firstorder_Mean	0.886
wavelet-LHH_firstorder_Median	0.878
wavelet-LHH_firstorder_Minimum	0.915
wavelet-LHH_firstorder_Range	0.959
wavelet-LHH_firstorder_RobustMeanAbsoluteDeviation	0.998
wavelet-LHH_firstorder_RootMeanSquared	0.974
wavelet-LHH_firstorder_Skewness	0.929
wavelet-LHH_firstorder_TotalEnergy	0.960
wavelet-LHH_firstorder_Uniformity	0.739
wavelet-LHH_firstorder_Variance	0.935
wavelet-LHH_glcm_Autocorrelation	0.918
wavelet-LHH_glcm_ClusterProminence	0.996
wavelet-LHH_glcm_ClusterShade	0.915
wavelet-LHH_glcm_ClusterTendency	0.996
wavelet-LHH_glcm_Contrast	0.997
wavelet-LHH_glcm_Correlation	0.997
wavelet-LHH_glcm_DifferenceAverage	0.997
wavelet-LHH_glcm_DifferenceEntropy	1.000
wavelet-LHH_glcm_DifferenceVariance	1.000
wavelet-LHH_glcm_Id	0.997
wavelet-LHH_glcm_Idm	0.997
wavelet-LHH_glcm_Idmn	0.997
wavelet-LHH_glcm_Idn	0.997
wavelet-LHH_glcm_Imc1	1.000
wavelet-LHH_glcm_Imc2	0.999
wavelet-LHH_glcm_InverseVariance	0.997
wavelet-LHH_glcm_JointAverage	0.923
wavelet-LHH_glcm_JointEnergy	0.998
wavelet-LHH_glcm_JointEntropy	0.998
wavelet-LHH_glcm_MCC	0.999
wavelet-LHH_glcm_MaximumProbability	0.977
wavelet-LHH_glcm_SumAverage	0.923
wavelet-LHH_glcm_SumEntropy	0.993
wavelet-LHH_glcm_SumSquares	0.751
wavelet-LHH_gldm_DependenceEntropy	0.998
wavelet-LHH_gldm_DependenceNonUniformity	0.999
wavelet-LHH_gldm_DependenceNonUniformityNormalized	0.999
wavelet-LHH_gldm_DependenceVariance	0.999
wavelet-LHH_gldm_GrayLevelNonUniformity	0.999
wavelet-LHH_gldm_GrayLevelVariance	0.739
wavelet-LHH_gldm_HighGrayLevelEmphasis	0.922
wavelet-LHH_gldm_LargeDependenceEmphasis	0.997

wavelet-LHH_gldm_LargeDependenceHighGrayLevelEmphasis	0.995
wavelet-LHH_gldm_LargeDependenceLowGrayLevelEmphasis	0.995
wavelet-LHH_gldm_LowGrayLevelEmphasis	0.922
wavelet-LHH_gldm_SmallDependenceEmphasis	0.989
wavelet-LHH_gldm_SmallDependenceHighGrayLevelEmphasis	0.979
wavelet-LHH_gldm_SmallDependenceLowGrayLevelEmphasis	0.991
wavelet-LHH_glrlm_GrayLevelNonUniformity	0.999
wavelet-LHH_glrlm_GrayLevelNonUniformityNormalized	0.865
wavelet-LHH_glrlm_GrayLevelVariance	0.865
wavelet-LHH_glrlm_HighGrayLevelRunEmphasis	0.934
wavelet-LHH_glrlm_LongRunEmphasis	0.999
wavelet-LHH_glrlm_LongRunHighGrayLevelEmphasis	0.999
wavelet-LHH_glrlm_LongRunLowGrayLevelEmphasis	0.998
wavelet-LHH_glrlm_LowGrayLevelRunEmphasis	0.934
wavelet-LHH_glrlm_RunEntropy	0.997
wavelet-LHH_glrlm_RunLengthNonUniformity	0.999
wavelet-LHH_glrlm_RunLengthNonUniformityNormalized	0.996
wavelet-LHH_glrlm_RunPercentage	0.997
wavelet-LHH_glrlm_RunVariance	0.996
wavelet-LHH_glrlm_ShortRunEmphasis	0.997
wavelet-LHH_glrlm_ShortRunHighGrayLevelEmphasis	0.995
wavelet-LHH_glrlm_ShortRunLowGrayLevelEmphasis	0.992
wavelet-LHH_glszm_GrayLevelNonUniformity	0.978
wavelet-LHH_glszm_GrayLevelNonUniformityNormalized	0.612
wavelet-LHH_glszm_GrayLevelVariance	0.612
wavelet-LHH_glszm_HighGrayLevelZoneEmphasis	0.728
wavelet-LHH_glszm_LargeAreaEmphasis	0.969
wavelet-LHH_glszm_LargeAreaHighGrayLevelEmphasis	0.970
wavelet-LHH_glszm_LargeAreaLowGrayLevelEmphasis	0.969
wavelet-LHH_glszm_LowGrayLevelZoneEmphasis	0.728
wavelet-LHH_glszm_SizeZoneNonUniformity	0.953
wavelet-LHH_glszm_SizeZoneNonUniformityNormalized	0.725
wavelet-LHH_glszm_SmallAreaEmphasis	0.746
wavelet-LHH_glszm_SmallAreaHighGrayLevelEmphasis	0.732
wavelet-LHH_glszm_SmallAreaLowGrayLevelEmphasis	0.737
wavelet-LHH_glszm_ZoneEntropy	0.911
wavelet-LHH_glszm_ZonePercentage	0.980
wavelet-LHH_glszm_ZoneVariance	0.979
wavelet-LHH_ngtdm_Busyness	0.999
wavelet-LHH_ngtdm_Coarseness	0.997
wavelet-LHH_ngtdm_Complexity	0.997
wavelet-LHH_ngtdm_Contrast	0.997
wavelet-LHH_ngtdm_Strength	0.997
wavelet-HLL_firstorder_10Percentile	0.989
wavelet-HLL_firstorder_90Percentile	0.997
wavelet-HLL_firstorder_Energy	0.965
wavelet-HLL_firstorder_Entropy	0.991
wavelet-HLL_firstorder_InterquartileRange	0.998
wavelet-HLL_firstorder_Kurtosis	0.693
wavelet-HLL_firstorder_Maximum	0.904

wavelet-HLL_firstorder_MeanAbsoluteDeviation	0.987
wavelet-HLL_firstorder_Mean	0.977
wavelet-HLL_firstorder_Median	0.990
wavelet-HLL_firstorder_Minimum	0.887
wavelet-HLL_firstorder_Range	0.908
wavelet-HLL_firstorder_RobustMeanAbsoluteDeviation	0.997
wavelet-HLL_firstorder_RootMeanSquared	0.966
wavelet-HLL_firstorder_Skewness	0.809
wavelet-HLL_firstorder_TotalEnergy	0.965
wavelet-HLL_firstorder_Uniformity	0.991
wavelet-HLL_firstorder_Variance	0.953
wavelet-HLL_glcm_Autocorrelation	0.994
wavelet-HLL_glcm_ClusterProminence	0.956
wavelet-HLL_glcm_ClusterShade	0.991
wavelet-HLL_glcm_ClusterTendency	0.957
wavelet-HLL_glcm_Contrast	0.971
wavelet-HLL_glcm_Correlation	0.960
wavelet-HLL_glcm_DifferenceAverage	0.971
wavelet-HLL_glcm_DifferenceEntropy	0.997
wavelet-HLL_glcm_DifferenceVariance	0.997
wavelet-HLL_glcm_Id	0.971
wavelet-HLL_glcm_Idm	0.971
wavelet-HLL_glcm_Idmn	0.971
wavelet-HLL_glcm_Idn	0.971
wavelet-HLL_glcm_Imc1	0.997
wavelet-HLL_glcm_Imc2	0.985
wavelet-HLL_glcm_InverseVariance	0.971
wavelet-HLL_glcm_JointAverage	0.993
wavelet-HLL_glcm_JointEnergy	0.992
wavelet-HLL_glcm_JointEntropy	0.993
wavelet-HLL_glcm_MCC	0.987
wavelet-HLL_glcm_MaximumProbability	0.988
wavelet-HLL_glcm_SumAverage	0.993
wavelet-HLL_glcm_SumEntropy	0.978
wavelet-HLL_glcm_SumSquares	0.991
wavelet-HLL_gldm_DependenceEntropy	0.978
wavelet-HLL_gldm_DependenceNonUniformity	0.999
wavelet-HLL_gldm_DependenceNonUniformityNormalized	0.988
wavelet-HLL_gldm_DependenceVariance	0.979
wavelet-HLL_gldm_GrayLevelNonUniformity	0.999
wavelet-HLL_gldm_GrayLevelVariance	0.991
wavelet-HLL_gldm_HighGrayLevelEmphasis	0.993
wavelet-HLL_gldm_LargeDependenceEmphasis	0.977
wavelet-HLL_gldm_LargeDependenceHighGrayLevelEmphasis	0.995
wavelet-HLL_gldm_LargeDependenceLowGrayLevelEmphasis	0.966
wavelet-HLL_gldm_LowGrayLevelEmphasis	0.993
wavelet-HLL_gldm_SmallDependenceEmphasis	0.993
wavelet-HLL_gldm_SmallDependenceHighGrayLevelEmphasis	0.991
wavelet-HLL_gldm_SmallDependenceLowGrayLevelEmphasis	0.991
wavelet-HLL_glrlm_GrayLevelNonUniformity	0.999

wavelet-HLL_glrlm_GrayLevelNonUniformityNormalized	0.984
wavelet-HLL_glrlm_GrayLevelVariance	0.984
wavelet-HLL_glrlm_HighGrayLevelRunEmphasis	0.984
wavelet-HLL_glrlm_LongRunEmphasis	0.998
wavelet-HLL_glrlm_LongRunHighGrayLevelEmphasis	0.999
wavelet-HLL_glrlm_LongRunLowGrayLevelEmphasis	0.997
wavelet-HLL_glrlm_LowGrayLevelRunEmphasis	0.984
wavelet-HLL_glrlm_RunEntropy	0.990
wavelet-HLL_glrlm_RunLengthNonUniformity	1.000
wavelet-HLL_glrlm_RunLengthNonUniformityNormalized	0.986
wavelet-HLL_glrlm_RunPercentage	0.984
wavelet-HLL_glrlm_RunVariance	0.997
wavelet-HLL_glrlm_ShortRunEmphasis	0.990
wavelet-HLL_glrlm_ShortRunHighGrayLevelEmphasis	0.994
wavelet-HLL_glrlm_ShortRunLowGrayLevelEmphasis	0.977
wavelet-HLL_glszm_GrayLevelNonUniformity	0.939
wavelet-HLL_glszm_GrayLevelNonUniformityNormalized	0.896
wavelet-HLL_glszm_GrayLevelVariance	0.896
wavelet-HLL_glszm_HighGrayLevelZoneEmphasis	0.916
wavelet-HLL_glszm_LargeAreaEmphasis	0.986
wavelet-HLL_glszm_LargeAreaHighGrayLevelEmphasis	0.985
wavelet-HLL_glszm_LargeAreaLowGrayLevelEmphasis	0.987
wavelet-HLL_glszm_LowGrayLevelZoneEmphasis	0.916
wavelet-HLL_glszm_SizeZoneNonUniformity	0.884
wavelet-HLL_glszm_SizeZoneNonUniformityNormalized	0.625
wavelet-HLL_glszm_SmallAreaEmphasis	0.695
wavelet-HLL_glszm_SmallAreaHighGrayLevelEmphasis	0.767
wavelet-HLL_glszm_SmallAreaLowGrayLevelEmphasis	0.814
wavelet-HLL_glszm_ZoneEntropy	0.777
wavelet-HLL_glszm_ZonePercentage	0.956
wavelet-HLL_glszm_ZoneVariance	0.990
wavelet-HLL_ngtdm_Busyness	0.998
wavelet-HLL_ngtdm_Coarseness	0.997
wavelet-HLL_ngtdm_Complexity	0.975
wavelet-HLL_ngtdm_Contrast	0.983
wavelet-HLL_ngtdm_Strength	0.997
wavelet-HLH_firstorder_10Percentile	0.996
wavelet-HLH_firstorder_90Percentile	0.980
wavelet-HLH_firstorder_Energy	0.949
wavelet-HLH_firstorder_Entropy	0.960
wavelet-HLH_firstorder_InterquartileRange	0.998
wavelet-HLH_firstorder_Kurtosis	0.946
wavelet-HLH_firstorder_Maximum	0.947
wavelet-HLH_firstorder_MeanAbsoluteDeviation	0.990
wavelet-HLH_firstorder_Mean	0.945
wavelet-HLH_firstorder_Median	0.988
wavelet-HLH_firstorder_Minimum	0.948
wavelet-HLH_firstorder_Range	0.956
wavelet-HLH_firstorder_RobustMeanAbsoluteDeviation	0.997
wavelet-HLH_firstorder_RootMeanSquared	0.981

wavelet-HLH_firstorder_Skewness	0.938
wavelet-HLH_firstorder_TotalEnergy	0.949
wavelet-HLH_firstorder_Uniformity	0.960
wavelet-HLH_firstorder_Variance	0.977
wavelet-HLH_glcm_Autocorrelation	0.986
wavelet-HLH_glcm_ClusterProminence	0.992
wavelet-HLH_glcm_ClusterShade	0.985
wavelet-HLH_glcm_ClusterTendency	0.992
wavelet-HLH_glcm_Contrast	0.992
wavelet-HLH_glcm_Correlation	0.992
wavelet-HLH_glcm_DifferenceAverage	0.992
wavelet-HLH_glcm_DifferenceEntropy	1.000
wavelet-HLH_glcm_DifferenceVariance	1.000
wavelet-HLH_glcm_Id	0.992
wavelet-HLH_glcm_Idm	0.992
wavelet-HLH_glcm_Idmn	0.992
wavelet-HLH_glcm_Idn	0.992
wavelet-HLH_glcm_Imc1	1.000
wavelet-HLH_glcm_Imc2	0.999
wavelet-HLH_glcm_InverseVariance	0.992
wavelet-HLH_glcm_JointAverage	0.988
wavelet-HLH_glcm_JointEnergy	1.000
wavelet-HLH_glcm_JointEntropy	1.000
wavelet-HLH_glcm_MCC	0.999
wavelet-HLH_glcm_MaximumProbability	0.994
wavelet-HLH_glcm_SumAverage	0.988
wavelet-HLH_glcm_SumEntropy	0.985
wavelet-HLH_glcm_SumSquares	0.982
wavelet-HLH_gldm_DependenceEntropy	0.998
wavelet-HLH_gldm_DependenceNonUniformity	0.999
wavelet-HLH_gldm_DependenceNonUniformityNormalized	0.999
wavelet-HLH_gldm_DependenceVariance	0.997
wavelet-HLH_gldm_GrayLevelNonUniformity	0.999
wavelet-HLH_gldm_GrayLevelVariance	0.960
wavelet-HLH_gldm_HighGrayLevelEmphasis	0.978
wavelet-HLH_gldm_LargeDependenceEmphasis	0.996
wavelet-HLH_gldm_LargeDependenceHighGrayLevelEmphasis	0.995
wavelet-HLH_gldm_LargeDependenceLowGrayLevelEmphasis	0.997
wavelet-HLH_gldm_LowGrayLevelEmphasis	0.978
wavelet-HLH_gldm_SmallDependenceEmphasis	0.996
wavelet-HLH_gldm_SmallDependenceHighGrayLevelEmphasis	0.996
wavelet-HLH_gldm_SmallDependenceLowGrayLevelEmphasis	0.994
wavelet-HLH_glrlm_GrayLevelNonUniformity	0.999
wavelet-HLH_glrlm_GrayLevelNonUniformityNormalized	0.964
wavelet-HLH_glrlm_GrayLevelVariance	0.964
wavelet-HLH_glrlm_HighGrayLevelRunEmphasis	0.953
wavelet-HLH_glrlm_LongRunEmphasis	0.998
wavelet-HLH_glrlm_LongRunHighGrayLevelEmphasis	0.998
wavelet-HLH_glrlm_LongRunLowGrayLevelEmphasis	0.998
wavelet-HLH_glrlm_LowGrayLevelRunEmphasis	0.953

wavelet-HLH_glrlm_RunEntropy	0.997
wavelet-HLH_glrlm_RunLengthNonUniformity	0.999
wavelet-HLH_glrlm_RunLengthNonUniformityNormalized	0.996
wavelet-HLH_glrlm_RunPercentage	0.996
wavelet-HLH_glrlm_RunVariance	0.996
wavelet-HLH_glrlm_ShortRunEmphasis	0.997
wavelet-HLH_glrlm_ShortRunHighGrayLevelEmphasis	0.996
wavelet-HLH_glrlm_ShortRunLowGrayLevelEmphasis	0.996
wavelet-HLH_glszm_GrayLevelNonUniformity	0.946
wavelet-HLH_glszm_GrayLevelNonUniformityNormalized	0.777
wavelet-HLH_glszm_GrayLevelVariance	0.777
wavelet-HLH_glszm_HighGrayLevelZoneEmphasis	0.902
wavelet-HLH_glszm_LargeAreaEmphasis	0.981
wavelet-HLH_glszm_LargeAreaHighGrayLevelEmphasis	0.981
wavelet-HLH_glszm_LargeAreaLowGrayLevelEmphasis	0.980
wavelet-HLH_glszm_LowGrayLevelZoneEmphasis	0.902
wavelet-HLH_glszm_SizeZoneNonUniformity	0.912
wavelet-HLH_glszm_SizeZoneNonUniformityNormalized	0.681
wavelet-HLH_glszm_SmallAreaEmphasis	0.715
wavelet-HLH_glszm_SmallAreaHighGrayLevelEmphasis	0.795
wavelet-HLH_glszm_SmallAreaLowGrayLevelEmphasis	0.775
wavelet-HLH_glszm_ZoneEntropy	0.850
wavelet-HLH_glszm_ZonePercentage	0.978
wavelet-HLH_glszm_ZoneVariance	0.988
wavelet-HLH_ngtdm_Busyness	0.999
wavelet-HLH_ngtdm_Coarseness	0.997
wavelet-HLH_ngtdm_Complexity	0.992
wavelet-HLH_ngtdm_Contrast	0.992
wavelet-HLH_ngtdm_Strength	0.997
wavelet-HHL_firstorder_10Percentile	0.999
wavelet-HHL_firstorder_90Percentile	0.999
wavelet-HHL_firstorder_Energy	0.998
wavelet-HHL_firstorder_Entropy	0.952
wavelet-HHL_firstorder_InterquartileRange	1.000
wavelet-HHL_firstorder_Kurtosis	0.933
wavelet-HHL_firstorder_Maximum	0.874
wavelet-HHL_firstorder_MeanAbsoluteDeviation	0.999
wavelet-HHL_firstorder_Mean	0.882
wavelet-HHL_firstorder_Median	0.962
wavelet-HHL_firstorder_Minimum	0.967
wavelet-HHL_firstorder_Range	0.931
wavelet-HHL_firstorder_RobustMeanAbsoluteDeviation	1.000
wavelet-HHL_firstorder_RootMeanSquared	0.995
wavelet-HHL_firstorder_Skewness	0.897
wavelet-HHL_firstorder_TotalEnergy	0.998
wavelet-HHL_firstorder_Uniformity	0.952
wavelet-HHL_firstorder_Variance	0.996
wavelet-HHL_glcm_Autocorrelation	0.967
wavelet-HHL_glcm_ClusterProminence	0.961
wavelet-HHL_glcm_ClusterShade	0.969

wavelet-HHL_glcm_ClusterTendency	0.961
wavelet-HHL_glcm_Contrast	0.964
wavelet-HHL_glcm_Correlation	0.963
wavelet-HHL_glcm_DifferenceAverage	0.964
wavelet-HHL_glcm_DifferenceEntropy	0.998
wavelet-HHL_glcm_DifferenceVariance	0.997
wavelet-HHL_glcm_Id	0.964
wavelet-HHL_glcm_Idm	0.964
wavelet-HHL_glcm_Idmn	0.964
wavelet-HHL_glcm_Idn	0.964
wavelet-HHL_glcm_Imc1	0.998
wavelet-HHL_glcm_Imc2	0.991
wavelet-HHL_glcm_InverseVariance	0.964
wavelet-HHL_glcm_JointAverage	0.970
wavelet-HHL_glcm_JointEnergy	0.997
wavelet-HHL_glcm_JointEntropy	0.998
wavelet-HHL_glcm_MCC	0.992
wavelet-HHL_glcm_MaximumProbability	0.988
wavelet-HHL_glcm_SumAverage	0.970
wavelet-HHL_glcm_SumEntropy	0.991
wavelet-HHL_glcm_SumSquares	0.967
wavelet-HHL_gldm_DependenceEntropy	0.996
wavelet-HHL_gldm_DependenceNonUniformity	0.999
wavelet-HHL_gldm_DependenceNonUniformityNormalized	0.998
wavelet-HHL_gldm_DependenceVariance	0.995
wavelet-HHL_gldm_GrayLevelNonUniformity	0.999
wavelet-HHL_gldm_GrayLevelVariance	0.952
wavelet-HHL_gldm_HighGrayLevelEmphasis	0.958
wavelet-HHL_gldm_LargeDependenceEmphasis	0.997
wavelet-HHL_gldm_LargeDependenceHighGrayLevelEmphasis	0.998
wavelet-HHL_gldm_LargeDependenceLowGrayLevelEmphasis	0.996
wavelet-HHL_gldm_LowGrayLevelEmphasis	0.958
wavelet-HHL_gldm_SmallDependenceEmphasis	0.995
wavelet-HHL_gldm_SmallDependenceHighGrayLevelEmphasis	0.994
wavelet-HHL_gldm_SmallDependenceLowGrayLevelEmphasis	0.993
wavelet-HHL_glrlm_GrayLevelNonUniformity	0.999
wavelet-HHL_glrlm_GrayLevelNonUniformityNormalized	0.808
wavelet-HHL_glrlm_GrayLevelVariance	0.808
wavelet-HHL_glrlm_HighGrayLevelRunEmphasis	0.851
wavelet-HHL_glrlm_LongRunEmphasis	1.000
wavelet-HHL_glrlm_LongRunHighGrayLevelEmphasis	1.000
wavelet-HHL_glrlm_LongRunLowGrayLevelEmphasis	1.000
wavelet-HHL_glrlm_LowGrayLevelRunEmphasis	0.851
wavelet-HHL_glrlm_RunEntropy	0.998
wavelet-HHL_glrlm_RunLengthNonUniformity	0.999
wavelet-HHL_glrlm_RunLengthNonUniformityNormalized	0.997
wavelet-HHL_glrlm_RunPercentage	0.997
wavelet-HHL_glrlm_RunVariance	1.000
wavelet-HHL_glrlm_ShortRunEmphasis	0.998
wavelet-HHL_glrlm_ShortRunHighGrayLevelEmphasis	0.998

wavelet-HHL_glrlm_ShortRunLowGrayLevelEmphasis	0.996
wavelet-HHL_glszm_GrayLevelNonUniformity	0.817
wavelet-HHL_glszm_GrayLevelNonUniformityNormalized	0.679
wavelet-HHL_glszm_GrayLevelVariance	0.679
wavelet-HHL_glszm_HighGrayLevelZoneEmphasis	0.793
wavelet-HHL_glszm_LargeAreaEmphasis	0.948
wavelet-HHL_glszm_LargeAreaHighGrayLevelEmphasis	0.948
wavelet-HHL_glszm_LargeAreaLowGrayLevelEmphasis	0.947
wavelet-HHL_glszm_LowGrayLevelZoneEmphasis	0.793
wavelet-HHL_glszm_SizeZoneNonUniformity	0.846
wavelet-HHL_glszm_SizeZoneNonUniformityNormalized	0.728
wavelet-HHL_glszm_SmallAreaEmphasis	0.755
wavelet-HHL_glszm_SmallAreaHighGrayLevelEmphasis	0.810
wavelet-HHL_glszm_SmallAreaLowGrayLevelEmphasis	0.659
wavelet-HHL_glszm_ZoneEntropy	0.753
wavelet-HHL_glszm_ZonePercentage	0.926
wavelet-HHL_glszm_ZoneVariance	0.982
wavelet-HHL_ngtdm_Busyness	0.999
wavelet-HHL_ngtdm_Coarseness	0.997
wavelet-HHL_ngtdm_Complexity	0.969
wavelet-HHL_ngtdm_Contrast	0.970
wavelet-HHL_ngtdm_Strength	0.997
wavelet-HHH_firstorder_10Percentile	0.999
wavelet-HHH_firstorder_90Percentile	0.999
wavelet-HHH_firstorder_Energy	0.978
wavelet-HHH_firstorder_Entropy	0.776
wavelet-HHH_firstorder_InterquartileRange	0.999
wavelet-HHH_firstorder_Kurtosis	0.934
wavelet-HHH_firstorder_Maximum	0.974
wavelet-HHH_firstorder_MeanAbsoluteDeviation	0.998
wavelet-HHH_firstorder_Mean	0.926
wavelet-HHH_firstorder_Median	0.955
wavelet-HHH_firstorder_Minimum	0.943
wavelet-HHH_firstorder_Range	0.965
wavelet-HHH_firstorder_RobustMeanAbsoluteDeviation	0.999
wavelet-HHH_firstorder_RootMeanSquared	0.992
wavelet-HHH_firstorder_Skewness	0.767
wavelet-HHH_firstorder_TotalEnergy	0.978
wavelet-HHH_firstorder_Uniformity	0.776
wavelet-HHH_firstorder_Variance	0.985
wavelet-HHH_glcm_Autocorrelation	0.956
wavelet-HHH_glcm_ClusterProminence	0.990
wavelet-HHH_glcm_ClusterShade	0.955
wavelet-HHH_glcm_ClusterTendency	0.990
wavelet-HHH_glcm_Contrast	0.990
wavelet-HHH_glcm_Correlation	0.990
wavelet-HHH_glcm_DifferenceAverage	0.990
wavelet-HHH_glcm_DifferenceEntropy	1.000
wavelet-HHH_glcm_DifferenceVariance	0.999
wavelet-HHH_glcm_Id	0.990

wavelet-HHH_glcm_Idm	0.990
wavelet-HHH_glcm_Idmn	0.990
wavelet-HHH_glcm_Idn	0.990
wavelet-HHH_glcm_Imc1	1.000
wavelet-HHH_glcm_Imc2	0.999
wavelet-HHH_glcm_InverseVariance	0.990
wavelet-HHH_glcm_JointAverage	0.960
wavelet-HHH_glcm_JointEnergy	0.999
wavelet-HHH_glcm_JointEntropy	0.999
wavelet-HHH_glcm_MCC	0.999
wavelet-HHH_glcm_MaximumProbability	0.996
wavelet-HHH_glcm_SumAverage	0.960
wavelet-HHH_glcm_SumEntropy	0.998
wavelet-HHH_glcm_SumSquares	0.865
wavelet-HHH_gldm_DependenceEntropy	0.997
wavelet-HHH_gldm_DependenceNonUniformity	0.999
wavelet-HHH_gldm_DependenceNonUniformityNormalized	0.998
wavelet-HHH_gldm_DependenceVariance	0.996
wavelet-HHH_gldm_GrayLevelNonUniformity	0.999
wavelet-HHH_gldm_GrayLevelVariance	0.776
wavelet-HHH_gldm_HighGrayLevelEmphasis	0.933
wavelet-HHH_gldm_LargeDependenceEmphasis	0.998
wavelet-HHH_gldm_LargeDependenceHighGrayLevelEmphasis	0.997
wavelet-HHH_gldm_LargeDependenceLowGrayLevelEmphasis	0.998
wavelet-HHH_gldm_LowGrayLevelEmphasis	0.933
wavelet-HHH_gldm_SmallDependenceEmphasis	0.996
wavelet-HHH_gldm_SmallDependenceHighGrayLevelEmphasis	0.996
wavelet-HHH_gldm_SmallDependenceLowGrayLevelEmphasis	0.995
wavelet-HHH_glrlm_GrayLevelNonUniformity	0.999
wavelet-HHH_glrlm_GrayLevelNonUniformityNormalized	0.970
wavelet-HHH_glrlm_GrayLevelVariance	0.970
wavelet-HHH_glrlm_HighGrayLevelRunEmphasis	0.879
wavelet-HHH_glrlm_LongRunEmphasis	1.000
wavelet-HHH_glrlm_LongRunHighGrayLevelEmphasis	0.999
wavelet-HHH_glrlm_LongRunLowGrayLevelEmphasis	1.000
wavelet-HHH_glrlm_LowGrayLevelRunEmphasis	0.879
wavelet-HHH_glrlm_RunEntropy	0.998
wavelet-HHH_glrlm_RunLengthNonUniformity	0.999
wavelet-HHH_glrlm_RunLengthNonUniformityNormalized	0.998
wavelet-HHH_glrlm_RunPercentage	0.998
wavelet-HHH_glrlm_RunVariance	0.998
wavelet-HHH_glrlm_ShortRunEmphasis	0.999
wavelet-HHH_glrlm_ShortRunHighGrayLevelEmphasis	0.998
wavelet-HHH_glrlm_ShortRunLowGrayLevelEmphasis	0.998
wavelet-HHH_glszm_GrayLevelNonUniformity	0.914
wavelet-HHH_glszm_GrayLevelNonUniformityNormalized	0.512
wavelet-HHH_glszm_GrayLevelVariance	0.512
wavelet-HHH_glszm_HighGrayLevelZoneEmphasis	0.646
wavelet-HHH_glszm_LargeAreaEmphasis	0.972
wavelet-HHH_glszm_LargeAreaHighGrayLevelEmphasis	0.972

wavelet-HHH_glszm_LargeAreaLowGrayLevelEmphasis	0.972
wavelet-HHH_glszm_LowGrayLevelZoneEmphasis	0.646
wavelet-HHH_glszm_SizeZoneNonUniformity	0.954
wavelet-HHH_glszm_SizeZoneNonUniformityNormalized	0.785
wavelet-HHH_glszm_SmallAreaEmphasis	0.733
wavelet-HHH_glszm_SmallAreaHighGrayLevelEmphasis	0.668
wavelet-HHH_glszm_SmallAreaLowGrayLevelEmphasis	0.767
wavelet-HHH_glszm_ZoneEntropy	0.862
wavelet-HHH_glszm_ZonePercentage	0.986
wavelet-HHH_glszm_ZoneVariance	0.981
wavelet-HHH_ngtdm_Busyness	0.999
wavelet-HHH_ngtdm_Coarseness	0.997
wavelet-HHH_ngtdm_Complexity	0.990
wavelet-HHH_ngtdm_Contrast	0.990
wavelet-HHH_ngtdm_Strength	0.997
wavelet-LLL_firstorder_10Percentile	0.999
wavelet-LLL_firstorder_90Percentile	1.000
wavelet-LLL_firstorder_Energy	0.999
wavelet-LLL_firstorder_Entropy	0.992
wavelet-LLL_firstorder_InterquartileRange	0.978
wavelet-LLL_firstorder_Kurtosis	0.663
wavelet-LLL_firstorder_Maximum	0.993
wavelet-LLL_firstorder_MeanAbsoluteDeviation	0.930
wavelet-LLL_firstorder_Mean	1.000
wavelet-LLL_firstorder_Median	1.000
wavelet-LLL_firstorder_Minimum	0.893
wavelet-LLL_firstorder_Range	0.965
wavelet-LLL_firstorder_RobustMeanAbsoluteDeviation	0.975
wavelet-LLL_firstorder_RootMeanSquared	1.000
wavelet-LLL_firstorder_Skewness	0.769
wavelet-LLL_firstorder_TotalEnergy	0.999
wavelet-LLL_firstorder_Uniformity	0.995
wavelet-LLL_firstorder_Variance	0.872
wavelet-LLL_glcm_Autocorrelation	0.591
wavelet-LLL_glcm_ClusterProminence	1.000
wavelet-LLL_glcm_ClusterShade	0.995
wavelet-LLL_glcm_ClusterTendency	1.000
wavelet-LLL_glcm_Contrast	0.993
wavelet-LLL_glcm_Correlation	0.425
wavelet-LLL_glcm_DifferenceAverage	0.993
wavelet-LLL_glcm_DifferenceEntropy	0.993
wavelet-LLL_glcm_DifferenceVariance	0.993
wavelet-LLL_glcm_Id	0.993
wavelet-LLL_glcm_Idm	0.993
wavelet-LLL_glcm_Idmn	0.992
wavelet-LLL_glcm_Idn	0.993
wavelet-LLL_glcm_Imc1	0.994
wavelet-LLL_glcm_Imc2	1.000
wavelet-LLL_glcm_InverseVariance	0.993
wavelet-LLL_glcm_JointAverage	0.590

wavelet-LLL_glcm_JointEnergy	0.998
wavelet-LLL_glcm_JointEntropy	0.997
wavelet-LLL_glcm_MCC	0.350
wavelet-LLL_glcm_MaximumProbability	0.998
wavelet-LLL_glcm_SumAverage	0.590
wavelet-LLL_glcm_SumEntropy	0.997
wavelet-LLL_glcm_SumSquares	0.999
wavelet-LLL_gldm_DependenceEntropy	0.998
wavelet-LLL_gldm_DependenceNonUniformity	0.999
wavelet-LLL_gldm_DependenceNonUniformityNormalized	0.998
wavelet-LLL_gldm_DependenceVariance	0.998
wavelet-LLL_gldm_GrayLevelNonUniformity	0.999
wavelet-LLL_gldm_GrayLevelVariance	0.995
wavelet-LLL_gldm_HighGrayLevelEmphasis	0.591
wavelet-LLL_gldm_LargeDependenceEmphasis	0.998
wavelet-LLL_gldm_LargeDependenceHighGrayLevelEmphasis	0.626
wavelet-LLL_gldm_LargeDependenceLowGrayLevelEmphasis	0.599
wavelet-LLL_gldm_LowGrayLevelEmphasis	0.589
wavelet-LLL_gldm_SmallDependenceEmphasis	0.994
wavelet-LLL_gldm_SmallDependenceHighGrayLevelEmphasis	0.563
wavelet-LLL_gldm_SmallDependenceLowGrayLevelEmphasis	0.904
wavelet-LLL_glrlm_GrayLevelNonUniformity	0.999
wavelet-LLL_glrlm_GrayLevelNonUniformityNormalized	0.967
wavelet-LLL_glrlm_GrayLevelVariance	0.966
wavelet-LLL_glrlm_HighGrayLevelRunEmphasis	0.596
wavelet-LLL_glrlm_LongRunEmphasis	0.999
wavelet-LLL_glrlm_LongRunHighGrayLevelEmphasis	0.702
wavelet-LLL_glrlm_LongRunLowGrayLevelEmphasis	0.870
wavelet-LLL_glrlm_LowGrayLevelRunEmphasis	0.586
wavelet-LLL_glrlm_RunEntropy	0.998
wavelet-LLL_glrlm_RunLengthNonUniformity	0.998
wavelet-LLL_glrlm_RunLengthNonUniformityNormalized	0.998
wavelet-LLL_glrlm_RunPercentage	0.998
wavelet-LLL_glrlm_RunVariance	0.999
wavelet-LLL_glrlm_ShortRunEmphasis	0.990
wavelet-LLL_glrlm_ShortRunHighGrayLevelEmphasis	0.624
wavelet-LLL_glrlm_ShortRunLowGrayLevelEmphasis	0.936
wavelet-LLL_glszm_GrayLevelNonUniformity	0.625
wavelet-LLL_glszm_GrayLevelNonUniformityNormalized	0.452
wavelet-LLL_glszm_GrayLevelVariance	0.506
wavelet-LLL_glszm_HighGrayLevelZoneEmphasis	0.303
wavelet-LLL_glszm_LargeAreaEmphasis	0.926
wavelet-LLL_glszm_LargeAreaHighGrayLevelEmphasis	0.794
wavelet-LLL_glszm_LargeAreaLowGrayLevelEmphasis	0.699
wavelet-LLL_glszm_LowGrayLevelZoneEmphasis	0.373
wavelet-LLL_glszm_SizeZoneNonUniformity	0.320
wavelet-LLL_glszm_SizeZoneNonUniformityNormalized	0.660
wavelet-LLL_glszm_SmallAreaEmphasis	0.122
wavelet-LLL_glszm_SmallAreaHighGrayLevelEmphasis	0.129
wavelet-LLL_glszm_SmallAreaLowGrayLevelEmphasis	0.118

wavelet-LLL_glszm_ZoneEntropy	0.802
wavelet-LLL_glszm_ZonePercentage	0.994
wavelet-LLL_glszm_ZoneVariance	0.046
wavelet-LLL_ngtdm_Busyness	0.986
wavelet-LLL_ngtdm_Coarseness	0.653
wavelet-LLL_ngtdm_Complexity	0.990
wavelet-LLL_ngtdm_Contrast	0.973
wavelet-LLL_ngtdm_Strength	0.047
original_shape_Elongation_1	0.684
original_shape_Flatness_1	0.884
original_shape_LeastAxisLength_1	0.961
original_shape_MajorAxisLength_1	0.939
original_shape_Maximum2DDiameterColumn_1	0.972
original_shape_Maximum2DDiameterRow_1	0.989
original_shape_Maximum2DDiameterSlice_1	0.987
original_shape_Maximum3DDiameter_1	0.995
original_shape_MeshVolume_1	0.517
original_shape_MinorAxisLength_1	0.960
original_shape_Sphericity_1	0.326
original_shape_SurfaceArea_1	0.715
original_shape_SurfaceVolumeRatio_1	0.039
original_shape_VoxelVolume_1	0.547
original_firstorder_10Percentile_1	0.908
original_firstorder_90Percentile_1	0.988
original_firstorder_Energy_1	0.605
original_firstorder_Entropy_1	0.000
original_firstorder_InterquartileRange_1	0.094
original_firstorder_Kurtosis_1	0.643
original_firstorder_Maximum_1	0.939
original_firstorder_MeanAbsoluteDeviation_1	0.198
original_firstorder_Mean_1	0.964
original_firstorder_Median_1	0.971
original_firstorder_Minimum_1	0.669
original_firstorder_Range_1	0.883
original_firstorder_RobustMeanAbsoluteDeviation_1	0.095
original_firstorder_RootMeanSquared_1	0.967
original_firstorder_Skewness_1	0.490
original_firstorder_TotalEnergy_1	0.605
original_firstorder_Variance_1	0.161
original_glcm_DifferenceEntropy_1	0.000
original_glcm_JointEntropy_1	0.000
original_glcm_SumEntropy_1	0.000
original_gldm_DependenceEntropy_1	0.308
original_gldm_DependenceNonUniformity_1	0.078
original_gldm_DependenceNonUniformityNormalized_1	0.113
original_gldm_DependenceVariance_1	0.308
original_gldm_GrayLevelNonUniformity_1	0.547
original_gldm_LargeDependenceEmphasis_1	0.033
original_gldm_LargeDependenceHighGrayLevelEmphasis_1	0.070
original_gldm_LargeDependenceLowGrayLevelEmphasis_1	0.022

original_gldm_SmallDependenceEmphasis_1	0.081
original_gldm_SmallDependenceHighGrayLevelEmphasis_1	0.024
original_gldm_SmallDependenceLowGrayLevelEmphasis_1	0.093
original_glrlm_GrayLevelNonUniformity_1	0.727
original_glrlm_LongRunEmphasis_1	0.010
original_glrlm_LongRunHighGrayLevelEmphasis_1	0.010
original_glrlm_LongRunLowGrayLevelEmphasis_1	0.010
original_glrlm_RunEntropy_1	0.057
original_glrlm_RunLengthNonUniformity_1	0.661
original_glrlm_RunLengthNonUniformityNormalized_1	0.080
original_glrlm_RunPercentage_1	0.005
original_glrlm_RunVariance_1	0.053
original_glrlm_ShortRunEmphasis_1	0.136
original_glrlm_ShortRunHighGrayLevelEmphasis_1	0.080
original_glrlm_ShortRunLowGrayLevelEmphasis_1	0.000
original_glszm_GrayLevelNonUniformity_1	0.218
original_glszm_LargeAreaEmphasis_1	0.000
original_glszm_LargeAreaHighGrayLevelEmphasis_1	0.000
original_glszm_LargeAreaLowGrayLevelEmphasis_1	0.000
original_glszm_SizeZoneNonUniformity_1	0.271
original_glszm_SizeZoneNonUniformityNormalized_1	0.123
original_glszm_SmallAreaEmphasis_1	0.710
original_glszm_SmallAreaHighGrayLevelEmphasis_1	0.708
original_glszm_SmallAreaLowGrayLevelEmphasis_1	0.710
original_glszm_ZoneEntropy_1	0.165
original_glszm_ZonePercentage_1	0.195
original_glszm_ZoneVariance_1	0.063
exponential_firstorder_10Percentile_1	0.954
exponential_firstorder_90Percentile_1	0.983
exponential_firstorder_Energy_1	0.576
exponential_firstorder_Entropy_1	0.000
exponential_firstorder_InterquartileRange_1	0.379
exponential_firstorder_Kurtosis_1	0.590
exponential_firstorder_Maximum_1	0.961
exponential_firstorder_MeanAbsoluteDeviation_1	0.440
exponential_firstorder_Mean_1	0.974
exponential_firstorder_Median_1	0.977
exponential_firstorder_Minimum_1	0.808
exponential_firstorder_Range_1	0.927
exponential_firstorder_RobustMeanAbsoluteDeviation_1	0.367
exponential_firstorder_RootMeanSquared_1	0.974
exponential_firstorder_Skewness_1	0.445
exponential_firstorder_TotalEnergy_1	0.576
exponential_firstorder_Variance_1	0.321
exponential_glcm_DifferenceEntropy_1	0.000
exponential_glcm_JointEntropy_1	0.000
exponential_glcm_SumEntropy_1	0.000
exponential_gldm_DependenceEntropy_1	0.308
exponential_gldm_DependenceNonUniformity_1	0.078
exponential_gldm_DependenceNonUniformityNormalized_1	0.113

exponential_gldm_DependenceVariance_1	0.308
exponential_gldm_GrayLevelNonUniformity_1	0.547
exponential_gldm_LargeDependenceEmphasis_1	0.033
exponential_gldm_LargeDependenceHighGrayLevelEmphasis_1	0.033
exponential_gldm_LargeDependenceLowGrayLevelEmphasis_1	0.033
exponential_gldm_SmallDependenceEmphasis_1	0.081
exponential_gldm_SmallDependenceHighGrayLevelEmphasis_1	0.081
exponential_gldm_SmallDependenceLowGrayLevelEmphasis_1	0.081
exponential_glrlm_GrayLevelNonUniformity_1	0.727
exponential_glrlm_LongRunEmphasis_1	0.010
exponential_glrlm_LongRunHighGrayLevelEmphasis_1	0.010
exponential_glrlm_LongRunLowGrayLevelEmphasis_1	0.010
exponential_glrlm_RunEntropy_1	0.057
exponential_glrlm_RunLengthNonUniformity_1	0.661
exponential_glrlm_RunLengthNonUniformityNormalized_1	0.080
exponential_glrlm_RunPercentage_1	0.005
exponential_glrlm_RunVariance_1	0.053
exponential_glrlm_ShortRunEmphasis_1	0.136
exponential_glrlm_ShortRunHighGrayLevelEmphasis_1	0.136
exponential_glrlm_ShortRunLowGrayLevelEmphasis_1	0.136
exponential_glszm_GrayLevelNonUniformity_1	0.217
exponential_glszm_LargeAreaEmphasis_1	0.007
exponential_glszm_LargeAreaHighGrayLevelEmphasis_1	0.007
exponential_glszm_LargeAreaLowGrayLevelEmphasis_1	0.007
exponential_glszm_SizeZoneNonUniformity_1	0.271
exponential_glszm_SizeZoneNonUniformityNormalized_1	0.123
exponential_glszm_SmallAreaEmphasis_1	0.710
exponential_glszm_SmallAreaHighGrayLevelEmphasis_1	0.710
exponential_glszm_SmallAreaLowGrayLevelEmphasis_1	0.710
exponential_glszm_ZoneEntropy_1	0.166
exponential_glszm_ZonePercentage_1	0.195
exponential_glszm_ZoneVariance_1	0.063
gradient_firstorder_10Percentile_1	0.005
gradient_firstorder_90Percentile_1	0.129
gradient_firstorder_Energy_1	0.916
gradient_firstorder_Entropy_1	0.000
gradient_firstorder_InterquartileRange_1	0.152
gradient_firstorder_Kurtosis_1	0.375
gradient_firstorder_Maximum_1	0.823
gradient_firstorder_MeanAbsoluteDeviation_1	0.249
gradient_firstorder_Mean_1	0.048
gradient_firstorder_Median_1	0.020
gradient_firstorder_Minimum_1	0.015
gradient_firstorder_Range_1	0.835
gradient_firstorder_RobustMeanAbsoluteDeviation_1	0.167
gradient_firstorder_RootMeanSquared_1	0.120
gradient_firstorder_Skewness_1	0.530
gradient_firstorder_TotalEnergy_1	0.916
gradient_firstorder_Variance_1	0.234
gradient_glcm_DifferenceEntropy_1	0.000

gradient_glcm_JointEntropy_1	0.000
gradient_glcm_SumEntropy_1	0.000
gradient_gldm_DependenceEntropy_1	0.308
gradient_gldm_DependenceNonUniformity_1	0.078
gradient_gldm_DependenceNonUniformityNormalized_1	0.113
gradient_gldm_DependenceVariance_1	0.308
gradient_gldm_GrayLevelNonUniformity_1	0.547
gradient_gldm_LargeDependenceEmphasis_1	0.033
gradient_gldm_LargeDependenceHighGrayLevelEmphasis_1	0.033
gradient_gldm_LargeDependenceLowGrayLevelEmphasis_1	0.033
gradient_gldm_SmallDependenceEmphasis_1	0.081
gradient_gldm_SmallDependenceHighGrayLevelEmphasis_1	0.081
gradient_gldm_SmallDependenceLowGrayLevelEmphasis_1	0.081
gradient_glrlm_GrayLevelNonUniformity_1	0.727
gradient_glrlm_LongRunEmphasis_1	0.010
gradient_glrlm_LongRunHighGrayLevelEmphasis_1	0.010
gradient_glrlm_LongRunLowGrayLevelEmphasis_1	0.010
gradient_glrlm_RunEntropy_1	0.057
gradient_glrlm_RunLengthNonUniformity_1	0.661
gradient_glrlm_RunLengthNonUniformityNormalized_1	0.080
gradient_glrlm_RunPercentage_1	0.005
gradient_glrlm_RunVariance_1	0.053
gradient_glrlm_ShortRunEmphasis_1	0.136
gradient_glrlm_ShortRunHighGrayLevelEmphasis_1	0.136
gradient_glrlm_ShortRunLowGrayLevelEmphasis_1	0.136
gradient_glszm_GrayLevelNonUniformity_1	0.217
gradient_glszm_LargeAreaEmphasis_1	0.007
gradient_glszm_LargeAreaHighGrayLevelEmphasis_1	0.007
gradient_glszm_LargeAreaLowGrayLevelEmphasis_1	0.007
gradient_glszm_SizeZoneNonUniformity_1	0.271
gradient_glszm_SizeZoneNonUniformityNormalized_1	0.123
gradient_glszm_SmallAreaEmphasis_1	0.710
gradient_glszm_SmallAreaHighGrayLevelEmphasis_1	0.710
gradient_glszm_SmallAreaLowGrayLevelEmphasis_1	0.710
gradient_glszm_ZoneEntropy_1	0.166
gradient_glszm_ZonePercentage_1	0.195
gradient_glszm_ZoneVariance_1	0.063
lbp-2D_firstorder_10Percentile_1	0.070
lbp-2D_firstorder_90Percentile_1	0.166
lbp-2D_firstorder_Energy_1	0.581
lbp-2D_firstorder_Entropy_1	0.316
lbp-2D_firstorder_InterquartileRange_1	0.126
lbp-2D_firstorder_Kurtosis_1	0.185
lbp-2D_firstorder_MeanAbsoluteDeviation_1	0.145
lbp-2D_firstorder_Mean_1	0.362
lbp-2D_firstorder_Median_1	0.298
lbp-2D_firstorder_Minimum_1	0.010
lbp-2D_firstorder_Range_1	0.010
lbp-2D_firstorder_RobustMeanAbsoluteDeviation_1	0.106
lbp-2D_firstorder_RootMeanSquared_1	0.387

lbp-2D_firstorder_Skewness_1	0.229
lbp-2D_firstorder_TotalEnergy_1	0.581
lbp-2D_firstorder_Uniformity_1	0.312
lbp-2D_firstorder_Variance_1	0.136
lbp-2D_glcm_Autocorrelation_1	0.331
lbp-2D_glcm_ClusterProminence_1	0.147
lbp-2D_glcm_ClusterShade_1	0.312
lbp-2D_glcm_ClusterTendency_1	0.208
lbp-2D_glcm_Contrast_1	0.152
lbp-2D_glcm_Correlation_1	0.059
lbp-2D_glcm_DifferenceAverage_1	0.152
lbp-2D_glcm_DifferenceEntropy_1	0.146
lbp-2D_glcm_DifferenceVariance_1	0.167
lbp-2D_glcm_Id_1	0.152
lbp-2D_glcm_Idm_1	0.152
lbp-2D_glcm_Idmn_1	0.152
lbp-2D_glcm_Idn_1	0.152
lbp-2D_glcm_Imc1_1	0.243
lbp-2D_glcm_Imc2_1	0.143
lbp-2D_glcm_InverseVariance_1	0.152
lbp-2D_glcm_JointAverage_1	0.313
lbp-2D_glcm_JointEnergy_1	0.228
lbp-2D_glcm_JointEntropy_1	0.223
lbp-2D_glcm_MCC_1	0.153
lbp-2D_glcm_MaximumProbability_1	0.248
lbp-2D_glcm_SumAverage_1	0.313
lbp-2D_glcm_SumEntropy_1	0.226
lbp-2D_glcm_SumSquares_1	0.268
lbp-2D_gldm_DependenceEntropy_1	0.414
lbp-2D_gldm_DependenceNonUniformity_1	0.480
lbp-2D_gldm_DependenceNonUniformityNormalized_1	0.335
lbp-2D_gldm_DependenceVariance_1	0.505
lbp-2D_gldm_GrayLevelNonUniformity_1	0.567
lbp-2D_gldm_GrayLevelVariance_1	0.312
lbp-2D_gldm_HighGrayLevelEmphasis_1	0.388
lbp-2D_gldm_LargeDependenceEmphasis_1	0.164
lbp-2D_gldm_LargeDependenceHighGrayLevelEmphasis_1	0.182
lbp-2D_gldm_LargeDependenceLowGrayLevelEmphasis_1	0.226
lbp-2D_gldm_LowGrayLevelEmphasis_1	0.388
lbp-2D_gldm_SmallDependenceEmphasis_1	0.039
lbp-2D_gldm_SmallDependenceHighGrayLevelEmphasis_1	0.032
lbp-2D_gldm_SmallDependenceLowGrayLevelEmphasis_1	0.105
lbp-2D_glrlm_GrayLevelNonUniformity_1	0.625
lbp-2D_glrlm_GrayLevelNonUniformityNormalized_1	0.327
lbp-2D_glrlm_GrayLevelVariance_1	0.327
lbp-2D_glrlm_HighGrayLevelRunEmphasis_1	0.414
lbp-2D_glrlm_LongRunEmphasis_1	0.569
lbp-2D_glrlm_LongRunHighGrayLevelEmphasis_1	0.587
lbp-2D_glrlm_LongRunLowGrayLevelEmphasis_1	0.516
lbp-2D_glrlm_LowGrayLevelRunEmphasis_1	0.414

lbp-2D_glrlm_RunEntropy_1	0.124
lbp-2D_glrlm_RunLengthNonUniformity_1	0.704
lbp-2D_glrlm_RunLengthNonUniformityNormalized_1	0.029
lbp-2D_glrlm_RunPercentage_1	0.098
lbp-2D_glrlm_RunVariance_1	0.551
lbp-2D_glrlm_ShortRunEmphasis_1	0.088
lbp-2D_glrlm_ShortRunHighGrayLevelEmphasis_1	0.273
lbp-2D_glrlm_ShortRunLowGrayLevelEmphasis_1	0.302
lbp-2D_glszm_GrayLevelNonUniformity_1	0.646
lbp-2D_glszm_GrayLevelNonUniformityNormalized_1	0.389
lbp-2D_glszm_GrayLevelVariance_1	0.389
lbp-2D_glszm_HighGrayLevelZoneEmphasis_1	0.174
lbp-2D_glszm_LargeAreaEmphasis_1	0.004
lbp-2D_glszm_LargeAreaHighGrayLevelEmphasis_1	0.001
lbp-2D_glszm_LargeAreaLowGrayLevelEmphasis_1	0.007
lbp-2D_glszm_LowGrayLevelZoneEmphasis_1	0.174
lbp-2D_glszm_SizeZoneNonUniformity_1	0.625
lbp-2D_glszm_SizeZoneNonUniformityNormalized_1	0.063
lbp-2D_glszm_SmallAreaEmphasis_1	0.095
lbp-2D_glszm_SmallAreaHighGrayLevelEmphasis_1	0.017
lbp-2D_glszm_SmallAreaLowGrayLevelEmphasis_1	0.386
lbp-2D_glszm_ZoneEntropy_1	0.202
lbp-2D_glszm_ZonePercentage_1	0.057
lbp-2D_glszm_ZoneVariance_1	0.003
lbp-2D_ngtdm_Busyness_1	0.423
lbp-2D_ngtdm_Coarseness_1	0.313
lbp-2D_ngtdm_Complexity_1	0.069
lbp-2D_ngtdm_Contrast_1	0.125
lbp-2D_ngtdm_Strength_1	0.314
lbp-3D-m1_firstorder_10Percentile_1	0.112
lbp-3D-m1_firstorder_90Percentile_1	0.498
lbp-3D-m1_firstorder_Energy_1	0.632
lbp-3D-m1_firstorder_Entropy_1	0.244
lbp-3D-m1_firstorder_InterquartileRange_1	0.244
lbp-3D-m1_firstorder_Kurtosis_1	0.182
lbp-3D-m1_firstorder_Maximum_1	0.269
lbp-3D-m1_firstorder_MeanAbsoluteDeviation_1	0.237
lbp-3D-m1_firstorder_Mean_1	0.301
lbp-3D-m1_firstorder_Median_1	0.335
lbp-3D-m1_firstorder_Minimum_1	0.013
lbp-3D-m1_firstorder_Range_1	0.185
lbp-3D-m1_firstorder_RobustMeanAbsoluteDeviation_1	0.234
lbp-3D-m1_firstorder_RootMeanSquared_1	0.365
lbp-3D-m1_firstorder_Skewness_1	0.324
lbp-3D-m1_firstorder_TotalEnergy_1	0.632
lbp-3D-m1_firstorder_Uniformity_1	0.156
lbp-3D-m1_firstorder_Variance_1	0.264
lbp-3D-m1_glcm_Autocorrelation_1	0.248
lbp-3D-m1_glcm_ClusterProminence_1	0.350
lbp-3D-m1_glcm_ClusterShade_1	0.368

lbp-3D-m1_glcm_ClusterTendency_1	0.279
lbp-3D-m1_glcm_Contrast_1	0.132
lbp-3D-m1_glcm_Correlation_1	0.113
lbp-3D-m1_glcm_DifferenceAverage_1	0.052
lbp-3D-m1_glcm_DifferenceEntropy_1	0.112
lbp-3D-m1_glcm_DifferenceVariance_1	0.207
lbp-3D-m1_glcm_Id_1	0.001
lbp-3D-m1_glcm_Idm_1	0.009
lbp-3D-m1_glcm_Idmn_1	0.041
lbp-3D-m1_glcm_Idn_1	0.020
lbp-3D-m1_glcm_Imc1_1	0.235
lbp-3D-m1_glcm_Imc2_1	0.385
lbp-3D-m1_glcm_InverseVariance_1	0.200
lbp-3D-m1_glcm_JointAverage_1	0.239
lbp-3D-m1_glcm_JointEnergy_1	0.063
lbp-3D-m1_glcm_JointEntropy_1	0.151
lbp-3D-m1_glcm_MCC_1	0.338
lbp-3D-m1_glcm_MaximumProbability_1	0.009
lbp-3D-m1_glcm_SumAverage_1	0.239
lbp-3D-m1_glcm_SumEntropy_1	0.208
lbp-3D-m1_glcm_SumSquares_1	0.197
lbp-3D-m1_gldm_DependenceEntropy_1	0.416
lbp-3D-m1_gldm_DependenceNonUniformity_1	0.534
lbp-3D-m1_gldm_DependenceNonUniformityNormalized_1	0.562
lbp-3D-m1_gldm_DependenceVariance_1	0.509
lbp-3D-m1_gldm_GrayLevelNonUniformity_1	0.592
lbp-3D-m1_gldm_GrayLevelVariance_1	0.306
lbp-3D-m1_gldm_HighGrayLevelEmphasis_1	0.328
lbp-3D-m1_gldm_LargeDependenceEmphasis_1	0.598
lbp-3D-m1_gldm_LargeDependenceHighGrayLevelEmphasis_1	0.553
lbp-3D-m1_gldm_LargeDependenceLowGrayLevelEmphasis_1	0.223
lbp-3D-m1_gldm_LowGrayLevelEmphasis_1	0.214
lbp-3D-m1_gldm_SmallDependenceEmphasis_1	0.298
lbp-3D-m1_gldm_SmallDependenceHighGrayLevelEmphasis_1	0.371
lbp-3D-m1_gldm_SmallDependenceLowGrayLevelEmphasis_1	0.390
lbp-3D-m1_glrlm_GrayLevelNonUniformity_1	0.597
lbp-3D-m1_glrlm_GrayLevelNonUniformityNormalized_1	0.176
lbp-3D-m1_glrlm_GrayLevelVariance_1	0.313
lbp-3D-m1_glrlm_HighGrayLevelRunEmphasis_1	0.346
lbp-3D-m1_glrlm_LongRunEmphasis_1	0.849
lbp-3D-m1_glrlm_LongRunHighGrayLevelEmphasis_1	0.678
lbp-3D-m1_glrlm_LongRunLowGrayLevelEmphasis_1	0.349
lbp-3D-m1_glrlm_LowGrayLevelRunEmphasis_1	0.209
lbp-3D-m1_glrlm_RunEntropy_1	0.249
lbp-3D-m1_glrlm_RunLengthNonUniformity_1	0.588
lbp-3D-m1_glrlm_RunLengthNonUniformityNormalized_1	0.395
lbp-3D-m1_glrlm_RunPercentage_1	0.508
lbp-3D-m1_glrlm_RunVariance_1	0.807
lbp-3D-m1_glrlm_ShortRunEmphasis_1	0.542
lbp-3D-m1_glrlm_ShortRunHighGrayLevelEmphasis_1	0.343

lbp-3D-m1_glrlm_ShortRunLowGrayLevelEmphasis_1	0.221
lbp-3D-m1_glszm_GrayLevelNonUniformity_1	0.733
lbp-3D-m1_glszm_GrayLevelNonUniformityNormalized_1	0.310
lbp-3D-m1_glszm_GrayLevelVariance_1	0.125
lbp-3D-m1_glszm_HighGrayLevelZoneEmphasis_1	0.266
lbp-3D-m1_glszm_LargeAreaEmphasis_1	0.084
lbp-3D-m1_glszm_LargeAreaHighGrayLevelEmphasis_1	0.119
lbp-3D-m1_glszm_LargeAreaLowGrayLevelEmphasis_1	0.034
lbp-3D-m1_glszm_LowGrayLevelZoneEmphasis_1	0.466
lbp-3D-m1_glszm_SizeZoneNonUniformity_1	0.685
lbp-3D-m1_glszm_SizeZoneNonUniformityNormalized_1	0.765
lbp-3D-m1_glszm_SmallAreaEmphasis_1	0.805
lbp-3D-m1_glszm_SmallAreaHighGrayLevelEmphasis_1	0.277
lbp-3D-m1_glszm_SmallAreaLowGrayLevelEmphasis_1	0.533
lbp-3D-m1_glszm_ZoneEntropy_1	0.774
lbp-3D-m1_glszm_ZonePercentage_1	0.137
lbp-3D-m1_glszm_ZoneVariance_1	0.084
lbp-3D-m1_ngtdm_Busyness_1	0.393
lbp-3D-m1_ngtdm_Coarseness_1	0.127
lbp-3D-m1_ngtdm_Complexity_1	0.018
lbp-3D-m1_ngtdm_Contrast_1	0.153
lbp-3D-m1_ngtdm_Strength_1	0.479
lbp-3D-m2_firstorder_10Percentile_1	0.120
lbp-3D-m2_firstorder_90Percentile_1	0.427
lbp-3D-m2_firstorder_Energy_1	0.615
lbp-3D-m2_firstorder_Entropy_1	0.030
lbp-3D-m2_firstorder_InterquartileRange_1	0.020
lbp-3D-m2_firstorder_Kurtosis_1	0.086
lbp-3D-m2_firstorder_Maximum_1	0.206
lbp-3D-m2_firstorder_MeanAbsoluteDeviation_1	0.036
lbp-3D-m2_firstorder_Mean_1	0.297
lbp-3D-m2_firstorder_Median_1	0.392
lbp-3D-m2_firstorder_Minimum_1	0.004
lbp-3D-m2_firstorder_Range_1	0.041
lbp-3D-m2_firstorder_RobustMeanAbsoluteDeviation_1	0.036
lbp-3D-m2_firstorder_RootMeanSquared_1	0.341
lbp-3D-m2_firstorder_Skewness_1	0.455
lbp-3D-m2_firstorder_TotalEnergy_1	0.615
lbp-3D-m2_firstorder_Uniformity_1	0.042
lbp-3D-m2_firstorder_Variance_1	0.027
lbp-3D-m2_glcm_Autocorrelation_1	0.235
lbp-3D-m2_glcm_ClusterProminence_1	0.069
lbp-3D-m2_glcm_ClusterShade_1	0.421
lbp-3D-m2_glcm_ClusterTendency_1	0.034
lbp-3D-m2_glcm_Contrast_1	0.108
lbp-3D-m2_glcm_Correlation_1	0.145
lbp-3D-m2_glcm_DifferenceAverage_1	0.109
lbp-3D-m2_glcm_DifferenceEntropy_1	0.043
lbp-3D-m2_glcm_DifferenceVariance_1	0.055
lbp-3D-m2_glcm_Id_1	0.096

lbp-3D-m2_glcm_Idm_1	0.100
lbp-3D-m2_glcm_Idmn_1	0.103
lbp-3D-m2_glcm_Idn_1	0.014
lbp-3D-m2_glcm_Imc1_1	0.084
lbp-3D-m2_glcm_Imc2_1	0.323
lbp-3D-m2_glcm_InverseVariance_1	0.209
lbp-3D-m2_glcm_JointAverage_1	0.262
lbp-3D-m2_glcm_JointEnergy_1	0.011
lbp-3D-m2_glcm_JointEntropy_1	0.004
lbp-3D-m2_glcm_MCC_1	0.278
lbp-3D-m2_glcm_MaximumProbability_1	0.042
lbp-3D-m2_glcm_SumAverage_1	0.262
lbp-3D-m2_glcm_SumEntropy_1	0.027
lbp-3D-m2_glcm_SumSquares_1	0.055
lbp-3D-m2_gldm_DependenceEntropy_1	0.445
lbp-3D-m2_gldm_DependenceNonUniformity_1	0.539
lbp-3D-m2_gldm_DependenceNonUniformityNormalized_1	0.441
lbp-3D-m2_gldm_DependenceVariance_1	0.447
lbp-3D-m2_gldm_GrayLevelNonUniformity_1	0.600
lbp-3D-m2_gldm_GrayLevelVariance_1	0.072
lbp-3D-m2_gldm_HighGrayLevelEmphasis_1	0.336
lbp-3D-m2_gldm_LargeDependenceEmphasis_1	0.557
lbp-3D-m2_gldm_LargeDependenceHighGrayLevelEmphasis_1	0.318
lbp-3D-m2_gldm_LargeDependenceLowGrayLevelEmphasis_1	0.465
lbp-3D-m2_gldm_LowGrayLevelEmphasis_1	0.264
lbp-3D-m2_gldm_SmallDependenceEmphasis_1	0.303
lbp-3D-m2_gldm_SmallDependenceHighGrayLevelEmphasis_1	0.252
lbp-3D-m2_gldm_SmallDependenceLowGrayLevelEmphasis_1	0.441
lbp-3D-m2_glrlm_GrayLevelNonUniformity_1	0.620
lbp-3D-m2_glrlm_GrayLevelNonUniformityNormalized_1	0.040
lbp-3D-m2_glrlm_GrayLevelVariance_1	0.090
lbp-3D-m2_glrlm_HighGrayLevelRunEmphasis_1	0.326
lbp-3D-m2_glrlm_LongRunEmphasis_1	0.823
lbp-3D-m2_glrlm_LongRunHighGrayLevelEmphasis_1	0.458
lbp-3D-m2_glrlm_LongRunLowGrayLevelEmphasis_1	0.492
lbp-3D-m2_glrlm_LowGrayLevelRunEmphasis_1	0.252
lbp-3D-m2_glrlm_RunEntropy_1	0.149
lbp-3D-m2_glrlm_RunLengthNonUniformity_1	0.595
lbp-3D-m2_glrlm_RunLengthNonUniformityNormalized_1	0.392
lbp-3D-m2_glrlm_RunPercentage_1	0.490
lbp-3D-m2_glrlm_RunVariance_1	0.776
lbp-3D-m2_glrlm_ShortRunEmphasis_1	0.521
lbp-3D-m2_glrlm_ShortRunHighGrayLevelEmphasis_1	0.343
lbp-3D-m2_glrlm_ShortRunLowGrayLevelEmphasis_1	0.247
lbp-3D-m2_glszm_GrayLevelNonUniformity_1	0.816
lbp-3D-m2_glszm_GrayLevelNonUniformityNormalized_1	0.094
lbp-3D-m2_glszm_GrayLevelVariance_1	0.161
lbp-3D-m2_glszm_HighGrayLevelZoneEmphasis_1	0.008
lbp-3D-m2_glszm_LargeAreaEmphasis_1	0.037
lbp-3D-m2_glszm_LargeAreaHighGrayLevelEmphasis_1	0.049

lbp-3D-m2_glszm_LargeAreaLowGrayLevelEmphasis_1	0.014
lbp-3D-m2_glszm_LowGrayLevelZoneEmphasis_1	0.106
lbp-3D-m2_glszm_SizeZoneNonUniformity_1	0.740
lbp-3D-m2_glszm_SizeZoneNonUniformityNormalized_1	0.740
lbp-3D-m2_glszm_SmallAreaEmphasis_1	0.725
lbp-3D-m2_glszm_SmallAreaHighGrayLevelEmphasis_1	0.309
lbp-3D-m2_glszm_SmallAreaLowGrayLevelEmphasis_1	0.080
lbp-3D-m2_glszm_ZoneEntropy_1	0.738
lbp-3D-m2_glszm_ZonePercentage_1	0.150
lbp-3D-m2_glszm_ZoneVariance_1	0.037
lbp-3D-m2_ngtdm_Busyness_1	0.239
lbp-3D-m2_ngtdm_Coarseness_1	0.242
lbp-3D-m2_ngtdm_Complexity_1	0.494
lbp-3D-m2_ngtdm_Contrast_1	0.051
lbp-3D-m2_ngtdm_Strength_1	0.441
lbp-3D-k_firstorder_10Percentile_1	0.116
lbp-3D-k_firstorder_90Percentile_1	0.340
lbp-3D-k_firstorder_Energy_1	0.594
lbp-3D-k_firstorder_Entropy_1	0.404
lbp-3D-k_firstorder_InterquartileRange_1	0.359
lbp-3D-k_firstorder_Kurtosis_1	0.829
lbp-3D-k_firstorder_Maximum_1	0.637
lbp-3D-k_firstorder_MeanAbsoluteDeviation_1	0.545
lbp-3D-k_firstorder_Mean_1	0.170
lbp-3D-k_firstorder_Median_1	0.067
lbp-3D-k_firstorder_Minimum_1	0.778
lbp-3D-k_firstorder_Range_1	0.641
lbp-3D-k_firstorder_RobustMeanAbsoluteDeviation_1	0.412
lbp-3D-k_firstorder_RootMeanSquared_1	0.077
lbp-3D-k_firstorder_Skewness_1	0.711
lbp-3D-k_firstorder_TotalEnergy_1	0.594
lbp-3D-k_firstorder_Uniformity_1	0.449
lbp-3D-k_firstorder_Variance_1	0.679
lbp-3D-k_glcm_Autocorrelation_1	0.361
lbp-3D-k_glcm_ClusterProminence_1	0.424
lbp-3D-k_glcm_ClusterShade_1	0.369
lbp-3D-k_glcm_ClusterTendency_1	0.353
lbp-3D-k_glcm_Contrast_1	0.263
lbp-3D-k_glcm_Correlation_1	0.101
lbp-3D-k_glcm_DifferenceAverage_1	0.263
lbp-3D-k_glcm_DifferenceEntropy_1	0.169
lbp-3D-k_glcm_DifferenceVariance_1	0.214
lbp-3D-k_glcm_Id_1	0.263
lbp-3D-k_glcm_Idm_1	0.263
lbp-3D-k_glcm_Idmn_1	0.299
lbp-3D-k_glcm_Idn_1	0.268
lbp-3D-k_glcm_Imc1_1	0.248
lbp-3D-k_glcm_Imc2_1	0.453
lbp-3D-k_glcm_InverseVariance_1	0.263
lbp-3D-k_glcm_JointAverage_1	0.339

lbp-3D-k_glcm_JointEnergy_1	0.275
lbp-3D-k_glcm_JointEntropy_1	0.254
lbp-3D-k_glcm_MCC_1	0.477
lbp-3D-k_glcm_MaximumProbability_1	0.307
lbp-3D-k_glcm_SumAverage_1	0.339
lbp-3D-k_glcm_SumEntropy_1	0.252
lbp-3D-k_glcm_SumSquares_1	0.315
lbp-3D-k_gldm_DependenceEntropy_1	0.173
lbp-3D-k_gldm_DependenceNonUniformity_1	0.275
lbp-3D-k_gldm_DependenceNonUniformityNormalized_1	0.099
lbp-3D-k_gldm_DependenceVariance_1	0.302
lbp-3D-k_gldm_GrayLevelNonUniformity_1	0.562
lbp-3D-k_gldm_GrayLevelVariance_1	0.450
lbp-3D-k_gldm_HighGrayLevelEmphasis_1	0.470
lbp-3D-k_gldm_LargeDependenceEmphasis_1	0.190
lbp-3D-k_gldm_LargeDependenceHighGrayLevelEmphasis_1	0.162
lbp-3D-k_gldm_LargeDependenceLowGrayLevelEmphasis_1	0.198
lbp-3D-k_gldm_LowGrayLevelEmphasis_1	0.469
lbp-3D-k_gldm_SmallDependenceEmphasis_1	0.289
lbp-3D-k_gldm_SmallDependenceHighGrayLevelEmphasis_1	0.405
lbp-3D-k_gldm_SmallDependenceLowGrayLevelEmphasis_1	0.182
lbp-3D-k_glrlm_GrayLevelNonUniformity_1	0.758
lbp-3D-k_glrlm_GrayLevelNonUniformityNormalized_1	0.054
lbp-3D-k_glrlm_GrayLevelVariance_1	0.058
lbp-3D-k_glrlm_HighGrayLevelRunEmphasis_1	0.002
lbp-3D-k_glrlm_LongRunEmphasis_1	0.161
lbp-3D-k_glrlm_LongRunHighGrayLevelEmphasis_1	0.150
lbp-3D-k_glrlm_LongRunLowGrayLevelEmphasis_1	0.163
lbp-3D-k_glrlm_LowGrayLevelRunEmphasis_1	0.000
lbp-3D-k_glrlm_RunEntropy_1	0.059
lbp-3D-k_glrlm_RunLengthNonUniformity_1	0.778
lbp-3D-k_glrlm_RunLengthNonUniformityNormalized_1	0.142
lbp-3D-k_glrlm_RunPercentage_1	0.169
lbp-3D-k_glrlm_RunVariance_1	0.143
lbp-3D-k_glrlm_ShortRunEmphasis_1	0.228
lbp-3D-k_glrlm_ShortRunHighGrayLevelEmphasis_1	0.410
lbp-3D-k_glrlm_ShortRunLowGrayLevelEmphasis_1	0.048
lbp-3D-k_glszm_GrayLevelNonUniformity_1	0.677
lbp-3D-k_glszm_GrayLevelNonUniformityNormalized_1	0.105
lbp-3D-k_glszm_GrayLevelVariance_1	0.106
lbp-3D-k_glszm_HighGrayLevelZoneEmphasis_1	0.164
lbp-3D-k_glszm_LargeAreaEmphasis_1	0.265
lbp-3D-k_glszm_LargeAreaHighGrayLevelEmphasis_1	0.263
lbp-3D-k_glszm_LargeAreaLowGrayLevelEmphasis_1	0.265
lbp-3D-k_glszm_LowGrayLevelZoneEmphasis_1	0.169
lbp-3D-k_glszm_SizeZoneNonUniformity_1	0.699
lbp-3D-k_glszm_SizeZoneNonUniformityNormalized_1	0.529
lbp-3D-k_glszm_SmallAreaEmphasis_1	0.449
lbp-3D-k_glszm_SmallAreaHighGrayLevelEmphasis_1	0.133
lbp-3D-k_glszm_SmallAreaLowGrayLevelEmphasis_1	0.553

lbp-3D-k_glszm_ZoneEntropy_1	0.437
lbp-3D-k_glszm_ZonePercentage_1	0.249
lbp-3D-k_glszm_ZoneVariance_1	0.265
lbp-3D-k_ngtdm_Busyness_1	0.421
lbp-3D-k_ngtdm_Coarseness_1	0.006
lbp-3D-k_ngtdm_Complexity_1	0.704
lbp-3D-k_ngtdm_Contrast_1	0.484
lbp-3D-k_ngtdm_Strength_1	0.008
logarithm_firstorder_10Percentile_1	0.958
logarithm_firstorder_90Percentile_1	0.990
logarithm_firstorder_Energy_1	0.621
logarithm_firstorder_Entropy_1	0.000
logarithm_firstorder_InterquartileRange_1	0.110
logarithm_firstorder_Kurtosis_1	0.695
logarithm_firstorder_Maximum_1	0.978
logarithm_firstorder_MeanAbsoluteDeviation_1	0.209
logarithm_firstorder_Mean_1	0.980
logarithm_firstorder_Median_1	0.983
logarithm_firstorder_Minimum_1	0.725
logarithm_firstorder_Range_1	0.853
logarithm_firstorder_RobustMeanAbsoluteDeviation_1	0.109
logarithm_firstorder_RootMeanSquared_1	0.981
logarithm_firstorder_Skewness_1	0.548
logarithm_firstorder_TotalEnergy_1	0.621
logarithm_firstorder_Variance_1	0.168
logarithm_glcm_DifferenceEntropy_1	0.000
logarithm_glcm_JointEntropy_1	0.000
logarithm_glcm_SumEntropy_1	0.000
logarithm_gldm_DependenceEntropy_1	0.308
logarithm_gldm_DependenceNonUniformity_1	0.078
logarithm_gldm_DependenceNonUniformityNormalized_1	0.113
logarithm_gldm_DependenceVariance_1	0.308
logarithm_gldm_GrayLevelNonUniformity_1	0.547
logarithm_gldm_LargeDependenceEmphasis_1	0.033
logarithm_gldm_LargeDependenceHighGrayLevelEmphasis_1	0.070
logarithm_gldm_LargeDependenceLowGrayLevelEmphasis_1	0.022
logarithm_gldm_SmallDependenceEmphasis_1	0.081
logarithm_gldm_SmallDependenceHighGrayLevelEmphasis_1	0.024
logarithm_gldm_SmallDependenceLowGrayLevelEmphasis_1	0.093
logarithm_glrlm_GrayLevelNonUniformity_1	0.727
logarithm_glrlm_LongRunEmphasis_1	0.010
logarithm_glrlm_LongRunHighGrayLevelEmphasis_1	0.010
logarithm_glrlm_LongRunLowGrayLevelEmphasis_1	0.010
logarithm_glrlm_RunEntropy_1	0.057
logarithm_glrlm_RunLengthNonUniformity_1	0.661
logarithm_glrlm_RunLengthNonUniformityNormalized_1	0.080
logarithm_glrlm_RunPercentage_1	0.005
logarithm_glrlm_RunVariance_1	0.053
logarithm_glrlm_ShortRunEmphasis_1	0.136
logarithm_glrlm_ShortRunHighGrayLevelEmphasis_1	0.080

logarithm_glrlm_ShortRunLowGrayLevelEmphasis_1	0.098
logarithm_glszm_GrayLevelNonUniformity_1	0.218
logarithm_glszm_LargeAreaEmphasis_1	0.007
logarithm_glszm_LargeAreaHighGrayLevelEmphasis_1	0.007
logarithm_glszm_LargeAreaLowGrayLevelEmphasis_1	0.007
logarithm_glszm_SizeZoneNonUniformity_1	0.271
logarithm_glszm_SizeZoneNonUniformityNormalized_1	0.123
logarithm_glszm_SmallAreaEmphasis_1	0.710
logarithm_glszm_SmallAreaHighGrayLevelEmphasis_1	0.708
logarithm_glszm_SmallAreaLowGrayLevelEmphasis_1	0.710
logarithm_glszm_ZoneEntropy_1	0.165
logarithm_glszm_ZonePercentage_1	0.195
logarithm_glszm_ZoneVariance_1	0.063
square_firstorder_10Percentile_1	0.789
square_firstorder_90Percentile_1	0.899
square_firstorder_Energy_1	0.601
square_firstorder_Entropy_1	0.000
square_firstorder_InterquartileRange_1	0.162
square_firstorder_Kurtosis_1	0.435
square_firstorder_Maximum_1	0.755
square_firstorder_MeanAbsoluteDeviation_1	0.263
square_firstorder_Mean_1	0.871
square_firstorder_Median_1	0.884
square_firstorder_Minimum_1	0.711
square_firstorder_Range_1	0.873
square_firstorder_RobustMeanAbsoluteDeviation_1	0.166
square_firstorder_RootMeanSquared_1	0.876
square_firstorder_Skewness_1	0.377
square_firstorder_TotalEnergy_1	0.601
square_firstorder_Variance_1	0.285
square_glcm_DifferenceEntropy_1	0.000
square_glcm_JointEntropy_1	0.000
square_glcm_SumEntropy_1	0.000
square_gldm_DependenceEntropy_1	0.308
square_gldm_DependenceNonUniformity_1	0.078
square_gldm_DependenceNonUniformityNormalized_1	0.113
square_gldm_DependenceVariance_1	0.308
square_gldm_GrayLevelNonUniformity_1	0.547
square_gldm_LargeDependenceEmphasis_1	0.033
square_gldm_LargeDependenceHighGrayLevelEmphasis_1	0.033
square_gldm_LargeDependenceLowGrayLevelEmphasis_1	0.033
square_gldm_SmallDependenceEmphasis_1	0.081
square_gldm_SmallDependenceHighGrayLevelEmphasis_1	0.081
square_gldm_SmallDependenceLowGrayLevelEmphasis_1	0.081
square_glrlm_GrayLevelNonUniformity_1	0.727
square_glrlm_LongRunEmphasis_1	0.010
square_glrlm_LongRunHighGrayLevelEmphasis_1	0.010
square_glrlm_LongRunLowGrayLevelEmphasis_1	0.010
square_glrlm_RunEntropy_1	0.057
square_glrlm_RunLengthNonUniformity_1	0.661

square_glrlm_RunLengthNonUniformityNormalized_1	0.080
square_glrlm_RunPercentage_1	0.005
square_glrlm_RunVariance_1	0.053
square_glrlm_ShortRunEmphasis_1	0.136
square_glrlm_ShortRunHighGrayLevelEmphasis_1	0.136
square_glrlm_ShortRunLowGrayLevelEmphasis_1	0.136
square_glszm_GrayLevelNonUniformity_1	0.217
square_glszm_LargeAreaEmphasis_1	0.007
square_glszm_LargeAreaHighGrayLevelEmphasis_1	0.007
square_glszm_LargeAreaLowGrayLevelEmphasis_1	0.007
square_glszm_SizeZoneNonUniformity_1	0.271
square_glszm_SizeZoneNonUniformityNormalized_1	0.123
square_glszm_SmallAreaEmphasis_1	0.710
square_glszm_SmallAreaHighGrayLevelEmphasis_1	0.710
square_glszm_SmallAreaLowGrayLevelEmphasis_1	0.710
square_glszm_ZoneEntropy_1	0.166
square_glszm_ZonePercentage_1	0.195
square_glszm_ZoneVariance_1	0.063
squareroot_firstorder_10Percentile_1	0.966
squareroot_firstorder_90Percentile_1	0.986
squareroot_firstorder_Energy_1	0.620
squareroot_firstorder_Entropy_1	0.000
squareroot_firstorder_InterquartileRange_1	0.136
squareroot_firstorder_Kurtosis_1	0.693
squareroot_firstorder_Maximum_1	0.980
squareroot_firstorder_MeanAbsoluteDeviation_1	0.232
squareroot_firstorder_Mean_1	0.980
squareroot_firstorder_Median_1	0.981
squareroot_firstorder_Minimum_1	0.685
squareroot_firstorder_Range_1	0.756
squareroot_firstorder_RobustMeanAbsoluteDeviation_1	0.133
squareroot_firstorder_RootMeanSquared_1	0.980
squareroot_firstorder_Skewness_1	0.540
squareroot_firstorder_TotalEnergy_1	0.620
squareroot_firstorder_Variance_1	0.182
squareroot_glcm_DifferenceEntropy_1	0.000
squareroot_glcm_JointEntropy_1	0.000
squareroot_glcm_SumEntropy_1	0.000
squareroot_gldm_DependenceEntropy_1	0.308
squareroot_gldm_DependenceNonUniformity_1	0.078
squareroot_gldm_DependenceNonUniformityNormalized_1	0.113
squareroot_gldm_DependenceVariance_1	0.308
squareroot_gldm_GrayLevelNonUniformity_1	0.547
squareroot_gldm_LargeDependenceEmphasis_1	0.033
squareroot_gldm_LargeDependenceHighGrayLevelEmphasis_1	0.070
squareroot_gldm_LargeDependenceLowGrayLevelEmphasis_1	0.022
squareroot_gldm_SmallDependenceEmphasis_1	0.081
squareroot_gldm_SmallDependenceHighGrayLevelEmphasis_1	0.024
squareroot_gldm_SmallDependenceLowGrayLevelEmphasis_1	0.093
squareroot_glrlm_GrayLevelNonUniformity_1	0.727

squareroot_glrlm_LongRunEmphasis_1	0.010
squareroot_glrlm_LongRunHighGrayLevelEmphasis_1	0.010
squareroot_glrlm_LongRunLowGrayLevelEmphasis_1	0.010
squareroot_glrlm_RunEntropy_1	0.057
squareroot_glrlm_RunLengthNonUniformity_1	0.661
squareroot_glrlm_RunLengthNonUniformityNormalized_1	0.080
squareroot_glrlm_RunPercentage_1	0.005
squareroot_glrlm_RunVariance_1	0.053
squareroot_glrlm_ShortRunEmphasis_1	0.136
squareroot_glrlm_ShortRunHighGrayLevelEmphasis_1	0.080
squareroot_glrlm_ShortRunLowGrayLevelEmphasis_1	0.098
squareroot_glszm_GrayLevelNonUniformity_1	0.218
squareroot_glszm_LargeAreaEmphasis_1	0.007
squareroot_glszm_LargeAreaHighGrayLevelEmphasis_1	0.007
squareroot_glszm_LargeAreaLowGrayLevelEmphasis_1	0.007
squareroot_glszm_SizeZoneNonUniformity_1	0.271
squareroot_glszm_SizeZoneNonUniformityNormalized_1	0.123
squareroot_glszm_SmallAreaEmphasis_1	0.710
squareroot_glszm_SmallAreaHighGrayLevelEmphasis_1	0.708
squareroot_glszm_SmallAreaLowGrayLevelEmphasis_1	0.710
squareroot_glszm_ZoneEntropy_1	0.165
squareroot_glszm_ZonePercentage_1	0.195
squareroot_glszm_ZoneVariance_1	0.063
wavelet-LLH_firstorder_10Percentile_1	0.602
wavelet-LLH_firstorder_90Percentile_1	0.110
wavelet-LLH_firstorder_Energy_1	0.874
wavelet-LLH_firstorder_Entropy_1	0.562
wavelet-LLH_firstorder_InterquartileRange_1	0.187
wavelet-LLH_firstorder_Kurtosis_1	0.611
wavelet-LLH_firstorder_Maximum_1	0.655
wavelet-LLH_firstorder_MeanAbsoluteDeviation_1	0.443
wavelet-LLH_firstorder_Mean_1	0.579
wavelet-LLH_firstorder_Median_1	0.630
wavelet-LLH_firstorder_Minimum_1	0.940
wavelet-LLH_firstorder_Range_1	0.845
wavelet-LLH_firstorder_RobustMeanAbsoluteDeviation_1	0.241
wavelet-LLH_firstorder_RootMeanSquared_1	0.604
wavelet-LLH_firstorder_Skewness_1	0.706
wavelet-LLH_firstorder_TotalEnergy_1	0.874
wavelet-LLH_firstorder_Uniformity_1	0.564
wavelet-LLH_firstorder_Variance_1	0.314
wavelet-LLH_glcm_Autocorrelation_1	0.354
wavelet-LLH_glcm_ClusterProminence_1	0.769
wavelet-LLH_glcm_ClusterShade_1	0.263
wavelet-LLH_glcm_ClusterTendency_1	0.658
wavelet-LLH_glcm_Contrast_1	0.721
wavelet-LLH_glcm_Correlation_1	0.710
wavelet-LLH_glcm_DifferenceAverage_1	0.721
wavelet-LLH_glcm_DifferenceEntropy_1	0.616
wavelet-LLH_glcm_DifferenceVariance_1	0.644

wavelet-LLH_glcm_Id_1	0.721
wavelet-LLH_glcm_Idm_1	0.721
wavelet-LLH_glcm_Idmn_1	0.721
wavelet-LLH_glcm_Idn_1	0.721
wavelet-LLH_glcm_Imc1_1	0.505
wavelet-LLH_glcm_Imc2_1	0.739
wavelet-LLH_glcm_InverseVariance_1	0.721
wavelet-LLH_glcm_JointAverage_1	0.401
wavelet-LLH_glcm_JointEnergy_1	0.687
wavelet-LLH_glcm_JointEntropy_1	0.716
wavelet-LLH_glcm_MCC_1	0.699
wavelet-LLH_glcm_MaximumProbability_1	0.616
wavelet-LLH_glcm_SumAverage_1	0.401
wavelet-LLH_glcm_SumEntropy_1	0.728
wavelet-LLH_glcm_SumSquares_1	0.672
wavelet-LLH_gldm_DependenceEntropy_1	0.444
wavelet-LLH_gldm_DependenceNonUniformity_1	0.445
wavelet-LLH_gldm_DependenceNonUniformityNormalized_1	0.202
wavelet-LLH_gldm_DependenceVariance_1	0.478
wavelet-LLH_gldm_GrayLevelNonUniformity_1	0.543
wavelet-LLH_gldm_GrayLevelVariance_1	0.564
wavelet-LLH_gldm_HighGrayLevelEmphasis_1	0.358
wavelet-LLH_gldm_LargeDependenceEmphasis_1	0.275
wavelet-LLH_gldm_LargeDependenceHighGrayLevelEmphasis_1	0.472
wavelet-LLH_gldm_LargeDependenceLowGrayLevelEmphasis_1	0.185
wavelet-LLH_gldm_LowGrayLevelEmphasis_1	0.358
wavelet-LLH_gldm_SmallDependenceEmphasis_1	0.169
wavelet-LLH_gldm_SmallDependenceHighGrayLevelEmphasis_1	0.159
wavelet-LLH_gldm_SmallDependenceLowGrayLevelEmphasis_1	0.183
wavelet-LLH_glrlm_GrayLevelNonUniformity_1	0.670
wavelet-LLH_glrlm_GrayLevelNonUniformityNormalized_1	0.685
wavelet-LLH_glrlm_GrayLevelVariance_1	0.685
wavelet-LLH_glrlm_HighGrayLevelRunEmphasis_1	0.490
wavelet-LLH_glrlm_LongRunEmphasis_1	0.126
wavelet-LLH_glrlm_LongRunHighGrayLevelEmphasis_1	0.235
wavelet-LLH_glrlm_LongRunLowGrayLevelEmphasis_1	0.098
wavelet-LLH_glrlm_LowGrayLevelRunEmphasis_1	0.490
wavelet-LLH_glrlm_RunEntropy_1	0.218
wavelet-LLH_glrlm_RunLengthNonUniformity_1	0.750
wavelet-LLH_glrlm_RunLengthNonUniformityNormalized_1	0.154
wavelet-LLH_glrlm_RunPercentage_1	0.218
wavelet-LLH_glrlm_RunVariance_1	0.071
wavelet-LLH_glrlm_ShortRunEmphasis_1	0.168
wavelet-LLH_glrlm_ShortRunHighGrayLevelEmphasis_1	0.305
wavelet-LLH_glrlm_ShortRunLowGrayLevelEmphasis_1	0.077
wavelet-LLH_glszm_GrayLevelNonUniformity_1	0.601
wavelet-LLH_glszm_GrayLevelNonUniformityNormalized_1	0.033
wavelet-LLH_glszm_GrayLevelVariance_1	0.033
wavelet-LLH_glszm_HighGrayLevelZoneEmphasis_1	0.079
wavelet-LLH_glszm_LargeAreaEmphasis_1	0.008

wavelet-LLH_glszm_LargeAreaHighGrayLevelEmphasis_1	0.002
wavelet-LLH_glszm_LargeAreaLowGrayLevelEmphasis_1	0.013
wavelet-LLH_glszm_LowGrayLevelZoneEmphasis_1	0.079
wavelet-LLH_glszm_SizeZoneNonUniformity_1	0.560
wavelet-LLH_glszm_SizeZoneNonUniformityNormalized_1	0.405
wavelet-LLH_glszm_SmallAreaEmphasis_1	0.460
wavelet-LLH_glszm_SmallAreaHighGrayLevelEmphasis_1	0.160
wavelet-LLH_glszm_SmallAreaLowGrayLevelEmphasis_1	0.615
wavelet-LLH_glszm_ZoneEntropy_1	0.452
wavelet-LLH_glszm_ZonePercentage_1	0.148
wavelet-LLH_glszm_ZoneVariance_1	0.007
wavelet-LLH_ngtdm_Busyness_1	0.031
wavelet-LLH_ngtdm_Coarseness_1	0.595
wavelet-LLH_ngtdm_Complexity_1	0.742
wavelet-LLH_ngtdm_Contrast_1	0.775
wavelet-LLH_ngtdm_Strength_1	0.567
wavelet-LHL_firstorder_10Percentile_1	0.160
wavelet-LHL_firstorder_90Percentile_1	0.892
wavelet-LHL_firstorder_Energy_1	0.779
wavelet-LHL_firstorder_Entropy_1	0.060
wavelet-LHL_firstorder_InterquartileRange_1	0.361
wavelet-LHL_firstorder_Kurtosis_1	0.648
wavelet-LHL_firstorder_Maximum_1	0.875
wavelet-LHL_firstorder_MeanAbsoluteDeviation_1	0.405
wavelet-LHL_firstorder_Mean_1	0.006
wavelet-LHL_firstorder_Median_1	0.023
wavelet-LHL_firstorder_Minimum_1	0.859
wavelet-LHL_firstorder_Range_1	0.898
wavelet-LHL_firstorder_RobustMeanAbsoluteDeviation_1	0.372
wavelet-LHL_firstorder_RootMeanSquared_1	0.339
wavelet-LHL_firstorder_Skewness_1	0.719
wavelet-LHL_firstorder_TotalEnergy_1	0.779
wavelet-LHL_firstorder_Uniformity_1	0.068
wavelet-LHL_firstorder_Variance_1	0.202
wavelet-LHL_glcm_Autocorrelation_1	0.301
wavelet-LHL_glcm_ClusterProminence_1	0.280
wavelet-LHL_glcm_ClusterShade_1	0.401
wavelet-LHL_glcm_ClusterTendency_1	0.223
wavelet-LHL_glcm_Contrast_1	0.158
wavelet-LHL_glcm_Correlation_1	0.277
wavelet-LHL_glcm_DifferenceAverage_1	0.158
wavelet-LHL_glcm_DifferenceEntropy_1	0.223
wavelet-LHL_glcm_DifferenceVariance_1	0.233
wavelet-LHL_glcm_Id_1	0.158
wavelet-LHL_glcm_Idm_1	0.158
wavelet-LHL_glcm_Idmn_1	0.158
wavelet-LHL_glcm_Idn_1	0.158
wavelet-LHL_glcm_Imc1_1	0.487
wavelet-LHL_glcm_Imc2_1	0.464
wavelet-LHL_glcm_InverseVariance_1	0.158

wavelet-LHL_glcm_JointAverage_1	0.270
wavelet-LHL_glcm_JointEnergy_1	0.018
wavelet-LHL_glcm_JointEntropy_1	0.028
wavelet-LHL_glcm_MCC_1	0.471
wavelet-LHL_glcm_MaximumProbability_1	0.113
wavelet-LHL_glcm_SumAverage_1	0.270
wavelet-LHL_glcm_SumEntropy_1	0.105
wavelet-LHL_glcm_SumSquares_1	0.020
wavelet-LHL_gldm_DependenceEntropy_1	0.331
wavelet-LHL_gldm_DependenceNonUniformity_1	0.502
wavelet-LHL_gldm_DependenceNonUniformityNormalized_1	0.357
wavelet-LHL_gldm_DependenceVariance_1	0.526
wavelet-LHL_gldm_GrayLevelNonUniformity_1	0.552
wavelet-LHL_gldm_GrayLevelVariance_1	0.068
wavelet-LHL_gldm_HighGrayLevelEmphasis_1	0.327
wavelet-LHL_gldm_LargeDependenceEmphasis_1	0.154
wavelet-LHL_gldm_LargeDependenceHighGrayLevelEmphasis_1	0.149
wavelet-LHL_gldm_LargeDependenceLowGrayLevelEmphasis_1	0.214
wavelet-LHL_gldm_LowGrayLevelEmphasis_1	0.327
wavelet-LHL_gldm_SmallDependenceEmphasis_1	0.047
wavelet-LHL_gldm_SmallDependenceHighGrayLevelEmphasis_1	0.036
wavelet-LHL_gldm_SmallDependenceLowGrayLevelEmphasis_1	0.061
wavelet-LHL_glrlm_GrayLevelNonUniformity_1	0.629
wavelet-LHL_glrlm_GrayLevelNonUniformityNormalized_1	0.044
wavelet-LHL_glrlm_GrayLevelVariance_1	0.044
wavelet-LHL_glrlm_HighGrayLevelRunEmphasis_1	0.230
wavelet-LHL_glrlm_LongRunEmphasis_1	0.506
wavelet-LHL_glrlm_LongRunHighGrayLevelEmphasis_1	0.485
wavelet-LHL_glrlm_LongRunLowGrayLevelEmphasis_1	0.529
wavelet-LHL_glrlm_LowGrayLevelRunEmphasis_1	0.230
wavelet-LHL_glrlm_RunEntropy_1	0.101
wavelet-LHL_glrlm_RunLengthNonUniformity_1	0.714
wavelet-LHL_glrlm_RunLengthNonUniformityNormalized_1	0.025
wavelet-LHL_glrlm_RunPercentage_1	0.092
wavelet-LHL_glrlm_RunVariance_1	0.490
wavelet-LHL_glrlm_ShortRunEmphasis_1	0.074
wavelet-LHL_glrlm_ShortRunHighGrayLevelEmphasis_1	0.293
wavelet-LHL_glrlm_ShortRunLowGrayLevelEmphasis_1	0.067
wavelet-LHL_glszm_GrayLevelNonUniformity_1	0.506
wavelet-LHL_glszm_GrayLevelNonUniformityNormalized_1	0.295
wavelet-LHL_glszm_GrayLevelVariance_1	0.295
wavelet-LHL_glszm_HighGrayLevelZoneEmphasis_1	0.368
wavelet-LHL_glszm_LargeAreaEmphasis_1	0.013
wavelet-LHL_glszm_LargeAreaHighGrayLevelEmphasis_1	0.015
wavelet-LHL_glszm_LargeAreaLowGrayLevelEmphasis_1	0.011
wavelet-LHL_glszm_LowGrayLevelZoneEmphasis_1	0.368
wavelet-LHL_glszm_SizeZoneNonUniformity_1	0.572
wavelet-LHL_glszm_SizeZoneNonUniformityNormalized_1	0.454
wavelet-LHL_glszm_SmallAreaEmphasis_1	0.577
wavelet-LHL_glszm_SmallAreaHighGrayLevelEmphasis_1	0.470

wavelet-LHL_glszm_SmallAreaLowGrayLevelEmphasis_1	0.609
wavelet-LHL_glszm_ZoneEntropy_1	0.270
wavelet-LHL_glszm_ZonePercentage_1	0.067
wavelet-LHL_glszm_ZoneVariance_1	0.012
wavelet-LHL_ngtdm_Busyness_1	0.609
wavelet-LHL_ngtdm_Coarseness_1	0.254
wavelet-LHL_ngtdm_Complexity_1	0.087
wavelet-LHL_ngtdm_Contrast_1	0.084
wavelet-LHL_ngtdm_Strength_1	0.262
wavelet-LHH_firstorder_10Percentile_1	0.612
wavelet-LHH_firstorder_90Percentile_1	0.185
wavelet-LHH_firstorder_Energy_1	0.883
wavelet-LHH_firstorder_Entropy_1	0.010
wavelet-LHH_firstorder_InterquartileRange_1	0.438
wavelet-LHH_firstorder_Kurtosis_1	0.678
wavelet-LHH_firstorder_Maximum_1	0.852
wavelet-LHH_firstorder_MeanAbsoluteDeviation_1	0.361
wavelet-LHH_firstorder_Mean_1	0.007
wavelet-LHH_firstorder_Median_1	0.009
wavelet-LHH_firstorder_Minimum_1	0.819
wavelet-LHH_firstorder_Range_1	0.878
wavelet-LHH_firstorder_RobustMeanAbsoluteDeviation_1	0.385
wavelet-LHH_firstorder_RootMeanSquared_1	0.356
wavelet-LHH_firstorder_Skewness_1	0.630
wavelet-LHH_firstorder_TotalEnergy_1	0.883
wavelet-LHH_firstorder_Uniformity_1	0.010
wavelet-LHH_firstorder_Variance_1	0.086
wavelet-LHH_glcm_Autocorrelation_1	0.106
wavelet-LHH_glcm_ClusterProminence_1	0.669
wavelet-LHH_glcm_ClusterShade_1	0.219
wavelet-LHH_glcm_ClusterTendency_1	0.691
wavelet-LHH_glcm_Contrast_1	0.747
wavelet-LHH_glcm_Correlation_1	0.750
wavelet-LHH_glcm_DifferenceAverage_1	0.747
wavelet-LHH_glcm_DifferenceEntropy_1	0.700
wavelet-LHH_glcm_DifferenceVariance_1	0.701
wavelet-LHH_glcm_Id_1	0.747
wavelet-LHH_glcm_Idm_1	0.747
wavelet-LHH_glcm_Idmn_1	0.747
wavelet-LHH_glcm_Idn_1	0.747
wavelet-LHH_glcm_Imc1_1	0.668
wavelet-LHH_glcm_Imc2_1	0.723
wavelet-LHH_glcm_InverseVariance_1	0.747
wavelet-LHH_glcm_JointAverage_1	0.158
wavelet-LHH_glcm_JointEnergy_1	0.289
wavelet-LHH_glcm_JointEntropy_1	0.287
wavelet-LHH_glcm_MCC_1	0.727
wavelet-LHH_glcm_MaximumProbability_1	0.320
wavelet-LHH_glcm_SumAverage_1	0.158
wavelet-LHH_glcm_SumEntropy_1	0.236

wavelet-LHH_glcm_SumSquares_1	0.063
wavelet-LHH_gldm_DependenceEntropy_1	0.433
wavelet-LHH_gldm_DependenceNonUniformity_1	0.451
wavelet-LHH_gldm_DependenceNonUniformityNormalized_1	0.341
wavelet-LHH_gldm_DependenceVariance_1	0.695
wavelet-LHH_gldm_GrayLevelNonUniformity_1	0.548
wavelet-LHH_gldm_GrayLevelVariance_1	0.010
wavelet-LHH_gldm_HighGrayLevelEmphasis_1	0.097
wavelet-LHH_gldm_LargeDependenceEmphasis_1	0.211
wavelet-LHH_gldm_LargeDependenceHighGrayLevelEmphasis_1	0.204
wavelet-LHH_gldm_LargeDependenceLowGrayLevelEmphasis_1	0.218
wavelet-LHH_gldm_LowGrayLevelEmphasis_1	0.097
wavelet-LHH_gldm_SmallDependenceEmphasis_1	0.052
wavelet-LHH_gldm_SmallDependenceHighGrayLevelEmphasis_1	0.065
wavelet-LHH_gldm_SmallDependenceLowGrayLevelEmphasis_1	0.040
wavelet-LHH_glrlm_GrayLevelNonUniformity_1	0.623
wavelet-LHH_glrlm_GrayLevelNonUniformityNormalized_1	0.007
wavelet-LHH_glrlm_GrayLevelVariance_1	0.007
wavelet-LHH_glrlm_HighGrayLevelRunEmphasis_1	0.145
wavelet-LHH_glrlm_LongRunEmphasis_1	0.431
wavelet-LHH_glrlm_LongRunHighGrayLevelEmphasis_1	0.442
wavelet-LHH_glrlm_LongRunLowGrayLevelEmphasis_1	0.419
wavelet-LHH_glrlm_LowGrayLevelRunEmphasis_1	0.145
wavelet-LHH_glrlm_RunEntropy_1	0.138
wavelet-LHH_glrlm_RunLengthNonUniformity_1	0.711
wavelet-LHH_glrlm_RunLengthNonUniformityNormalized_1	0.067
wavelet-LHH_glrlm_RunPercentage_1	0.126
wavelet-LHH_glrlm_RunVariance_1	0.339
wavelet-LHH_glrlm_ShortRunEmphasis_1	0.126
wavelet-LHH_glrlm_ShortRunHighGrayLevelEmphasis_1	0.127
wavelet-LHH_glrlm_ShortRunLowGrayLevelEmphasis_1	0.134
wavelet-LHH_glszm_GrayLevelNonUniformity_1	0.519
wavelet-LHH_glszm_GrayLevelNonUniformityNormalized_1	0.295
wavelet-LHH_glszm_GrayLevelVariance_1	0.295
wavelet-LHH_glszm_HighGrayLevelZoneEmphasis_1	0.372
wavelet-LHH_glszm_LargeAreaEmphasis_1	0.010
wavelet-LHH_glszm_LargeAreaHighGrayLevelEmphasis_1	0.008
wavelet-LHH_glszm_LargeAreaLowGrayLevelEmphasis_1	0.011
wavelet-LHH_glszm_LowGrayLevelZoneEmphasis_1	0.372
wavelet-LHH_glszm_SizeZoneNonUniformity_1	0.559
wavelet-LHH_glszm_SizeZoneNonUniformityNormalized_1	0.418
wavelet-LHH_glszm_SmallAreaEmphasis_1	0.631
wavelet-LHH_glszm_SmallAreaHighGrayLevelEmphasis_1	0.595
wavelet-LHH_glszm_SmallAreaLowGrayLevelEmphasis_1	0.565
wavelet-LHH_glszm_ZoneEntropy_1	0.269
wavelet-LHH_glszm_ZonePercentage_1	0.087
wavelet-LHH_glszm_ZoneVariance_1	0.009
wavelet-LHH_ngtdm_Busyness_1	0.453
wavelet-LHH_ngtdm_Coarseness_1	0.406
wavelet-LHH_ngtdm_Complexity_1	0.634

wavelet-LHH_ngtdm_Contrast_1	0.576
wavelet-LHH_ngtdm_Strength_1	0.403
wavelet-HLL_firstorder_10Percentile_1	0.335
wavelet-HLL_firstorder_90Percentile_1	0.908
wavelet-HLL_firstorder_Energy_1	0.846
wavelet-HLL_firstorder_Entropy_1	0.459
wavelet-HLL_firstorder_InterquartileRange_1	0.602
wavelet-HLL_firstorder_Kurtosis_1	0.621
wavelet-HLL_firstorder_Maximum_1	0.694
wavelet-HLL_firstorder_MeanAbsoluteDeviation_1	0.471
wavelet-HLL_firstorder_Mean_1	0.370
wavelet-HLL_firstorder_Median_1	0.360
wavelet-HLL_firstorder_Minimum_1	0.884
wavelet-HLL_firstorder_Range_1	0.912
wavelet-HLL_firstorder_RobustMeanAbsoluteDeviation_1	0.532
wavelet-HLL_firstorder_RootMeanSquared_1	0.508
wavelet-HLL_firstorder_Skewness_1	0.641
wavelet-HLL_firstorder_TotalEnergy_1	0.846
wavelet-HLL_firstorder_Uniformity_1	0.457
wavelet-HLL_firstorder_Variance_1	0.430
wavelet-HLL_glcm_Autocorrelation_1	0.537
wavelet-HLL_glcm_ClusterProminence_1	0.439
wavelet-HLL_glcm_ClusterShade_1	0.467
wavelet-HLL_glcm_ClusterTendency_1	0.471
wavelet-HLL_glcm_Contrast_1	0.399
wavelet-HLL_glcm_Correlation_1	0.397
wavelet-HLL_glcm_DifferenceAverage_1	0.399
wavelet-HLL_glcm_DifferenceEntropy_1	0.523
wavelet-HLL_glcm_DifferenceVariance_1	0.516
wavelet-HLL_glcm_Id_1	0.399
wavelet-HLL_glcm_Idm_1	0.399
wavelet-HLL_glcm_Idmn_1	0.399
wavelet-HLL_glcm_Idn_1	0.399
wavelet-HLL_glcm_Imc1_1	0.673
wavelet-HLL_glcm_Imc2_1	0.585
wavelet-HLL_glcm_InverseVariance_1	0.399
wavelet-HLL_glcm_JointAverage_1	0.515
wavelet-HLL_glcm_JointEnergy_1	0.420
wavelet-HLL_glcm_JointEntropy_1	0.416
wavelet-HLL_glcm_MCC_1	0.610
wavelet-HLL_glcm_MaximumProbability_1	0.399
wavelet-HLL_glcm_SumAverage_1	0.515
wavelet-HLL_glcm_SumEntropy_1	0.460
wavelet-HLL_glcm_SumSquares_1	0.437
wavelet-HLL_gldm_DependenceEntropy_1	0.337
wavelet-HLL_gldm_DependenceNonUniformity_1	0.480
wavelet-HLL_gldm_DependenceNonUniformityNormalized_1	0.268
wavelet-HLL_gldm_DependenceVariance_1	0.490
wavelet-HLL_gldm_GrayLevelNonUniformity_1	0.547
wavelet-HLL_gldm_GrayLevelVariance_1	0.457

wavelet-HLL_gldm_HighGrayLevelEmphasis_1	0.528
wavelet-HLL_gldm_LargeDependenceEmphasis_1	0.148
wavelet-HLL_gldm_LargeDependenceHighGrayLevelEmphasis_1	0.256
wavelet-HLL_gldm_LargeDependenceLowGrayLevelEmphasis_1	0.127
wavelet-HLL_gldm_LowGrayLevelEmphasis_1	0.528
wavelet-HLL_gldm_SmallDependenceEmphasis_1	0.046
wavelet-HLL_gldm_SmallDependenceHighGrayLevelEmphasis_1	0.034
wavelet-HLL_gldm_SmallDependenceLowGrayLevelEmphasis_1	0.062
wavelet-HLL_glrlm_GrayLevelNonUniformity_1	0.627
wavelet-HLL_glrlm_GrayLevelNonUniformityNormalized_1	0.436
wavelet-HLL_glrlm_GrayLevelVariance_1	0.436
wavelet-HLL_glrlm_HighGrayLevelRunEmphasis_1	0.436
wavelet-HLL_glrlm_LongRunEmphasis_1	0.483
wavelet-HLL_glrlm_LongRunHighGrayLevelEmphasis_1	0.513
wavelet-HLL_glrlm_LongRunLowGrayLevelEmphasis_1	0.460
wavelet-HLL_glrlm_LowGrayLevelRunEmphasis_1	0.436
wavelet-HLL_glrlm_RunEntropy_1	0.114
wavelet-HLL_glrlm_RunLengthNonUniformity_1	0.717
wavelet-HLL_glrlm_RunLengthNonUniformityNormalized_1	0.028
wavelet-HLL_glrlm_RunPercentage_1	0.091
wavelet-HLL_glrlm_RunVariance_1	0.463
wavelet-HLL_glrlm_ShortRunEmphasis_1	0.075
wavelet-HLL_glrlm_ShortRunHighGrayLevelEmphasis_1	0.420
wavelet-HLL_glrlm_ShortRunLowGrayLevelEmphasis_1	0.142
wavelet-HLL_glszm_GrayLevelNonUniformity_1	0.468
wavelet-HLL_glszm_GrayLevelNonUniformityNormalized_1	0.128
wavelet-HLL_glszm_GrayLevelVariance_1	0.128
wavelet-HLL_glszm_HighGrayLevelZoneEmphasis_1	0.274
wavelet-HLL_glszm_LargeAreaEmphasis_1	0.006
wavelet-HLL_glszm_LargeAreaHighGrayLevelEmphasis_1	0.006
wavelet-HLL_glszm_LargeAreaLowGrayLevelEmphasis_1	0.006
wavelet-HLL_glszm_LowGrayLevelZoneEmphasis_1	0.274
wavelet-HLL_glszm_SizeZoneNonUniformity_1	0.503
wavelet-HLL_glszm_SizeZoneNonUniformityNormalized_1	0.467
wavelet-HLL_glszm_SmallAreaEmphasis_1	0.630
wavelet-HLL_glszm_SmallAreaHighGrayLevelEmphasis_1	0.492
wavelet-HLL_glszm_SmallAreaLowGrayLevelEmphasis_1	0.558
wavelet-HLL_glszm_ZoneEntropy_1	0.294
wavelet-HLL_glszm_ZonePercentage_1	0.041
wavelet-HLL_glszm_ZoneVariance_1	0.006
wavelet-HLL_ngtdm_Busyness_1	0.362
wavelet-HLL_ngtdm_Coarseness_1	0.335
wavelet-HLL_ngtdm_Complexity_1	0.239
wavelet-HLL_ngtdm_Contrast_1	0.281
wavelet-HLL_ngtdm_Strength_1	0.331
wavelet-HLH_firstorder_10Percentile_1	0.667
wavelet-HLH_firstorder_90Percentile_1	0.490
wavelet-HLH_firstorder_Energy_1	0.838
wavelet-HLH_firstorder_Entropy_1	0.610
wavelet-HLH_firstorder_InterquartileRange_1	0.602

wavelet-HLH_firstorder_Kurtosis_1	0.731
wavelet-HLH_firstorder_Maximum_1	0.950
wavelet-HLH_firstorder_MeanAbsoluteDeviation_1	0.613
wavelet-HLH_firstorder_Mean_1	0.434
wavelet-HLH_firstorder_Median_1	0.195
wavelet-HLH_firstorder_Minimum_1	0.843
wavelet-HLH_firstorder_Range_1	0.945
wavelet-HLH_firstorder_RobustMeanAbsoluteDeviation_1	0.590
wavelet-HLH_firstorder_RootMeanSquared_1	0.671
wavelet-HLH_firstorder_Skewness_1	0.583
wavelet-HLH_firstorder_TotalEnergy_1	0.838
wavelet-HLH_firstorder_Uniformity_1	0.610
wavelet-HLH_firstorder_Variance_1	0.564
wavelet-HLH_glcm_Autocorrelation_1	0.162
wavelet-HLH_glcm_ClusterProminence_1	0.381
wavelet-HLH_glcm_ClusterShade_1	0.206
wavelet-HLH_glcm_ClusterTendency_1	0.446
wavelet-HLH_glcm_Contrast_1	0.419
wavelet-HLH_glcm_Correlation_1	0.422
wavelet-HLH_glcm_DifferenceAverage_1	0.419
wavelet-HLH_glcm_DifferenceEntropy_1	0.865
wavelet-HLH_glcm_DifferenceVariance_1	0.862
wavelet-HLH_glcm_Id_1	0.419
wavelet-HLH_glcm_Idm_1	0.419
wavelet-HLH_glcm_Idmn_1	0.419
wavelet-HLH_glcm_Idn_1	0.419
wavelet-HLH_glcm_Imc1_1	0.872
wavelet-HLH_glcm_Imc2_1	0.826
wavelet-HLH_glcm_InverseVariance_1	0.419
wavelet-HLH_glcm_JointAverage_1	0.177
wavelet-HLH_glcm_JointEnergy_1	0.635
wavelet-HLH_glcm_JointEntropy_1	0.645
wavelet-HLH_glcm_MCC_1	0.835
wavelet-HLH_glcm_MaximumProbability_1	0.624
wavelet-HLH_glcm_SumAverage_1	0.177
wavelet-HLH_glcm_SumEntropy_1	0.308
wavelet-HLH_glcm_SumSquares_1	0.360
wavelet-HLH_gldm_DependenceEntropy_1	0.447
wavelet-HLH_gldm_DependenceNonUniformity_1	0.433
wavelet-HLH_gldm_DependenceNonUniformityNormalized_1	0.280
wavelet-HLH_gldm_DependenceVariance_1	0.613
wavelet-HLH_gldm_GrayLevelNonUniformity_1	0.548
wavelet-HLH_gldm_GrayLevelVariance_1	0.610
wavelet-HLH_gldm_HighGrayLevelEmphasis_1	0.350
wavelet-HLH_gldm_LargeDependenceEmphasis_1	0.229
wavelet-HLH_gldm_LargeDependenceHighGrayLevelEmphasis_1	0.231
wavelet-HLH_gldm_LargeDependenceLowGrayLevelEmphasis_1	0.229
wavelet-HLH_gldm_LowGrayLevelEmphasis_1	0.350
wavelet-HLH_gldm_SmallDependenceEmphasis_1	0.088
wavelet-HLH_gldm_SmallDependenceHighGrayLevelEmphasis_1	0.084

wavelet-HLH_gldm_SmallDependenceLowGrayLevelEmphasis_1	0.092
wavelet-HLH_glrlm_GrayLevelNonUniformity_1	0.623
wavelet-HLH_glrlm_GrayLevelNonUniformityNormalized_1	0.378
wavelet-HLH_glrlm_GrayLevelVariance_1	0.378
wavelet-HLH_glrlm_HighGrayLevelRunEmphasis_1	0.499
wavelet-HLH_glrlm_LongRunEmphasis_1	0.433
wavelet-HLH_glrlm_LongRunHighGrayLevelEmphasis_1	0.439
wavelet-HLH_glrlm_LongRunLowGrayLevelEmphasis_1	0.428
wavelet-HLH_glrlm_LowGrayLevelRunEmphasis_1	0.499
wavelet-HLH_glrlm_RunEntropy_1	0.154
wavelet-HLH_glrlm_RunLengthNonUniformity_1	0.712
wavelet-HLH_glrlm_RunLengthNonUniformityNormalized_1	0.069
wavelet-HLH_glrlm_RunPercentage_1	0.139
wavelet-HLH_glrlm_RunVariance_1	0.344
wavelet-HLH_glrlm_ShortRunEmphasis_1	0.133
wavelet-HLH_glrlm_ShortRunHighGrayLevelEmphasis_1	0.166
wavelet-HLH_glrlm_ShortRunLowGrayLevelEmphasis_1	0.152
wavelet-HLH_glszm_GrayLevelNonUniformity_1	0.493
wavelet-HLH_glszm_GrayLevelNonUniformityNormalized_1	0.046
wavelet-HLH_glszm_GrayLevelVariance_1	0.046
wavelet-HLH_glszm_HighGrayLevelZoneEmphasis_1	0.296
wavelet-HLH_glszm_LargeAreaEmphasis_1	0.008
wavelet-HLH_glszm_LargeAreaHighGrayLevelEmphasis_1	0.008
wavelet-HLH_glszm_LargeAreaLowGrayLevelEmphasis_1	0.008
wavelet-HLH_glszm_LowGrayLevelZoneEmphasis_1	0.296
wavelet-HLH_glszm_SizeZoneNonUniformity_1	0.495
wavelet-HLH_glszm_SizeZoneNonUniformityNormalized_1	0.365
wavelet-HLH_glszm_SmallAreaEmphasis_1	0.584
wavelet-HLH_glszm_SmallAreaHighGrayLevelEmphasis_1	0.473
wavelet-HLH_glszm_SmallAreaLowGrayLevelEmphasis_1	0.626
wavelet-HLH_glszm_ZoneEntropy_1	0.276
wavelet-HLH_glszm_ZonePercentage_1	0.080
wavelet-HLH_glszm_ZoneVariance_1	0.008
wavelet-HLH_ngtdm_Busyness_1	0.507
wavelet-HLH_ngtdm_Coarseness_1	0.399
wavelet-HLH_ngtdm_Complexity_1	0.480
wavelet-HLH_ngtdm_Contrast_1	0.480
wavelet-HLH_ngtdm_Strength_1	0.398
wavelet-HHL_firstorder_10Percentile_1	0.946
wavelet-HHL_firstorder_90Percentile_1	0.915
wavelet-HHL_firstorder_Energy_1	0.684
wavelet-HHL_firstorder_Entropy_1	0.243
wavelet-HHL_firstorder_InterquartileRange_1	0.959
wavelet-HHL_firstorder_Kurtosis_1	0.936
wavelet-HHL_firstorder_Maximum_1	0.934
wavelet-HHL_firstorder_MeanAbsoluteDeviation_1	0.906
wavelet-HHL_firstorder_Mean_1	0.204
wavelet-HHL_firstorder_Median_1	0.236
wavelet-HHL_firstorder_Minimum_1	0.873
wavelet-HHL_firstorder_Range_1	0.965

wavelet-HHL_firstorder_RobustMeanAbsoluteDeviation_1	0.953
wavelet-HHL_firstorder_RootMeanSquared_1	0.810
wavelet-HHL_firstorder_Skewness_1	0.486
wavelet-HHL_firstorder_TotalEnergy_1	0.684
wavelet-HHL_firstorder_Uniformity_1	0.244
wavelet-HHL_firstorder_Variance_1	0.824
wavelet-HHL_glcm_Autocorrelation_1	0.230
wavelet-HHL_glcm_ClusterProminence_1	0.330
wavelet-HHL_glcm_ClusterShade_1	0.225
wavelet-HHL_glcm_ClusterTendency_1	0.354
wavelet-HHL_glcm_Contrast_1	0.283
wavelet-HHL_glcm_Correlation_1	0.331
wavelet-HHL_glcm_DifferenceAverage_1	0.283
wavelet-HHL_glcm_DifferenceEntropy_1	0.856
wavelet-HHL_glcm_DifferenceVariance_1	0.832
wavelet-HHL_glcm_Id_1	0.283
wavelet-HHL_glcm_Idm_1	0.283
wavelet-HHL_glcm_Idmn_1	0.283
wavelet-HHL_glcm_Idn_1	0.283
wavelet-HHL_glcm_Imc1_1	0.822
wavelet-HHL_glcm_Imc2_1	0.669
wavelet-HHL_glcm_InverseVariance_1	0.283
wavelet-HHL_glcm_JointAverage_1	0.230
wavelet-HHL_glcm_JointEnergy_1	0.558
wavelet-HHL_glcm_JointEntropy_1	0.598
wavelet-HHL_glcm_MCC_1	0.703
wavelet-HHL_glcm_MaximumProbability_1	0.405
wavelet-HHL_glcm_SumAverage_1	0.230
wavelet-HHL_glcm_SumEntropy_1	0.618
wavelet-HHL_glcm_SumSquares_1	0.075
wavelet-HHL_gldm_DependenceEntropy_1	0.309
wavelet-HHL_gldm_DependenceNonUniformity_1	0.426
wavelet-HHL_gldm_DependenceNonUniformityNormalized_1	0.248
wavelet-HHL_gldm_DependenceVariance_1	0.432
wavelet-HHL_gldm_GrayLevelNonUniformity_1	0.547
wavelet-HHL_gldm_GrayLevelVariance_1	0.244
wavelet-HHL_gldm_HighGrayLevelEmphasis_1	0.233
wavelet-HHL_gldm_LargeDependenceEmphasis_1	0.093
wavelet-HHL_gldm_LargeDependenceHighGrayLevelEmphasis_1	0.077
wavelet-HHL_gldm_LargeDependenceLowGrayLevelEmphasis_1	0.117
wavelet-HHL_gldm_LowGrayLevelEmphasis_1	0.233
wavelet-HHL_gldm_SmallDependenceEmphasis_1	0.120
wavelet-HHL_gldm_SmallDependenceHighGrayLevelEmphasis_1	0.120
wavelet-HHL_gldm_SmallDependenceLowGrayLevelEmphasis_1	0.120
wavelet-HHL_glrlm_GrayLevelNonUniformity_1	0.623
wavelet-HHL_glrlm_GrayLevelNonUniformityNormalized_1	0.194
wavelet-HHL_glrlm_GrayLevelVariance_1	0.194
wavelet-HHL_glrlm_HighGrayLevelRunEmphasis_1	0.181
wavelet-HHL_glrlm_LongRunEmphasis_1	0.547
wavelet-HHL_glrlm_LongRunHighGrayLevelEmphasis_1	0.515

wavelet-HHL_glrlm_LongRunLowGrayLevelEmphasis_1	0.577
wavelet-HHL_glrlm_LowGrayLevelRunEmphasis_1	0.181
wavelet-HHL_glrlm_RunEntropy_1	0.073
wavelet-HHL_glrlm_RunLengthNonUniformity_1	0.719
wavelet-HHL_glrlm_RunLengthNonUniformityNormalized_1	0.002
wavelet-HHL_glrlm_RunPercentage_1	0.055
wavelet-HHL_glrlm_RunVariance_1	0.518
wavelet-HHL_glrlm_ShortRunEmphasis_1	0.055
wavelet-HHL_glrlm_ShortRunHighGrayLevelEmphasis_1	0.115
wavelet-HHL_glrlm_ShortRunLowGrayLevelEmphasis_1	0.003
wavelet-HHL_glszm_GrayLevelNonUniformity_1	0.453
wavelet-HHL_glszm_GrayLevelNonUniformityNormalized_1	0.065
wavelet-HHL_glszm_GrayLevelVariance_1	0.065
wavelet-HHL_glszm_HighGrayLevelZoneEmphasis_1	0.127
wavelet-HHL_glszm_LargeAreaEmphasis_1	0.003
wavelet-HHL_glszm_LargeAreaHighGrayLevelEmphasis_1	0.003
wavelet-HHL_glszm_LargeAreaLowGrayLevelEmphasis_1	0.003
wavelet-HHL_glszm_LowGrayLevelZoneEmphasis_1	0.127
wavelet-HHL_glszm_SizeZoneNonUniformity_1	0.519
wavelet-HHL_glszm_SizeZoneNonUniformityNormalized_1	0.400
wavelet-HHL_glszm_SmallAreaEmphasis_1	0.685
wavelet-HHL_glszm_SmallAreaHighGrayLevelEmphasis_1	0.704
wavelet-HHL_glszm_SmallAreaLowGrayLevelEmphasis_1	0.476
wavelet-HHL_glszm_ZoneEntropy_1	0.216
wavelet-HHL_glszm_ZonePercentage_1	0.098
wavelet-HHL_glszm_ZoneVariance_1	0.004
wavelet-HHL_ngtdm_Busyness_1	0.550
wavelet-HHL_ngtdm_Coarseness_1	0.377
wavelet-HHL_ngtdm_Complexity_1	0.182
wavelet-HHL_ngtdm_Contrast_1	0.176
wavelet-HHL_ngtdm_Strength_1	0.377
wavelet-HHH_firstorder_10Percentile_1	0.732
wavelet-HHH_firstorder_90Percentile_1	0.805
wavelet-HHH_firstorder_Energy_1	0.745
wavelet-HHH_firstorder_Entropy_1	0.061
wavelet-HHH_firstorder_InterquartileRange_1	0.836
wavelet-HHH_firstorder_Kurtosis_1	0.794
wavelet-HHH_firstorder_Maximum_1	0.942
wavelet-HHH_firstorder_MeanAbsoluteDeviation_1	0.736
wavelet-HHH_firstorder_Mean_1	0.169
wavelet-HHH_firstorder_Median_1	0.134
wavelet-HHH_firstorder_Minimum_1	0.915
wavelet-HHH_firstorder_Range_1	0.961
wavelet-HHH_firstorder_RobustMeanAbsoluteDeviation_1	0.826
wavelet-HHH_firstorder_RootMeanSquared_1	0.684
wavelet-HHH_firstorder_Skewness_1	0.106
wavelet-HHH_firstorder_TotalEnergy_1	0.745
wavelet-HHH_firstorder_Uniformity_1	0.061
wavelet-HHH_firstorder_Variance_1	0.471
wavelet-HHH_glcm_Autocorrelation_1	0.330

wavelet-HHH_glcm_ClusterProminence_1	0.198
wavelet-HHH_glcm_ClusterShade_1	0.295
wavelet-HHH_glcm_ClusterTendency_1	0.238
wavelet-HHH_glcm_Contrast_1	0.315
wavelet-HHH_glcm_Correlation_1	0.308
wavelet-HHH_glcm_DifferenceAverage_1	0.315
wavelet-HHH_glcm_DifferenceEntropy_1	0.910
wavelet-HHH_glcm_DifferenceVariance_1	0.901
wavelet-HHH_glcm_Id_1	0.315
wavelet-HHH_glcm_Idm_1	0.315
wavelet-HHH_glcm_Idmn_1	0.315
wavelet-HHH_glcm_Idn_1	0.315
wavelet-HHH_glcm_Imc1_1	0.850
wavelet-HHH_glcm_Imc2_1	0.800
wavelet-HHH_glcm_InverseVariance_1	0.315
wavelet-HHH_glcm_JointAverage_1	0.324
wavelet-HHH_glcm_JointEnergy_1	0.509
wavelet-HHH_glcm_JointEntropy_1	0.481
wavelet-HHH_glcm_MCC_1	0.820
wavelet-HHH_glcm_MaximumProbability_1	0.466
wavelet-HHH_glcm_SumAverage_1	0.324
wavelet-HHH_glcm_SumEntropy_1	0.415
wavelet-HHH_glcm_SumSquares_1	0.071
wavelet-HHH_gldm_DependenceEntropy_1	0.372
wavelet-HHH_gldm_DependenceNonUniformity_1	0.397
wavelet-HHH_gldm_DependenceNonUniformityNormalized_1	0.303
wavelet-HHH_gldm_DependenceVariance_1	0.463
wavelet-HHH_gldm_GrayLevelNonUniformity_1	0.547
wavelet-HHH_gldm_GrayLevelVariance_1	0.061
wavelet-HHH_gldm_HighGrayLevelEmphasis_1	0.292
wavelet-HHH_gldm_LargeDependenceEmphasis_1	0.102
wavelet-HHH_gldm_LargeDependenceHighGrayLevelEmphasis_1	0.122
wavelet-HHH_gldm_LargeDependenceLowGrayLevelEmphasis_1	0.085
wavelet-HHH_gldm_LowGrayLevelEmphasis_1	0.292
wavelet-HHH_gldm_SmallDependenceEmphasis_1	0.058
wavelet-HHH_gldm_SmallDependenceHighGrayLevelEmphasis_1	0.053
wavelet-HHH_gldm_SmallDependenceLowGrayLevelEmphasis_1	0.062
wavelet-HHH_glrlm_GrayLevelNonUniformity_1	0.623
wavelet-HHH_glrlm_GrayLevelNonUniformityNormalized_1	0.046
wavelet-HHH_glrlm_GrayLevelVariance_1	0.046
wavelet-HHH_glrlm_HighGrayLevelRunEmphasis_1	0.253
wavelet-HHH_glrlm_LongRunEmphasis_1	0.467
wavelet-HHH_glrlm_LongRunHighGrayLevelEmphasis_1	0.486
wavelet-HHH_glrlm_LongRunLowGrayLevelEmphasis_1	0.448
wavelet-HHH_glrlm_LowGrayLevelRunEmphasis_1	0.253
wavelet-HHH_glrlm_RunEntropy_1	0.076
wavelet-HHH_glrlm_RunLengthNonUniformity_1	0.720
wavelet-HHH_glrlm_RunLengthNonUniformityNormalized_1	0.013
wavelet-HHH_glrlm_RunPercentage_1	0.057
wavelet-HHH_glrlm_RunVariance_1	0.326

wavelet-HHH_glrlm_ShortRunEmphasis_1	0.078
wavelet-HHH_glrlm_ShortRunHighGrayLevelEmphasis_1	0.070
wavelet-HHH_glrlm_ShortRunLowGrayLevelEmphasis_1	0.087
wavelet-HHH_glszm_GrayLevelNonUniformity_1	0.453
wavelet-HHH_glszm_GrayLevelNonUniformityNormalized_1	0.053
wavelet-HHH_glszm_GrayLevelVariance_1	0.053
wavelet-HHH_glszm_HighGrayLevelZoneEmphasis_1	0.152
wavelet-HHH_glszm_LargeAreaEmphasis_1	0.004
wavelet-HHH_glszm_LargeAreaHighGrayLevelEmphasis_1	0.004
wavelet-HHH_glszm_LargeAreaLowGrayLevelEmphasis_1	0.004
wavelet-HHH_glszm_LowGrayLevelZoneEmphasis_1	0.152
wavelet-HHH_glszm_SizeZoneNonUniformity_1	0.505
wavelet-HHH_glszm_SizeZoneNonUniformityNormalized_1	0.533
wavelet-HHH_glszm_SmallAreaEmphasis_1	0.640
wavelet-HHH_glszm_SmallAreaHighGrayLevelEmphasis_1	0.530
wavelet-HHH_glszm_SmallAreaLowGrayLevelEmphasis_1	0.651
wavelet-HHH_glszm_ZoneEntropy_1	0.258
wavelet-HHH_glszm_ZonePercentage_1	0.097
wavelet-HHH_glszm_ZoneVariance_1	0.005
wavelet-HHH_ngtdm_Busyness_1	0.512
wavelet-HHH_ngtdm_Coarseness_1	0.422
wavelet-HHH_ngtdm_Complexity_1	0.497
wavelet-HHH_ngtdm_Contrast_1	0.491
wavelet-HHH_ngtdm_Strength_1	0.422
wavelet-LLL_firstorder_10Percentile_1	0.909
wavelet-LLL_firstorder_90Percentile_1	0.989
wavelet-LLL_firstorder_Energy_1	0.607
wavelet-LLL_firstorder_Entropy_1	0.005
wavelet-LLL_firstorder_InterquartileRange_1	0.058
wavelet-LLL_firstorder_Kurtosis_1	0.533
wavelet-LLL_firstorder_Maximum_1	0.953
wavelet-LLL_firstorder_MeanAbsoluteDeviation_1	0.187
wavelet-LLL_firstorder_Mean_1	0.965
wavelet-LLL_firstorder_Median_1	0.971
wavelet-LLL_firstorder_Minimum_1	0.686
wavelet-LLL_firstorder_Range_1	0.890
wavelet-LLL_firstorder_RobustMeanAbsoluteDeviation_1	0.062
wavelet-LLL_firstorder_RootMeanSquared_1	0.967
wavelet-LLL_firstorder_Skewness_1	0.510
wavelet-LLL_firstorder_TotalEnergy_1	0.607
wavelet-LLL_firstorder_Uniformity_1	0.004
wavelet-LLL_firstorder_Variance_1	0.179
wavelet-LLL_glcm_Autocorrelation_1	0.005
wavelet-LLL_glcm_ClusterProminence_1	0.004
wavelet-LLL_glcm_ClusterShade_1	0.004
wavelet-LLL_glcm_ClusterTendency_1	0.005
wavelet-LLL_glcm_Contrast_1	0.005
wavelet-LLL_glcm_Correlation_1	0.018
wavelet-LLL_glcm_DifferenceAverage_1	0.005
wavelet-LLL_glcm_DifferenceEntropy_1	0.006

wavelet-LLL_glcm_DifferenceVariance_1	0.005
wavelet-LLL_glcm_Id_1	0.005
wavelet-LLL_glcm_Idm_1	0.005
wavelet-LLL_glcm_Idmn_1	0.005
wavelet-LLL_glcm_Idn_1	0.005
wavelet-LLL_glcm_Imc1_1	0.017
wavelet-LLL_glcm_Imc2_1	0.015
wavelet-LLL_glcm_InverseVariance_1	0.005
wavelet-LLL_glcm_JointAverage_1	0.005
wavelet-LLL_glcm_JointEnergy_1	0.005
wavelet-LLL_glcm_JointEntropy_1	0.006
wavelet-LLL_glcm_MCC_1	0.018
wavelet-LLL_glcm_MaximumProbability_1	0.005
wavelet-LLL_glcm_SumAverage_1	0.005
wavelet-LLL_glcm_SumEntropy_1	0.006
wavelet-LLL_glcm_SumSquares_1	0.005
wavelet-LLL_gldm_DependenceEntropy_1	0.308
wavelet-LLL_gldm_DependenceNonUniformity_1	0.078
wavelet-LLL_gldm_DependenceNonUniformityNormalized_1	0.113
wavelet-LLL_gldm_DependenceVariance_1	0.308
wavelet-LLL_gldm_GrayLevelNonUniformity_1	0.547
wavelet-LLL_gldm_GrayLevelVariance_1	0.004
wavelet-LLL_gldm_HighGrayLevelEmphasis_1	0.004
wavelet-LLL_gldm_LargeDependenceEmphasis_1	0.033
wavelet-LLL_gldm_LargeDependenceHighGrayLevelEmphasis_1	0.033
wavelet-LLL_gldm_LargeDependenceLowGrayLevelEmphasis_1	0.033
wavelet-LLL_gldm_LowGrayLevelEmphasis_1	0.004
wavelet-LLL_gldm_SmallDependenceEmphasis_1	0.081
wavelet-LLL_gldm_SmallDependenceHighGrayLevelEmphasis_1	0.081
wavelet-LLL_gldm_SmallDependenceLowGrayLevelEmphasis_1	0.081
wavelet-LLL_glrlm_GrayLevelNonUniformity_1	0.727
wavelet-LLL_glrlm_GrayLevelNonUniformityNormalized_1	0.006
wavelet-LLL_glrlm_GrayLevelVariance_1	0.006
wavelet-LLL_glrlm_HighGrayLevelRunEmphasis_1	0.006
wavelet-LLL_glrlm_LongRunEmphasis_1	0.010
wavelet-LLL_glrlm_LongRunHighGrayLevelEmphasis_1	0.010
wavelet-LLL_glrlm_LongRunLowGrayLevelEmphasis_1	0.010
wavelet-LLL_glrlm_LowGrayLevelRunEmphasis_1	0.006
wavelet-LLL_glrlm_RunEntropy_1	0.058
wavelet-LLL_glrlm_RunLengthNonUniformity_1	0.661
wavelet-LLL_glrlm_RunLengthNonUniformityNormalized_1	0.080
wavelet-LLL_glrlm_RunPercentage_1	0.005
wavelet-LLL_glrlm_RunVariance_1	0.053
wavelet-LLL_glrlm_ShortRunEmphasis_1	0.135
wavelet-LLL_glrlm_ShortRunHighGrayLevelEmphasis_1	0.135
wavelet-LLL_glrlm_ShortRunLowGrayLevelEmphasis_1	0.136
wavelet-LLL_glszm_GrayLevelNonUniformity_1	0.218
wavelet-LLL_glszm_GrayLevelNonUniformityNormalized_1	0.018
wavelet-LLL_glszm_GrayLevelVariance_1	0.018
wavelet-LLL_glszm_HighGrayLevelZoneEmphasis_1	0.018

wavelet-LLL_glszm_LargeAreaEmphasis_1	0.012
wavelet-LLL_glszm_LargeAreaHighGrayLevelEmphasis_1	0.012
wavelet-LLL_glszm_LargeAreaLowGrayLevelEmphasis_1	0.012
wavelet-LLL_glszm_LowGrayLevelZoneEmphasis_1	0.018
wavelet-LLL_glszm_SizeZoneNonUniformity_1	0.271
wavelet-LLL_glszm_SizeZoneNonUniformityNormalized_1	0.154
wavelet-LLL_glszm_SmallAreaEmphasis_1	0.668
wavelet-LLL_glszm_SmallAreaHighGrayLevelEmphasis_1	0.345
wavelet-LLL_glszm_SmallAreaLowGrayLevelEmphasis_1	0.706
wavelet-LLL_glszm_ZoneEntropy_1	0.192
wavelet-LLL_glszm_ZonePercentage_1	0.195
wavelet-LLL_glszm_ZoneVariance_1	0.009
wavelet-LLL_ngtdm_Busyness_1	0.016
wavelet-LLL_ngtdm_Coarseness_1	0.018
wavelet-LLL_ngtdm_Complexity_1	0.004
wavelet-LLL_ngtdm_Contrast_1	0.000
wavelet-LLL_ngtdm_Strength_1	0.015
original_shape_Elongation_2	0.603
original_shape_Flatness_2	0.833
original_shape_LeastAxisLength_2	0.869
original_shape_MajorAxisLength_2	0.826
original_shape_Maximum2DDiameterColumn_2	0.781
original_shape_Maximum2DDiameterRow_2	0.924
original_shape_Maximum2DDiameterSlice_2	0.872
original_shape_Maximum3DDiameter_2	0.914
original_shape_MeshVolume_2	0.499
original_shape_MinorAxisLength_2	0.798
original_shape_Sphericity_2	0.348
original_shape_SurfaceArea_2	0.541
original_shape_SurfaceVolumeRatio_2	0.206
original_shape_VoxelVolume_2	0.509
original_firstorder_10Percentile_2	0.682
original_firstorder_90Percentile_2	0.841
original_firstorder_Energy_2	0.477
original_firstorder_Entropy_2	0.000
original_firstorder_InterquartileRange_2	0.029
original_firstorder_Kurtosis_2	0.448
original_firstorder_Maximum_2	0.977
original_firstorder_MeanAbsoluteDeviation_2	0.165
original_firstorder_Mean_2	0.850
original_firstorder_Median_2	0.879
original_firstorder_Minimum_2	0.196
original_firstorder_Range_2	0.837
original_firstorder_RobustMeanAbsoluteDeviation_2	0.048
original_firstorder_RootMeanSquared_2	0.856
original_firstorder_Skewness_2	0.574
original_firstorder_TotalEnergy_2	0.477
original_firstorder_Uniformity_2	0.000
original_firstorder_Variance_2	0.173
original_glcm_Autocorrelation_2	0.000

original_glcm_ClusterProminence_2	0.000
original_glcm_ClusterShade_2	0.000
original_glcm_ClusterTendency_2	0.000
original_glcm_Contrast_2	0.000
original_glcm_Correlation_2	0.000
original_glcm_DifferenceAverage_2	0.000
original_glcm_DifferenceEntropy_2	0.000
original_glcm_DifferenceVariance_2	0.000
original_glcm_Id_2	0.000
original_glcm_Idm_2	0.000
original_glcm_Idmn_2	0.000
original_glcm_Idn_2	0.000
original_glcm_Imc1_2	0.000
original_glcm_Imc2_2	0.000
original_glcm_InverseVariance_2	0.000
original_glcm_JointAverage_2	0.000
original_glcm_JointEnergy_2	0.000
original_glcm_JointEntropy_2	0.000
original_glcm_MCC_2	0.000
original_glcm_MaximumProbability_2	0.000
original_glcm_SumAverage_2	0.000
original_glcm_SumEntropy_2	0.000
original_glcm_SumSquares_2	0.000
original_gldm_DependenceEntropy_2	0.231
original_gldm_DependenceNonUniformity_2	0.195
original_gldm_DependenceNonUniformityNormalized_2	0.122
original_gldm_DependenceVariance_2	0.426
original_gldm_GrayLevelNonUniformity_2	0.509
original_gldm_GrayLevelVariance_2	0.000
original_gldm_HighGrayLevelEmphasis_2	0.000
original_gldm_LargeDependenceEmphasis_2	0.219
original_gldm_LargeDependenceHighGrayLevelEmphasis_2	0.220
original_gldm_LargeDependenceLowGrayLevelEmphasis_2	0.194
original_gldm_LowGrayLevelEmphasis_2	0.000
original_gldm_SmallDependenceEmphasis_2	0.220
original_gldm_SmallDependenceHighGrayLevelEmphasis_2	0.172
original_gldm_SmallDependenceLowGrayLevelEmphasis_2	0.228
original_glrlm_GrayLevelNonUniformity_2	0.550
original_glrlm_GrayLevelNonUniformityNormalized_2	0.000
original_glrlm_GrayLevelVariance_2	0.000
original_glrlm_HighGrayLevelRunEmphasis_2	0.000
original_glrlm_LongRunEmphasis_2	0.096
original_glrlm_LongRunHighGrayLevelEmphasis_2	0.097
original_glrlm_LongRunLowGrayLevelEmphasis_2	0.096
original_glrlm_LowGrayLevelRunEmphasis_2	0.000
original_glrlm_RunEntropy_2	0.223
original_glrlm_RunLengthNonUniformity_2	0.557
original_glrlm_RunLengthNonUniformityNormalized_2	0.136
original_glrlm_RunPercentage_2	0.199
original_glrlm_RunVariance_2	0.160

original_glrlm_ShortRunEmphasis_2	0.146
original_glrlm_ShortRunHighGrayLevelEmphasis_2	0.002
original_glrlm_ShortRunLowGrayLevelEmphasis_2	0.167
original_glszm_GrayLevelNonUniformity_2	0.035
original_glszm_GrayLevelNonUniformityNormalized_2	0.000
original_glszm_GrayLevelVariance_2	0.000
original_glszm_HighGrayLevelZoneEmphasis_2	0.000
original_glszm_LargeAreaEmphasis_2	0.238
original_glszm_LargeAreaHighGrayLevelEmphasis_2	0.238
original_glszm_LargeAreaLowGrayLevelEmphasis_2	0.238
original_glszm_LowGrayLevelZoneEmphasis_2	0.000
original_glszm_SizeZoneNonUniformity_2	0.020
original_glszm_SizeZoneNonUniformityNormalized_2	0.126
original_glszm_SmallAreaEmphasis_2	0.530
original_glszm_SmallAreaHighGrayLevelEmphasis_2	0.525
original_glszm_SmallAreaLowGrayLevelEmphasis_2	0.532
original_glszm_ZoneEntropy_2	0.127
original_glszm_ZonePercentage_2	0.375
original_glszm_ZoneVariance_2	0.330
original_ngtdm_Busyness_2	0.000
original_ngtdm_Coarseness_2	0.000
original_ngtdm_Complexity_2	0.000
original_ngtdm_Contrast_2	0.000
original_ngtdm_Strength_2	0.000
exponential_firstorder_10Percentile_2	0.856
exponential_firstorder_90Percentile_2	0.876
exponential_firstorder_Energy_2	0.493
exponential_firstorder_Entropy_2	0.000
exponential_firstorder_InterquartileRange_2	0.121
exponential_firstorder_Kurtosis_2	0.421
exponential_firstorder_Maximum_2	0.985
exponential_firstorder_MeanAbsoluteDeviation_2	0.269
exponential_firstorder_Mean_2	0.912
exponential_firstorder_Median_2	0.929
exponential_firstorder_Minimum_2	0.375
exponential_firstorder_Range_2	0.934
exponential_firstorder_RobustMeanAbsoluteDeviation_2	0.146
exponential_firstorder_RootMeanSquared_2	0.911
exponential_firstorder_Skewness_2	0.572
exponential_firstorder_TotalEnergy_2	0.493
exponential_firstorder_Variance_2	0.159
exponential_glcm_DifferenceEntropy_2	0.000
exponential_glcm_JointEntropy_2	0.000
exponential_glcm_SumEntropy_2	0.000
exponential_gldm_DependenceEntropy_2	0.231
exponential_gldm_DependenceNonUniformity_2	0.195
exponential_gldm_DependenceNonUniformityNormalized_2	0.122
exponential_gldm_DependenceVariance_2	0.426
exponential_gldm_GrayLevelNonUniformity_2	0.509
exponential_gldm_LargeDependenceEmphasis_2	0.219

exponential_gldm_LargeDependenceHighGrayLevelEmphasis_2	0.219
exponential_gldm_LargeDependenceLowGrayLevelEmphasis_2	0.219
exponential_gldm_SmallDependenceEmphasis_2	0.220
exponential_gldm_SmallDependenceHighGrayLevelEmphasis_2	0.220
exponential_gldm_SmallDependenceLowGrayLevelEmphasis_2	0.220
exponential_glrlm_GrayLevelNonUniformity_2	0.550
exponential_glrlm_LongRunEmphasis_2	0.096
exponential_glrlm_LongRunHighGrayLevelEmphasis_2	0.096
exponential_glrlm_LongRunLowGrayLevelEmphasis_2	0.096
exponential_glrlm_RunEntropy_2	0.223
exponential_glrlm_RunLengthNonUniformity_2	0.557
exponential_glrlm_RunLengthNonUniformityNormalized_2	0.136
exponential_glrlm_RunPercentage_2	0.199
exponential_glrlm_RunVariance_2	0.160
exponential_glrlm_ShortRunEmphasis_2	0.146
exponential_glrlm_ShortRunHighGrayLevelEmphasis_2	0.146
exponential_glrlm_ShortRunLowGrayLevelEmphasis_2	0.146
exponential_glszm_GrayLevelNonUniformity_2	0.035
exponential_glszm_LargeAreaEmphasis_2	0.238
exponential_glszm_LargeAreaHighGrayLevelEmphasis_2	0.238
exponential_glszm_LargeAreaLowGrayLevelEmphasis_2	0.238
exponential_glszm_SizeZoneNonUniformity_2	0.021
exponential_glszm_SizeZoneNonUniformityNormalized_2	0.125
exponential_glszm_SmallAreaEmphasis_2	0.532
exponential_glszm_SmallAreaHighGrayLevelEmphasis_2	0.532
exponential_glszm_SmallAreaLowGrayLevelEmphasis_2	0.532
exponential_glszm_ZoneEntropy_2	0.124
exponential_glszm_ZonePercentage_2	0.375
exponential_glszm_ZoneVariance_2	0.330
gradient_firstorder_10Percentile_2	0.025
gradient_firstorder_90Percentile_2	0.133
gradient_firstorder_Energy_2	0.740
gradient_firstorder_Entropy_2	0.000
gradient_firstorder_InterquartileRange_2	0.156
gradient_firstorder_Kurtosis_2	0.319
gradient_firstorder_Maximum_2	0.797
gradient_firstorder_MeanAbsoluteDeviation_2	0.282
gradient_firstorder_Mean_2	0.001
gradient_firstorder_Median_2	0.059
gradient_firstorder_Minimum_2	0.067
gradient_firstorder_Range_2	0.805
gradient_firstorder_RobustMeanAbsoluteDeviation_2	0.195
gradient_firstorder_RootMeanSquared_2	0.088
gradient_firstorder_Skewness_2	0.368
gradient_firstorder_TotalEnergy_2	0.740
gradient_firstorder_Variance_2	0.287
gradient_glcm_DifferenceEntropy_2	0.000
gradient_glcm_JointEntropy_2	0.000
gradient_glcm_SumEntropy_2	0.000
gradient_gldm_DependenceEntropy_2	0.231

gradient_gldm_DependenceNonUniformity_2	0.195
gradient_gldm_DependenceNonUniformityNormalized_2	0.122
gradient_gldm_DependenceVariance_2	0.426
gradient_gldm_GrayLevelNonUniformity_2	0.509
gradient_gldm_LargeDependenceEmphasis_2	0.219
gradient_gldm_LargeDependenceHighGrayLevelEmphasis_2	0.219
gradient_gldm_LargeDependenceLowGrayLevelEmphasis_2	0.219
gradient_gldm_SmallDependenceEmphasis_2	0.220
gradient_gldm_SmallDependenceHighGrayLevelEmphasis_2	0.220
gradient_gldm_SmallDependenceLowGrayLevelEmphasis_2	0.220
gradient_glrlm_GrayLevelNonUniformity_2	0.550
gradient_glrlm_LongRunEmphasis_2	0.096
gradient_glrlm_LongRunHighGrayLevelEmphasis_2	0.096
gradient_glrlm_LongRunLowGrayLevelEmphasis_2	0.096
gradient_glrlm_RunEntropy_2	0.223
gradient_glrlm_RunLengthNonUniformity_2	0.557
gradient_glrlm_RunLengthNonUniformityNormalized_2	0.136
gradient_glrlm_RunPercentage_2	0.199
gradient_glrlm_RunVariance_2	0.160
gradient_glrlm_ShortRunEmphasis_2	0.146
gradient_glrlm_ShortRunHighGrayLevelEmphasis_2	0.146
gradient_glrlm_ShortRunLowGrayLevelEmphasis_2	0.146
gradient_glszm_GrayLevelNonUniformity_2	0.035
gradient_glszm_LargeAreaEmphasis_2	0.238
gradient_glszm_LargeAreaHighGrayLevelEmphasis_2	0.238
gradient_glszm_LargeAreaLowGrayLevelEmphasis_2	0.238
gradient_glszm_SizeZoneNonUniformity_2	0.021
gradient_glszm_SizeZoneNonUniformityNormalized_2	0.125
gradient_glszm_SmallAreaEmphasis_2	0.532
gradient_glszm_SmallAreaHighGrayLevelEmphasis_2	0.532
gradient_glszm_SmallAreaLowGrayLevelEmphasis_2	0.532
gradient_glszm_ZoneEntropy_2	0.124
gradient_glszm_ZonePercentage_2	0.375
gradient_glszm_ZoneVariance_2	0.330
lbp-2D_firstorder_10Percentile_2	0.021
lbp-2D_firstorder_90Percentile_2	0.122
lbp-2D_firstorder_Energy_2	0.492
lbp-2D_firstorder_Entropy_2	0.160
lbp-2D_firstorder_InterquartileRange_2	0.065
lbp-2D_firstorder_Kurtosis_2	0.012
lbp-2D_firstorder_Maximum_2	0.000
lbp-2D_firstorder_MeanAbsoluteDeviation_2	0.046
lbp-2D_firstorder_Mean_2	0.051
lbp-2D_firstorder_Median_2	0.103
lbp-2D_firstorder_Minimum_2	0.237
lbp-2D_firstorder_Range_2	0.145
lbp-2D_firstorder_RobustMeanAbsoluteDeviation_2	0.068
lbp-2D_firstorder_RootMeanSquared_2	0.076
lbp-2D_firstorder_Skewness_2	0.115
lbp-2D_firstorder_TotalEnergy_2	0.492

lbp-2D_firstorder_Uniformity_2	0.168
lbp-2D_firstorder_Variance_2	0.041
lbp-2D_glcm_Autocorrelation_2	0.087
lbp-2D_glcm_ClusterProminence_2	0.309
lbp-2D_glcm_ClusterShade_2	0.165
lbp-2D_glcm_ClusterTendency_2	0.273
lbp-2D_glcm_Contrast_2	0.154
lbp-2D_glcm_Correlation_2	0.283
lbp-2D_glcm_DifferenceAverage_2	0.154
lbp-2D_glcm_DifferenceEntropy_2	0.145
lbp-2D_glcm_DifferenceVariance_2	0.149
lbp-2D_glcm_Id_2	0.154
lbp-2D_glcm_Idm_2	0.154
lbp-2D_glcm_Idmn_2	0.154
lbp-2D_glcm_Idn_2	0.154
lbp-2D_glcm_Imc1_2	0.518
lbp-2D_glcm_Imc2_2	0.487
lbp-2D_glcm_InverseVariance_2	0.154
lbp-2D_glcm_JointAverage_2	0.082
lbp-2D_glcm_JointEnergy_2	0.084
lbp-2D_glcm_JointEntropy_2	0.102
lbp-2D_glcm_MCC_2	0.479
lbp-2D_glcm_MaximumProbability_2	0.143
lbp-2D_glcm_SumAverage_2	0.082
lbp-2D_glcm_SumEntropy_2	0.169
lbp-2D_glcm_SumSquares_2	0.118
lbp-2D_gldm_DependenceEntropy_2	0.550
lbp-2D_gldm_DependenceNonUniformity_2	0.503
lbp-2D_gldm_DependenceNonUniformityNormalized_2	0.616
lbp-2D_gldm_DependenceVariance_2	0.546
lbp-2D_gldm_GrayLevelNonUniformity_2	0.511
lbp-2D_gldm_GrayLevelVariance_2	0.168
lbp-2D_gldm_HighGrayLevelEmphasis_2	0.076
lbp-2D_gldm_LargeDependenceEmphasis_2	0.381
lbp-2D_gldm_LargeDependenceHighGrayLevelEmphasis_2	0.264
lbp-2D_gldm_LargeDependenceLowGrayLevelEmphasis_2	0.354
lbp-2D_gldm_LowGrayLevelEmphasis_2	0.076
lbp-2D_gldm_SmallDependenceEmphasis_2	0.346
lbp-2D_gldm_SmallDependenceHighGrayLevelEmphasis_2	0.395
lbp-2D_gldm_SmallDependenceLowGrayLevelEmphasis_2	0.280
lbp-2D_glrlm_GrayLevelNonUniformity_2	0.529
lbp-2D_glrlm_GrayLevelNonUniformityNormalized_2	0.168
lbp-2D_glrlm_GrayLevelVariance_2	0.168
lbp-2D_glrlm_HighGrayLevelRunEmphasis_2	0.059
lbp-2D_glrlm_LongRunEmphasis_2	0.662
lbp-2D_glrlm_LongRunHighGrayLevelEmphasis_2	0.466
lbp-2D_glrlm_LongRunLowGrayLevelEmphasis_2	0.599
lbp-2D_glrlm_LowGrayLevelRunEmphasis_2	0.059
lbp-2D_glrlm_RunEntropy_2	0.293
lbp-2D_glrlm_RunLengthNonUniformity_2	0.550

lbp-2D_glrlm_RunLengthNonUniformityNormalized_2	0.289
lbp-2D_glrlm_RunPercentage_2	0.337
lbp-2D_glrlm_RunVariance_2	0.622
lbp-2D_glrlm_ShortRunEmphasis_2	0.339
lbp-2D_glrlm_ShortRunHighGrayLevelEmphasis_2	0.235
lbp-2D_glrlm_ShortRunLowGrayLevelEmphasis_2	0.098
lbp-2D_glszm_GrayLevelNonUniformity_2	0.542
lbp-2D_glszm_GrayLevelNonUniformityNormalized_2	0.170
lbp-2D_glszm_GrayLevelVariance_2	0.170
lbp-2D_glszm_HighGrayLevelZoneEmphasis_2	0.065
lbp-2D_glszm_LargeAreaEmphasis_2	0.144
lbp-2D_glszm_LargeAreaHighGrayLevelEmphasis_2	0.130
lbp-2D_glszm_LargeAreaLowGrayLevelEmphasis_2	0.158
lbp-2D_glszm_LowGrayLevelZoneEmphasis_2	0.065
lbp-2D_glszm_SizeZoneNonUniformity_2	0.461
lbp-2D_glszm_SizeZoneNonUniformityNormalized_2	0.292
lbp-2D_glszm_SmallAreaEmphasis_2	0.352
lbp-2D_glszm_SmallAreaHighGrayLevelEmphasis_2	0.223
lbp-2D_glszm_SmallAreaLowGrayLevelEmphasis_2	0.124
lbp-2D_glszm_ZoneEntropy_2	0.152
lbp-2D_glszm_ZonePercentage_2	0.411
lbp-2D_glszm_ZoneVariance_2	0.150
lbp-2D_ngtdm_Busyness_2	0.163
lbp-2D_ngtdm_Coarseness_2	0.354
lbp-2D_ngtdm_Complexity_2	0.145
lbp-2D_ngtdm_Contrast_2	0.169
lbp-2D_ngtdm_Strength_2	0.362
lbp-3D-m1_firstorder_10Percentile_2	0.003
lbp-3D-m1_firstorder_90Percentile_2	0.249
lbp-3D-m1_firstorder_Energy_2	0.497
lbp-3D-m1_firstorder_Entropy_2	0.250
lbp-3D-m1_firstorder_InterquartileRange_2	0.183
lbp-3D-m1_firstorder_Kurtosis_2	0.002
lbp-3D-m1_firstorder_Maximum_2	0.721
lbp-3D-m1_firstorder_MeanAbsoluteDeviation_2	0.173
lbp-3D-m1_firstorder_Mean_2	0.054
lbp-3D-m1_firstorder_Median_2	0.064
lbp-3D-m1_firstorder_Minimum_2	0.478
lbp-3D-m1_firstorder_Range_2	0.652
lbp-3D-m1_firstorder_RobustMeanAbsoluteDeviation_2	0.137
lbp-3D-m1_firstorder_RootMeanSquared_2	0.116
lbp-3D-m1_firstorder_Skewness_2	0.177
lbp-3D-m1_firstorder_TotalEnergy_2	0.497
lbp-3D-m1_firstorder_Uniformity_2	0.193
lbp-3D-m1_firstorder_Variance_2	0.173
lbp-3D-m1_glcm_Autocorrelation_2	0.095
lbp-3D-m1_glcm_ClusterProminence_2	0.213
lbp-3D-m1_glcm_ClusterShade_2	0.194
lbp-3D-m1_glcm_ClusterTendency_2	0.222
lbp-3D-m1_glcm_Contrast_2	0.141

lbp-3D-m1_glcm_Correlation_2	0.075
lbp-3D-m1_glcm_DifferenceAverage_2	0.131
lbp-3D-m1_glcm_DifferenceEntropy_2	0.221
lbp-3D-m1_glcm_DifferenceVariance_2	0.216
lbp-3D-m1_glcm_Id_2	0.131
lbp-3D-m1_glcm_Idm_2	0.131
lbp-3D-m1_glcm_Idmn_2	0.073
lbp-3D-m1_glcm_Idn_2	0.071
lbp-3D-m1_glcm_Imc1_2	0.218
lbp-3D-m1_glcm_Imc2_2	0.216
lbp-3D-m1_glcm_InverseVariance_2	0.293
lbp-3D-m1_glcm_JointAverage_2	0.108
lbp-3D-m1_glcm_JointEnergy_2	0.244
lbp-3D-m1_glcm_JointEntropy_2	0.260
lbp-3D-m1_glcm_MCC_2	0.119
lbp-3D-m1_glcm_MaximumProbability_2	0.155
lbp-3D-m1_glcm_SumAverage_2	0.108
lbp-3D-m1_glcm_SumEntropy_2	0.314
lbp-3D-m1_glcm_SumSquares_2	0.180
lbp-3D-m1_gldm_DependenceEntropy_2	0.477
lbp-3D-m1_gldm_DependenceNonUniformity_2	0.496
lbp-3D-m1_gldm_DependenceNonUniformityNormalized_2	0.499
lbp-3D-m1_gldm_DependenceVariance_2	0.404
lbp-3D-m1_gldm_GrayLevelNonUniformity_2	0.553
lbp-3D-m1_gldm_GrayLevelVariance_2	0.189
lbp-3D-m1_gldm_HighGrayLevelEmphasis_2	0.110
lbp-3D-m1_gldm_LargeDependenceEmphasis_2	0.435
lbp-3D-m1_gldm_LargeDependenceHighGrayLevelEmphasis_2	0.418
lbp-3D-m1_gldm_LargeDependenceLowGrayLevelEmphasis_2	0.203
lbp-3D-m1_gldm_LowGrayLevelEmphasis_2	0.011
lbp-3D-m1_gldm_SmallDependenceEmphasis_2	0.336
lbp-3D-m1_gldm_SmallDependenceHighGrayLevelEmphasis_2	0.247
lbp-3D-m1_gldm_SmallDependenceLowGrayLevelEmphasis_2	0.446
lbp-3D-m1_glrlm_GrayLevelNonUniformity_2	0.546
lbp-3D-m1_glrlm_GrayLevelNonUniformityNormalized_2	0.223
lbp-3D-m1_glrlm_GrayLevelVariance_2	0.208
lbp-3D-m1_glrlm_HighGrayLevelRunEmphasis_2	0.128
lbp-3D-m1_glrlm_LongRunEmphasis_2	0.730
lbp-3D-m1_glrlm_LongRunHighGrayLevelEmphasis_2	0.566
lbp-3D-m1_glrlm_LongRunLowGrayLevelEmphasis_2	0.115
lbp-3D-m1_glrlm_LowGrayLevelRunEmphasis_2	0.013
lbp-3D-m1_glrlm_RunEntropy_2	0.359
lbp-3D-m1_glrlm_RunLengthNonUniformity_2	0.509
lbp-3D-m1_glrlm_RunLengthNonUniformityNormalized_2	0.435
lbp-3D-m1_glrlm_RunPercentage_2	0.484
lbp-3D-m1_glrlm_RunVariance_2	0.718
lbp-3D-m1_glrlm_ShortRunEmphasis_2	0.480
lbp-3D-m1_glrlm_ShortRunHighGrayLevelEmphasis_2	0.125
lbp-3D-m1_glrlm_ShortRunLowGrayLevelEmphasis_2	0.030
lbp-3D-m1_glszm_GrayLevelNonUniformity_2	0.579

lbp-3D-m1_glszm_GrayLevelNonUniformityNormalized_2	0.443
lbp-3D-m1_glszm_GrayLevelVariance_2	0.114
lbp-3D-m1_glszm_HighGrayLevelZoneEmphasis_2	0.262
lbp-3D-m1_glszm_LargeAreaEmphasis_2	0.417
lbp-3D-m1_glszm_LargeAreaHighGrayLevelEmphasis_2	0.408
lbp-3D-m1_glszm_LargeAreaLowGrayLevelEmphasis_2	0.369
lbp-3D-m1_glszm_LowGrayLevelZoneEmphasis_2	0.152
lbp-3D-m1_glszm_SizeZoneNonUniformity_2	0.680
lbp-3D-m1_glszm_SizeZoneNonUniformityNormalized_2	0.717
lbp-3D-m1_glszm_SmallAreaEmphasis_2	0.722
lbp-3D-m1_glszm_SmallAreaHighGrayLevelEmphasis_2	0.497
lbp-3D-m1_glszm_SmallAreaLowGrayLevelEmphasis_2	0.283
lbp-3D-m1_glszm_ZoneEntropy_2	0.793
lbp-3D-m1_glszm_ZonePercentage_2	0.260
lbp-3D-m1_glszm_ZoneVariance_2	0.417
lbp-3D-m1_ngtdm_Busyness_2	0.425
lbp-3D-m1_ngtdm_Coarseness_2	0.543
lbp-3D-m1_ngtdm_Complexity_2	0.146
lbp-3D-m1_ngtdm_Contrast_2	0.094
lbp-3D-m1_ngtdm_Strength_2	0.396
lbp-3D-m2_firstorder_10Percentile_2	0.049
lbp-3D-m2_firstorder_90Percentile_2	0.052
lbp-3D-m2_firstorder_Energy_2	0.528
lbp-3D-m2_firstorder_Entropy_2	0.111
lbp-3D-m2_firstorder_InterquartileRange_2	0.015
lbp-3D-m2_firstorder_Kurtosis_2	0.102
lbp-3D-m2_firstorder_Maximum_2	0.041
lbp-3D-m2_firstorder_MeanAbsoluteDeviation_2	0.019
lbp-3D-m2_firstorder_Mean_2	0.158
lbp-3D-m2_firstorder_Median_2	0.237
lbp-3D-m2_firstorder_Minimum_2	0.470
lbp-3D-m2_firstorder_Range_2	0.398
lbp-3D-m2_firstorder_RobustMeanAbsoluteDeviation_2	0.027
lbp-3D-m2_firstorder_RootMeanSquared_2	0.182
lbp-3D-m2_firstorder_Skewness_2	0.373
lbp-3D-m2_firstorder_TotalEnergy_2	0.528
lbp-3D-m2_firstorder_Uniformity_2	0.167
lbp-3D-m2_firstorder_Variance_2	0.025
lbp-3D-m2_glcm_Autocorrelation_2	0.111
lbp-3D-m2_glcm_ClusterProminence_2	0.108
lbp-3D-m2_glcm_ClusterShade_2	0.335
lbp-3D-m2_glcm_ClusterTendency_2	0.139
lbp-3D-m2_glcm_Contrast_2	0.156
lbp-3D-m2_glcm_Correlation_2	0.399
lbp-3D-m2_glcm_DifferenceAverage_2	0.225
lbp-3D-m2_glcm_DifferenceEntropy_2	0.365
lbp-3D-m2_glcm_DifferenceVariance_2	0.234
lbp-3D-m2_glcm_Id_2	0.298
lbp-3D-m2_glcm_Idm_2	0.269
lbp-3D-m2_glcm_Idmn_2	0.089

lbp-3D-m2_glcm_Idn_2	0.217
lbp-3D-m2_glcm_Imc1_2	0.656
lbp-3D-m2_glcm_Imc2_2	0.350
lbp-3D-m2_glcm_InverseVariance_2	0.609
lbp-3D-m2_glcm_JointAverage_2	0.095
lbp-3D-m2_glcm_JointEnergy_2	0.658
lbp-3D-m2_glcm_JointEntropy_2	0.352
lbp-3D-m2_glcm_MCC_2	0.431
lbp-3D-m2_glcm_MaximumProbability_2	0.517
lbp-3D-m2_glcm_SumAverage_2	0.095
lbp-3D-m2_glcm_SumEntropy_2	0.466
lbp-3D-m2_glcm_SumSquares_2	0.146
lbp-3D-m2_gldm_DependenceEntropy_2	0.512
lbp-3D-m2_gldm_DependenceNonUniformity_2	0.498
lbp-3D-m2_gldm_DependenceNonUniformityNormalized_2	0.561
lbp-3D-m2_gldm_DependenceVariance_2	0.592
lbp-3D-m2_gldm_GrayLevelNonUniformity_2	0.549
lbp-3D-m2_gldm_GrayLevelVariance_2	0.055
lbp-3D-m2_gldm_HighGrayLevelEmphasis_2	0.131
lbp-3D-m2_gldm_LargeDependenceEmphasis_2	0.592
lbp-3D-m2_gldm_LargeDependenceHighGrayLevelEmphasis_2	0.462
lbp-3D-m2_gldm_LargeDependenceLowGrayLevelEmphasis_2	0.465
lbp-3D-m2_gldm_LowGrayLevelEmphasis_2	0.017
lbp-3D-m2_gldm_SmallDependenceEmphasis_2	0.468
lbp-3D-m2_gldm_SmallDependenceHighGrayLevelEmphasis_2	0.307
lbp-3D-m2_gldm_SmallDependenceLowGrayLevelEmphasis_2	0.609
lbp-3D-m2_glrlm_GrayLevelNonUniformity_2	0.548
lbp-3D-m2_glrlm_GrayLevelNonUniformityNormalized_2	0.093
lbp-3D-m2_glrlm_GrayLevelVariance_2	0.040
lbp-3D-m2_glrlm_HighGrayLevelRunEmphasis_2	0.131
lbp-3D-m2_glrlm_LongRunEmphasis_2	0.807
lbp-3D-m2_glrlm_LongRunHighGrayLevelEmphasis_2	0.391
lbp-3D-m2_glrlm_LongRunLowGrayLevelEmphasis_2	0.264
lbp-3D-m2_glrlm_LowGrayLevelRunEmphasis_2	0.015
lbp-3D-m2_glrlm_RunEntropy_2	0.264
lbp-3D-m2_glrlm_RunLengthNonUniformity_2	0.511
lbp-3D-m2_glrlm_RunLengthNonUniformityNormalized_2	0.545
lbp-3D-m2_glrlm_RunPercentage_2	0.607
lbp-3D-m2_glrlm_RunVariance_2	0.793
lbp-3D-m2_glrlm_ShortRunEmphasis_2	0.582
lbp-3D-m2_glrlm_ShortRunHighGrayLevelEmphasis_2	0.171
lbp-3D-m2_glrlm_ShortRunLowGrayLevelEmphasis_2	0.017
lbp-3D-m2_glszm_GrayLevelNonUniformity_2	0.670
lbp-3D-m2_glszm_GrayLevelNonUniformityNormalized_2	0.211
lbp-3D-m2_glszm_GrayLevelVariance_2	0.036
lbp-3D-m2_glszm_HighGrayLevelZoneEmphasis_2	0.162
lbp-3D-m2_glszm_LargeAreaEmphasis_2	0.354
lbp-3D-m2_glszm_LargeAreaHighGrayLevelEmphasis_2	0.316
lbp-3D-m2_glszm_LargeAreaLowGrayLevelEmphasis_2	0.378
lbp-3D-m2_glszm_LowGrayLevelZoneEmphasis_2	0.014

lbp-3D-m2_glszm_SizeZoneNonUniformity_2	0.619
lbp-3D-m2_glszm_SizeZoneNonUniformityNormalized_2	0.635
lbp-3D-m2_glszm_SmallAreaEmphasis_2	0.556
lbp-3D-m2_glszm_SmallAreaHighGrayLevelEmphasis_2	0.407
lbp-3D-m2_glszm_SmallAreaLowGrayLevelEmphasis_2	0.052
lbp-3D-m2_glszm_ZoneEntropy_2	0.805
lbp-3D-m2_glszm_ZonePercentage_2	0.344
lbp-3D-m2_glszm_ZoneVariance_2	0.354
lbp-3D-m2_ngtdm_Busyness_2	0.255
lbp-3D-m2_ngtdm_Coarseness_2	0.868
lbp-3D-m2_ngtdm_Complexity_2	0.347
lbp-3D-m2_ngtdm_Contrast_2	0.039
lbp-3D-m2_ngtdm_Strength_2	0.735
lbp-3D-k_firstorder_10Percentile_2	0.099
lbp-3D-k_firstorder_90Percentile_2	0.471
lbp-3D-k_firstorder_Energy_2	0.539
lbp-3D-k_firstorder_Entropy_2	0.464
lbp-3D-k_firstorder_InterquartileRange_2	0.510
lbp-3D-k_firstorder_Kurtosis_2	0.489
lbp-3D-k_firstorder_Maximum_2	0.563
lbp-3D-k_firstorder_MeanAbsoluteDeviation_2	0.602
lbp-3D-k_firstorder_Mean_2	0.320
lbp-3D-k_firstorder_Median_2	0.184
lbp-3D-k_firstorder_Minimum_2	0.598
lbp-3D-k_firstorder_Range_2	0.563
lbp-3D-k_firstorder_RobustMeanAbsoluteDeviation_2	0.533
lbp-3D-k_firstorder_RootMeanSquared_2	0.225
lbp-3D-k_firstorder_Skewness_2	0.378
lbp-3D-k_firstorder_TotalEnergy_2	0.539
lbp-3D-k_firstorder_Uniformity_2	0.441
lbp-3D-k_firstorder_Variance_2	0.597
lbp-3D-k_glcm_Autocorrelation_2	0.426
lbp-3D-k_glcm_ClusterProminence_2	0.520
lbp-3D-k_glcm_ClusterShade_2	0.511
lbp-3D-k_glcm_ClusterTendency_2	0.449
lbp-3D-k_glcm_Contrast_2	0.380
lbp-3D-k_glcm_Correlation_2	0.930
lbp-3D-k_glcm_DifferenceAverage_2	0.380
lbp-3D-k_glcm_DifferenceEntropy_2	0.412
lbp-3D-k_glcm_DifferenceVariance_2	0.380
lbp-3D-k_glcm_Id_2	0.380
lbp-3D-k_glcm_Idm_2	0.380
lbp-3D-k_glcm_Idmn_2	0.379
lbp-3D-k_glcm_Idn_2	0.382
lbp-3D-k_glcm_Imc1_2	0.288
lbp-3D-k_glcm_Imc2_2	0.586
lbp-3D-k_glcm_InverseVariance_2	0.381
lbp-3D-k_glcm_JointAverage_2	0.418
lbp-3D-k_glcm_JointEnergy_2	0.406
lbp-3D-k_glcm_JointEntropy_2	0.422

lbp-3D-k_glcm_MCC_2	0.931
lbp-3D-k_glcm_MaximumProbability_2	0.404
lbp-3D-k_glcm_SumAverage_2	0.418
lbp-3D-k_glcm_SumEntropy_2	0.434
lbp-3D-k_glcm_SumSquares_2	0.421
lbp-3D-k_gldm_DependenceEntropy_2	0.329
lbp-3D-k_gldm_DependenceNonUniformity_2	0.436
lbp-3D-k_gldm_DependenceNonUniformityNormalized_2	0.212
lbp-3D-k_gldm_DependenceVariance_2	0.322
lbp-3D-k_gldm_GrayLevelNonUniformity_2	0.512
lbp-3D-k_gldm_GrayLevelVariance_2	0.441
lbp-3D-k_gldm_HighGrayLevelEmphasis_2	0.434
lbp-3D-k_gldm_LargeDependenceEmphasis_2	0.370
lbp-3D-k_gldm_LargeDependenceHighGrayLevelEmphasis_2	0.348
lbp-3D-k_gldm_LargeDependenceLowGrayLevelEmphasis_2	0.376
lbp-3D-k_gldm_LowGrayLevelEmphasis_2	0.434
lbp-3D-k_gldm_SmallDependenceEmphasis_2	0.297
lbp-3D-k_gldm_SmallDependenceHighGrayLevelEmphasis_2	0.447
lbp-3D-k_gldm_SmallDependenceLowGrayLevelEmphasis_2	0.243
lbp-3D-k_glrlm_GrayLevelNonUniformity_2	0.572
lbp-3D-k_glrlm_GrayLevelNonUniformityNormalized_2	0.291
lbp-3D-k_glrlm_GrayLevelVariance_2	0.291
lbp-3D-k_glrlm_HighGrayLevelRunEmphasis_2	0.268
lbp-3D-k_glrlm_LongRunEmphasis_2	0.420
lbp-3D-k_glrlm_LongRunHighGrayLevelEmphasis_2	0.404
lbp-3D-k_glrlm_LongRunLowGrayLevelEmphasis_2	0.424
lbp-3D-k_glrlm_LowGrayLevelRunEmphasis_2	0.269
lbp-3D-k_glrlm_RunEntropy_2	0.273
lbp-3D-k_glrlm_RunLengthNonUniformity_2	0.578
lbp-3D-k_glrlm_RunLengthNonUniformityNormalized_2	0.283
lbp-3D-k_glrlm_RunPercentage_2	0.348
lbp-3D-k_glrlm_RunVariance_2	0.340
lbp-3D-k_glrlm_ShortRunEmphasis_2	0.429
lbp-3D-k_glrlm_ShortRunHighGrayLevelEmphasis_2	0.471
lbp-3D-k_glrlm_ShortRunLowGrayLevelEmphasis_2	0.224
lbp-3D-k_glszm_GrayLevelNonUniformity_2	0.527
lbp-3D-k_glszm_GrayLevelNonUniformityNormalized_2	0.097
lbp-3D-k_glszm_GrayLevelVariance_2	0.098
lbp-3D-k_glszm_HighGrayLevelZoneEmphasis_2	0.155
lbp-3D-k_glszm_LargeAreaEmphasis_2	0.423
lbp-3D-k_glszm_LargeAreaHighGrayLevelEmphasis_2	0.420
lbp-3D-k_glszm_LargeAreaLowGrayLevelEmphasis_2	0.424
lbp-3D-k_glszm_LowGrayLevelZoneEmphasis_2	0.156
lbp-3D-k_glszm_SizeZoneNonUniformity_2	0.652
lbp-3D-k_glszm_SizeZoneNonUniformityNormalized_2	0.451
lbp-3D-k_glszm_SmallAreaEmphasis_2	0.514
lbp-3D-k_glszm_SmallAreaHighGrayLevelEmphasis_2	0.332
lbp-3D-k_glszm_SmallAreaLowGrayLevelEmphasis_2	0.577
lbp-3D-k_glszm_ZoneEntropy_2	0.351
lbp-3D-k_glszm_ZonePercentage_2	0.310

lbp-3D-k_glszm_ZoneVariance_2	0.422
lbp-3D-k_ngtdm_Busyness_2	0.505
lbp-3D-k_ngtdm_Coarseness_2	1.000
lbp-3D-k_ngtdm_Complexity_2	0.228
lbp-3D-k_ngtdm_Contrast_2	0.358
lbp-3D-k_ngtdm_Strength_2	0.042
logarithm_firstorder_10Percentile_2	0.880
logarithm_firstorder_90Percentile_2	0.969
logarithm_firstorder_Energy_2	0.480
logarithm_firstorder_Entropy_2	0.000
logarithm_firstorder_InterquartileRange_2	0.025
logarithm_firstorder_Kurtosis_2	0.507
logarithm_firstorder_Maximum_2	0.983
logarithm_firstorder_MeanAbsoluteDeviation_2	0.166
logarithm_firstorder_Mean_2	0.954
logarithm_firstorder_Median_2	0.963
logarithm_firstorder_Minimum_2	0.299
logarithm_firstorder_Range_2	0.715
logarithm_firstorder_RobustMeanAbsoluteDeviation_2	0.043
logarithm_firstorder_RootMeanSquared_2	0.958
logarithm_firstorder_Skewness_2	0.589
logarithm_firstorder_TotalEnergy_2	0.480
logarithm_firstorder_Uniformity_2	0.000
logarithm_firstorder_Variance_2	0.196
logarithm_glcm_Autocorrelation_2	0.000
logarithm_glcm_ClusterProminence_2	0.000
logarithm_glcm_ClusterShade_2	0.000
logarithm_glcm_ClusterTendency_2	0.000
logarithm_glcm_Contrast_2	0.000
logarithm_glcm_Correlation_2	0.000
logarithm_glcm_DifferenceAverage_2	0.000
logarithm_glcm_DifferenceEntropy_2	0.000
logarithm_glcm_DifferenceVariance_2	0.000
logarithm_glcm_Id_2	0.000
logarithm_glcm_Idm_2	0.000
logarithm_glcm_Idmn_2	0.000
logarithm_glcm_Idn_2	0.000
logarithm_glcm_Imc1_2	0.000
logarithm_glcm_Imc2_2	0.000
logarithm_glcm_InverseVariance_2	0.000
logarithm_glcm_JointAverage_2	0.000
logarithm_glcm_JointEnergy_2	0.000
logarithm_glcm_JointEntropy_2	0.000
logarithm_glcm_MCC_2	0.000
logarithm_glcm_MaximumProbability_2	0.000
logarithm_glcm_SumAverage_2	0.000
logarithm_glcm_SumEntropy_2	0.000
logarithm_glcm_SumSquares_2	0.000
logarithm_gldm_DependenceEntropy_2	0.231
logarithm_gldm_DependenceNonUniformity_2	0.195

logarithm_gldm_DependenceNonUniformityNormalized_2	0.122
logarithm_gldm_DependenceVariance_2	0.426
logarithm_gldm_GrayLevelNonUniformity_2	0.509
logarithm_gldm_GrayLevelVariance_2	0.000
logarithm_gldm_HighGrayLevelEmphasis_2	0.000
logarithm_gldm_LargeDependenceEmphasis_2	0.219
logarithm_gldm_LargeDependenceHighGrayLevelEmphasis_2	0.220
logarithm_gldm_LargeDependenceLowGrayLevelEmphasis_2	0.194
logarithm_gldm_LowGrayLevelEmphasis_2	0.000
logarithm_gldm_SmallDependenceEmphasis_2	0.220
logarithm_gldm_SmallDependenceHighGrayLevelEmphasis_2	0.172
logarithm_gldm_SmallDependenceLowGrayLevelEmphasis_2	0.228
logarithm_glrlm_GrayLevelNonUniformity_2	0.550
logarithm_glrlm_GrayLevelNonUniformityNormalized_2	0.000
logarithm_glrlm_GrayLevelVariance_2	0.000
logarithm_glrlm_HighGrayLevelRunEmphasis_2	0.000
logarithm_glrlm_LongRunEmphasis_2	0.096
logarithm_glrlm_LongRunHighGrayLevelEmphasis_2	0.097
logarithm_glrlm_LongRunLowGrayLevelEmphasis_2	0.096
logarithm_glrlm_LowGrayLevelRunEmphasis_2	0.000
logarithm_glrlm_RunEntropy_2	0.223
logarithm_glrlm_RunLengthNonUniformity_2	0.557
logarithm_glrlm_RunLengthNonUniformityNormalized_2	0.136
logarithm_glrlm_RunPercentage_2	0.199
logarithm_glrlm_RunVariance_2	0.160
logarithm_glrlm_ShortRunEmphasis_2	0.146
logarithm_glrlm_ShortRunHighGrayLevelEmphasis_2	0.002
logarithm_glrlm_ShortRunLowGrayLevelEmphasis_2	0.167
logarithm_glszm_GrayLevelNonUniformity_2	0.035
logarithm_glszm_GrayLevelNonUniformityNormalized_2	0.000
logarithm_glszm_GrayLevelVariance_2	0.000
logarithm_glszm_HighGrayLevelZoneEmphasis_2	0.000
logarithm_glszm_LargeAreaEmphasis_2	0.238
logarithm_glszm_LargeAreaHighGrayLevelEmphasis_2	0.238
logarithm_glszm_LargeAreaLowGrayLevelEmphasis_2	0.238
logarithm_glszm_LowGrayLevelZoneEmphasis_2	0.000
logarithm_glszm_SizeZoneNonUniformity_2	0.020
logarithm_glszm_SizeZoneNonUniformityNormalized_2	0.126
logarithm_glszm_SmallAreaEmphasis_2	0.530
logarithm_glszm_SmallAreaHighGrayLevelEmphasis_2	0.525
logarithm_glszm_SmallAreaLowGrayLevelEmphasis_2	0.532
logarithm_glszm_ZoneEntropy_2	0.127
logarithm_glszm_ZonePercentage_2	0.375
logarithm_glszm_ZoneVariance_2	0.330
logarithm_ngtdm_Busyness_2	0.000
logarithm_ngtdm_Coarseness_2	0.000
logarithm_ngtdm_Complexity_2	0.000
logarithm_ngtdm_Contrast_2	0.000
logarithm_ngtdm_Strength_2	0.000
square_firstorder_10Percentile_2	0.360

square_firstorder_90Percentile_2	0.467
square_firstorder_Energy_2	0.460
square_firstorder_Entropy_2	0.000
square_firstorder_InterquartileRange_2	0.064
square_firstorder_Kurtosis_2	0.393
square_firstorder_Maximum_2	0.930
square_firstorder_MeanAbsoluteDeviation_2	0.139
square_firstorder_Mean_2	0.565
square_firstorder_Median_2	0.643
square_firstorder_Minimum_2	0.516
square_firstorder_Range_2	0.949
square_firstorder_RobustMeanAbsoluteDeviation_2	0.068
square_firstorder_RootMeanSquared_2	0.558
square_firstorder_Skewness_2	0.552
square_firstorder_TotalEnergy_2	0.460
square_firstorder_Variance_2	0.070
square_glcm_DifferenceEntropy_2	0.000
square_glcm_JointEntropy_2	0.000
square_glcm_SumEntropy_2	0.000
square_gldm_DependenceEntropy_2	0.231
square_gldm_DependenceNonUniformity_2	0.195
square_gldm_DependenceNonUniformityNormalized_2	0.122
square_gldm_DependenceVariance_2	0.426
square_gldm_GrayLevelNonUniformity_2	0.509
square_gldm_LargeDependenceEmphasis_2	0.219
square_gldm_LargeDependenceHighGrayLevelEmphasis_2	0.219
square_gldm_LargeDependenceLowGrayLevelEmphasis_2	0.219
square_gldm_SmallDependenceEmphasis_2	0.220
square_gldm_SmallDependenceHighGrayLevelEmphasis_2	0.220
square_gldm_SmallDependenceLowGrayLevelEmphasis_2	0.220
square_glrlm_GrayLevelNonUniformity_2	0.550
square_glrlm_LongRunEmphasis_2	0.096
square_glrlm_LongRunHighGrayLevelEmphasis_2	0.096
square_glrlm_LongRunLowGrayLevelEmphasis_2	0.096
square_glrlm_RunEntropy_2	0.223
square_glrlm_RunLengthNonUniformity_2	0.557
square_glrlm_RunLengthNonUniformityNormalized_2	0.136
square_glrlm_RunPercentage_2	0.199
square_glrlm_RunVariance_2	0.160
square_glrlm_ShortRunEmphasis_2	0.146
square_glrlm_ShortRunHighGrayLevelEmphasis_2	0.146
square_glrlm_ShortRunLowGrayLevelEmphasis_2	0.146
square_glszm_GrayLevelNonUniformity_2	0.035
square_glszm_LargeAreaEmphasis_2	0.238
square_glszm_LargeAreaHighGrayLevelEmphasis_2	0.238
square_glszm_LargeAreaLowGrayLevelEmphasis_2	0.238
square_glszm_SizeZoneNonUniformity_2	0.021
square_glszm_SizeZoneNonUniformityNormalized_2	0.125
square_glszm_SmallAreaEmphasis_2	0.532
square_glszm_SmallAreaHighGrayLevelEmphasis_2	0.532

square_glszm_SmallAreaLowGrayLevelEmphasis_2	0.532
square_glszm_ZoneEntropy_2	0.124
square_glszm_ZonePercentage_2	0.375
square_glszm_ZoneVariance_2	0.330
squareroot_firstorder_10Percentile_2	0.923
squareroot_firstorder_90Percentile_2	0.981
squareroot_firstorder_Energy_2	0.482
squareroot_firstorder_Entropy_2	0.000
squareroot_firstorder_InterquartileRange_2	0.036
squareroot_firstorder_Kurtosis_2	0.518
squareroot_firstorder_Maximum_2	0.984
squareroot_firstorder_MeanAbsoluteDeviation_2	0.178
squareroot_firstorder_Mean_2	0.970
squareroot_firstorder_Median_2	0.975
squareroot_firstorder_Minimum_2	0.364
squareroot_firstorder_Range_2	0.618
squareroot_firstorder_RobustMeanAbsoluteDeviation_2	0.055
squareroot_firstorder_RootMeanSquared_2	0.971
squareroot_firstorder_Skewness_2	0.593
squareroot_firstorder_TotalEnergy_2	0.482
squareroot_firstorder_Uniformity_2	0.000
squareroot_firstorder_Variance_2	0.197
squareroot_glcm_Autocorrelation_2	0.000
squareroot_glcm_ClusterProminence_2	0.000
squareroot_glcm_ClusterShade_2	0.000
squareroot_glcm_ClusterTendency_2	0.000
squareroot_glcm_Contrast_2	0.000
squareroot_glcm_Correlation_2	0.000
squareroot_glcm_DifferenceAverage_2	0.000
squareroot_glcm_DifferenceEntropy_2	0.000
squareroot_glcm_DifferenceVariance_2	0.000
squareroot_glcm_Id_2	0.000
squareroot_glcm_Idm_2	0.000
squareroot_glcm_Idmn_2	0.000
squareroot_glcm_Idn_2	0.000
squareroot_glcm_Imc1_2	0.000
squareroot_glcm_Imc2_2	0.000
squareroot_glcm_InverseVariance_2	0.000
squareroot_glcm_JointAverage_2	0.000
squareroot_glcm_JointEnergy_2	0.000
squareroot_glcm_JointEntropy_2	0.000
squareroot_glcm_MCC_2	0.000
squareroot_glcm_MaximumProbability_2	0.000
squareroot_glcm_SumAverage_2	0.000
squareroot_glcm_SumEntropy_2	0.000
squareroot_glcm_SumSquares_2	0.000
squareroot_gldm_DependenceEntropy_2	0.231
squareroot_gldm_DependenceNonUniformity_2	0.195
squareroot_gldm_DependenceNonUniformityNormalized_2	0.122
squareroot_gldm_DependenceVariance_2	0.426

squareroot_gldm_GrayLevelNonUniformity_2	0.509
squareroot_gldm_GrayLevelVariance_2	0.000
squareroot_gldm_HighGrayLevelEmphasis_2	0.000
squareroot_gldm_LargeDependenceEmphasis_2	0.219
squareroot_gldm_LargeDependenceHighGrayLevelEmphasis_2	0.220
squareroot_gldm_LargeDependenceLowGrayLevelEmphasis_2	0.194
squareroot_gldm_LowGrayLevelEmphasis_2	0.000
squareroot_gldm_SmallDependenceEmphasis_2	0.220
squareroot_gldm_SmallDependenceHighGrayLevelEmphasis_2	0.172
squareroot_gldm_SmallDependenceLowGrayLevelEmphasis_2	0.228
squareroot_glrlm_GrayLevelNonUniformity_2	0.550
squareroot_glrlm_GrayLevelNonUniformityNormalized_2	0.000
squareroot_glrlm_GrayLevelVariance_2	0.000
squareroot_glrlm_HighGrayLevelRunEmphasis_2	0.000
squareroot_glrlm_LongRunEmphasis_2	0.096
squareroot_glrlm_LongRunHighGrayLevelEmphasis_2	0.097
squareroot_glrlm_LongRunLowGrayLevelEmphasis_2	0.096
squareroot_glrlm_LowGrayLevelRunEmphasis_2	0.000
squareroot_glrlm_RunEntropy_2	0.223
squareroot_glrlm_RunLengthNonUniformity_2	0.557
squareroot_glrlm_RunLengthNonUniformityNormalized_2	0.136
squareroot_glrlm_RunPercentage_2	0.199
squareroot_glrlm_RunVariance_2	0.160
squareroot_glrlm_ShortRunEmphasis_2	0.146
squareroot_glrlm_ShortRunHighGrayLevelEmphasis_2	0.002
squareroot_glrlm_ShortRunLowGrayLevelEmphasis_2	0.167
squareroot_glszm_GrayLevelNonUniformity_2	0.035
squareroot_glszm_GrayLevelNonUniformityNormalized_2	0.000
squareroot_glszm_GrayLevelVariance_2	0.000
squareroot_glszm_HighGrayLevelZoneEmphasis_2	0.000
squareroot_glszm_LargeAreaEmphasis_2	0.238
squareroot_glszm_LargeAreaHighGrayLevelEmphasis_2	0.238
squareroot_glszm_LargeAreaLowGrayLevelEmphasis_2	0.238
squareroot_glszm_LowGrayLevelZoneEmphasis_2	0.000
squareroot_glszm_SizeZoneNonUniformity_2	0.020
squareroot_glszm_SizeZoneNonUniformityNormalized_2	0.126
squareroot_glszm_SmallAreaEmphasis_2	0.530
squareroot_glszm_SmallAreaHighGrayLevelEmphasis_2	0.525
squareroot_glszm_SmallAreaLowGrayLevelEmphasis_2	0.532
squareroot_glszm_ZoneEntropy_2	0.127
squareroot_glszm_ZonePercentage_2	0.375
squareroot_glszm_ZoneVariance_2	0.330
squareroot_ngtdm_Busyness_2	0.000
squareroot_ngtdm_Coarseness_2	0.000
squareroot_ngtdm_Complexity_2	0.000
squareroot_ngtdm_Contrast_2	0.000
squareroot_ngtdm_Strength_2	0.000
wavelet-LLH_firstorder_10Percentile_2	0.508
wavelet-LLH_firstorder_90Percentile_2	0.084
wavelet-LLH_firstorder_Energy_2	0.810

wavelet-LLH_firstorder_Entropy_2	0.590
wavelet-LLH_firstorder_InterquartileRange_2	0.119
wavelet-LLH_firstorder_Kurtosis_2	0.527
wavelet-LLH_firstorder_Maximum_2	0.656
wavelet-LLH_firstorder_MeanAbsoluteDeviation_2	0.342
wavelet-LLH_firstorder_Mean_2	0.349
wavelet-LLH_firstorder_Median_2	0.297
wavelet-LLH_firstorder_Minimum_2	0.914
wavelet-LLH_firstorder_Range_2	0.840
wavelet-LLH_firstorder_RobustMeanAbsoluteDeviation_2	0.173
wavelet-LLH_firstorder_RootMeanSquared_2	0.445
wavelet-LLH_firstorder_Skewness_2	0.688
wavelet-LLH_firstorder_TotalEnergy_2	0.810
wavelet-LLH_firstorder_Uniformity_2	0.581
wavelet-LLH_firstorder_Variance_2	0.303
wavelet-LLH_glcm_Autocorrelation_2	0.377
wavelet-LLH_glcm_ClusterProminence_2	0.654
wavelet-LLH_glcm_ClusterShade_2	0.288
wavelet-LLH_glcm_ClusterTendency_2	0.710
wavelet-LLH_glcm_Contrast_2	0.645
wavelet-LLH_glcm_Correlation_2	0.523
wavelet-LLH_glcm_DifferenceAverage_2	0.645
wavelet-LLH_glcm_DifferenceEntropy_2	0.712
wavelet-LLH_glcm_DifferenceVariance_2	0.686
wavelet-LLH_glcm_Id_2	0.645
wavelet-LLH_glcm_Idm_2	0.645
wavelet-LLH_glcm_Idmn_2	0.645
wavelet-LLH_glcm_Idn_2	0.645
wavelet-LLH_glcm_Imc1_2	0.469
wavelet-LLH_glcm_Imc2_2	0.597
wavelet-LLH_glcm_InverseVariance_2	0.645
wavelet-LLH_glcm_JointAverage_2	0.424
wavelet-LLH_glcm_JointEnergy_2	0.781
wavelet-LLH_glcm_JointEntropy_2	0.789
wavelet-LLH_glcm_MCC_2	0.561
wavelet-LLH_glcm_MaximumProbability_2	0.664
wavelet-LLH_glcm_SumAverage_2	0.424
wavelet-LLH_glcm_SumEntropy_2	0.808
wavelet-LLH_glcm_SumSquares_2	0.776
wavelet-LLH_gldm_DependenceEntropy_2	0.486
wavelet-LLH_gldm_DependenceNonUniformity_2	0.511
wavelet-LLH_gldm_DependenceNonUniformityNormalized_2	0.217
wavelet-LLH_gldm_DependenceVariance_2	0.585
wavelet-LLH_gldm_GrayLevelNonUniformity_2	0.510
wavelet-LLH_gldm_GrayLevelVariance_2	0.581
wavelet-LLH_gldm_HighGrayLevelEmphasis_2	0.352
wavelet-LLH_gldm_LargeDependenceEmphasis_2	0.411
wavelet-LLH_gldm_LargeDependenceHighGrayLevelEmphasis_2	0.584
wavelet-LLH_gldm_LargeDependenceLowGrayLevelEmphasis_2	0.216
wavelet-LLH_gldm_LowGrayLevelEmphasis_2	0.352

wavelet-LLH_gldm_SmallDependenceEmphasis_2	0.183
wavelet-LLH_gldm_SmallDependenceHighGrayLevelEmphasis_2	0.232
wavelet-LLH_gldm_SmallDependenceLowGrayLevelEmphasis_2	0.141
wavelet-LLH_glrlm_GrayLevelNonUniformity_2	0.555
wavelet-LLH_glrlm_GrayLevelNonUniformityNormalized_2	0.690
wavelet-LLH_glrlm_GrayLevelVariance_2	0.690
wavelet-LLH_glrlm_HighGrayLevelRunEmphasis_2	0.528
wavelet-LLH_glrlm_LongRunEmphasis_2	0.374
wavelet-LLH_glrlm_LongRunHighGrayLevelEmphasis_2	0.477
wavelet-LLH_glrlm_LongRunLowGrayLevelEmphasis_2	0.282
wavelet-LLH_glrlm_LowGrayLevelRunEmphasis_2	0.528
wavelet-LLH_glrlm_RunEntropy_2	0.367
wavelet-LLH_glrlm_RunLengthNonUniformity_2	0.605
wavelet-LLH_glrlm_RunLengthNonUniformityNormalized_2	0.260
wavelet-LLH_glrlm_RunPercentage_2	0.342
wavelet-LLH_glrlm_RunVariance_2	0.389
wavelet-LLH_glrlm_ShortRunEmphasis_2	0.298
wavelet-LLH_glrlm_ShortRunHighGrayLevelEmphasis_2	0.359
wavelet-LLH_glrlm_ShortRunLowGrayLevelEmphasis_2	0.421
wavelet-LLH_glszm_GrayLevelNonUniformity_2	0.486
wavelet-LLH_glszm_GrayLevelNonUniformityNormalized_2	0.050
wavelet-LLH_glszm_GrayLevelVariance_2	0.050
wavelet-LLH_glszm_HighGrayLevelZoneEmphasis_2	0.028
wavelet-LLH_glszm_LargeAreaEmphasis_2	0.255
wavelet-LLH_glszm_LargeAreaHighGrayLevelEmphasis_2	0.241
wavelet-LLH_glszm_LargeAreaLowGrayLevelEmphasis_2	0.263
wavelet-LLH_glszm_LowGrayLevelZoneEmphasis_2	0.028
wavelet-LLH_glszm_SizeZoneNonUniformity_2	0.378
wavelet-LLH_glszm_SizeZoneNonUniformityNormalized_2	0.480
wavelet-LLH_glszm_SmallAreaEmphasis_2	0.565
wavelet-LLH_glszm_SmallAreaHighGrayLevelEmphasis_2	0.317
wavelet-LLH_glszm_SmallAreaLowGrayLevelEmphasis_2	0.522
wavelet-LLH_glszm_ZoneEntropy_2	0.542
wavelet-LLH_glszm_ZonePercentage_2	0.226
wavelet-LLH_glszm_ZoneVariance_2	0.257
wavelet-LLH_ngtdm_Busyness_2	0.023
wavelet-LLH_ngtdm_Coarseness_2	0.966
wavelet-LLH_ngtdm_Complexity_2	0.650
wavelet-LLH_ngtdm_Contrast_2	0.660
wavelet-LLH_ngtdm_Strength_2	0.968
wavelet-LHL_firstorder_10Percentile_2	0.368
wavelet-LHL_firstorder_90Percentile_2	0.536
wavelet-LHL_firstorder_Energy_2	0.649
wavelet-LHL_firstorder_Entropy_2	0.345
wavelet-LHL_firstorder_InterquartileRange_2	0.552
wavelet-LHL_firstorder_Kurtosis_2	0.443
wavelet-LHL_firstorder_Maximum_2	0.826
wavelet-LHL_firstorder_MeanAbsoluteDeviation_2	0.504
wavelet-LHL_firstorder_Mean_2	0.100
wavelet-LHL_firstorder_Median_2	0.022

wavelet-LHL_firstorder_Minimum_2	0.870
wavelet-LHL_firstorder_Range_2	0.909
wavelet-LHL_firstorder_RobustMeanAbsoluteDeviation_2	0.524
wavelet-LHL_firstorder_RootMeanSquared_2	0.471
wavelet-LHL_firstorder_Skewness_2	0.165
wavelet-LHL_firstorder_TotalEnergy_2	0.649
wavelet-LHL_firstorder_Uniformity_2	0.346
wavelet-LHL_firstorder_Variance_2	0.233
wavelet-LHL_glcm_Autocorrelation_2	0.341
wavelet-LHL_glcm_ClusterProminence_2	0.183
wavelet-LHL_glcm_ClusterShade_2	0.323
wavelet-LHL_glcm_ClusterTendency_2	0.459
wavelet-LHL_glcm_Contrast_2	0.073
wavelet-LHL_glcm_Correlation_2	0.126
wavelet-LHL_glcm_DifferenceAverage_2	0.073
wavelet-LHL_glcm_DifferenceEntropy_2	0.220
wavelet-LHL_glcm_DifferenceVariance_2	0.251
wavelet-LHL_glcm_Id_2	0.073
wavelet-LHL_glcm_Idm_2	0.073
wavelet-LHL_glcm_Idmn_2	0.073
wavelet-LHL_glcm_Idn_2	0.073
wavelet-LHL_glcm_Imc1_2	0.430
wavelet-LHL_glcm_Imc2_2	0.518
wavelet-LHL_glcm_InverseVariance_2	0.073
wavelet-LHL_glcm_JointAverage_2	0.361
wavelet-LHL_glcm_JointEnergy_2	0.028
wavelet-LHL_glcm_JointEntropy_2	0.046
wavelet-LHL_glcm_MCC_2	0.510
wavelet-LHL_glcm_MaximumProbability_2	0.141
wavelet-LHL_glcm_SumAverage_2	0.361
wavelet-LHL_glcm_SumEntropy_2	0.328
wavelet-LHL_glcm_SumSquares_2	0.023
wavelet-LHL_gldm_DependenceEntropy_2	0.445
wavelet-LHL_gldm_DependenceNonUniformity_2	0.506
wavelet-LHL_gldm_DependenceNonUniformityNormalized_2	0.487
wavelet-LHL_gldm_DependenceVariance_2	0.469
wavelet-LHL_gldm_GrayLevelNonUniformity_2	0.511
wavelet-LHL_gldm_GrayLevelVariance_2	0.346
wavelet-LHL_gldm_HighGrayLevelEmphasis_2	0.388
wavelet-LHL_gldm_LargeDependenceEmphasis_2	0.322
wavelet-LHL_gldm_LargeDependenceHighGrayLevelEmphasis_2	0.313
wavelet-LHL_gldm_LargeDependenceLowGrayLevelEmphasis_2	0.305
wavelet-LHL_gldm_LowGrayLevelEmphasis_2	0.388
wavelet-LHL_gldm_SmallDependenceEmphasis_2	0.314
wavelet-LHL_gldm_SmallDependenceHighGrayLevelEmphasis_2	0.312
wavelet-LHL_gldm_SmallDependenceLowGrayLevelEmphasis_2	0.315
wavelet-LHL_glrlm_GrayLevelNonUniformity_2	0.531
wavelet-LHL_glrlm_GrayLevelNonUniformityNormalized_2	0.469
wavelet-LHL_glrlm_GrayLevelVariance_2	0.469
wavelet-LHL_glrlm_HighGrayLevelRunEmphasis_2	0.386

wavelet-LHL_glrlm_LongRunEmphasis_2	0.645
wavelet-LHL_glrlm_LongRunHighGrayLevelEmphasis_2	0.558
wavelet-LHL_glrlm_LongRunLowGrayLevelEmphasis_2	0.671
wavelet-LHL_glrlm_LowGrayLevelRunEmphasis_2	0.386
wavelet-LHL_glrlm_RunEntropy_2	0.281
wavelet-LHL_glrlm_RunLengthNonUniformity_2	0.546
wavelet-LHL_glrlm_RunLengthNonUniformityNormalized_2	0.241
wavelet-LHL_glrlm_RunPercentage_2	0.282
wavelet-LHL_glrlm_RunVariance_2	0.583
wavelet-LHL_glrlm_ShortRunEmphasis_2	0.305
wavelet-LHL_glrlm_ShortRunHighGrayLevelEmphasis_2	0.462
wavelet-LHL_glrlm_ShortRunLowGrayLevelEmphasis_2	0.284
wavelet-LHL_glszm_GrayLevelNonUniformity_2	0.365
wavelet-LHL_glszm_GrayLevelNonUniformityNormalized_2	0.030
wavelet-LHL_glszm_GrayLevelVariance_2	0.030
wavelet-LHL_glszm_HighGrayLevelZoneEmphasis_2	0.008
wavelet-LHL_glszm_LargeAreaEmphasis_2	0.311
wavelet-LHL_glszm_LargeAreaHighGrayLevelEmphasis_2	0.319
wavelet-LHL_glszm_LargeAreaLowGrayLevelEmphasis_2	0.304
wavelet-LHL_glszm_LowGrayLevelZoneEmphasis_2	0.008
wavelet-LHL_glszm_SizeZoneNonUniformity_2	0.391
wavelet-LHL_glszm_SizeZoneNonUniformityNormalized_2	0.583
wavelet-LHL_glszm_SmallAreaEmphasis_2	0.755
wavelet-LHL_glszm_SmallAreaHighGrayLevelEmphasis_2	0.598
wavelet-LHL_glszm_SmallAreaLowGrayLevelEmphasis_2	0.709
wavelet-LHL_glszm_ZoneEntropy_2	0.530
wavelet-LHL_glszm_ZonePercentage_2	0.292
wavelet-LHL_glszm_ZoneVariance_2	0.313
wavelet-LHL_ngtdm_Busyness_2	0.551
wavelet-LHL_ngtdm_Coarseness_2	0.672
wavelet-LHL_ngtdm_Complexity_2	0.201
wavelet-LHL_ngtdm_Contrast_2	0.200
wavelet-LHL_ngtdm_Strength_2	0.677
wavelet-LHH_firstorder_10Percentile_2	0.689
wavelet-LHH_firstorder_90Percentile_2	0.599
wavelet-LHH_firstorder_Energy_2	0.779
wavelet-LHH_firstorder_Entropy_2	0.714
wavelet-LHH_firstorder_InterquartileRange_2	0.702
wavelet-LHH_firstorder_Kurtosis_2	0.762
wavelet-LHH_firstorder_Maximum_2	0.840
wavelet-LHH_firstorder_MeanAbsoluteDeviation_2	0.641
wavelet-LHH_firstorder_Mean_2	0.499
wavelet-LHH_firstorder_Median_2	0.361
wavelet-LHH_firstorder_Minimum_2	0.870
wavelet-LHH_firstorder_Range_2	0.904
wavelet-LHH_firstorder_RobustMeanAbsoluteDeviation_2	0.690
wavelet-LHH_firstorder_RootMeanSquared_2	0.630
wavelet-LHH_firstorder_Skewness_2	0.459
wavelet-LHH_firstorder_TotalEnergy_2	0.779
wavelet-LHH_firstorder_Uniformity_2	0.713

wavelet-LHH_firstorder_Variance_2	0.415
wavelet-LHH_glcm_Autocorrelation_2	0.559
wavelet-LHH_glcm_ClusterProminence_2	0.731
wavelet-LHH_glcm_ClusterShade_2	0.423
wavelet-LHH_glcm_ClusterTendency_2	0.776
wavelet-LHH_glcm_Contrast_2	0.647
wavelet-LHH_glcm_Correlation_2	0.699
wavelet-LHH_glcm_DifferenceAverage_2	0.647
wavelet-LHH_glcm_DifferenceEntropy_2	0.919
wavelet-LHH_glcm_DifferenceVariance_2	0.919
wavelet-LHH_glcm_Id_2	0.647
wavelet-LHH_glcm_Idm_2	0.647
wavelet-LHH_glcm_Idmn_2	0.647
wavelet-LHH_glcm_Idn_2	0.647
wavelet-LHH_glcm_Imc1_2	0.909
wavelet-LHH_glcm_Imc2_2	0.881
wavelet-LHH_glcm_InverseVariance_2	0.647
wavelet-LHH_glcm_JointAverage_2	0.581
wavelet-LHH_glcm_JointEnergy_2	0.855
wavelet-LHH_glcm_JointEntropy_2	0.867
wavelet-LHH_glcm_MCC_2	0.890
wavelet-LHH_glcm_MaximumProbability_2	0.694
wavelet-LHH_glcm_SumAverage_2	0.581
wavelet-LHH_glcm_SumEntropy_2	0.810
wavelet-LHH_glcm_SumSquares_2	0.821
wavelet-LHH_gldm_DependenceEntropy_2	0.538
wavelet-LHH_gldm_DependenceNonUniformity_2	0.499
wavelet-LHH_gldm_DependenceNonUniformityNormalized_2	0.351
wavelet-LHH_gldm_DependenceVariance_2	0.729
wavelet-LHH_gldm_GrayLevelNonUniformity_2	0.509
wavelet-LHH_gldm_GrayLevelVariance_2	0.713
wavelet-LHH_gldm_HighGrayLevelEmphasis_2	0.497
wavelet-LHH_gldm_LargeDependenceEmphasis_2	0.328
wavelet-LHH_gldm_LargeDependenceHighGrayLevelEmphasis_2	0.330
wavelet-LHH_gldm_LargeDependenceLowGrayLevelEmphasis_2	0.315
wavelet-LHH_gldm_LowGrayLevelEmphasis_2	0.497
wavelet-LHH_gldm_SmallDependenceEmphasis_2	0.246
wavelet-LHH_gldm_SmallDependenceHighGrayLevelEmphasis_2	0.241
wavelet-LHH_gldm_SmallDependenceLowGrayLevelEmphasis_2	0.253
wavelet-LHH_glrlm_GrayLevelNonUniformity_2	0.532
wavelet-LHH_glrlm_GrayLevelNonUniformityNormalized_2	0.704
wavelet-LHH_glrlm_GrayLevelVariance_2	0.704
wavelet-LHH_glrlm_HighGrayLevelRunEmphasis_2	0.533
wavelet-LHH_glrlm_LongRunEmphasis_2	0.537
wavelet-LHH_glrlm_LongRunHighGrayLevelEmphasis_2	0.563
wavelet-LHH_glrlm_LongRunLowGrayLevelEmphasis_2	0.493
wavelet-LHH_glrlm_LowGrayLevelRunEmphasis_2	0.533
wavelet-LHH_glrlm_RunEntropy_2	0.284
wavelet-LHH_glrlm_RunLengthNonUniformity_2	0.555
wavelet-LHH_glrlm_RunLengthNonUniformityNormalized_2	0.228

wavelet-LHH_glrlm_RunPercentage_2	0.267
wavelet-LHH_glrlm_RunVariance_2	0.444
wavelet-LHH_glrlm_ShortRunEmphasis_2	0.291
wavelet-LHH_glrlm_ShortRunHighGrayLevelEmphasis_2	0.303
wavelet-LHH_glrlm_ShortRunLowGrayLevelEmphasis_2	0.331
wavelet-LHH_glszm_GrayLevelNonUniformity_2	0.359
wavelet-LHH_glszm_GrayLevelNonUniformityNormalized_2	0.017
wavelet-LHH_glszm_GrayLevelVariance_2	0.017
wavelet-LHH_glszm_HighGrayLevelZoneEmphasis_2	0.132
wavelet-LHH_glszm_LargeAreaEmphasis_2	0.139
wavelet-LHH_glszm_LargeAreaHighGrayLevelEmphasis_2	0.138
wavelet-LHH_glszm_LargeAreaLowGrayLevelEmphasis_2	0.140
wavelet-LHH_glszm_LowGrayLevelZoneEmphasis_2	0.132
wavelet-LHH_glszm_SizeZoneNonUniformity_2	0.339
wavelet-LHH_glszm_SizeZoneNonUniformityNormalized_2	0.636
wavelet-LHH_glszm_SmallAreaEmphasis_2	0.797
wavelet-LHH_glszm_SmallAreaHighGrayLevelEmphasis_2	0.747
wavelet-LHH_glszm_SmallAreaLowGrayLevelEmphasis_2	0.748
wavelet-LHH_glszm_ZoneEntropy_2	0.506
wavelet-LHH_glszm_ZonePercentage_2	0.289
wavelet-LHH_glszm_ZoneVariance_2	0.145
wavelet-LHH_ngtdm_Busyness_2	0.480
wavelet-LHH_ngtdm_Coarseness_2	0.769
wavelet-LHH_ngtdm_Complexity_2	0.676
wavelet-LHH_ngtdm_Contrast_2	0.666
wavelet-LHH_ngtdm_Strength_2	0.768
wavelet-HLL_firstorder_10Percentile_2	0.112
wavelet-HLL_firstorder_90Percentile_2	0.310
wavelet-HLL_firstorder_Energy_2	0.777
wavelet-HLL_firstorder_Entropy_2	0.066
wavelet-HLL_firstorder_InterquartileRange_2	0.263
wavelet-HLL_firstorder_Kurtosis_2	0.392
wavelet-HLL_firstorder_Maximum_2	0.553
wavelet-HLL_firstorder_MeanAbsoluteDeviation_2	0.318
wavelet-HLL_firstorder_Mean_2	0.035
wavelet-HLL_firstorder_Median_2	0.000
wavelet-HLL_firstorder_Minimum_2	0.833
wavelet-HLL_firstorder_Range_2	0.843
wavelet-HLL_firstorder_RobustMeanAbsoluteDeviation_2	0.279
wavelet-HLL_firstorder_RootMeanSquared_2	0.182
wavelet-HLL_firstorder_Skewness_2	0.401
wavelet-HLL_firstorder_TotalEnergy_2	0.777
wavelet-HLL_firstorder_Uniformity_2	0.105
wavelet-HLL_firstorder_Variance_2	0.211
wavelet-HLL_glcm_Autocorrelation_2	0.500
wavelet-HLL_glcm_ClusterProminence_2	0.212
wavelet-HLL_glcm_ClusterShade_2	0.618
wavelet-HLL_glcm_ClusterTendency_2	0.196
wavelet-HLL_glcm_Contrast_2	0.142
wavelet-HLL_glcm_Correlation_2	0.333

wavelet-HLL_glcm_DifferenceAverage_2	0.142
wavelet-HLL_glcm_DifferenceEntropy_2	0.082
wavelet-HLL_glcm_DifferenceVariance_2	0.100
wavelet-HLL_glcm_Id_2	0.142
wavelet-HLL_glcm_Idm_2	0.142
wavelet-HLL_glcm_Idmn_2	0.142
wavelet-HLL_glcm_Idn_2	0.142
wavelet-HLL_glcm_Imc1_2	0.693
wavelet-HLL_glcm_Imc2_2	0.478
wavelet-HLL_glcm_InverseVariance_2	0.142
wavelet-HLL_glcm_JointAverage_2	0.461
wavelet-HLL_glcm_JointEnergy_2	0.078
wavelet-HLL_glcm_JointEntropy_2	0.083
wavelet-HLL_glcm_MCC_2	0.497
wavelet-HLL_glcm_MaximumProbability_2	0.255
wavelet-HLL_glcm_SumAverage_2	0.461
wavelet-HLL_glcm_SumEntropy_2	0.114
wavelet-HLL_glcm_SumSquares_2	0.094
wavelet-HLL_gldm_DependenceEntropy_2	0.404
wavelet-HLL_gldm_DependenceNonUniformity_2	0.499
wavelet-HLL_gldm_DependenceNonUniformityNormalized_2	0.468
wavelet-HLL_gldm_DependenceVariance_2	0.593
wavelet-HLL_gldm_GrayLevelNonUniformity_2	0.509
wavelet-HLL_gldm_GrayLevelVariance_2	0.105
wavelet-HLL_gldm_HighGrayLevelEmphasis_2	0.411
wavelet-HLL_gldm_LargeDependenceEmphasis_2	0.361
wavelet-HLL_gldm_LargeDependenceHighGrayLevelEmphasis_2	0.425
wavelet-HLL_gldm_LargeDependenceLowGrayLevelEmphasis_2	0.352
wavelet-HLL_gldm_LowGrayLevelEmphasis_2	0.411
wavelet-HLL_gldm_SmallDependenceEmphasis_2	0.340
wavelet-HLL_gldm_SmallDependenceHighGrayLevelEmphasis_2	0.337
wavelet-HLL_gldm_SmallDependenceLowGrayLevelEmphasis_2	0.342
wavelet-HLL_glrlm_GrayLevelNonUniformity_2	0.527
wavelet-HLL_glrlm_GrayLevelNonUniformityNormalized_2	0.062
wavelet-HLL_glrlm_GrayLevelVariance_2	0.062
wavelet-HLL_glrlm_HighGrayLevelRunEmphasis_2	0.328
wavelet-HLL_glrlm_LongRunEmphasis_2	0.605
wavelet-HLL_glrlm_LongRunHighGrayLevelEmphasis_2	0.571
wavelet-HLL_glrlm_LongRunLowGrayLevelEmphasis_2	0.617
wavelet-HLL_glrlm_LowGrayLevelRunEmphasis_2	0.328
wavelet-HLL_glrlm_RunEntropy_2	0.274
wavelet-HLL_glrlm_RunLengthNonUniformity_2	0.540
wavelet-HLL_glrlm_RunLengthNonUniformityNormalized_2	0.269
wavelet-HLL_glrlm_RunPercentage_2	0.310
wavelet-HLL_glrlm_RunVariance_2	0.522
wavelet-HLL_glrlm_ShortRunEmphasis_2	0.310
wavelet-HLL_glrlm_ShortRunHighGrayLevelEmphasis_2	0.436
wavelet-HLL_glrlm_ShortRunLowGrayLevelEmphasis_2	0.207
wavelet-HLL_glszm_GrayLevelNonUniformity_2	0.318
wavelet-HLL_glszm_GrayLevelNonUniformityNormalized_2	0.024

wavelet-HLL_glszm_GrayLevelVariance_2	0.024
wavelet-HLL_glszm_HighGrayLevelZoneEmphasis_2	0.106
wavelet-HLL_glszm_LargeAreaEmphasis_2	0.171
wavelet-HLL_glszm_LargeAreaHighGrayLevelEmphasis_2	0.171
wavelet-HLL_glszm_LargeAreaLowGrayLevelEmphasis_2	0.171
wavelet-HLL_glszm_LowGrayLevelZoneEmphasis_2	0.106
wavelet-HLL_glszm_SizeZoneNonUniformity_2	0.341
wavelet-HLL_glszm_SizeZoneNonUniformityNormalized_2	0.560
wavelet-HLL_glszm_SmallAreaEmphasis_2	0.648
wavelet-HLL_glszm_SmallAreaHighGrayLevelEmphasis_2	0.530
wavelet-HLL_glszm_SmallAreaLowGrayLevelEmphasis_2	0.560
wavelet-HLL_glszm_ZoneEntropy_2	0.523
wavelet-HLL_glszm_ZonePercentage_2	0.381
wavelet-HLL_glszm_ZoneVariance_2	0.176
wavelet-HLL_ngtdm_Busyness_2	0.477
wavelet-HLL_ngtdm_Coarseness_2	0.021
wavelet-HLL_ngtdm_Complexity_2	0.149
wavelet-HLL_ngtdm_Contrast_2	0.233
wavelet-HLL_ngtdm_Strength_2	0.020
wavelet-HLH_firstorder_10Percentile_2	0.458
wavelet-HLH_firstorder_90Percentile_2	0.133
wavelet-HLH_firstorder_Energy_2	0.746
wavelet-HLH_firstorder_Entropy_2	0.038
wavelet-HLH_firstorder_InterquartileRange_2	0.177
wavelet-HLH_firstorder_Kurtosis_2	0.714
wavelet-HLH_firstorder_Maximum_2	0.826
wavelet-HLH_firstorder_MeanAbsoluteDeviation_2	0.270
wavelet-HLH_firstorder_Mean_2	0.006
wavelet-HLH_firstorder_Median_2	0.002
wavelet-HLH_firstorder_Minimum_2	0.836
wavelet-HLH_firstorder_Range_2	0.884
wavelet-HLH_firstorder_RobustMeanAbsoluteDeviation_2	0.192
wavelet-HLH_firstorder_RootMeanSquared_2	0.322
wavelet-HLH_firstorder_Skewness_2	0.463
wavelet-HLH_firstorder_TotalEnergy_2	0.746
wavelet-HLH_firstorder_Uniformity_2	0.040
wavelet-HLH_firstorder_Variance_2	0.123
wavelet-HLH_glcm_Autocorrelation_2	0.252
wavelet-HLH_glcm_ClusterProminence_2	0.552
wavelet-HLH_glcm_ClusterShade_2	0.343
wavelet-HLH_glcm_ClusterTendency_2	0.523
wavelet-HLH_glcm_Contrast_2	0.561
wavelet-HLH_glcm_Correlation_2	0.619
wavelet-HLH_glcm_DifferenceAverage_2	0.561
wavelet-HLH_glcm_DifferenceEntropy_2	0.619
wavelet-HLH_glcm_DifferenceVariance_2	0.624
wavelet-HLH_glcm_Id_2	0.561
wavelet-HLH_glcm_Idm_2	0.561
wavelet-HLH_glcm_Idmn_2	0.561
wavelet-HLH_glcm_Idn_2	0.561

wavelet-HLH_glcm_Imc1_2	0.645
wavelet-HLH_glcm_Imc2_2	0.721
wavelet-HLH_glcm_InverseVariance_2	0.561
wavelet-HLH_glcm_JointAverage_2	0.226
wavelet-HLH_glcm_JointEnergy_2	0.229
wavelet-HLH_glcm_JointEntropy_2	0.223
wavelet-HLH_glcm_MCC_2	0.724
wavelet-HLH_glcm_MaximumProbability_2	0.328
wavelet-HLH_glcm_SumAverage_2	0.226
wavelet-HLH_glcm_SumEntropy_2	0.075
wavelet-HLH_glcm_SumSquares_2	0.041
wavelet-HLH_gldm_DependenceEntropy_2	0.493
wavelet-HLH_gldm_DependenceNonUniformity_2	0.494
wavelet-HLH_gldm_DependenceNonUniformityNormalized_2	0.446
wavelet-HLH_gldm_DependenceVariance_2	0.759
wavelet-HLH_gldm_GrayLevelNonUniformity_2	0.509
wavelet-HLH_gldm_GrayLevelVariance_2	0.040
wavelet-HLH_gldm_HighGrayLevelEmphasis_2	0.160
wavelet-HLH_gldm_LargeDependenceEmphasis_2	0.392
wavelet-HLH_gldm_LargeDependenceHighGrayLevelEmphasis_2	0.438
wavelet-HLH_gldm_LargeDependenceLowGrayLevelEmphasis_2	0.334
wavelet-HLH_gldm_LowGrayLevelEmphasis_2	0.160
wavelet-HLH_gldm_SmallDependenceEmphasis_2	0.301
wavelet-HLH_gldm_SmallDependenceHighGrayLevelEmphasis_2	0.266
wavelet-HLH_gldm_SmallDependenceLowGrayLevelEmphasis_2	0.336
wavelet-HLH_glrlm_GrayLevelNonUniformity_2	0.531
wavelet-HLH_glrlm_GrayLevelNonUniformityNormalized_2	0.036
wavelet-HLH_glrlm_GrayLevelVariance_2	0.036
wavelet-HLH_glrlm_HighGrayLevelRunEmphasis_2	0.187
wavelet-HLH_glrlm_LongRunEmphasis_2	0.541
wavelet-HLH_glrlm_LongRunHighGrayLevelEmphasis_2	0.582
wavelet-HLH_glrlm_LongRunLowGrayLevelEmphasis_2	0.479
wavelet-HLH_glrlm_LowGrayLevelRunEmphasis_2	0.187
wavelet-HLH_glrlm_RunEntropy_2	0.309
wavelet-HLH_glrlm_RunLengthNonUniformity_2	0.553
wavelet-HLH_glrlm_RunLengthNonUniformityNormalized_2	0.264
wavelet-HLH_glrlm_RunPercentage_2	0.318
wavelet-HLH_glrlm_RunVariance_2	0.452
wavelet-HLH_glrlm_ShortRunEmphasis_2	0.319
wavelet-HLH_glrlm_ShortRunHighGrayLevelEmphasis_2	0.254
wavelet-HLH_glrlm_ShortRunLowGrayLevelEmphasis_2	0.373
wavelet-HLH_glszm_GrayLevelNonUniformity_2	0.347
wavelet-HLH_glszm_GrayLevelNonUniformityNormalized_2	0.049
wavelet-HLH_glszm_GrayLevelVariance_2	0.049
wavelet-HLH_glszm_HighGrayLevelZoneEmphasis_2	0.013
wavelet-HLH_glszm_LargeAreaEmphasis_2	0.165
wavelet-HLH_glszm_LargeAreaHighGrayLevelEmphasis_2	0.165
wavelet-HLH_glszm_LargeAreaLowGrayLevelEmphasis_2	0.164
wavelet-HLH_glszm_LowGrayLevelZoneEmphasis_2	0.013
wavelet-HLH_glszm_SizeZoneNonUniformity_2	0.305

wavelet-HLH_glszm_SizeZoneNonUniformityNormalized_2	0.470
wavelet-HLH_glszm_SmallAreaEmphasis_2	0.629
wavelet-HLH_glszm_SmallAreaHighGrayLevelEmphasis_2	0.524
wavelet-HLH_glszm_SmallAreaLowGrayLevelEmphasis_2	0.541
wavelet-HLH_glszm_ZoneEntropy_2	0.438
wavelet-HLH_glszm_ZonePercentage_2	0.317
wavelet-HLH_glszm_ZoneVariance_2	0.170
wavelet-HLH_ngtdm_Busyness_2	0.490
wavelet-HLH_ngtdm_Coarseness_2	0.582
wavelet-HLH_ngtdm_Complexity_2	0.427
wavelet-HLH_ngtdm_Contrast_2	0.386
wavelet-HLH_ngtdm_Strength_2	0.593
wavelet-HHL_firstorder_10Percentile_2	0.897
wavelet-HHL_firstorder_90Percentile_2	0.739
wavelet-HHL_firstorder_Energy_2	0.583
wavelet-HHL_firstorder_Entropy_2	0.281
wavelet-HHL_firstorder_InterquartileRange_2	0.894
wavelet-HHL_firstorder_Kurtosis_2	0.857
wavelet-HHL_firstorder_Maximum_2	0.754
wavelet-HHL_firstorder_MeanAbsoluteDeviation_2	0.803
wavelet-HHL_firstorder_Mean_2	0.039
wavelet-HHL_firstorder_Median_2	0.050
wavelet-HHL_firstorder_Minimum_2	0.836
wavelet-HHL_firstorder_Range_2	0.853
wavelet-HHL_firstorder_RobustMeanAbsoluteDeviation_2	0.883
wavelet-HHL_firstorder_RootMeanSquared_2	0.682
wavelet-HHL_firstorder_Skewness_2	0.355
wavelet-HHL_firstorder_TotalEnergy_2	0.583
wavelet-HHL_firstorder_Uniformity_2	0.282
wavelet-HHL_firstorder_Variance_2	0.609
wavelet-HHL_glcm_Autocorrelation_2	0.052
wavelet-HHL_glcm_ClusterProminence_2	0.446
wavelet-HHL_glcm_ClusterShade_2	0.082
wavelet-HHL_glcm_ClusterTendency_2	0.530
wavelet-HHL_glcm_Contrast_2	0.410
wavelet-HHL_glcm_Correlation_2	0.473
wavelet-HHL_glcm_DifferenceAverage_2	0.410
wavelet-HHL_glcm_DifferenceEntropy_2	0.520
wavelet-HHL_glcm_DifferenceVariance_2	0.483
wavelet-HHL_glcm_Id_2	0.410
wavelet-HHL_glcm_Idm_2	0.410
wavelet-HHL_glcm_Idmn_2	0.410
wavelet-HHL_glcm_Idn_2	0.410
wavelet-HHL_glcm_Imc1_2	0.402
wavelet-HHL_glcm_Imc2_2	0.479
wavelet-HHL_glcm_InverseVariance_2	0.410
wavelet-HHL_glcm_JointAverage_2	0.085
wavelet-HHL_glcm_JointEnergy_2	0.414
wavelet-HHL_glcm_JointEntropy_2	0.350
wavelet-HHL_glcm_MCC_2	0.480

wavelet-HHL_glcm_MaximumProbability_2	0.467
wavelet-HHL_glcm_SumAverage_2	0.085
wavelet-HHL_glcm_SumEntropy_2	0.387
wavelet-HHL_glcm_SumSquares_2	0.341
wavelet-HHL_gldm_DependenceEntropy_2	0.376
wavelet-HHL_gldm_DependenceNonUniformity_2	0.487
wavelet-HHL_gldm_DependenceNonUniformityNormalized_2	0.256
wavelet-HHL_gldm_DependenceVariance_2	0.512
wavelet-HHL_gldm_GrayLevelNonUniformity_2	0.509
wavelet-HHL_gldm_GrayLevelVariance_2	0.282
wavelet-HHL_gldm_HighGrayLevelEmphasis_2	0.164
wavelet-HHL_gldm_LargeDependenceEmphasis_2	0.255
wavelet-HHL_gldm_LargeDependenceHighGrayLevelEmphasis_2	0.244
wavelet-HHL_gldm_LargeDependenceLowGrayLevelEmphasis_2	0.265
wavelet-HHL_gldm_LowGrayLevelEmphasis_2	0.164
wavelet-HHL_gldm_SmallDependenceEmphasis_2	0.254
wavelet-HHL_gldm_SmallDependenceHighGrayLevelEmphasis_2	0.300
wavelet-HHL_gldm_SmallDependenceLowGrayLevelEmphasis_2	0.204
wavelet-HHL_glrlm_GrayLevelNonUniformity_2	0.531
wavelet-HHL_glrlm_GrayLevelNonUniformityNormalized_2	0.323
wavelet-HHL_glrlm_GrayLevelVariance_2	0.323
wavelet-HHL_glrlm_HighGrayLevelRunEmphasis_2	0.238
wavelet-HHL_glrlm_LongRunEmphasis_2	0.561
wavelet-HHL_glrlm_LongRunHighGrayLevelEmphasis_2	0.543
wavelet-HHL_glrlm_LongRunLowGrayLevelEmphasis_2	0.576
wavelet-HHL_glrlm_LowGrayLevelRunEmphasis_2	0.238
wavelet-HHL_glrlm_RunEntropy_2	0.242
wavelet-HHL_glrlm_RunLengthNonUniformity_2	0.555
wavelet-HHL_glrlm_RunLengthNonUniformityNormalized_2	0.200
wavelet-HHL_glrlm_RunPercentage_2	0.228
wavelet-HHL_glrlm_RunVariance_2	0.470
wavelet-HHL_glrlm_ShortRunEmphasis_2	0.247
wavelet-HHL_glrlm_ShortRunHighGrayLevelEmphasis_2	0.395
wavelet-HHL_glrlm_ShortRunLowGrayLevelEmphasis_2	0.090
wavelet-HHL_glszm_GrayLevelNonUniformity_2	0.288
wavelet-HHL_glszm_GrayLevelNonUniformityNormalized_2	0.020
wavelet-HHL_glszm_GrayLevelVariance_2	0.020
wavelet-HHL_glszm_HighGrayLevelZoneEmphasis_2	0.078
wavelet-HHL_glszm_LargeAreaEmphasis_2	0.103
wavelet-HHL_glszm_LargeAreaHighGrayLevelEmphasis_2	0.105
wavelet-HHL_glszm_LargeAreaLowGrayLevelEmphasis_2	0.101
wavelet-HHL_glszm_LowGrayLevelZoneEmphasis_2	0.078
wavelet-HHL_glszm_SizeZoneNonUniformity_2	0.301
wavelet-HHL_glszm_SizeZoneNonUniformityNormalized_2	0.575
wavelet-HHL_glszm_SmallAreaEmphasis_2	0.722
wavelet-HHL_glszm_SmallAreaHighGrayLevelEmphasis_2	0.564
wavelet-HHL_glszm_SmallAreaLowGrayLevelEmphasis_2	0.640
wavelet-HHL_glszm_ZoneEntropy_2	0.453
wavelet-HHL_glszm_ZonePercentage_2	0.278
wavelet-HHL_glszm_ZoneVariance_2	0.112

wavelet-HHL_ngtdm_Busyness_2	0.509
wavelet-HHL_ngtdm_Coarseness_2	0.732
wavelet-HHL_ngtdm_Complexity_2	0.474
wavelet-HHL_ngtdm_Contrast_2	0.422
wavelet-HHL_ngtdm_Strength_2	0.731
wavelet-HHH_firstorder_10Percentile_2	0.743
wavelet-HHH_firstorder_90Percentile_2	0.818
wavelet-HHH_firstorder_Energy_2	0.683
wavelet-HHH_firstorder_Entropy_2	0.455
wavelet-HHH_firstorder_InterquartileRange_2	0.869
wavelet-HHH_firstorder_Kurtosis_2	0.923
wavelet-HHH_firstorder_Maximum_2	0.908
wavelet-HHH_firstorder_MeanAbsoluteDeviation_2	0.747
wavelet-HHH_firstorder_Mean_2	0.305
wavelet-HHH_firstorder_Median_2	0.592
wavelet-HHH_firstorder_Minimum_2	0.890
wavelet-HHH_firstorder_Range_2	0.924
wavelet-HHH_firstorder_RobustMeanAbsoluteDeviation_2	0.862
wavelet-HHH_firstorder_RootMeanSquared_2	0.665
wavelet-HHH_firstorder_Skewness_2	0.300
wavelet-HHH_firstorder_TotalEnergy_2	0.683
wavelet-HHH_firstorder_Uniformity_2	0.456
wavelet-HHH_firstorder_Variance_2	0.394
wavelet-HHH_glcm_Autocorrelation_2	0.179
wavelet-HHH_glcm_ClusterProminence_2	0.347
wavelet-HHH_glcm_ClusterShade_2	0.242
wavelet-HHH_glcm_ClusterTendency_2	0.394
wavelet-HHH_glcm_Contrast_2	0.532
wavelet-HHH_glcm_Correlation_2	0.460
wavelet-HHH_glcm_DifferenceAverage_2	0.532
wavelet-HHH_glcm_DifferenceEntropy_2	0.926
wavelet-HHH_glcm_DifferenceVariance_2	0.918
wavelet-HHH_glcm_Id_2	0.532
wavelet-HHH_glcm_Idm_2	0.532
wavelet-HHH_glcm_Idmn_2	0.532
wavelet-HHH_glcm_Idn_2	0.532
wavelet-HHH_glcm_Imc1_2	0.874
wavelet-HHH_glcm_Imc2_2	0.766
wavelet-HHH_glcm_InverseVariance_2	0.532
wavelet-HHH_glcm_JointAverage_2	0.168
wavelet-HHH_glcm_JointEnergy_2	0.731
wavelet-HHH_glcm_JointEntropy_2	0.680
wavelet-HHH_glcm_MCC_2	0.786
wavelet-HHH_glcm_MaximumProbability_2	0.687
wavelet-HHH_glcm_SumAverage_2	0.168
wavelet-HHH_glcm_SumEntropy_2	0.290
wavelet-HHH_glcm_SumSquares_2	0.244
wavelet-HHH_gldm_DependenceEntropy_2	0.439
wavelet-HHH_gldm_DependenceNonUniformity_2	0.471
wavelet-HHH_gldm_DependenceNonUniformityNormalized_2	0.335

wavelet-HHH_gldm_DependenceVariance_2	0.579
wavelet-HHH_gldm_GrayLevelNonUniformity_2	0.509
wavelet-HHH_gldm_GrayLevelVariance_2	0.456
wavelet-HHH_gldm_HighGrayLevelEmphasis_2	0.440
wavelet-HHH_gldm_LargeDependenceEmphasis_2	0.261
wavelet-HHH_gldm_LargeDependenceHighGrayLevelEmphasis_2	0.259
wavelet-HHH_gldm_LargeDependenceLowGrayLevelEmphasis_2	0.263
wavelet-HHH_gldm_LowGrayLevelEmphasis_2	0.440
wavelet-HHH_gldm_SmallDependenceEmphasis_2	0.252
wavelet-HHH_gldm_SmallDependenceHighGrayLevelEmphasis_2	0.247
wavelet-HHH_gldm_SmallDependenceLowGrayLevelEmphasis_2	0.257
wavelet-HHH_glrlm_GrayLevelNonUniformity_2	0.531
wavelet-HHH_glrlm_GrayLevelNonUniformityNormalized_2	0.511
wavelet-HHH_glrlm_GrayLevelVariance_2	0.511
wavelet-HHH_glrlm_HighGrayLevelRunEmphasis_2	0.505
wavelet-HHH_glrlm_LongRunEmphasis_2	0.493
wavelet-HHH_glrlm_LongRunHighGrayLevelEmphasis_2	0.502
wavelet-HHH_glrlm_LongRunLowGrayLevelEmphasis_2	0.482
wavelet-HHH_glrlm_LowGrayLevelRunEmphasis_2	0.505
wavelet-HHH_glrlm_RunEntropy_2	0.243
wavelet-HHH_glrlm_RunLengthNonUniformity_2	0.556
wavelet-HHH_glrlm_RunLengthNonUniformityNormalized_2	0.201
wavelet-HHH_glrlm_RunPercentage_2	0.229
wavelet-HHH_glrlm_RunVariance_2	0.360
wavelet-HHH_glrlm_ShortRunEmphasis_2	0.252
wavelet-HHH_glrlm_ShortRunHighGrayLevelEmphasis_2	0.165
wavelet-HHH_glrlm_ShortRunLowGrayLevelEmphasis_2	0.371
wavelet-HHH_glszm_GrayLevelNonUniformity_2	0.308
wavelet-HHH_glszm_GrayLevelNonUniformityNormalized_2	0.021
wavelet-HHH_glszm_GrayLevelVariance_2	0.021
wavelet-HHH_glszm_HighGrayLevelZoneEmphasis_2	0.261
wavelet-HHH_glszm_LargeAreaEmphasis_2	0.075
wavelet-HHH_glszm_LargeAreaHighGrayLevelEmphasis_2	0.074
wavelet-HHH_glszm_LargeAreaLowGrayLevelEmphasis_2	0.075
wavelet-HHH_glszm_LowGrayLevelZoneEmphasis_2	0.261
wavelet-HHH_glszm_SizeZoneNonUniformity_2	0.295
wavelet-HHH_glszm_SizeZoneNonUniformityNormalized_2	0.516
wavelet-HHH_glszm_SmallAreaEmphasis_2	0.604
wavelet-HHH_glszm_SmallAreaHighGrayLevelEmphasis_2	0.575
wavelet-HHH_glszm_SmallAreaLowGrayLevelEmphasis_2	0.560
wavelet-HHH_glszm_ZoneEntropy_2	0.416
wavelet-HHH_glszm_ZonePercentage_2	0.292
wavelet-HHH_glszm_ZoneVariance_2	0.085
wavelet-HHH_ngtdm_Busyness_2	0.507
wavelet-HHH_ngtdm_Coarseness_2	0.743
wavelet-HHH_ngtdm_Complexity_2	0.684
wavelet-HHH_ngtdm_Contrast_2	0.675
wavelet-HHH_ngtdm_Strength_2	0.743
wavelet-LLL_firstorder_10Percentile_2	0.687
wavelet-LLL_firstorder_90Percentile_2	0.841

wavelet-LLL_firstorder_Energy_2	0.478
wavelet-LLL_firstorder_Entropy_2	0.088
wavelet-LLL_firstorder_InterquartileRange_2	0.025
wavelet-LLL_firstorder_Kurtosis_2	0.408
wavelet-LLL_firstorder_Maximum_2	0.982
wavelet-LLL_firstorder_MeanAbsoluteDeviation_2	0.178
wavelet-LLL_firstorder_Mean_2	0.852
wavelet-LLL_firstorder_Median_2	0.881
wavelet-LLL_firstorder_Minimum_2	0.213
wavelet-LLL_firstorder_Range_2	0.845
wavelet-LLL_firstorder_RobustMeanAbsoluteDeviation_2	0.053
wavelet-LLL_firstorder_RootMeanSquared_2	0.857
wavelet-LLL_firstorder_Skewness_2	0.600
wavelet-LLL_firstorder_TotalEnergy_2	0.478
wavelet-LLL_firstorder_Uniformity_2	0.038
wavelet-LLL_firstorder_Variance_2	0.193
wavelet-LLL_glcm_Autocorrelation_2	0.009
wavelet-LLL_glcm_ClusterProminence_2	0.153
wavelet-LLL_glcm_ClusterShade_2	0.361
wavelet-LLL_glcm_ClusterTendency_2	0.046
wavelet-LLL_glcm_Contrast_2	0.029
wavelet-LLL_glcm_Correlation_2	0.366
wavelet-LLL_glcm_DifferenceAverage_2	0.029
wavelet-LLL_glcm_DifferenceEntropy_2	0.080
wavelet-LLL_glcm_DifferenceVariance_2	0.036
wavelet-LLL_glcm_Id_2	0.029
wavelet-LLL_glcm_Idm_2	0.029
wavelet-LLL_glcm_Idmn_2	0.029
wavelet-LLL_glcm_Idn_2	0.029
wavelet-LLL_glcm_Imc1_2	0.882
wavelet-LLL_glcm_Imc2_2	0.669
wavelet-LLL_glcm_InverseVariance_2	0.029
wavelet-LLL_glcm_JointAverage_2	0.009
wavelet-LLL_glcm_JointEnergy_2	0.041
wavelet-LLL_glcm_JointEntropy_2	0.079
wavelet-LLL_glcm_MCC_2	0.216
wavelet-LLL_glcm_MaximumProbability_2	0.025
wavelet-LLL_glcm_SumAverage_2	0.009
wavelet-LLL_glcm_SumEntropy_2	0.086
wavelet-LLL_glcm_SumSquares_2	0.042
wavelet-LLL_gldm_DependenceEntropy_2	0.111
wavelet-LLL_gldm_DependenceNonUniformity_2	0.192
wavelet-LLL_gldm_DependenceNonUniformityNormalized_2	0.073
wavelet-LLL_gldm_DependenceVariance_2	0.427
wavelet-LLL_gldm_GrayLevelNonUniformity_2	0.508
wavelet-LLL_gldm_GrayLevelVariance_2	0.038
wavelet-LLL_gldm_HighGrayLevelEmphasis_2	0.008
wavelet-LLL_gldm_LargeDependenceEmphasis_2	0.174
wavelet-LLL_gldm_LargeDependenceHighGrayLevelEmphasis_2	0.269
wavelet-LLL_gldm_LargeDependenceLowGrayLevelEmphasis_2	0.111

wavelet-LLL_gldm_LowGrayLevelEmphasis_2	0.008
wavelet-LLL_gldm_SmallDependenceEmphasis_2	0.217
wavelet-LLL_gldm_SmallDependenceHighGrayLevelEmphasis_2	0.164
wavelet-LLL_gldm_SmallDependenceLowGrayLevelEmphasis_2	0.227
wavelet-LLL_glrlm_GrayLevelNonUniformity_2	0.550
wavelet-LLL_glrlm_GrayLevelNonUniformityNormalized_2	0.083
wavelet-LLL_glrlm_GrayLevelVariance_2	0.083
wavelet-LLL_glrlm_HighGrayLevelRunEmphasis_2	0.005
wavelet-LLL_glrlm_LongRunEmphasis_2	0.090
wavelet-LLL_glrlm_LongRunHighGrayLevelEmphasis_2	0.095
wavelet-LLL_glrlm_LongRunLowGrayLevelEmphasis_2	0.089
wavelet-LLL_glrlm_LowGrayLevelRunEmphasis_2	0.005
wavelet-LLL_glrlm_RunEntropy_2	0.238
wavelet-LLL_glrlm_RunLengthNonUniformity_2	0.558
wavelet-LLL_glrlm_RunLengthNonUniformityNormalized_2	0.121
wavelet-LLL_glrlm_RunPercentage_2	0.165
wavelet-LLL_glrlm_RunVariance_2	0.151
wavelet-LLL_glrlm_ShortRunEmphasis_2	0.107
wavelet-LLL_glrlm_ShortRunHighGrayLevelEmphasis_2	0.043
wavelet-LLL_glrlm_ShortRunLowGrayLevelEmphasis_2	0.142
wavelet-LLL_glszm_GrayLevelNonUniformity_2	0.035
wavelet-LLL_glszm_GrayLevelNonUniformityNormalized_2	0.367
wavelet-LLL_glszm_GrayLevelVariance_2	0.367
wavelet-LLL_glszm_HighGrayLevelZoneEmphasis_2	0.005
wavelet-LLL_glszm_LargeAreaEmphasis_2	0.238
wavelet-LLL_glszm_LargeAreaHighGrayLevelEmphasis_2	0.238
wavelet-LLL_glszm_LargeAreaLowGrayLevelEmphasis_2	0.238
wavelet-LLL_glszm_LowGrayLevelZoneEmphasis_2	0.005
wavelet-LLL_glszm_SizeZoneNonUniformity_2	0.020
wavelet-LLL_glszm_SizeZoneNonUniformityNormalized_2	0.129
wavelet-LLL_glszm_SmallAreaEmphasis_2	0.531
wavelet-LLL_glszm_SmallAreaHighGrayLevelEmphasis_2	0.526
wavelet-LLL_glszm_SmallAreaLowGrayLevelEmphasis_2	0.532
wavelet-LLL_glszm_ZoneEntropy_2	0.135
wavelet-LLL_glszm_ZonePercentage_2	0.375
wavelet-LLL_glszm_ZoneVariance_2	0.330
wavelet-LLL_ngtdm_Busyness_2	0.036
wavelet-LLL_ngtdm_Coarseness_2	0.746
wavelet-LLL_ngtdm_Complexity_2	0.025
wavelet-LLL_ngtdm_Contrast_2	0.000
wavelet-LLL_ngtdm_Strength_2	0.000
original_shape_Elongation_3	0.709
original_shape_Flatness_3	0.889
original_shape_LeastAxisLength_3	0.914
original_shape_MajorAxisLength_3	0.898
original_shape_Maximum2DDiameterColumn_3	0.974
original_shape_Maximum2DDiameterRow_3	0.914
original_shape_Maximum2DDiameterSlice_3	0.968
original_shape_Maximum3DDiameter_3	0.988
original_shape_MeshVolume_3	0.456

original_shape_MinorAxisLength_3	0.921
original_shape_Sphericity_3	0.592
original_shape_SurfaceArea_3	0.515
original_shape_SurfaceVolumeRatio_3	0.299
original_shape_VoxelVolume_3	0.473
original_firstorder_10Percentile_3	0.794
original_firstorder_90Percentile_3	0.970
original_firstorder_Energy_3	0.438
original_firstorder_Entropy_3	0.938
original_firstorder_InterquartileRange_3	0.691
original_firstorder_Kurtosis_3	0.833
original_firstorder_Maximum_3	0.962
original_firstorder_MeanAbsoluteDeviation_3	0.699
original_firstorder_Mean_3	0.877
original_firstorder_Median_3	0.879
original_firstorder_Minimum_3	0.662
original_firstorder_Range_3	0.851
original_firstorder_RobustMeanAbsoluteDeviation_3	0.707
original_firstorder_RootMeanSquared_3	0.888
original_firstorder_Skewness_3	0.794
original_firstorder_TotalEnergy_3	0.438
original_firstorder_Uniformity_3	0.905
original_firstorder_Variance_3	0.745
original_glcm_Autocorrelation_3	0.481
original_glcm_ClusterProminence_3	0.889
original_glcm_ClusterShade_3	0.929
original_glcm_ClusterTendency_3	0.916
original_glcm_Contrast_3	0.942
original_glcm_Correlation_3	0.855
original_glcm_DifferenceAverage_3	0.942
original_glcm_DifferenceEntropy_3	0.984
original_glcm_DifferenceVariance_3	0.973
original_glcm_Id_3	0.942
original_glcm_Idm_3	0.942
original_glcm_Idmn_3	0.942
original_glcm_Idn_3	0.942
original_glcm_Imc1_3	0.979
original_glcm_Imc2_3	0.958
original_glcm_InverseVariance_3	0.942
original_glcm_JointAverage_3	0.504
original_glcm_JointEnergy_3	0.950
original_glcm_JointEntropy_3	0.960
original_glcm_MCC_3	0.442
original_glcm_MaximumProbability_3	0.924
original_glcm_SumAverage_3	0.504
original_glcm_SumEntropy_3	0.962
original_glcm_SumSquares_3	0.931
original_gldm_DependenceEntropy_3	0.258
original_gldm_DependenceNonUniformity_3	0.427
original_gldm_DependenceNonUniformityNormalized_3	0.153

original_gldm_DependenceVariance_3	0.379
original_gldm_GrayLevelNonUniformity_3	0.473
original_gldm_GrayLevelVariance_3	0.905
original_gldm_HighGrayLevelEmphasis_3	0.528
original_gldm_LargeDependenceEmphasis_3	0.190
original_gldm_LargeDependenceHighGrayLevelEmphasis_3	0.292
original_gldm_LargeDependenceLowGrayLevelEmphasis_3	0.189
original_gldm_LowGrayLevelEmphasis_3	0.528
original_gldm_SmallDependenceEmphasis_3	0.279
original_gldm_SmallDependenceHighGrayLevelEmphasis_3	0.226
original_gldm_SmallDependenceLowGrayLevelEmphasis_3	0.317
original_glrlm_GrayLevelNonUniformity_3	0.528
original_glrlm_GrayLevelNonUniformityNormalized_3	0.923
original_glrlm_GrayLevelVariance_3	0.923
original_glrlm_HighGrayLevelRunEmphasis_3	0.514
original_glrlm_LongRunEmphasis_3	0.095
original_glrlm_LongRunHighGrayLevelEmphasis_3	0.160
original_glrlm_LongRunLowGrayLevelEmphasis_3	0.086
original_glrlm_LowGrayLevelRunEmphasis_3	0.514
original_glrlm_RunEntropy_3	0.155
original_glrlm_RunLengthNonUniformity_3	0.572
original_glrlm_RunLengthNonUniformityNormalized_3	0.240
original_glrlm_RunPercentage_3	0.230
original_glrlm_RunVariance_3	0.068
original_glrlm_ShortRunEmphasis_3	0.263
original_glrlm_ShortRunHighGrayLevelEmphasis_3	0.459
original_glrlm_ShortRunLowGrayLevelEmphasis_3	0.425
original_glszm_GrayLevelNonUniformity_3	0.559
original_glszm_GrayLevelNonUniformityNormalized_3	0.960
original_glszm_GrayLevelVariance_3	0.960
original_glszm_HighGrayLevelZoneEmphasis_3	0.331
original_glszm_LargeAreaEmphasis_3	0.311
original_glszm_LargeAreaHighGrayLevelEmphasis_3	0.311
original_glszm_LargeAreaLowGrayLevelEmphasis_3	0.311
original_glszm_LowGrayLevelZoneEmphasis_3	0.331
original_glszm_SizeZoneNonUniformity_3	0.511
original_glszm_SizeZoneNonUniformityNormalized_3	0.183
original_glszm_SmallAreaEmphasis_3	0.645
original_glszm_SmallAreaHighGrayLevelEmphasis_3	0.632
original_glszm_SmallAreaLowGrayLevelEmphasis_3	0.648
original_glszm_ZoneEntropy_3	0.345
original_glszm_ZonePercentage_3	0.156
original_glszm_ZoneVariance_3	0.327
original_ngtdm_Busyness_3	0.758
original_ngtdm_Coarseness_3	0.586
original_ngtdm_Complexity_3	0.904
original_ngtdm_Contrast_3	0.729
original_ngtdm_Strength_3	0.019
exponential_firstorder_10Percentile_3	0.833
exponential_firstorder_90Percentile_3	0.974

exponential_firstorder_Energy_3	0.459
exponential_firstorder_Entropy_3	0.000
exponential_firstorder_InterquartileRange_3	0.658
exponential_firstorder_Kurtosis_3	0.879
exponential_firstorder_Maximum_3	0.971
exponential_firstorder_MeanAbsoluteDeviation_3	0.653
exponential_firstorder_Mean_3	0.921
exponential_firstorder_Median_3	0.922
exponential_firstorder_Minimum_3	0.633
exponential_firstorder_Range_3	0.912
exponential_firstorder_RobustMeanAbsoluteDeviation_3	0.662
exponential_firstorder_RootMeanSquared_3	0.923
exponential_firstorder_Skewness_3	0.815
exponential_firstorder_TotalEnergy_3	0.459
exponential_firstorder_Variance_3	0.634
exponential_glcm_DifferenceEntropy_3	0.000
exponential_glcm_JointEntropy_3	0.000
exponential_glcm_SumEntropy_3	0.000
exponential_gldm_DependenceEntropy_3	0.298
exponential_gldm_DependenceNonUniformity_3	0.427
exponential_gldm_DependenceNonUniformityNormalized_3	0.167
exponential_gldm_DependenceVariance_3	0.350
exponential_gldm_GrayLevelNonUniformity_3	0.473
exponential_gldm_LargeDependenceEmphasis_3	0.171
exponential_gldm_LargeDependenceHighGrayLevelEmphasis_3	0.171
exponential_gldm_LargeDependenceLowGrayLevelEmphasis_3	0.171
exponential_gldm_SmallDependenceEmphasis_3	0.279
exponential_gldm_SmallDependenceHighGrayLevelEmphasis_3	0.279
exponential_gldm_SmallDependenceLowGrayLevelEmphasis_3	0.279
exponential_glrlm_GrayLevelNonUniformity_3	0.528
exponential_glrlm_LongRunEmphasis_3	0.092
exponential_glrlm_LongRunHighGrayLevelEmphasis_3	0.092
exponential_glrlm_LongRunLowGrayLevelEmphasis_3	0.092
exponential_glrlm_RunEntropy_3	0.179
exponential_glrlm_RunLengthNonUniformity_3	0.573
exponential_glrlm_RunLengthNonUniformityNormalized_3	0.213
exponential_glrlm_RunPercentage_3	0.207
exponential_glrlm_RunVariance_3	0.067
exponential_glrlm_ShortRunEmphasis_3	0.236
exponential_glrlm_ShortRunHighGrayLevelEmphasis_3	0.236
exponential_glrlm_ShortRunLowGrayLevelEmphasis_3	0.236
exponential_glszm_GrayLevelNonUniformity_3	0.557
exponential_glszm_LargeAreaEmphasis_3	0.311
exponential_glszm_LargeAreaHighGrayLevelEmphasis_3	0.311
exponential_glszm_LargeAreaLowGrayLevelEmphasis_3	0.311
exponential_glszm_SizeZoneNonUniformity_3	0.509
exponential_glszm_SizeZoneNonUniformityNormalized_3	0.169
exponential_glszm_SmallAreaEmphasis_3	0.663
exponential_glszm_SmallAreaHighGrayLevelEmphasis_3	0.663
exponential_glszm_SmallAreaLowGrayLevelEmphasis_3	0.663

exponential_glszm_ZoneEntropy_3	0.300
exponential_glszm_ZonePercentage_3	0.153
exponential_glszm_ZoneVariance_3	0.327
gradient_firstorder_10Percentile_3	0.623
gradient_firstorder_90Percentile_3	0.590
gradient_firstorder_Energy_3	0.860
gradient_firstorder_Entropy_3	0.000
gradient_firstorder_InterquartileRange_3	0.425
gradient_firstorder_Kurtosis_3	0.543
gradient_firstorder_Maximum_3	0.761
gradient_firstorder_MeanAbsoluteDeviation_3	0.462
gradient_firstorder_Mean_3	0.647
gradient_firstorder_Median_3	0.683
gradient_firstorder_Minimum_3	0.266
gradient_firstorder_Range_3	0.767
gradient_firstorder_RobustMeanAbsoluteDeviation_3	0.419
gradient_firstorder_RootMeanSquared_3	0.630
gradient_firstorder_Skewness_3	0.622
gradient_firstorder_TotalEnergy_3	0.860
gradient_firstorder_Variance_3	0.405
gradient_glcm_DifferenceEntropy_3	0.000
gradient_glcm_JointEntropy_3	0.000
gradient_glcm_SumEntropy_3	0.000
gradient_gldm_DependenceEntropy_3	0.298
gradient_gldm_DependenceNonUniformity_3	0.427
gradient_gldm_DependenceNonUniformityNormalized_3	0.167
gradient_gldm_DependenceVariance_3	0.350
gradient_gldm_GrayLevelNonUniformity_3	0.473
gradient_gldm_LargeDependenceEmphasis_3	0.171
gradient_gldm_LargeDependenceHighGrayLevelEmphasis_3	0.171
gradient_gldm_LargeDependenceLowGrayLevelEmphasis_3	0.171
gradient_gldm_SmallDependenceEmphasis_3	0.279
gradient_gldm_SmallDependenceHighGrayLevelEmphasis_3	0.279
gradient_gldm_SmallDependenceLowGrayLevelEmphasis_3	0.279
gradient_glrlm_GrayLevelNonUniformity_3	0.528
gradient_glrlm_LongRunEmphasis_3	0.092
gradient_glrlm_LongRunHighGrayLevelEmphasis_3	0.092
gradient_glrlm_LongRunLowGrayLevelEmphasis_3	0.092
gradient_glrlm_RunEntropy_3	0.179
gradient_glrlm_RunLengthNonUniformity_3	0.573
gradient_glrlm_RunLengthNonUniformityNormalized_3	0.213
gradient_glrlm_RunPercentage_3	0.207
gradient_glrlm_RunVariance_3	0.067
gradient_glrlm_ShortRunEmphasis_3	0.236
gradient_glrlm_ShortRunHighGrayLevelEmphasis_3	0.236
gradient_glrlm_ShortRunLowGrayLevelEmphasis_3	0.236
gradient_glszm_GrayLevelNonUniformity_3	0.557
gradient_glszm_LargeAreaEmphasis_3	0.311
gradient_glszm_LargeAreaHighGrayLevelEmphasis_3	0.311
gradient_glszm_LargeAreaLowGrayLevelEmphasis_3	0.311

gradient_glszm_SizeZoneNonUniformity_3	0.509
gradient_glszm_SizeZoneNonUniformityNormalized_3	0.169
gradient_glszm_SmallAreaEmphasis_3	0.663
gradient_glszm_SmallAreaHighGrayLevelEmphasis_3	0.663
gradient_glszm_SmallAreaLowGrayLevelEmphasis_3	0.663
gradient_glszm_ZoneEntropy_3	0.300
gradient_glszm_ZonePercentage_3	0.153
gradient_glszm_ZoneVariance_3	0.327
lbp-2D_firstorder_10Percentile_3	0.197
lbp-2D_firstorder_90Percentile_3	0.377
lbp-2D_firstorder_Energy_3	0.461
lbp-2D_firstorder_Entropy_3	0.252
lbp-2D_firstorder_InterquartileRange_3	0.212
lbp-2D_firstorder_Kurtosis_3	0.458
lbp-2D_firstorder_Maximum_3	0.000
lbp-2D_firstorder_MeanAbsoluteDeviation_3	0.284
lbp-2D_firstorder_Mean_3	0.345
lbp-2D_firstorder_Median_3	0.409
lbp-2D_firstorder_Minimum_3	0.441
lbp-2D_firstorder_Range_3	0.105
lbp-2D_firstorder_RobustMeanAbsoluteDeviation_3	0.220
lbp-2D_firstorder_RootMeanSquared_3	0.372
lbp-2D_firstorder_Skewness_3	0.484
lbp-2D_firstorder_TotalEnergy_3	0.461
lbp-2D_firstorder_Uniformity_3	0.313
lbp-2D_firstorder_Variance_3	0.227
lbp-2D_glcm_Autocorrelation_3	0.349
lbp-2D_glcm_ClusterProminence_3	0.143
lbp-2D_glcm_ClusterShade_3	0.424
lbp-2D_glcm_ClusterTendency_3	0.302
lbp-2D_glcm_Contrast_3	0.300
lbp-2D_glcm_Correlation_3	0.021
lbp-2D_glcm_DifferenceAverage_3	0.300
lbp-2D_glcm_DifferenceEntropy_3	0.149
lbp-2D_glcm_DifferenceVariance_3	0.212
lbp-2D_glcm_Id_3	0.300
lbp-2D_glcm_Idm_3	0.300
lbp-2D_glcm_Idmn_3	0.300
lbp-2D_glcm_Idn_3	0.300
lbp-2D_glcm_Imc1_3	0.660
lbp-2D_glcm_Imc2_3	0.465
lbp-2D_glcm_InverseVariance_3	0.300
lbp-2D_glcm_JointAverage_3	0.342
lbp-2D_glcm_JointEnergy_3	0.280
lbp-2D_glcm_JointEntropy_3	0.251
lbp-2D_glcm_MCC_3	0.030
lbp-2D_glcm_MaximumProbability_3	0.380
lbp-2D_glcm_SumAverage_3	0.342
lbp-2D_glcm_SumEntropy_3	0.232
lbp-2D_glcm_SumSquares_3	0.309

lbp-2D_gldm_DependenceEntropy_3	0.271
lbp-2D_gldm_DependenceNonUniformity_3	0.485
lbp-2D_gldm_DependenceNonUniformityNormalized_3	0.224
lbp-2D_gldm_DependenceVariance_3	0.545
lbp-2D_gldm_GrayLevelNonUniformity_3	0.473
lbp-2D_gldm_GrayLevelVariance_3	0.313
lbp-2D_gldm_HighGrayLevelEmphasis_3	0.368
lbp-2D_gldm_LargeDependenceEmphasis_3	0.318
lbp-2D_gldm_LargeDependenceHighGrayLevelEmphasis_3	0.282
lbp-2D_gldm_LargeDependenceLowGrayLevelEmphasis_3	0.411
lbp-2D_gldm_LowGrayLevelEmphasis_3	0.368
lbp-2D_gldm_SmallDependenceEmphasis_3	0.416
lbp-2D_gldm_SmallDependenceHighGrayLevelEmphasis_3	0.441
lbp-2D_gldm_SmallDependenceLowGrayLevelEmphasis_3	0.377
lbp-2D_glrlm_GrayLevelNonUniformity_3	0.493
lbp-2D_glrlm_GrayLevelNonUniformityNormalized_3	0.240
lbp-2D_glrlm_GrayLevelVariance_3	0.240
lbp-2D_glrlm_HighGrayLevelRunEmphasis_3	0.374
lbp-2D_glrlm_LongRunEmphasis_3	0.504
lbp-2D_glrlm_LongRunHighGrayLevelEmphasis_3	0.351
lbp-2D_glrlm_LongRunLowGrayLevelEmphasis_3	0.573
lbp-2D_glrlm_LowGrayLevelRunEmphasis_3	0.374
lbp-2D_glrlm_RunEntropy_3	0.193
lbp-2D_glrlm_RunLengthNonUniformity_3	0.530
lbp-2D_glrlm_RunLengthNonUniformityNormalized_3	0.281
lbp-2D_glrlm_RunPercentage_3	0.327
lbp-2D_glrlm_RunVariance_3	0.321
lbp-2D_glrlm_ShortRunEmphasis_3	0.352
lbp-2D_glrlm_ShortRunHighGrayLevelEmphasis_3	0.372
lbp-2D_glrlm_ShortRunLowGrayLevelEmphasis_3	0.341
lbp-2D_glszm_GrayLevelNonUniformity_3	0.757
lbp-2D_glszm_GrayLevelNonUniformityNormalized_3	0.055
lbp-2D_glszm_GrayLevelVariance_3	0.055
lbp-2D_glszm_HighGrayLevelZoneEmphasis_3	0.081
lbp-2D_glszm_LargeAreaEmphasis_3	0.198
lbp-2D_glszm_LargeAreaHighGrayLevelEmphasis_3	0.297
lbp-2D_glszm_LargeAreaLowGrayLevelEmphasis_3	0.105
lbp-2D_glszm_LowGrayLevelZoneEmphasis_3	0.081
lbp-2D_glszm_SizeZoneNonUniformity_3	0.757
lbp-2D_glszm_SizeZoneNonUniformityNormalized_3	0.342
lbp-2D_glszm_SmallAreaEmphasis_3	0.412
lbp-2D_glszm_SmallAreaHighGrayLevelEmphasis_3	0.099
lbp-2D_glszm_SmallAreaLowGrayLevelEmphasis_3	0.351
lbp-2D_glszm_ZoneEntropy_3	0.099
lbp-2D_glszm_ZonePercentage_3	0.301
lbp-2D_glszm_ZoneVariance_3	0.200
lbp-2D_ngtdm_Busyness_3	0.150
lbp-2D_ngtdm_Coarseness_3	0.000
lbp-2D_ngtdm_Complexity_3	0.309
lbp-2D_ngtdm_Contrast_3	0.387

lbp-2D_ngtdm_Strength_3	0.009
lbp-3D-m1_firstorder_10Percentile_3	0.210
lbp-3D-m1_firstorder_90Percentile_3	0.478
lbp-3D-m1_firstorder_Energy_3	0.461
lbp-3D-m1_firstorder_Entropy_3	0.550
lbp-3D-m1_firstorder_InterquartileRange_3	0.332
lbp-3D-m1_firstorder_Kurtosis_3	0.446
lbp-3D-m1_firstorder_Maximum_3	0.334
lbp-3D-m1_firstorder_MeanAbsoluteDeviation_3	0.383
lbp-3D-m1_firstorder_Mean_3	0.322
lbp-3D-m1_firstorder_Median_3	0.301
lbp-3D-m1_firstorder_Minimum_3	0.596
lbp-3D-m1_firstorder_Range_3	0.452
lbp-3D-m1_firstorder_RobustMeanAbsoluteDeviation_3	0.331
lbp-3D-m1_firstorder_RootMeanSquared_3	0.392
lbp-3D-m1_firstorder_Skewness_3	0.671
lbp-3D-m1_firstorder_TotalEnergy_3	0.461
lbp-3D-m1_firstorder_Uniformity_3	0.603
lbp-3D-m1_firstorder_Variance_3	0.360
lbp-3D-m1_glcm_Autocorrelation_3	0.352
lbp-3D-m1_glcm_ClusterProminence_3	0.404
lbp-3D-m1_glcm_ClusterShade_3	0.341
lbp-3D-m1_glcm_ClusterTendency_3	0.429
lbp-3D-m1_glcm_Contrast_3	0.299
lbp-3D-m1_glcm_Correlation_3	0.438
lbp-3D-m1_glcm_DifferenceAverage_3	0.400
lbp-3D-m1_glcm_DifferenceEntropy_3	0.474
lbp-3D-m1_glcm_DifferenceVariance_3	0.387
lbp-3D-m1_glcm_Id_3	0.503
lbp-3D-m1_glcm_Idm_3	0.466
lbp-3D-m1_glcm_Idmn_3	0.303
lbp-3D-m1_glcm_Idn_3	0.453
lbp-3D-m1_glcm_Imc1_3	0.035
lbp-3D-m1_glcm_Imc2_3	0.253
lbp-3D-m1_glcm_InverseVariance_3	0.804
lbp-3D-m1_glcm_JointAverage_3	0.343
lbp-3D-m1_glcm_JointEnergy_3	0.676
lbp-3D-m1_glcm_JointEntropy_3	0.507
lbp-3D-m1_glcm_MCC_3	0.182
lbp-3D-m1_glcm_MaximumProbability_3	0.581
lbp-3D-m1_glcm_SumAverage_3	0.343
lbp-3D-m1_glcm_SumEntropy_3	0.547
lbp-3D-m1_glcm_SumSquares_3	0.364
lbp-3D-m1_gldm_DependenceEntropy_3	0.367
lbp-3D-m1_gldm_DependenceNonUniformity_3	0.467
lbp-3D-m1_gldm_DependenceNonUniformityNormalized_3	0.320
lbp-3D-m1_gldm_DependenceVariance_3	0.630
lbp-3D-m1_gldm_GrayLevelNonUniformity_3	0.507
lbp-3D-m1_gldm_GrayLevelVariance_3	0.429
lbp-3D-m1_gldm_HighGrayLevelEmphasis_3	0.415

lbp-3D-m1_gldm_LargeDependenceEmphasis_3	0.803
lbp-3D-m1_gldm_LargeDependenceHighGrayLevelEmphasis_3	0.451
lbp-3D-m1_gldm_LargeDependenceLowGrayLevelEmphasis_3	0.577
lbp-3D-m1_gldm_LowGrayLevelEmphasis_3	0.138
lbp-3D-m1_gldm_SmallDependenceEmphasis_3	0.444
lbp-3D-m1_gldm_SmallDependenceHighGrayLevelEmphasis_3	0.622
lbp-3D-m1_gldm_SmallDependenceLowGrayLevelEmphasis_3	0.215
lbp-3D-m1_glrlm_GrayLevelNonUniformity_3	0.502
lbp-3D-m1_glrlm_GrayLevelNonUniformityNormalized_3	0.603
lbp-3D-m1_glrlm_GrayLevelVariance_3	0.440
lbp-3D-m1_glrlm_HighGrayLevelRunEmphasis_3	0.427
lbp-3D-m1_glrlm_LongRunEmphasis_3	0.941
lbp-3D-m1_glrlm_LongRunHighGrayLevelEmphasis_3	0.536
lbp-3D-m1_glrlm_LongRunLowGrayLevelEmphasis_3	0.369
lbp-3D-m1_glrlm_LowGrayLevelRunEmphasis_3	0.156
lbp-3D-m1_glrlm_RunEntropy_3	0.448
lbp-3D-m1_glrlm_RunLengthNonUniformity_3	0.478
lbp-3D-m1_glrlm_RunLengthNonUniformityNormalized_3	0.611
lbp-3D-m1_glrlm_RunPercentage_3	0.748
lbp-3D-m1_glrlm_RunVariance_3	0.857
lbp-3D-m1_glrlm_ShortRunEmphasis_3	0.772
lbp-3D-m1_glrlm_ShortRunHighGrayLevelEmphasis_3	0.453
lbp-3D-m1_glrlm_ShortRunLowGrayLevelEmphasis_3	0.144
lbp-3D-m1_glszm_GrayLevelNonUniformity_3	0.584
lbp-3D-m1_glszm_GrayLevelNonUniformityNormalized_3	0.476
lbp-3D-m1_glszm_GrayLevelVariance_3	0.358
lbp-3D-m1_glszm_HighGrayLevelZoneEmphasis_3	0.508
lbp-3D-m1_glszm_LargeAreaEmphasis_3	0.244
lbp-3D-m1_glszm_LargeAreaHighGrayLevelEmphasis_3	0.381
lbp-3D-m1_glszm_LargeAreaLowGrayLevelEmphasis_3	0.080
lbp-3D-m1_glszm_LowGrayLevelZoneEmphasis_3	0.543
lbp-3D-m1_glszm_SizeZoneNonUniformity_3	0.683
lbp-3D-m1_glszm_SizeZoneNonUniformityNormalized_3	0.786
lbp-3D-m1_glszm_SmallAreaEmphasis_3	0.791
lbp-3D-m1_glszm_SmallAreaHighGrayLevelEmphasis_3	0.614
lbp-3D-m1_glszm_SmallAreaLowGrayLevelEmphasis_3	0.409
lbp-3D-m1_glszm_ZoneEntropy_3	0.628
lbp-3D-m1_glszm_ZonePercentage_3	0.262
lbp-3D-m1_glszm_ZoneVariance_3	0.245
lbp-3D-m1_ngtdm_Busyness_3	0.348
lbp-3D-m1_ngtdm_Coarseness_3	0.116
lbp-3D-m1_ngtdm_Complexity_3	0.382
lbp-3D-m1_ngtdm_Contrast_3	0.283
lbp-3D-m1_ngtdm_Strength_3	0.448
lbp-3D-m2_firstorder_10Percentile_3	0.183
lbp-3D-m2_firstorder_90Percentile_3	0.540
lbp-3D-m2_firstorder_Energy_3	0.473
lbp-3D-m2_firstorder_Entropy_3	0.409
lbp-3D-m2_firstorder_InterquartileRange_3	0.391
lbp-3D-m2_firstorder_Kurtosis_3	0.689

lbp-3D-m2_firstorder_Maximum_3	0.224
lbp-3D-m2_firstorder_MeanAbsoluteDeviation_3	0.404
lbp-3D-m2_firstorder_Mean_3	0.291
lbp-3D-m2_firstorder_Median_3	0.414
lbp-3D-m2_firstorder_Minimum_3	0.567
lbp-3D-m2_firstorder_Range_3	0.511
lbp-3D-m2_firstorder_RobustMeanAbsoluteDeviation_3	0.410
lbp-3D-m2_firstorder_RootMeanSquared_3	0.363
lbp-3D-m2_firstorder_Skewness_3	0.734
lbp-3D-m2_firstorder_TotalEnergy_3	0.473
lbp-3D-m2_firstorder_Uniformity_3	0.450
lbp-3D-m2_firstorder_Variance_3	0.325
lbp-3D-m2_glcm_Autocorrelation_3	0.396
lbp-3D-m2_glcm_ClusterProminence_3	0.339
lbp-3D-m2_glcm_ClusterShade_3	0.490
lbp-3D-m2_glcm_ClusterTendency_3	0.353
lbp-3D-m2_glcm_Contrast_3	0.282
lbp-3D-m2_glcm_Correlation_3	0.096
lbp-3D-m2_glcm_DifferenceAverage_3	0.284
lbp-3D-m2_glcm_DifferenceEntropy_3	0.268
lbp-3D-m2_glcm_DifferenceVariance_3	0.293
lbp-3D-m2_glcm_Id_3	0.295
lbp-3D-m2_glcm_Idm_3	0.289
lbp-3D-m2_glcm_Idmn_3	0.210
lbp-3D-m2_glcm_Idn_3	0.201
lbp-3D-m2_glcm_Imc1_3	0.059
lbp-3D-m2_glcm_Imc2_3	0.237
lbp-3D-m2_glcm_InverseVariance_3	0.386
lbp-3D-m2_glcm_JointAverage_3	0.457
lbp-3D-m2_glcm_JointEnergy_3	0.357
lbp-3D-m2_glcm_JointEntropy_3	0.313
lbp-3D-m2_glcm_MCC_3	0.175
lbp-3D-m2_glcm_MaximumProbability_3	0.376
lbp-3D-m2_glcm_SumAverage_3	0.457
lbp-3D-m2_glcm_SumEntropy_3	0.311
lbp-3D-m2_glcm_SumSquares_3	0.318
lbp-3D-m2_gldm_DependenceEntropy_3	0.399
lbp-3D-m2_gldm_DependenceNonUniformity_3	0.472
lbp-3D-m2_gldm_DependenceNonUniformityNormalized_3	0.205
lbp-3D-m2_gldm_DependenceVariance_3	0.299
lbp-3D-m2_gldm_GrayLevelNonUniformity_3	0.501
lbp-3D-m2_gldm_GrayLevelVariance_3	0.343
lbp-3D-m2_gldm_HighGrayLevelEmphasis_3	0.487
lbp-3D-m2_gldm_LargeDependenceEmphasis_3	0.561
lbp-3D-m2_gldm_LargeDependenceHighGrayLevelEmphasis_3	0.348
lbp-3D-m2_gldm_LargeDependenceLowGrayLevelEmphasis_3	0.833
lbp-3D-m2_gldm_LowGrayLevelEmphasis_3	0.494
lbp-3D-m2_gldm_SmallDependenceEmphasis_3	0.392
lbp-3D-m2_gldm_SmallDependenceHighGrayLevelEmphasis_3	0.496
lbp-3D-m2_gldm_SmallDependenceLowGrayLevelEmphasis_3	0.192

lbp-3D-m2_glrlm_GrayLevelNonUniformity_3	0.502
lbp-3D-m2_glrlm_GrayLevelNonUniformityNormalized_3	0.469
lbp-3D-m2_glrlm_GrayLevelVariance_3	0.340
lbp-3D-m2_glrlm_HighGrayLevelRunEmphasis_3	0.488
lbp-3D-m2_glrlm_LongRunEmphasis_3	0.826
lbp-3D-m2_glrlm_LongRunHighGrayLevelEmphasis_3	0.435
lbp-3D-m2_glrlm_LongRunLowGrayLevelEmphasis_3	0.738
lbp-3D-m2_glrlm_LowGrayLevelRunEmphasis_3	0.471
lbp-3D-m2_glrlm_RunEntropy_3	0.395
lbp-3D-m2_glrlm_RunLengthNonUniformity_3	0.481
lbp-3D-m2_glrlm_RunLengthNonUniformityNormalized_3	0.497
lbp-3D-m2_glrlm_RunPercentage_3	0.569
lbp-3D-m2_glrlm_RunVariance_3	0.646
lbp-3D-m2_glrlm_ShortRunEmphasis_3	0.609
lbp-3D-m2_glrlm_ShortRunHighGrayLevelEmphasis_3	0.509
lbp-3D-m2_glrlm_ShortRunLowGrayLevelEmphasis_3	0.406
lbp-3D-m2_glszm_GrayLevelNonUniformity_3	0.660
lbp-3D-m2_glszm_GrayLevelNonUniformityNormalized_3	0.445
lbp-3D-m2_glszm_GrayLevelVariance_3	0.302
lbp-3D-m2_glszm_HighGrayLevelZoneEmphasis_3	0.498
lbp-3D-m2_glszm_LargeAreaEmphasis_3	0.344
lbp-3D-m2_glszm_LargeAreaHighGrayLevelEmphasis_3	0.411
lbp-3D-m2_glszm_LargeAreaLowGrayLevelEmphasis_3	0.144
lbp-3D-m2_glszm_LowGrayLevelZoneEmphasis_3	0.339
lbp-3D-m2_glszm_SizeZoneNonUniformity_3	0.616
lbp-3D-m2_glszm_SizeZoneNonUniformityNormalized_3	0.755
lbp-3D-m2_glszm_SmallAreaEmphasis_3	0.688
lbp-3D-m2_glszm_SmallAreaHighGrayLevelEmphasis_3	0.577
lbp-3D-m2_glszm_SmallAreaLowGrayLevelEmphasis_3	0.351
lbp-3D-m2_glszm_ZoneEntropy_3	0.681
lbp-3D-m2_glszm_ZonePercentage_3	0.238
lbp-3D-m2_glszm_ZoneVariance_3	0.344
lbp-3D-m2_ngtdm_Busyness_3	0.184
lbp-3D-m2_ngtdm_Coarseness_3	0.100
lbp-3D-m2_ngtdm_Complexity_3	0.462
lbp-3D-m2_ngtdm_Contrast_3	0.245
lbp-3D-m2_ngtdm_Strength_3	0.191
lbp-3D-k_firstorder_10Percentile_3	0.469
lbp-3D-k_firstorder_90Percentile_3	0.625
lbp-3D-k_firstorder_Energy_3	0.515
lbp-3D-k_firstorder_Entropy_3	0.637
lbp-3D-k_firstorder_InterquartileRange_3	0.567
lbp-3D-k_firstorder_Kurtosis_3	0.547
lbp-3D-k_firstorder_Maximum_3	0.573
lbp-3D-k_firstorder_MeanAbsoluteDeviation_3	0.664
lbp-3D-k_firstorder_Mean_3	0.536
lbp-3D-k_firstorder_Median_3	0.452
lbp-3D-k_firstorder_Minimum_3	0.469
lbp-3D-k_firstorder_Range_3	0.567
lbp-3D-k_firstorder_RobustMeanAbsoluteDeviation_3	0.601

lbp-3D-k_firstorder_RootMeanSquared_3	0.432
lbp-3D-k_firstorder_Skewness_3	0.564
lbp-3D-k_firstorder_TotalEnergy_3	0.515
lbp-3D-k_firstorder_Uniformity_3	0.683
lbp-3D-k_firstorder_Variance_3	0.739
lbp-3D-k_glcm_Autocorrelation_3	0.623
lbp-3D-k_glcm_ClusterProminence_3	0.654
lbp-3D-k_glcm_ClusterShade_3	0.594
lbp-3D-k_glcm_ClusterTendency_3	0.613
lbp-3D-k_glcm_Contrast_3	0.513
lbp-3D-k_glcm_Correlation_3	0.264
lbp-3D-k_glcm_DifferenceAverage_3	0.514
lbp-3D-k_glcm_DifferenceEntropy_3	0.444
lbp-3D-k_glcm_DifferenceVariance_3	0.470
lbp-3D-k_glcm_Id_3	0.514
lbp-3D-k_glcm_Idm_3	0.514
lbp-3D-k_glcm_Idmn_3	0.523
lbp-3D-k_glcm_Idn_3	0.519
lbp-3D-k_glcm_Imc1_3	0.500
lbp-3D-k_glcm_Imc2_3	0.557
lbp-3D-k_glcm_InverseVariance_3	0.514
lbp-3D-k_glcm_JointAverage_3	0.600
lbp-3D-k_glcm_JointEnergy_3	0.534
lbp-3D-k_glcm_JointEntropy_3	0.519
lbp-3D-k_glcm_MCC_3	0.077
lbp-3D-k_glcm_MaximumProbability_3	0.566
lbp-3D-k_glcm_SumAverage_3	0.600
lbp-3D-k_glcm_SumEntropy_3	0.519
lbp-3D-k_glcm_SumSquares_3	0.573
lbp-3D-k_gldm_DependenceEntropy_3	0.322
lbp-3D-k_gldm_DependenceNonUniformity_3	0.524
lbp-3D-k_gldm_DependenceNonUniformityNormalized_3	0.153
lbp-3D-k_gldm_DependenceVariance_3	0.364
lbp-3D-k_gldm_GrayLevelNonUniformity_3	0.487
lbp-3D-k_gldm_GrayLevelVariance_3	0.683
lbp-3D-k_gldm_HighGrayLevelEmphasis_3	0.708
lbp-3D-k_gldm_LargeDependenceEmphasis_3	0.258
lbp-3D-k_gldm_LargeDependenceHighGrayLevelEmphasis_3	0.244
lbp-3D-k_gldm_LargeDependenceLowGrayLevelEmphasis_3	0.263
lbp-3D-k_gldm_LowGrayLevelEmphasis_3	0.708
lbp-3D-k_gldm_SmallDependenceEmphasis_3	0.402
lbp-3D-k_gldm_SmallDependenceHighGrayLevelEmphasis_3	0.596
lbp-3D-k_gldm_SmallDependenceLowGrayLevelEmphasis_3	0.309
lbp-3D-k_glrlm_GrayLevelNonUniformity_3	0.538
lbp-3D-k_glrlm_GrayLevelNonUniformityNormalized_3	0.472
lbp-3D-k_glrlm_GrayLevelVariance_3	0.472
lbp-3D-k_glrlm_HighGrayLevelRunEmphasis_3	0.477
lbp-3D-k_glrlm_LongRunEmphasis_3	0.217
lbp-3D-k_glrlm_LongRunHighGrayLevelEmphasis_3	0.190
lbp-3D-k_glrlm_LongRunLowGrayLevelEmphasis_3	0.226

lbp-3D-k_glrlm_LowGrayLevelRunEmphasis_3	0.477
lbp-3D-k_glrlm_RunEntropy_3	0.233
lbp-3D-k_glrlm_RunLengthNonUniformity_3	0.546
lbp-3D-k_glrlm_RunLengthNonUniformityNormalized_3	0.296
lbp-3D-k_glrlm_RunPercentage_3	0.297
lbp-3D-k_glrlm_RunVariance_3	0.139
lbp-3D-k_glrlm_ShortRunEmphasis_3	0.406
lbp-3D-k_glrlm_ShortRunHighGrayLevelEmphasis_3	0.720
lbp-3D-k_glrlm_ShortRunLowGrayLevelEmphasis_3	0.261
lbp-3D-k_glszm_GrayLevelNonUniformity_3	0.523
lbp-3D-k_glszm_GrayLevelNonUniformityNormalized_3	0.159
lbp-3D-k_glszm_GrayLevelVariance_3	0.160
lbp-3D-k_glszm_HighGrayLevelZoneEmphasis_3	0.211
lbp-3D-k_glszm_LargeAreaEmphasis_3	0.396
lbp-3D-k_glszm_LargeAreaHighGrayLevelEmphasis_3	0.396
lbp-3D-k_glszm_LargeAreaLowGrayLevelEmphasis_3	0.396
lbp-3D-k_glszm_LowGrayLevelZoneEmphasis_3	0.211
lbp-3D-k_glszm_SizeZoneNonUniformity_3	0.617
lbp-3D-k_glszm_SizeZoneNonUniformityNormalized_3	0.373
lbp-3D-k_glszm_SmallAreaEmphasis_3	0.517
lbp-3D-k_glszm_SmallAreaHighGrayLevelEmphasis_3	0.267
lbp-3D-k_glszm_SmallAreaLowGrayLevelEmphasis_3	0.702
lbp-3D-k_glszm_ZoneEntropy_3	0.369
lbp-3D-k_glszm_ZonePercentage_3	0.230
lbp-3D-k_glszm_ZoneVariance_3	0.396
lbp-3D-k_ngtdm_Busyness_3	0.392
lbp-3D-k_ngtdm_Coarseness_3	0.000
lbp-3D-k_ngtdm_Complexity_3	0.486
lbp-3D-k_ngtdm_Contrast_3	0.733
lbp-3D-k_ngtdm_Strength_3	0.845
logarithm_firstorder_10Percentile_3	0.867
logarithm_firstorder_90Percentile_3	0.980
logarithm_firstorder_Energy_3	0.452
logarithm_firstorder_Entropy_3	0.938
logarithm_firstorder_InterquartileRange_3	0.765
logarithm_firstorder_Kurtosis_3	0.753
logarithm_firstorder_Maximum_3	0.981
logarithm_firstorder_MeanAbsoluteDeviation_3	0.798
logarithm_firstorder_Mean_3	0.925
logarithm_firstorder_Median_3	0.930
logarithm_firstorder_Minimum_3	0.681
logarithm_firstorder_Range_3	0.823
logarithm_firstorder_RobustMeanAbsoluteDeviation_3	0.791
logarithm_firstorder_RootMeanSquared_3	0.933
logarithm_firstorder_Skewness_3	0.765
logarithm_firstorder_TotalEnergy_3	0.452
logarithm_firstorder_Uniformity_3	0.905
logarithm_firstorder_Variance_3	0.884
logarithm_glcm_Autocorrelation_3	0.481
logarithm_glcm_ClusterProminence_3	0.889

logarithm_glcm_ClusterShade_3	0.929
logarithm_glcm_ClusterTendency_3	0.916
logarithm_glcm_Contrast_3	0.942
logarithm_glcm_Correlation_3	0.855
logarithm_glcm_DifferenceAverage_3	0.942
logarithm_glcm_DifferenceEntropy_3	0.984
logarithm_glcm_DifferenceVariance_3	0.973
logarithm_glcm_Id_3	0.942
logarithm_glcm_Idm_3	0.942
logarithm_glcm_Idmn_3	0.942
logarithm_glcm_Idn_3	0.942
logarithm_glcm_Imc1_3	0.979
logarithm_glcm_Imc2_3	0.958
logarithm_glcm_InverseVariance_3	0.942
logarithm_glcm_JointAverage_3	0.504
logarithm_glcm_JointEnergy_3	0.950
logarithm_glcm_JointEntropy_3	0.960
logarithm_glcm_MCC_3	0.442
logarithm_glcm_MaximumProbability_3	0.924
logarithm_glcm_SumAverage_3	0.504
logarithm_glcm_SumEntropy_3	0.962
logarithm_glcm_SumSquares_3	0.931
logarithm_gldm_DependenceEntropy_3	0.258
logarithm_gldm_DependenceNonUniformity_3	0.427
logarithm_gldm_DependenceNonUniformityNormalized_3	0.153
logarithm_gldm_DependenceVariance_3	0.379
logarithm_gldm_GrayLevelNonUniformity_3	0.473
logarithm_gldm_GrayLevelVariance_3	0.905
logarithm_gldm_HighGrayLevelEmphasis_3	0.528
logarithm_gldm_LargeDependenceEmphasis_3	0.190
logarithm_gldm_LargeDependenceHighGrayLevelEmphasis_3	0.292
logarithm_gldm_LargeDependenceLowGrayLevelEmphasis_3	0.189
logarithm_gldm_LowGrayLevelEmphasis_3	0.528
logarithm_gldm_SmallDependenceEmphasis_3	0.279
logarithm_gldm_SmallDependenceHighGrayLevelEmphasis_3	0.226
logarithm_gldm_SmallDependenceLowGrayLevelEmphasis_3	0.317
logarithm_glrlm_GrayLevelNonUniformity_3	0.528
logarithm_glrlm_GrayLevelNonUniformityNormalized_3	0.923
logarithm_glrlm_GrayLevelVariance_3	0.923
logarithm_glrlm_HighGrayLevelRunEmphasis_3	0.514
logarithm_glrlm_LongRunEmphasis_3	0.095
logarithm_glrlm_LongRunHighGrayLevelEmphasis_3	0.160
logarithm_glrlm_LongRunLowGrayLevelEmphasis_3	0.086
logarithm_glrlm_LowGrayLevelRunEmphasis_3	0.514
logarithm_glrlm_RunEntropy_3	0.155
logarithm_glrlm_RunLengthNonUniformity_3	0.572
logarithm_glrlm_RunLengthNonUniformityNormalized_3	0.240
logarithm_glrlm_RunPercentage_3	0.230
logarithm_glrlm_RunVariance_3	0.068
logarithm_glrlm_ShortRunEmphasis_3	0.263

logarithm_glrlm_ShortRunHighGrayLevelEmphasis_3	0.459
logarithm_glrlm_ShortRunLowGrayLevelEmphasis_3	0.425
logarithm_glszm_GrayLevelNonUniformity_3	0.559
logarithm_glszm_GrayLevelNonUniformityNormalized_3	0.960
logarithm_glszm_GrayLevelVariance_3	0.960
logarithm_glszm_HighGrayLevelZoneEmphasis_3	0.331
logarithm_glszm_LargeAreaEmphasis_3	0.311
logarithm_glszm_LargeAreaHighGrayLevelEmphasis_3	0.311
logarithm_glszm_LargeAreaLowGrayLevelEmphasis_3	0.311
logarithm_glszm_LowGrayLevelZoneEmphasis_3	0.331
logarithm_glszm_SizeZoneNonUniformity_3	0.511
logarithm_glszm_SizeZoneNonUniformityNormalized_3	0.183
logarithm_glszm_SmallAreaEmphasis_3	0.645
logarithm_glszm_SmallAreaHighGrayLevelEmphasis_3	0.632
logarithm_glszm_SmallAreaLowGrayLevelEmphasis_3	0.648
logarithm_glszm_ZoneEntropy_3	0.345
logarithm_glszm_ZonePercentage_3	0.156
logarithm_glszm_ZoneVariance_3	0.327
logarithm_ngtdm_Busyness_3	0.758
logarithm_ngtdm_Coarseness_3	0.586
logarithm_ngtdm_Complexity_3	0.904
logarithm_ngtdm_Contrast_3	0.729
logarithm_ngtdm_Strength_3	0.019
square_firstorder_10Percentile_3	0.674
square_firstorder_90Percentile_3	0.880
square_firstorder_Energy_3	0.395
square_firstorder_Entropy_3	0.000
square_firstorder_InterquartileRange_3	0.468
square_firstorder_Kurtosis_3	0.936
square_firstorder_Maximum_3	0.854
square_firstorder_MeanAbsoluteDeviation_3	0.427
square_firstorder_Mean_3	0.800
square_firstorder_Median_3	0.806
square_firstorder_Minimum_3	0.708
square_firstorder_Range_3	0.909
square_firstorder_RobustMeanAbsoluteDeviation_3	0.447
square_firstorder_RootMeanSquared_3	0.816
square_firstorder_Skewness_3	0.849
square_firstorder_TotalEnergy_3	0.395
square_firstorder_Variance_3	0.247
square_glcm_DifferenceEntropy_3	0.000
square_glcm_JointEntropy_3	0.000
square_glcm_SumEntropy_3	0.000
square_gldm_DependenceEntropy_3	0.298
square_gldm_DependenceNonUniformity_3	0.427
square_gldm_DependenceNonUniformityNormalized_3	0.167
square_gldm_DependenceVariance_3	0.350
square_gldm_GrayLevelNonUniformity_3	0.473
square_gldm_LargeDependenceEmphasis_3	0.171
square_gldm_LargeDependenceHighGrayLevelEmphasis_3	0.171

square_gldm_LargeDependenceLowGrayLevelEmphasis_3	0.171
square_gldm_SmallDependenceEmphasis_3	0.279
square_gldm_SmallDependenceHighGrayLevelEmphasis_3	0.279
square_gldm_SmallDependenceLowGrayLevelEmphasis_3	0.279
square_glrlm_GrayLevelNonUniformity_3	0.528
square_glrlm_LongRunEmphasis_3	0.092
square_glrlm_LongRunHighGrayLevelEmphasis_3	0.092
square_glrlm_LongRunLowGrayLevelEmphasis_3	0.092
square_glrlm_RunEntropy_3	0.179
square_glrlm_RunLengthNonUniformity_3	0.573
square_glrlm_RunLengthNonUniformityNormalized_3	0.213
square_glrlm_RunPercentage_3	0.207
square_glrlm_RunVariance_3	0.067
square_glrlm_ShortRunEmphasis_3	0.236
square_glrlm_ShortRunHighGrayLevelEmphasis_3	0.236
square_glrlm_ShortRunLowGrayLevelEmphasis_3	0.236
square_glszm_GrayLevelNonUniformity_3	0.557
square_glszm_LargeAreaEmphasis_3	0.311
square_glszm_LargeAreaHighGrayLevelEmphasis_3	0.311
square_glszm_LargeAreaLowGrayLevelEmphasis_3	0.311
square_glszm_SizeZoneNonUniformity_3	0.509
square_glszm_SizeZoneNonUniformityNormalized_3	0.169
square_glszm_SmallAreaEmphasis_3	0.663
square_glszm_SmallAreaHighGrayLevelEmphasis_3	0.663
square_glszm_SmallAreaLowGrayLevelEmphasis_3	0.663
square_glszm_ZoneEntropy_3	0.300
square_glszm_ZonePercentage_3	0.153
square_glszm_ZoneVariance_3	0.327
squareroot_firstorder_10Percentile_3	0.874
squareroot_firstorder_90Percentile_3	0.978
squareroot_firstorder_Energy_3	0.459
squareroot_firstorder_Entropy_3	0.938
squareroot_firstorder_InterquartileRange_3	0.783
squareroot_firstorder_Kurtosis_3	0.760
squareroot_firstorder_Maximum_3	0.980
squareroot_firstorder_MeanAbsoluteDeviation_3	0.866
squareroot_firstorder_Mean_3	0.939
squareroot_firstorder_Median_3	0.944
squareroot_firstorder_Minimum_3	0.706
squareroot_firstorder_Range_3	0.811
squareroot_firstorder_RobustMeanAbsoluteDeviation_3	0.813
squareroot_firstorder_RootMeanSquared_3	0.944
squareroot_firstorder_Skewness_3	0.770
squareroot_firstorder_TotalEnergy_3	0.459
squareroot_firstorder_Uniformity_3	0.905
squareroot_firstorder_Variance_3	0.927
squareroot_glcm_Autocorrelation_3	0.481
squareroot_glcm_ClusterProminence_3	0.889
squareroot_glcm_ClusterShade_3	0.929
squareroot_glcm_ClusterTendency_3	0.916

squareroot_glcm_Contrast_3	0.942
squareroot_glcm_Correlation_3	0.855
squareroot_glcm_DifferenceAverage_3	0.942
squareroot_glcm_DifferenceEntropy_3	0.984
squareroot_glcm_DifferenceVariance_3	0.973
squareroot_glcm_Id_3	0.942
squareroot_glcm_Idm_3	0.942
squareroot_glcm_Idmn_3	0.942
squareroot_glcm_Idn_3	0.942
squareroot_glcm_Imc1_3	0.979
squareroot_glcm_Imc2_3	0.958
squareroot_glcm_InverseVariance_3	0.942
squareroot_glcm_JointAverage_3	0.504
squareroot_glcm_JointEnergy_3	0.950
squareroot_glcm_JointEntropy_3	0.960
squareroot_glcm_MCC_3	0.442
squareroot_glcm_MaximumProbability_3	0.924
squareroot_glcm_SumAverage_3	0.504
squareroot_glcm_SumEntropy_3	0.962
squareroot_glcm_SumSquares_3	0.931
squareroot_gldm_DependenceEntropy_3	0.258
squareroot_gldm_DependenceNonUniformity_3	0.427
squareroot_gldm_DependenceNonUniformityNormalized_3	0.153
squareroot_gldm_DependenceVariance_3	0.379
squareroot_gldm_GrayLevelNonUniformity_3	0.473
squareroot_gldm_GrayLevelVariance_3	0.905
squareroot_gldm_HighGrayLevelEmphasis_3	0.528
squareroot_gldm_LargeDependenceEmphasis_3	0.190
squareroot_gldm_LargeDependenceHighGrayLevelEmphasis_3	0.292
squareroot_gldm_LargeDependenceLowGrayLevelEmphasis_3	0.189
squareroot_gldm_LowGrayLevelEmphasis_3	0.528
squareroot_gldm_SmallDependenceEmphasis_3	0.279
squareroot_gldm_SmallDependenceHighGrayLevelEmphasis_3	0.226
squareroot_gldm_SmallDependenceLowGrayLevelEmphasis_3	0.317
squareroot_glrlm_GrayLevelNonUniformity_3	0.528
squareroot_glrlm_GrayLevelNonUniformityNormalized_3	0.923
squareroot_glrlm_GrayLevelVariance_3	0.923
squareroot_glrlm_HighGrayLevelRunEmphasis_3	0.514
squareroot_glrlm_LongRunEmphasis_3	0.095
squareroot_glrlm_LongRunHighGrayLevelEmphasis_3	0.160
squareroot_glrlm_LongRunLowGrayLevelEmphasis_3	0.086
squareroot_glrlm_LowGrayLevelRunEmphasis_3	0.514
squareroot_glrlm_RunEntropy_3	0.155
squareroot_glrlm_RunLengthNonUniformity_3	0.572
squareroot_glrlm_RunLengthNonUniformityNormalized_3	0.240
squareroot_glrlm_RunPercentage_3	0.230
squareroot_glrlm_RunVariance_3	0.068
squareroot_glrlm_ShortRunEmphasis_3	0.263
squareroot_glrlm_ShortRunHighGrayLevelEmphasis_3	0.459
squareroot_glrlm_ShortRunLowGrayLevelEmphasis_3	0.425

squareroot_glszm_GrayLevelNonUniformity_3	0.559
squareroot_glszm_GrayLevelNonUniformityNormalized_3	0.960
squareroot_glszm_GrayLevelVariance_3	0.960
squareroot_glszm_HighGrayLevelZoneEmphasis_3	0.331
squareroot_glszm_LargeAreaEmphasis_3	0.311
squareroot_glszm_LargeAreaHighGrayLevelEmphasis_3	0.311
squareroot_glszm_LargeAreaLowGrayLevelEmphasis_3	0.311
squareroot_glszm_LowGrayLevelZoneEmphasis_3	0.331
squareroot_glszm_SizeZoneNonUniformity_3	0.511
squareroot_glszm_SizeZoneNonUniformityNormalized_3	0.183
squareroot_glszm_SmallAreaEmphasis_3	0.645
squareroot_glszm_SmallAreaHighGrayLevelEmphasis_3	0.632
squareroot_glszm_SmallAreaLowGrayLevelEmphasis_3	0.648
squareroot_glszm_ZoneEntropy_3	0.345
squareroot_glszm_ZonePercentage_3	0.156
squareroot_glszm_ZoneVariance_3	0.327
squareroot_ngtdm_Busyness_3	0.758
squareroot_ngtdm_Coarseness_3	0.586
squareroot_ngtdm_Complexity_3	0.904
squareroot_ngtdm_Contrast_3	0.729
squareroot_ngtdm_Strength_3	0.019
wavelet-LLH_firstorder_10Percentile_3	0.694
wavelet-LLH_firstorder_90Percentile_3	0.915
wavelet-LLH_firstorder_Energy_3	0.859
wavelet-LLH_firstorder_Entropy_3	0.257
wavelet-LLH_firstorder_InterquartileRange_3	0.770
wavelet-LLH_firstorder_Kurtosis_3	0.589
wavelet-LLH_firstorder_Maximum_3	0.916
wavelet-LLH_firstorder_MeanAbsoluteDeviation_3	0.795
wavelet-LLH_firstorder_Mean_3	0.657
wavelet-LLH_firstorder_Median_3	0.640
wavelet-LLH_firstorder_Minimum_3	0.917
wavelet-LLH_firstorder_Range_3	0.939
wavelet-LLH_firstorder_RobustMeanAbsoluteDeviation_3	0.774
wavelet-LLH_firstorder_RootMeanSquared_3	0.773
wavelet-LLH_firstorder_Skewness_3	0.613
wavelet-LLH_firstorder_TotalEnergy_3	0.859
wavelet-LLH_firstorder_Uniformity_3	0.317
wavelet-LLH_firstorder_Variance_3	0.867
wavelet-LLH_glcm_Autocorrelation_3	0.495
wavelet-LLH_glcm_ClusterProminence_3	0.311
wavelet-LLH_glcm_ClusterShade_3	0.423
wavelet-LLH_glcm_ClusterTendency_3	0.322
wavelet-LLH_glcm_Contrast_3	0.533
wavelet-LLH_glcm_Correlation_3	0.529
wavelet-LLH_glcm_DifferenceAverage_3	0.533
wavelet-LLH_glcm_DifferenceEntropy_3	0.240
wavelet-LLH_glcm_DifferenceVariance_3	0.313
wavelet-LLH_glcm_Id_3	0.533
wavelet-LLH_glcm_Idm_3	0.533

wavelet-LLH_glcm_Idmn_3	0.533
wavelet-LLH_glcm_Idn_3	0.533
wavelet-LLH_glcm_Imc1_3	0.199
wavelet-LLH_glcm_Imc2_3	0.439
wavelet-LLH_glcm_InverseVariance_3	0.533
wavelet-LLH_glcm_JointAverage_3	0.524
wavelet-LLH_glcm_JointEnergy_3	0.330
wavelet-LLH_glcm_JointEntropy_3	0.317
wavelet-LLH_glcm_MCC_3	0.395
wavelet-LLH_glcm_MaximumProbability_3	0.456
wavelet-LLH_glcm_SumAverage_3	0.524
wavelet-LLH_glcm_SumEntropy_3	0.280
wavelet-LLH_glcm_SumSquares_3	0.338
wavelet-LLH_gldm_DependenceEntropy_3	0.292
wavelet-LLH_gldm_DependenceNonUniformity_3	0.506
wavelet-LLH_gldm_DependenceNonUniformityNormalized_3	0.185
wavelet-LLH_gldm_DependenceVariance_3	0.454
wavelet-LLH_gldm_GrayLevelNonUniformity_3	0.464
wavelet-LLH_gldm_GrayLevelVariance_3	0.317
wavelet-LLH_gldm_HighGrayLevelEmphasis_3	0.537
wavelet-LLH_gldm_LargeDependenceEmphasis_3	0.284
wavelet-LLH_gldm_LargeDependenceHighGrayLevelEmphasis_3	0.336
wavelet-LLH_gldm_LargeDependenceLowGrayLevelEmphasis_3	0.309
wavelet-LLH_gldm_LowGrayLevelEmphasis_3	0.537
wavelet-LLH_gldm_SmallDependenceEmphasis_3	0.307
wavelet-LLH_gldm_SmallDependenceHighGrayLevelEmphasis_3	0.362
wavelet-LLH_gldm_SmallDependenceLowGrayLevelEmphasis_3	0.259
wavelet-LLH_glrlm_GrayLevelNonUniformity_3	0.507
wavelet-LLH_glrlm_GrayLevelNonUniformityNormalized_3	0.148
wavelet-LLH_glrlm_GrayLevelVariance_3	0.148
wavelet-LLH_glrlm_HighGrayLevelRunEmphasis_3	0.505
wavelet-LLH_glrlm_LongRunEmphasis_3	0.195
wavelet-LLH_glrlm_LongRunHighGrayLevelEmphasis_3	0.185
wavelet-LLH_glrlm_LongRunLowGrayLevelEmphasis_3	0.248
wavelet-LLH_glrlm_LowGrayLevelRunEmphasis_3	0.505
wavelet-LLH_glrlm_RunEntropy_3	0.201
wavelet-LLH_glrlm_RunLengthNonUniformity_3	0.547
wavelet-LLH_glrlm_RunLengthNonUniformityNormalized_3	0.348
wavelet-LLH_glrlm_RunPercentage_3	0.326
wavelet-LLH_glrlm_RunVariance_3	0.153
wavelet-LLH_glrlm_ShortRunEmphasis_3	0.347
wavelet-LLH_glrlm_ShortRunHighGrayLevelEmphasis_3	0.689
wavelet-LLH_glrlm_ShortRunLowGrayLevelEmphasis_3	0.006
wavelet-LLH_glszm_GrayLevelNonUniformity_3	0.819
wavelet-LLH_glszm_GrayLevelNonUniformityNormalized_3	0.082
wavelet-LLH_glszm_GrayLevelVariance_3	0.082
wavelet-LLH_glszm_HighGrayLevelZoneEmphasis_3	0.290
wavelet-LLH_glszm_LargeAreaEmphasis_3	0.279
wavelet-LLH_glszm_LargeAreaHighGrayLevelEmphasis_3	0.390
wavelet-LLH_glszm_LargeAreaLowGrayLevelEmphasis_3	0.202

wavelet-LLH_glszm_LowGrayLevelZoneEmphasis_3	0.290
wavelet-LLH_glszm_SizeZoneNonUniformity_3	0.842
wavelet-LLH_glszm_SizeZoneNonUniformityNormalized_3	0.579
wavelet-LLH_glszm_SmallAreaEmphasis_3	0.656
wavelet-LLH_glszm_SmallAreaHighGrayLevelEmphasis_3	0.461
wavelet-LLH_glszm_SmallAreaLowGrayLevelEmphasis_3	0.682
wavelet-LLH_glszm_ZoneEntropy_3	0.471
wavelet-LLH_glszm_ZonePercentage_3	0.238
wavelet-LLH_glszm_ZoneVariance_3	0.280
wavelet-LLH_ngtdm_Busyness_3	0.008
wavelet-LLH_ngtdm_Coarseness_3	0.009
wavelet-LLH_ngtdm_Complexity_3	0.558
wavelet-LLH_ngtdm_Contrast_3	0.585
wavelet-LLH_ngtdm_Strength_3	0.006
wavelet-LHL_firstorder_10Percentile_3	0.491
wavelet-LHL_firstorder_90Percentile_3	0.945
wavelet-LHL_firstorder_Energy_3	0.663
wavelet-LHL_firstorder_Entropy_3	0.084
wavelet-LHL_firstorder_InterquartileRange_3	0.868
wavelet-LHL_firstorder_Kurtosis_3	0.901
wavelet-LHL_firstorder_Maximum_3	0.827
wavelet-LHL_firstorder_MeanAbsoluteDeviation_3	0.823
wavelet-LHL_firstorder_Mean_3	0.715
wavelet-LHL_firstorder_Median_3	0.880
wavelet-LHL_firstorder_Minimum_3	0.908
wavelet-LHL_firstorder_Range_3	0.905
wavelet-LHL_firstorder_RobustMeanAbsoluteDeviation_3	0.843
wavelet-LHL_firstorder_RootMeanSquared_3	0.789
wavelet-LHL_firstorder_Skewness_3	0.566
wavelet-LHL_firstorder_TotalEnergy_3	0.663
wavelet-LHL_firstorder_Uniformity_3	0.116
wavelet-LHL_firstorder_Variance_3	0.858
wavelet-LHL_glcm_Autocorrelation_3	0.295
wavelet-LHL_glcm_ClusterProminence_3	0.347
wavelet-LHL_glcm_ClusterShade_3	0.517
wavelet-LHL_glcm_ClusterTendency_3	0.199
wavelet-LHL_glcm_Contrast_3	0.238
wavelet-LHL_glcm_Correlation_3	0.301
wavelet-LHL_glcm_DifferenceAverage_3	0.238
wavelet-LHL_glcm_DifferenceEntropy_3	0.119
wavelet-LHL_glcm_DifferenceVariance_3	0.166
wavelet-LHL_glcm_Id_3	0.238
wavelet-LHL_glcm_Idm_3	0.238
wavelet-LHL_glcm_Idmn_3	0.238
wavelet-LHL_glcm_Idn_3	0.238
wavelet-LHL_glcm_Imc1_3	0.695
wavelet-LHL_glcm_Imc2_3	0.643
wavelet-LHL_glcm_InverseVariance_3	0.238
wavelet-LHL_glcm_JointAverage_3	0.223
wavelet-LHL_glcm_JointEnergy_3	0.044

wavelet-LHL_glcm_JointEntropy_3	0.049
wavelet-LHL_glcm_MCC_3	0.676
wavelet-LHL_glcm_MaximumProbability_3	0.164
wavelet-LHL_glcm_SumAverage_3	0.223
wavelet-LHL_glcm_SumEntropy_3	0.046
wavelet-LHL_glcm_SumSquares_3	0.014
wavelet-LHL_gldm_DependenceEntropy_3	0.256
wavelet-LHL_gldm_DependenceNonUniformity_3	0.497
wavelet-LHL_gldm_DependenceNonUniformityNormalized_3	0.161
wavelet-LHL_gldm_DependenceVariance_3	0.428
wavelet-LHL_gldm_GrayLevelNonUniformity_3	0.476
wavelet-LHL_gldm_GrayLevelVariance_3	0.116
wavelet-LHL_gldm_HighGrayLevelEmphasis_3	0.525
wavelet-LHL_gldm_LargeDependenceEmphasis_3	0.258
wavelet-LHL_gldm_LargeDependenceHighGrayLevelEmphasis_3	0.243
wavelet-LHL_gldm_LargeDependenceLowGrayLevelEmphasis_3	0.294
wavelet-LHL_gldm_LowGrayLevelEmphasis_3	0.525
wavelet-LHL_gldm_SmallDependenceEmphasis_3	0.388
wavelet-LHL_gldm_SmallDependenceHighGrayLevelEmphasis_3	0.522
wavelet-LHL_gldm_SmallDependenceLowGrayLevelEmphasis_3	0.234
wavelet-LHL_glrlm_GrayLevelNonUniformity_3	0.495
wavelet-LHL_glrlm_GrayLevelNonUniformityNormalized_3	0.127
wavelet-LHL_glrlm_GrayLevelVariance_3	0.127
wavelet-LHL_glrlm_HighGrayLevelRunEmphasis_3	0.493
wavelet-LHL_glrlm_LongRunEmphasis_3	0.409
wavelet-LHL_glrlm_LongRunHighGrayLevelEmphasis_3	0.343
wavelet-LHL_glrlm_LongRunLowGrayLevelEmphasis_3	0.473
wavelet-LHL_glrlm_LowGrayLevelRunEmphasis_3	0.493
wavelet-LHL_glrlm_RunEntropy_3	0.245
wavelet-LHL_glrlm_RunLengthNonUniformity_3	0.521
wavelet-LHL_glrlm_RunLengthNonUniformityNormalized_3	0.271
wavelet-LHL_glrlm_RunPercentage_3	0.280
wavelet-LHL_glrlm_RunVariance_3	0.250
wavelet-LHL_glrlm_ShortRunEmphasis_3	0.326
wavelet-LHL_glrlm_ShortRunHighGrayLevelEmphasis_3	0.747
wavelet-LHL_glrlm_ShortRunLowGrayLevelEmphasis_3	0.189
wavelet-LHL_glszm_GrayLevelNonUniformity_3	0.747
wavelet-LHL_glszm_GrayLevelNonUniformityNormalized_3	0.022
wavelet-LHL_glszm_GrayLevelVariance_3	0.022
wavelet-LHL_glszm_HighGrayLevelZoneEmphasis_3	0.039
wavelet-LHL_glszm_LargeAreaEmphasis_3	0.228
wavelet-LHL_glszm_LargeAreaHighGrayLevelEmphasis_3	0.190
wavelet-LHL_glszm_LargeAreaLowGrayLevelEmphasis_3	0.264
wavelet-LHL_glszm_LowGrayLevelZoneEmphasis_3	0.039
wavelet-LHL_glszm_SizeZoneNonUniformity_3	0.822
wavelet-LHL_glszm_SizeZoneNonUniformityNormalized_3	0.621
wavelet-LHL_glszm_SmallAreaEmphasis_3	0.779
wavelet-LHL_glszm_SmallAreaHighGrayLevelEmphasis_3	0.627
wavelet-LHL_glszm_SmallAreaLowGrayLevelEmphasis_3	0.723
wavelet-LHL_glszm_ZoneEntropy_3	0.414

wavelet-LHL_glszm_ZonePercentage_3	0.285
wavelet-LHL_glszm_ZoneVariance_3	0.230
wavelet-LHL_ngtdm_Busyness_3	0.494
wavelet-LHL_ngtdm_Coarseness_3	0.003
wavelet-LHL_ngtdm_Complexity_3	0.291
wavelet-LHL_ngtdm_Contrast_3	0.311
wavelet-LHL_ngtdm_Strength_3	0.003
wavelet-LHH_firstorder_10Percentile_3	0.975
wavelet-LHH_firstorder_90Percentile_3	0.527
wavelet-LHH_firstorder_Energy_3	0.672
wavelet-LHH_firstorder_Entropy_3	0.232
wavelet-LHH_firstorder_InterquartileRange_3	0.893
wavelet-LHH_firstorder_Kurtosis_3	0.932
wavelet-LHH_firstorder_Maximum_3	0.906
wavelet-LHH_firstorder_MeanAbsoluteDeviation_3	0.862
wavelet-LHH_firstorder_Mean_3	0.537
wavelet-LHH_firstorder_Median_3	0.838
wavelet-LHH_firstorder_Minimum_3	0.819
wavelet-LHH_firstorder_Range_3	0.911
wavelet-LHH_firstorder_RobustMeanAbsoluteDeviation_3	0.879
wavelet-LHH_firstorder_RootMeanSquared_3	0.843
wavelet-LHH_firstorder_Skewness_3	0.755
wavelet-LHH_firstorder_TotalEnergy_3	0.672
wavelet-LHH_firstorder_Uniformity_3	0.238
wavelet-LHH_firstorder_Variance_3	0.909
wavelet-LHH_glcm_Autocorrelation_3	0.366
wavelet-LHH_glcm_ClusterProminence_3	0.750
wavelet-LHH_glcm_ClusterShade_3	0.455
wavelet-LHH_glcm_ClusterTendency_3	0.624
wavelet-LHH_glcm_Contrast_3	0.678
wavelet-LHH_glcm_Correlation_3	0.795
wavelet-LHH_glcm_DifferenceAverage_3	0.678
wavelet-LHH_glcm_DifferenceEntropy_3	0.199
wavelet-LHH_glcm_DifferenceVariance_3	0.260
wavelet-LHH_glcm_Id_3	0.678
wavelet-LHH_glcm_Idm_3	0.678
wavelet-LHH_glcm_Idmn_3	0.678
wavelet-LHH_glcm_Idn_3	0.678
wavelet-LHH_glcm_Imc1_3	0.379
wavelet-LHH_glcm_Imc2_3	0.635
wavelet-LHH_glcm_InverseVariance_3	0.678
wavelet-LHH_glcm_JointAverage_3	0.362
wavelet-LHH_glcm_JointEnergy_3	0.090
wavelet-LHH_glcm_JointEntropy_3	0.106
wavelet-LHH_glcm_MCC_3	0.622
wavelet-LHH_glcm_MaximumProbability_3	0.250
wavelet-LHH_glcm_SumAverage_3	0.362
wavelet-LHH_glcm_SumEntropy_3	0.121
wavelet-LHH_glcm_SumSquares_3	0.023
wavelet-LHH_gldm_DependenceEntropy_3	0.347

wavelet-LHH_gldm_DependenceNonUniformity_3	0.494
wavelet-LHH_gldm_DependenceNonUniformityNormalized_3	0.260
wavelet-LHH_gldm_DependenceVariance_3	0.654
wavelet-LHH_gldm_GrayLevelNonUniformity_3	0.473
wavelet-LHH_gldm_GrayLevelVariance_3	0.238
wavelet-LHH_gldm_HighGrayLevelEmphasis_3	0.416
wavelet-LHH_gldm_LargeDependenceEmphasis_3	0.255
wavelet-LHH_gldm_LargeDependenceHighGrayLevelEmphasis_3	0.249
wavelet-LHH_gldm_LargeDependenceLowGrayLevelEmphasis_3	0.271
wavelet-LHH_gldm_LowGrayLevelEmphasis_3	0.416
wavelet-LHH_gldm_SmallDependenceEmphasis_3	0.373
wavelet-LHH_gldm_SmallDependenceHighGrayLevelEmphasis_3	0.281
wavelet-LHH_gldm_SmallDependenceLowGrayLevelEmphasis_3	0.455
wavelet-LHH_glrlm_GrayLevelNonUniformity_3	0.495
wavelet-LHH_glrlm_GrayLevelNonUniformityNormalized_3	0.252
wavelet-LHH_glrlm_GrayLevelVariance_3	0.252
wavelet-LHH_glrlm_HighGrayLevelRunEmphasis_3	0.429
wavelet-LHH_glrlm_LongRunEmphasis_3	0.383
wavelet-LHH_glrlm_LongRunHighGrayLevelEmphasis_3	0.417
wavelet-LHH_glrlm_LongRunLowGrayLevelEmphasis_3	0.351
wavelet-LHH_glrlm_LowGrayLevelRunEmphasis_3	0.429
wavelet-LHH_glrlm_RunEntropy_3	0.261
wavelet-LHH_glrlm_RunLengthNonUniformity_3	0.525
wavelet-LHH_glrlm_RunLengthNonUniformityNormalized_3	0.261
wavelet-LHH_glrlm_RunPercentage_3	0.259
wavelet-LHH_glrlm_RunVariance_3	0.238
wavelet-LHH_glrlm_ShortRunEmphasis_3	0.274
wavelet-LHH_glrlm_ShortRunHighGrayLevelEmphasis_3	0.077
wavelet-LHH_glrlm_ShortRunLowGrayLevelEmphasis_3	0.555
wavelet-LHH_glszm_GrayLevelNonUniformity_3	0.770
wavelet-LHH_glszm_GrayLevelNonUniformityNormalized_3	0.018
wavelet-LHH_glszm_GrayLevelVariance_3	0.018
wavelet-LHH_glszm_HighGrayLevelZoneEmphasis_3	0.282
wavelet-LHH_glszm_LargeAreaEmphasis_3	0.283
wavelet-LHH_glszm_LargeAreaHighGrayLevelEmphasis_3	0.308
wavelet-LHH_glszm_LargeAreaLowGrayLevelEmphasis_3	0.260
wavelet-LHH_glszm_LowGrayLevelZoneEmphasis_3	0.282
wavelet-LHH_glszm_SizeZoneNonUniformity_3	0.853
wavelet-LHH_glszm_SizeZoneNonUniformityNormalized_3	0.552
wavelet-LHH_glszm_SmallAreaEmphasis_3	0.695
wavelet-LHH_glszm_SmallAreaHighGrayLevelEmphasis_3	0.611
wavelet-LHH_glszm_SmallAreaLowGrayLevelEmphasis_3	0.657
wavelet-LHH_glszm_ZoneEntropy_3	0.519
wavelet-LHH_glszm_ZonePercentage_3	0.295
wavelet-LHH_glszm_ZoneVariance_3	0.285
wavelet-LHH_ngtdm_Busyness_3	0.407
wavelet-LHH_ngtdm_Coarseness_3	0.019
wavelet-LHH_ngtdm_Complexity_3	0.609
wavelet-LHH_ngtdm_Contrast_3	0.573
wavelet-LHH_ngtdm_Strength_3	0.019

wavelet-HLL_firstorder_10Percentile_3	0.597
wavelet-HLL_firstorder_90Percentile_3	0.654
wavelet-HLL_firstorder_Energy_3	0.733
wavelet-HLL_firstorder_Entropy_3	0.494
wavelet-HLL_firstorder_InterquartileRange_3	0.517
wavelet-HLL_firstorder_Kurtosis_3	0.490
wavelet-HLL_firstorder_Maximum_3	0.687
wavelet-HLL_firstorder_MeanAbsoluteDeviation_3	0.588
wavelet-HLL_firstorder_Mean_3	0.619
wavelet-HLL_firstorder_Median_3	0.725
wavelet-HLL_firstorder_Minimum_3	0.808
wavelet-HLL_firstorder_Range_3	0.857
wavelet-HLL_firstorder_RobustMeanAbsoluteDeviation_3	0.527
wavelet-HLL_firstorder_RootMeanSquared_3	0.639
wavelet-HLL_firstorder_Skewness_3	0.616
wavelet-HLL_firstorder_TotalEnergy_3	0.733
wavelet-HLL_firstorder_Uniformity_3	0.491
wavelet-HLL_firstorder_Variance_3	0.514
wavelet-HLL_glcm_Autocorrelation_3	0.397
wavelet-HLL_glcm_ClusterProminence_3	0.224
wavelet-HLL_glcm_ClusterShade_3	0.402
wavelet-HLL_glcm_ClusterTendency_3	0.380
wavelet-HLL_glcm_Contrast_3	0.459
wavelet-HLL_glcm_Correlation_3	0.208
wavelet-HLL_glcm_DifferenceAverage_3	0.459
wavelet-HLL_glcm_DifferenceEntropy_3	0.373
wavelet-HLL_glcm_DifferenceVariance_3	0.429
wavelet-HLL_glcm_Id_3	0.459
wavelet-HLL_glcm_Idm_3	0.459
wavelet-HLL_glcm_Idmn_3	0.459
wavelet-HLL_glcm_Idn_3	0.459
wavelet-HLL_glcm_Imc1_3	0.680
wavelet-HLL_glcm_Imc2_3	0.231
wavelet-HLL_glcm_InverseVariance_3	0.459
wavelet-HLL_glcm_JointAverage_3	0.405
wavelet-HLL_glcm_JointEnergy_3	0.420
wavelet-HLL_glcm_JointEntropy_3	0.397
wavelet-HLL_glcm_MCC_3	0.302
wavelet-HLL_glcm_MaximumProbability_3	0.456
wavelet-HLL_glcm_SumAverage_3	0.405
wavelet-HLL_glcm_SumEntropy_3	0.372
wavelet-HLL_glcm_SumSquares_3	0.417
wavelet-HLL_gldm_DependenceEntropy_3	0.290
wavelet-HLL_gldm_DependenceNonUniformity_3	0.492
wavelet-HLL_gldm_DependenceNonUniformityNormalized_3	0.220
wavelet-HLL_gldm_DependenceVariance_3	0.628
wavelet-HLL_gldm_GrayLevelNonUniformity_3	0.473
wavelet-HLL_gldm_GrayLevelVariance_3	0.491
wavelet-HLL_gldm_HighGrayLevelEmphasis_3	0.456
wavelet-HLL_gldm_LargeDependenceEmphasis_3	0.335

wavelet-HLL_gldm_LargeDependenceHighGrayLevelEmphasis_3	0.234
wavelet-HLL_gldm_LargeDependenceLowGrayLevelEmphasis_3	0.519
wavelet-HLL_gldm_LowGrayLevelEmphasis_3	0.456
wavelet-HLL_gldm_SmallDependenceEmphasis_3	0.354
wavelet-HLL_gldm_SmallDependenceHighGrayLevelEmphasis_3	0.358
wavelet-HLL_gldm_SmallDependenceLowGrayLevelEmphasis_3	0.356
wavelet-HLL_glrlm_GrayLevelNonUniformity_3	0.493
wavelet-HLL_glrlm_GrayLevelNonUniformityNormalized_3	0.480
wavelet-HLL_glrlm_GrayLevelVariance_3	0.480
wavelet-HLL_glrlm_HighGrayLevelRunEmphasis_3	0.442
wavelet-HLL_glrlm_LongRunEmphasis_3	0.438
wavelet-HLL_glrlm_LongRunHighGrayLevelEmphasis_3	0.309
wavelet-HLL_glrlm_LongRunLowGrayLevelEmphasis_3	0.602
wavelet-HLL_glrlm_LowGrayLevelRunEmphasis_3	0.442
wavelet-HLL_glrlm_RunEntropy_3	0.229
wavelet-HLL_glrlm_RunLengthNonUniformity_3	0.519
wavelet-HLL_glrlm_RunLengthNonUniformityNormalized_3	0.274
wavelet-HLL_glrlm_RunPercentage_3	0.330
wavelet-HLL_glrlm_RunVariance_3	0.283
wavelet-HLL_glrlm_ShortRunEmphasis_3	0.342
wavelet-HLL_glrlm_ShortRunHighGrayLevelEmphasis_3	0.604
wavelet-HLL_glrlm_ShortRunLowGrayLevelEmphasis_3	0.221
wavelet-HLL_glszm_GrayLevelNonUniformity_3	0.773
wavelet-HLL_glszm_GrayLevelNonUniformityNormalized_3	0.190
wavelet-HLL_glszm_GrayLevelVariance_3	0.190
wavelet-HLL_glszm_HighGrayLevelZoneEmphasis_3	0.364
wavelet-HLL_glszm_LargeAreaEmphasis_3	0.234
wavelet-HLL_glszm_LargeAreaHighGrayLevelEmphasis_3	0.216
wavelet-HLL_glszm_LargeAreaLowGrayLevelEmphasis_3	0.250
wavelet-HLL_glszm_LowGrayLevelZoneEmphasis_3	0.364
wavelet-HLL_glszm_SizeZoneNonUniformity_3	0.859
wavelet-HLL_glszm_SizeZoneNonUniformityNormalized_3	0.611
wavelet-HLL_glszm_SmallAreaEmphasis_3	0.821
wavelet-HLL_glszm_SmallAreaHighGrayLevelEmphasis_3	0.765
wavelet-HLL_glszm_SmallAreaLowGrayLevelEmphasis_3	0.713
wavelet-HLL_glszm_ZoneEntropy_3	0.480
wavelet-HLL_glszm_ZonePercentage_3	0.254
wavelet-HLL_glszm_ZoneVariance_3	0.235
wavelet-HLL_ngtdm_Busyness_3	0.497
wavelet-HLL_ngtdm_Coarseness_3	0.000
wavelet-HLL_ngtdm_Complexity_3	0.461
wavelet-HLL_ngtdm_Contrast_3	0.459
wavelet-HLL_ngtdm_Strength_3	0.065
wavelet-HLH_firstorder_10Percentile_3	0.828
wavelet-HLH_firstorder_90Percentile_3	0.384
wavelet-HLH_firstorder_Energy_3	0.700
wavelet-HLH_firstorder_Entropy_3	0.237
wavelet-HLH_firstorder_InterquartileRange_3	0.520
wavelet-HLH_firstorder_Kurtosis_3	0.549
wavelet-HLH_firstorder_Maximum_3	0.835

wavelet-HLH_firstorder_MeanAbsoluteDeviation_3	0.593
wavelet-HLH_firstorder_Mean_3	0.207
wavelet-HLH_firstorder_Median_3	0.241
wavelet-HLH_firstorder_Minimum_3	0.886
wavelet-HLH_firstorder_Range_3	0.912
wavelet-HLH_firstorder_RobustMeanAbsoluteDeviation_3	0.505
wavelet-HLH_firstorder_RootMeanSquared_3	0.624
wavelet-HLH_firstorder_Skewness_3	0.279
wavelet-HLH_firstorder_TotalEnergy_3	0.700
wavelet-HLH_firstorder_Uniformity_3	0.269
wavelet-HLH_firstorder_Variance_3	0.591
wavelet-HLH_glcm_Autocorrelation_3	0.386
wavelet-HLH_glcm_ClusterProminence_3	0.308
wavelet-HLH_glcm_ClusterShade_3	0.553
wavelet-HLH_glcm_ClusterTendency_3	0.232
wavelet-HLH_glcm_Contrast_3	0.422
wavelet-HLH_glcm_Correlation_3	0.561
wavelet-HLH_glcm_DifferenceAverage_3	0.422
wavelet-HLH_glcm_DifferenceEntropy_3	0.260
wavelet-HLH_glcm_DifferenceVariance_3	0.317
wavelet-HLH_glcm_Id_3	0.422
wavelet-HLH_glcm_Idm_3	0.422
wavelet-HLH_glcm_Idmn_3	0.422
wavelet-HLH_glcm_Idn_3	0.422
wavelet-HLH_glcm_Imc1_3	0.627
wavelet-HLH_glcm_Imc2_3	0.784
wavelet-HLH_glcm_InverseVariance_3	0.422
wavelet-HLH_glcm_JointAverage_3	0.394
wavelet-HLH_glcm_JointEnergy_3	0.201
wavelet-HLH_glcm_JointEntropy_3	0.212
wavelet-HLH_glcm_MCC_3	0.784
wavelet-HLH_glcm_MaximumProbability_3	0.343
wavelet-HLH_glcm_SumAverage_3	0.394
wavelet-HLH_glcm_SumEntropy_3	0.186
wavelet-HLH_glcm_SumSquares_3	0.180
wavelet-HLH_gldm_DependenceEntropy_3	0.266
wavelet-HLH_gldm_DependenceNonUniformity_3	0.487
wavelet-HLH_gldm_DependenceNonUniformityNormalized_3	0.233
wavelet-HLH_gldm_DependenceVariance_3	0.661
wavelet-HLH_gldm_GrayLevelNonUniformity_3	0.473
wavelet-HLH_gldm_GrayLevelVariance_3	0.269
wavelet-HLH_gldm_HighGrayLevelEmphasis_3	0.445
wavelet-HLH_gldm_LargeDependenceEmphasis_3	0.294
wavelet-HLH_gldm_LargeDependenceHighGrayLevelEmphasis_3	0.381
wavelet-HLH_gldm_LargeDependenceLowGrayLevelEmphasis_3	0.254
wavelet-HLH_gldm_LowGrayLevelEmphasis_3	0.445
wavelet-HLH_gldm_SmallDependenceEmphasis_3	0.330
wavelet-HLH_gldm_SmallDependenceHighGrayLevelEmphasis_3	0.302
wavelet-HLH_gldm_SmallDependenceLowGrayLevelEmphasis_3	0.360
wavelet-HLH_glrlm_GrayLevelNonUniformity_3	0.493

wavelet-HLH_glrlm_GrayLevelNonUniformityNormalized_3	0.211
wavelet-HLH_glrlm_GrayLevelVariance_3	0.211
wavelet-HLH_glrlm_HighGrayLevelRunEmphasis_3	0.413
wavelet-HLH_glrlm_LongRunEmphasis_3	0.424
wavelet-HLH_glrlm_LongRunHighGrayLevelEmphasis_3	0.563
wavelet-HLH_glrlm_LongRunLowGrayLevelEmphasis_3	0.302
wavelet-HLH_glrlm_LowGrayLevelRunEmphasis_3	0.413
wavelet-HLH_glrlm_RunEntropy_3	0.236
wavelet-HLH_glrlm_RunLengthNonUniformity_3	0.521
wavelet-HLH_glrlm_RunLengthNonUniformityNormalized_3	0.281
wavelet-HLH_glrlm_RunPercentage_3	0.287
wavelet-HLH_glrlm_RunVariance_3	0.286
wavelet-HLH_glrlm_ShortRunEmphasis_3	0.305
wavelet-HLH_glrlm_ShortRunHighGrayLevelEmphasis_3	0.153
wavelet-HLH_glrlm_ShortRunLowGrayLevelEmphasis_3	0.541
wavelet-HLH_glszm_GrayLevelNonUniformity_3	0.754
wavelet-HLH_glszm_GrayLevelNonUniformityNormalized_3	0.197
wavelet-HLH_glszm_GrayLevelVariance_3	0.197
wavelet-HLH_glszm_HighGrayLevelZoneEmphasis_3	0.078
wavelet-HLH_glszm_LargeAreaEmphasis_3	0.250
wavelet-HLH_glszm_LargeAreaHighGrayLevelEmphasis_3	0.264
wavelet-HLH_glszm_LargeAreaLowGrayLevelEmphasis_3	0.237
wavelet-HLH_glszm_LowGrayLevelZoneEmphasis_3	0.078
wavelet-HLH_glszm_SizeZoneNonUniformity_3	0.808
wavelet-HLH_glszm_SizeZoneNonUniformityNormalized_3	0.660
wavelet-HLH_glszm_SmallAreaEmphasis_3	0.778
wavelet-HLH_glszm_SmallAreaHighGrayLevelEmphasis_3	0.718
wavelet-HLH_glszm_SmallAreaLowGrayLevelEmphasis_3	0.644
wavelet-HLH_glszm_ZoneEntropy_3	0.460
wavelet-HLH_glszm_ZonePercentage_3	0.270
wavelet-HLH_glszm_ZoneVariance_3	0.252
wavelet-HLH_ngtdm_Busyness_3	0.418
wavelet-HLH_ngtdm_Coarseness_3	0.011
wavelet-HLH_ngtdm_Complexity_3	0.557
wavelet-HLH_ngtdm_Contrast_3	0.564
wavelet-HLH_ngtdm_Strength_3	0.016
wavelet-HHL_firstorder_10Percentile_3	0.916
wavelet-HHL_firstorder_90Percentile_3	0.798
wavelet-HHL_firstorder_Energy_3	0.595
wavelet-HHL_firstorder_Entropy_3	0.324
wavelet-HHL_firstorder_InterquartileRange_3	0.840
wavelet-HHL_firstorder_Kurtosis_3	0.937
wavelet-HHL_firstorder_Maximum_3	0.775
wavelet-HHL_firstorder_MeanAbsoluteDeviation_3	0.850
wavelet-HHL_firstorder_Mean_3	0.626
wavelet-HHL_firstorder_Median_3	0.434
wavelet-HHL_firstorder_Minimum_3	0.931
wavelet-HHL_firstorder_Range_3	0.893
wavelet-HHL_firstorder_RobustMeanAbsoluteDeviation_3	0.861
wavelet-HHL_firstorder_RootMeanSquared_3	0.811

wavelet-HHL_firstorder_Skewness_3	0.293
wavelet-HHL_firstorder_TotalEnergy_3	0.595
wavelet-HHL_firstorder_Uniformity_3	0.326
wavelet-HHL_firstorder_Variance_3	0.812
wavelet-HHL_glcm_Autocorrelation_3	0.576
wavelet-HHL_glcm_ClusterProminence_3	0.073
wavelet-HHL_glcm_ClusterShade_3	0.635
wavelet-HHL_glcm_ClusterTendency_3	0.048
wavelet-HHL_glcm_Contrast_3	0.350
wavelet-HHL_glcm_Correlation_3	0.554
wavelet-HHL_glcm_DifferenceAverage_3	0.350
wavelet-HHL_glcm_DifferenceEntropy_3	0.183
wavelet-HHL_glcm_DifferenceVariance_3	0.223
wavelet-HHL_glcm_Id_3	0.350
wavelet-HHL_glcm_Idm_3	0.350
wavelet-HHL_glcm_Idmn_3	0.350
wavelet-HHL_glcm_Idn_3	0.350
wavelet-HHL_glcm_Imc1_3	0.231
wavelet-HHL_glcm_Imc2_3	0.328
wavelet-HHL_glcm_InverseVariance_3	0.350
wavelet-HHL_glcm_JointAverage_3	0.556
wavelet-HHL_glcm_JointEnergy_3	0.089
wavelet-HHL_glcm_JointEntropy_3	0.080
wavelet-HHL_glcm_MCC_3	0.343
wavelet-HHL_glcm_MaximumProbability_3	0.236
wavelet-HHL_glcm_SumAverage_3	0.556
wavelet-HHL_glcm_SumEntropy_3	0.026
wavelet-HHL_glcm_SumSquares_3	0.037
wavelet-HHL_gldm_DependenceEntropy_3	0.240
wavelet-HHL_gldm_DependenceNonUniformity_3	0.495
wavelet-HHL_gldm_DependenceNonUniformityNormalized_3	0.218
wavelet-HHL_gldm_DependenceVariance_3	0.385
wavelet-HHL_gldm_GrayLevelNonUniformity_3	0.473
wavelet-HHL_gldm_GrayLevelVariance_3	0.326
wavelet-HHL_gldm_HighGrayLevelEmphasis_3	0.474
wavelet-HHL_gldm_LargeDependenceEmphasis_3	0.183
wavelet-HHL_gldm_LargeDependenceHighGrayLevelEmphasis_3	0.148
wavelet-HHL_gldm_LargeDependenceLowGrayLevelEmphasis_3	0.231
wavelet-HHL_gldm_LowGrayLevelEmphasis_3	0.474
wavelet-HHL_gldm_SmallDependenceEmphasis_3	0.376
wavelet-HHL_gldm_SmallDependenceHighGrayLevelEmphasis_3	0.302
wavelet-HHL_gldm_SmallDependenceLowGrayLevelEmphasis_3	0.451
wavelet-HHL_glrlm_GrayLevelNonUniformity_3	0.494
wavelet-HHL_glrlm_GrayLevelNonUniformityNormalized_3	0.245
wavelet-HHL_glrlm_GrayLevelVariance_3	0.245
wavelet-HHL_glrlm_HighGrayLevelRunEmphasis_3	0.453
wavelet-HHL_glrlm_LongRunEmphasis_3	0.424
wavelet-HHL_glrlm_LongRunHighGrayLevelEmphasis_3	0.412
wavelet-HHL_glrlm_LongRunLowGrayLevelEmphasis_3	0.436
wavelet-HHL_glrlm_LowGrayLevelRunEmphasis_3	0.453

wavelet-HHL_glrlm_RunEntropy_3	0.197
wavelet-HHL_glrlm_RunLengthNonUniformity_3	0.526
wavelet-HHL_glrlm_RunLengthNonUniformityNormalized_3	0.210
wavelet-HHL_glrlm_RunPercentage_3	0.212
wavelet-HHL_glrlm_RunVariance_3	0.264
wavelet-HHL_glrlm_ShortRunEmphasis_3	0.250
wavelet-HHL_glrlm_ShortRunHighGrayLevelEmphasis_3	0.249
wavelet-HHL_glrlm_ShortRunLowGrayLevelEmphasis_3	0.285
wavelet-HHL_glszm_GrayLevelNonUniformity_3	0.740
wavelet-HHL_glszm_GrayLevelNonUniformityNormalized_3	0.053
wavelet-HHL_glszm_GrayLevelVariance_3	0.053
wavelet-HHL_glszm_HighGrayLevelZoneEmphasis_3	0.034
wavelet-HHL_glszm_LargeAreaEmphasis_3	0.251
wavelet-HHL_glszm_LargeAreaHighGrayLevelEmphasis_3	0.264
wavelet-HHL_glszm_LargeAreaLowGrayLevelEmphasis_3	0.239
wavelet-HHL_glszm_LowGrayLevelZoneEmphasis_3	0.034
wavelet-HHL_glszm_SizeZoneNonUniformity_3	0.813
wavelet-HHL_glszm_SizeZoneNonUniformityNormalized_3	0.560
wavelet-HHL_glszm_SmallAreaEmphasis_3	0.710
wavelet-HHL_glszm_SmallAreaHighGrayLevelEmphasis_3	0.686
wavelet-HHL_glszm_SmallAreaLowGrayLevelEmphasis_3	0.578
wavelet-HHL_glszm_ZoneEntropy_3	0.479
wavelet-HHL_glszm_ZonePercentage_3	0.320
wavelet-HHL_glszm_ZoneVariance_3	0.253
wavelet-HHL_ngtdm_Busyness_3	0.441
wavelet-HHL_ngtdm_Coarseness_3	0.022
wavelet-HHL_ngtdm_Complexity_3	0.496
wavelet-HHL_ngtdm_Contrast_3	0.489
wavelet-HHL_ngtdm_Strength_3	0.023
wavelet-HHH_firstorder_10Percentile_3	0.954
wavelet-HHH_firstorder_90Percentile_3	0.511
wavelet-HHH_firstorder_Energy_3	0.672
wavelet-HHH_firstorder_Entropy_3	0.002
wavelet-HHH_firstorder_InterquartileRange_3	0.926
wavelet-HHH_firstorder_Kurtosis_3	0.910
wavelet-HHH_firstorder_Maximum_3	0.918
wavelet-HHH_firstorder_MeanAbsoluteDeviation_3	0.765
wavelet-HHH_firstorder_Mean_3	0.045
wavelet-HHH_firstorder_Median_3	0.249
wavelet-HHH_firstorder_Minimum_3	0.912
wavelet-HHH_firstorder_Range_3	0.946
wavelet-HHH_firstorder_RobustMeanAbsoluteDeviation_3	0.832
wavelet-HHH_firstorder_RootMeanSquared_3	0.701
wavelet-HHH_firstorder_Skewness_3	0.388
wavelet-HHH_firstorder_TotalEnergy_3	0.672
wavelet-HHH_firstorder_Uniformity_3	0.002
wavelet-HHH_firstorder_Variance_3	0.593
wavelet-HHH_glcm_Autocorrelation_3	0.033
wavelet-HHH_glcm_ClusterProminence_3	0.213
wavelet-HHH_glcm_ClusterShade_3	0.266

wavelet-HHH_glcm_ClusterTendency_3	0.181
wavelet-HHH_glcm_Contrast_3	0.263
wavelet-HHH_glcm_Correlation_3	0.444
wavelet-HHH_glcm_DifferenceAverage_3	0.263
wavelet-HHH_glcm_DifferenceEntropy_3	0.168
wavelet-HHH_glcm_DifferenceVariance_3	0.212
wavelet-HHH_glcm_Id_3	0.263
wavelet-HHH_glcm_Idm_3	0.263
wavelet-HHH_glcm_Idmn_3	0.263
wavelet-HHH_glcm_Idn_3	0.263
wavelet-HHH_glcm_Imc1_3	0.302
wavelet-HHH_glcm_Imc2_3	0.541
wavelet-HHH_glcm_InverseVariance_3	0.263
wavelet-HHH_glcm_JointAverage_3	0.082
wavelet-HHH_glcm_JointEnergy_3	0.050
wavelet-HHH_glcm_JointEntropy_3	0.051
wavelet-HHH_glcm_MCC_3	0.546
wavelet-HHH_glcm_MaximumProbability_3	0.145
wavelet-HHH_glcm_SumAverage_3	0.082
wavelet-HHH_glcm_SumEntropy_3	0.035
wavelet-HHH_glcm_SumSquares_3	0.003
wavelet-HHH_gldm_DependenceEntropy_3	0.217
wavelet-HHH_gldm_DependenceNonUniformity_3	0.489
wavelet-HHH_gldm_DependenceNonUniformityNormalized_3	0.212
wavelet-HHH_gldm_DependenceVariance_3	0.397
wavelet-HHH_gldm_GrayLevelNonUniformity_3	0.473
wavelet-HHH_gldm_GrayLevelVariance_3	0.002
wavelet-HHH_gldm_HighGrayLevelEmphasis_3	0.212
wavelet-HHH_gldm_LargeDependenceEmphasis_3	0.186
wavelet-HHH_gldm_LargeDependenceHighGrayLevelEmphasis_3	0.194
wavelet-HHH_gldm_LargeDependenceLowGrayLevelEmphasis_3	0.181
wavelet-HHH_gldm_LowGrayLevelEmphasis_3	0.212
wavelet-HHH_gldm_SmallDependenceEmphasis_3	0.340
wavelet-HHH_gldm_SmallDependenceHighGrayLevelEmphasis_3	0.388
wavelet-HHH_gldm_SmallDependenceLowGrayLevelEmphasis_3	0.290
wavelet-HHH_glrlm_GrayLevelNonUniformity_3	0.494
wavelet-HHH_glrlm_GrayLevelNonUniformityNormalized_3	0.008
wavelet-HHH_glrlm_GrayLevelVariance_3	0.008
wavelet-HHH_glrlm_HighGrayLevelRunEmphasis_3	0.235
wavelet-HHH_glrlm_LongRunEmphasis_3	0.410
wavelet-HHH_glrlm_LongRunHighGrayLevelEmphasis_3	0.388
wavelet-HHH_glrlm_LongRunLowGrayLevelEmphasis_3	0.427
wavelet-HHH_glrlm_LowGrayLevelRunEmphasis_3	0.235
wavelet-HHH_glrlm_RunEntropy_3	0.207
wavelet-HHH_glrlm_RunLengthNonUniformity_3	0.526
wavelet-HHH_glrlm_RunLengthNonUniformityNormalized_3	0.225
wavelet-HHH_glrlm_RunPercentage_3	0.213
wavelet-HHH_glrlm_RunVariance_3	0.256
wavelet-HHH_glrlm_ShortRunEmphasis_3	0.238
wavelet-HHH_glrlm_ShortRunHighGrayLevelEmphasis_3	0.317

wavelet-HHH_glrlm_ShortRunLowGrayLevelEmphasis_3	0.128
wavelet-HHH_glszm_GrayLevelNonUniformity_3	0.719
wavelet-HHH_glszm_GrayLevelNonUniformityNormalized_3	0.045
wavelet-HHH_glszm_GrayLevelVariance_3	0.045
wavelet-HHH_glszm_HighGrayLevelZoneEmphasis_3	0.003
wavelet-HHH_glszm_LargeAreaEmphasis_3	0.263
wavelet-HHH_glszm_LargeAreaHighGrayLevelEmphasis_3	0.256
wavelet-HHH_glszm_LargeAreaLowGrayLevelEmphasis_3	0.270
wavelet-HHH_glszm_LowGrayLevelZoneEmphasis_3	0.003
wavelet-HHH_glszm_SizeZoneNonUniformity_3	0.789
wavelet-HHH_glszm_SizeZoneNonUniformityNormalized_3	0.650
wavelet-HHH_glszm_SmallAreaEmphasis_3	0.733
wavelet-HHH_glszm_SmallAreaHighGrayLevelEmphasis_3	0.532
wavelet-HHH_glszm_SmallAreaLowGrayLevelEmphasis_3	0.728
wavelet-HHH_glszm_ZoneEntropy_3	0.522
wavelet-HHH_glszm_ZonePercentage_3	0.296
wavelet-HHH_glszm_ZoneVariance_3	0.265
wavelet-HHH_ngtdm_Busyness_3	0.474
wavelet-HHH_ngtdm_Coarseness_3	0.046
wavelet-HHH_ngtdm_Complexity_3	0.507
wavelet-HHH_ngtdm_Contrast_3	0.459
wavelet-HHH_ngtdm_Strength_3	0.044
wavelet-LLL_firstorder_10Percentile_3	0.780
wavelet-LLL_firstorder_90Percentile_3	0.966
wavelet-LLL_firstorder_Energy_3	0.439
wavelet-LLL_firstorder_Entropy_3	0.928
wavelet-LLL_firstorder_InterquartileRange_3	0.659
wavelet-LLL_firstorder_Kurtosis_3	0.512
wavelet-LLL_firstorder_Maximum_3	0.964
wavelet-LLL_firstorder_MeanAbsoluteDeviation_3	0.669
wavelet-LLL_firstorder_Mean_3	0.873
wavelet-LLL_firstorder_Median_3	0.872
wavelet-LLL_firstorder_Minimum_3	0.660
wavelet-LLL_firstorder_Range_3	0.850
wavelet-LLL_firstorder_RobustMeanAbsoluteDeviation_3	0.676
wavelet-LLL_firstorder_RootMeanSquared_3	0.885
wavelet-LLL_firstorder_Skewness_3	0.679
wavelet-LLL_firstorder_TotalEnergy_3	0.439
wavelet-LLL_firstorder_Uniformity_3	0.891
wavelet-LLL_firstorder_Variance_3	0.705
wavelet-LLL_glcm_Autocorrelation_3	0.672
wavelet-LLL_glcm_ClusterProminence_3	0.864
wavelet-LLL_glcm_ClusterShade_3	0.909
wavelet-LLL_glcm_ClusterTendency_3	0.906
wavelet-LLL_glcm_Contrast_3	0.940
wavelet-LLL_glcm_Correlation_3	0.852
wavelet-LLL_glcm_DifferenceAverage_3	0.940
wavelet-LLL_glcm_DifferenceEntropy_3	0.982
wavelet-LLL_glcm_DifferenceVariance_3	0.970
wavelet-LLL_glcm_Id_3	0.940

wavelet-LLL_glcm_Idm_3	0.940
wavelet-LLL_glcm_Idmn_3	0.940
wavelet-LLL_glcm_Idn_3	0.940
wavelet-LLL_glcm_Imc1_3	0.792
wavelet-LLL_glcm_Imc2_3	0.942
wavelet-LLL_glcm_InverseVariance_3	0.940
wavelet-LLL_glcm_JointAverage_3	0.689
wavelet-LLL_glcm_JointEnergy_3	0.945
wavelet-LLL_glcm_JointEntropy_3	0.957
wavelet-LLL_glcm_MCC_3	0.732
wavelet-LLL_glcm_MaximumProbability_3	0.921
wavelet-LLL_glcm_SumAverage_3	0.689
wavelet-LLL_glcm_SumEntropy_3	0.959
wavelet-LLL_glcm_SumSquares_3	0.924
wavelet-LLL_gldm_DependenceEntropy_3	0.258
wavelet-LLL_gldm_DependenceNonUniformity_3	0.427
wavelet-LLL_gldm_DependenceNonUniformityNormalized_3	0.157
wavelet-LLL_gldm_DependenceVariance_3	0.375
wavelet-LLL_gldm_GrayLevelNonUniformity_3	0.473
wavelet-LLL_gldm_GrayLevelVariance_3	0.891
wavelet-LLL_gldm_HighGrayLevelEmphasis_3	0.707
wavelet-LLL_gldm_LargeDependenceEmphasis_3	0.188
wavelet-LLL_gldm_LargeDependenceHighGrayLevelEmphasis_3	0.201
wavelet-LLL_gldm_LargeDependenceLowGrayLevelEmphasis_3	0.228
wavelet-LLL_gldm_LowGrayLevelEmphasis_3	0.707
wavelet-LLL_gldm_SmallDependenceEmphasis_3	0.279
wavelet-LLL_gldm_SmallDependenceHighGrayLevelEmphasis_3	0.266
wavelet-LLL_gldm_SmallDependenceLowGrayLevelEmphasis_3	0.306
wavelet-LLL_glrlm_GrayLevelNonUniformity_3	0.528
wavelet-LLL_glrlm_GrayLevelNonUniformityNormalized_3	0.910
wavelet-LLL_glrlm_GrayLevelVariance_3	0.910
wavelet-LLL_glrlm_HighGrayLevelRunEmphasis_3	0.697
wavelet-LLL_glrlm_LongRunEmphasis_3	0.095
wavelet-LLL_glrlm_LongRunHighGrayLevelEmphasis_3	0.095
wavelet-LLL_glrlm_LongRunLowGrayLevelEmphasis_3	0.104
wavelet-LLL_glrlm_LowGrayLevelRunEmphasis_3	0.697
wavelet-LLL_glrlm_RunEntropy_3	0.156
wavelet-LLL_glrlm_RunLengthNonUniformity_3	0.573
wavelet-LLL_glrlm_RunLengthNonUniformityNormalized_3	0.238
wavelet-LLL_glrlm_RunPercentage_3	0.227
wavelet-LLL_glrlm_RunVariance_3	0.068
wavelet-LLL_glrlm_ShortRunEmphasis_3	0.260
wavelet-LLL_glrlm_ShortRunHighGrayLevelEmphasis_3	0.594
wavelet-LLL_glrlm_ShortRunLowGrayLevelEmphasis_3	0.384
wavelet-LLL_glszm_GrayLevelNonUniformity_3	0.558
wavelet-LLL_glszm_GrayLevelNonUniformityNormalized_3	0.847
wavelet-LLL_glszm_GrayLevelVariance_3	0.847
wavelet-LLL_glszm_HighGrayLevelZoneEmphasis_3	0.644
wavelet-LLL_glszm_LargeAreaEmphasis_3	0.312
wavelet-LLL_glszm_LargeAreaHighGrayLevelEmphasis_3	0.312

wavelet-LLL_glszm_LargeAreaLowGrayLevelEmphasis_3	0.312
wavelet-LLL_glszm_LowGrayLevelZoneEmphasis_3	0.644
wavelet-LLL_glszm_SizeZoneNonUniformity_3	0.508
wavelet-LLL_glszm_SizeZoneNonUniformityNormalized_3	0.169
wavelet-LLL_glszm_SmallAreaEmphasis_3	0.640
wavelet-LLL_glszm_SmallAreaHighGrayLevelEmphasis_3	0.632
wavelet-LLL_glszm_SmallAreaLowGrayLevelEmphasis_3	0.641
wavelet-LLL_glszm_ZoneEntropy_3	0.339
wavelet-LLL_glszm_ZonePercentage_3	0.155
wavelet-LLL_glszm_ZoneVariance_3	0.327
wavelet-LLL_ngtdm_Busyness_3	0.775
wavelet-LLL_ngtdm_Coarseness_3	0.794
wavelet-LLL_ngtdm_Complexity_3	0.899
wavelet-LLL_ngtdm_Contrast_3	0.701
wavelet-LLL_ngtdm_Strength_3	0.244
