# Supplementary material for: Incremental diagnostic value of tumor habitat radiomics for risk stratification in thymic epithelial tumors
Source: Front Oncol. 2025 Sep 3;15:1630485. doi: 10.3389/fonc.2025.1630485 (PMC12440988; doi:10.3389/fonc.2025.1630485)
Supplement: Supplementary file 3 [file DataSheet3.pdf]

**Radiomics (n=28):**

1. original_shape_Sphericity
2. wavelet-LLH_firstorder_MeanAbsoluteDeviation
3. lbp-3D-k_firstorder_Skewness
4. gradient_firstorder_Kurtosis
5. lbp-2D_glrlm_ShortRunHighGrayLevelEmphasis
6. lbp-3D-m1_glszm_ZonePercentage
7. wavelet-LLH_firstorder_Mean
8. lbp-3D-k_glszm_ZonePercentage
9. wavelet-LLH_firstorder_Kurtosis
10. lbp-3D-k_gldm_SmallDependenceHighGrayLevelEmphasis
11. wavelet-LHL_glszm_GrayLevelNonUniformityNormalized
12. lbp-3D-m1_gldm_SmallDependenceLowGrayLevelEmphasis
13. lbp-3D-k_firstorder_Range
14. wavelet-LHL_glcm_ClusterTendency
15. wavelet-LHL_glcm_JointEnergy
16. wavelet-LLH_gldm_DependenceNonUniformityNormalized
17. wavelet-LLH_firstorder_RobustMeanAbsoluteDeviation
18. wavelet-LLH_glcm_JointEntropy
19. lbp-2D_glrlm_RunLengthNonUniformityNormalized
20. wavelet-HLL_firstorder_Median
21. lbp-3D-m2_glcm_DifferenceVariance
22. lbp-3D-m1_glrlm_RunPercentage
23. wavelet-HLH_glszm_SmallAreaHighGrayLevelEmphasis
24. lbp-3D-m1_glrlm_RunLengthNonUniformityNormalized
25. wavelet-LLH_firstorder_Entropy
26. original_shape_Flatness
27. wavelet-HLL_glrlm_HighGrayLevelRunEmphasis
28. wavelet-LHL_glszm_SmallAreaHighGrayLevelEmphasis

**Habitat (n=40):**

1. lbp-3D-m1_glcm_InverseVariance_3
2. wavelet-LLH_glcm_SumEntropy_2
3. lbp-3D-k_firstorder_Kurtosis_1
4. wavelet-LLH_ngtdm_Contrast_1
5. wavelet-LLH_firstorder_MeanAbsoluteDeviation_3
6. squareroot_firstorder_Kurtosis_3

7. square_firstorder_90Percentile_1
8. wavelet-LLH_firstorder_RobustMeanAbsoluteDeviation_3
9. wavelet-LHL_firstorder_Energy_1
10. square_firstorder_Median_1
11. wavelet-LLH_firstorder_90Percentile_3
12. wavelet-LLH_firstorder_Energy_3
13. wavelet-LLH_glcm_JointEntropy_2
14. original_shape_Flatness_2
15. gradient_firstorder_Energy_1
16. square_firstorder_10Percentile_1
17. lbp-3D-m1_gldm_LargeDependenceEmphasis_3
18. wavelet-LLH_firstorder_Energy_1
19. logarithm_firstorder_10Percentile_2
20. wavelet-LLH_firstorder_Energy_2
21. lbp-3D-m2_glszm_SizeZoneNonUniformityNormalized_3
22. exponential_firstorder_Skewness_3
23. wavelet-LLH_glcm_SumSquares_2
24. logarithm_firstorder_Mean_2
25. wavelet-HLH_firstorder_Maximum_1
26. lbp-3D-m1_glrlm_LongRunEmphasis_1
27. wavelet-HLL_glszm_SmallAreaEmphasis_3
28. lbp-3D-k_ngtdm_Coarseness_2
29. original_firstorder_90Percentile_1
30. lbp-3D-m2_glrlm_RunVariance_2
31. original_firstorder_Maximum_1
32. square_firstorder_Mean_3
33. wavelet-LHL_glszm_SmallAreaEmphasis_3
34. exponential_firstorder_Range_2
35. lbp-3D-k_firstorder_Minimum_1
36. original_shape_Flatness_1
37. lbp-3D-m1_glszm_SizeZoneNonUniformityNormalized_1
38. original_shape_LeastAxisLength_2
39. lbp-3D-m2_glrlm_LongRunEmphasis_1
40. wavelet-LHL_firstorder_Maximum_2

**Combind (n=16):**

1. original_shape_Sphericity

2. lbp-3D-m1_gldm_GrayLevelVariance
3. lbp-3D-m1_gldm_SmallDependenceLowGrayLevelEmphasis
4. lbp-3D-m1_glszm_ZonePercentage
5. lbp-3D-m2_glcm_DifferenceVariance
6. squareroot_firstorder_InterquartileRange
7. wavelet-HLL_glcm_Correlation
8. original_shape_Maximum2DDiameterSlice_1
9. wavelet-HHL_firstorder_10Percentile_1
10. wavelet-HHH_glcm_DifferenceEntropy_1
11. wavelet-LLH_glcm_SumSquares_2
12. wavelet-LHH_glcm_ClusterTendency_2
13. lbp-3D-k_ngtdm_Strength_3
14. wavelet-LHL_glszm_SmallAreaEmphasis_3
15. wavelet-LHH_firstorder_RobustMeanAbsoluteDeviation_3
16. wavelet-HHH_firstorder_Range_3
